# Supplementary figures and images for: 7,8-Dihydroxyflavone modulates bone formation and resorption and ameliorates ovariectomy-induced osteoporosis (part 1 of 2)
Source: eLife. 2021 Jul 6;10:e64872. doi: 10.7554/eLife.64872 (PMC8285109; doi:10.7554/eLife.64872)

## 7,8-DHF: Control (0)

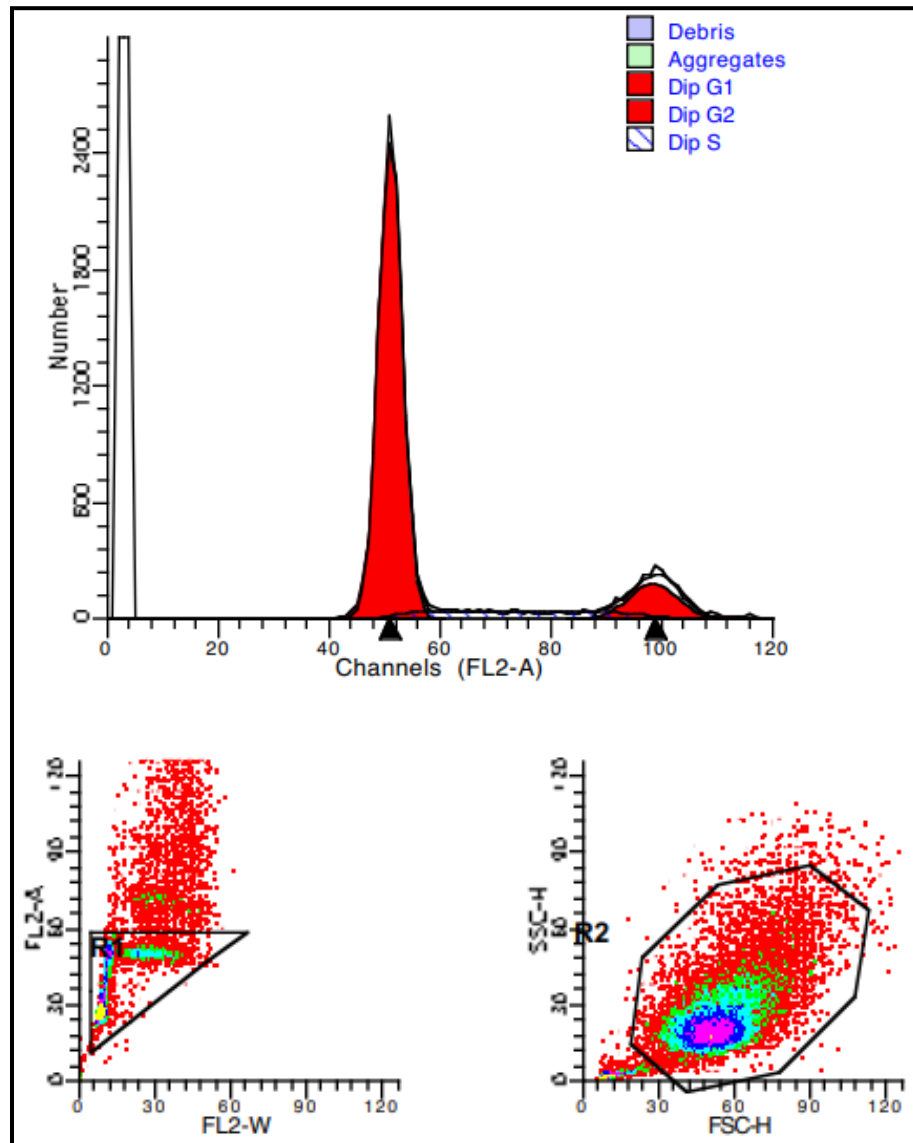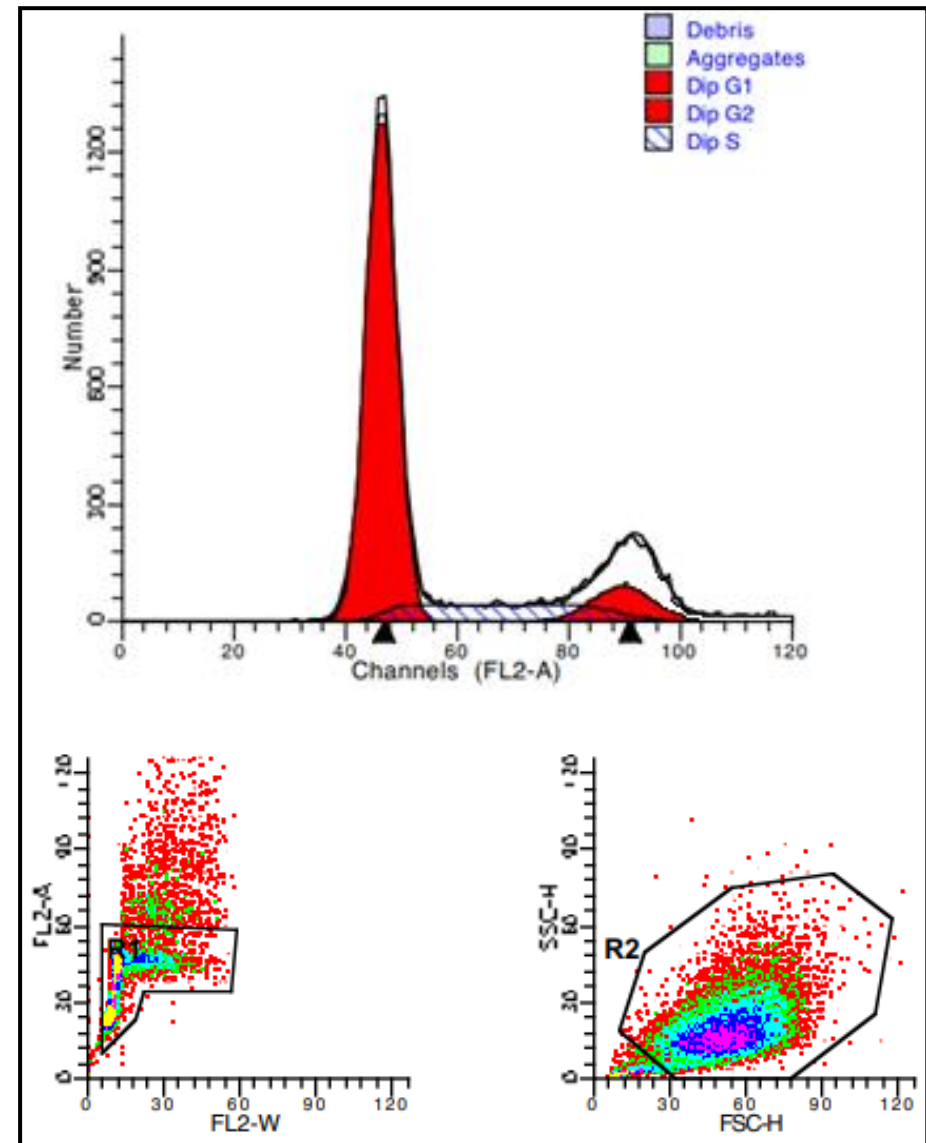

## 7,8-DHF: 0.5 $\mu$ M

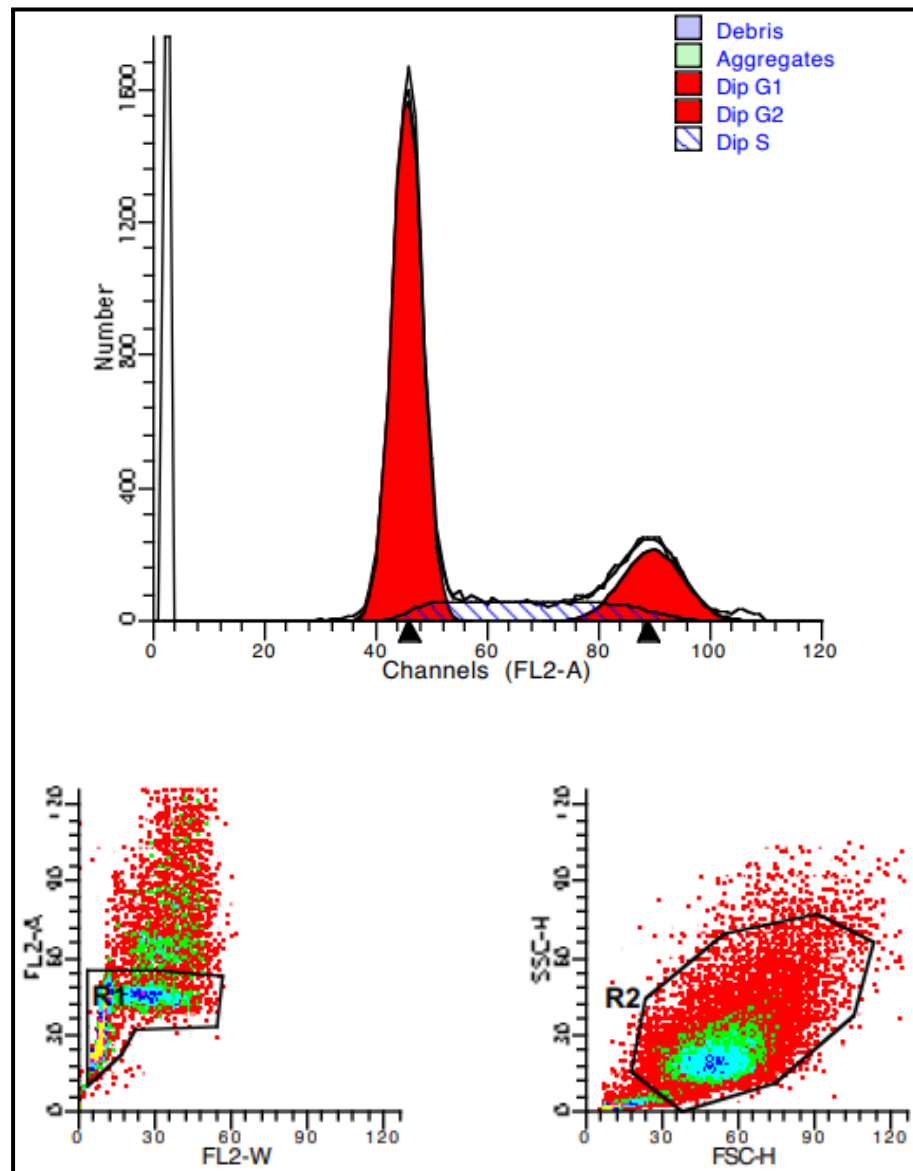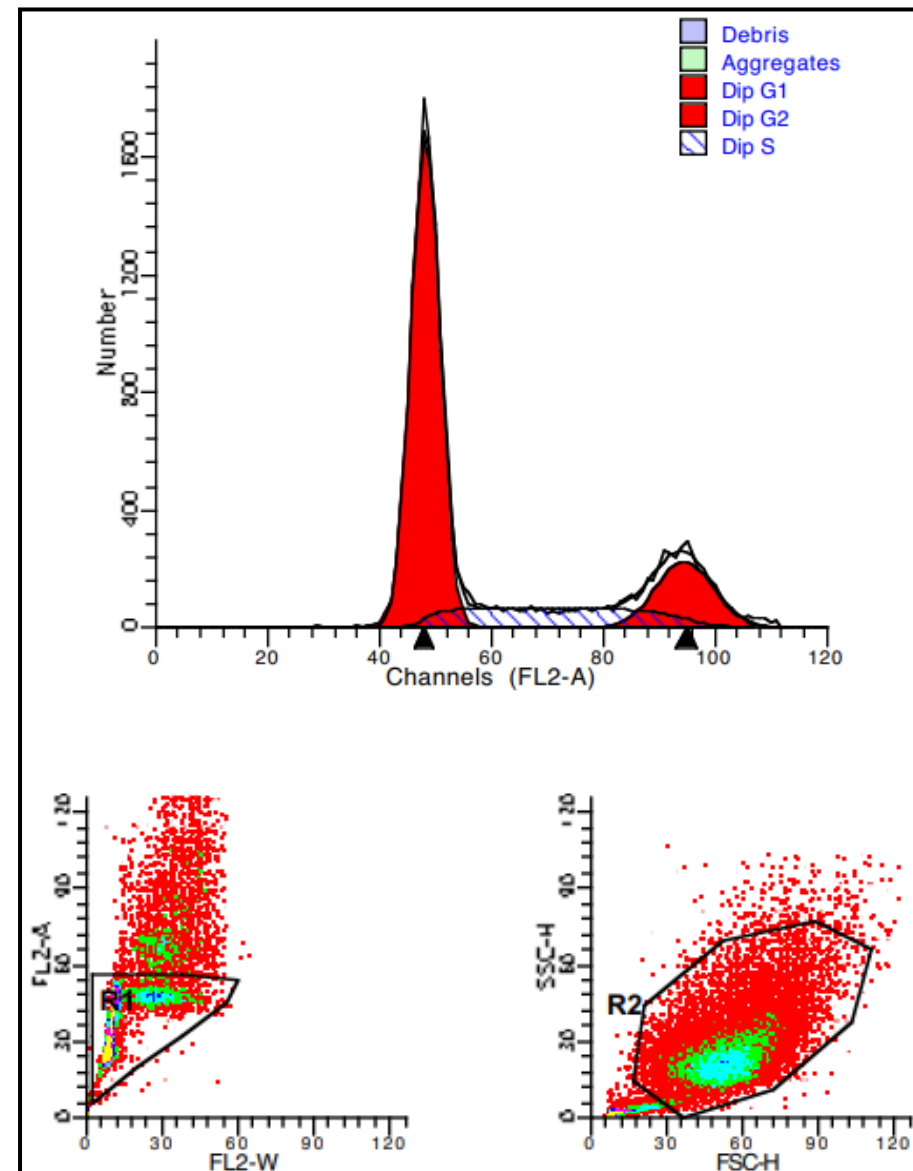

# 7,8-DHF: 1 $\mu$ M

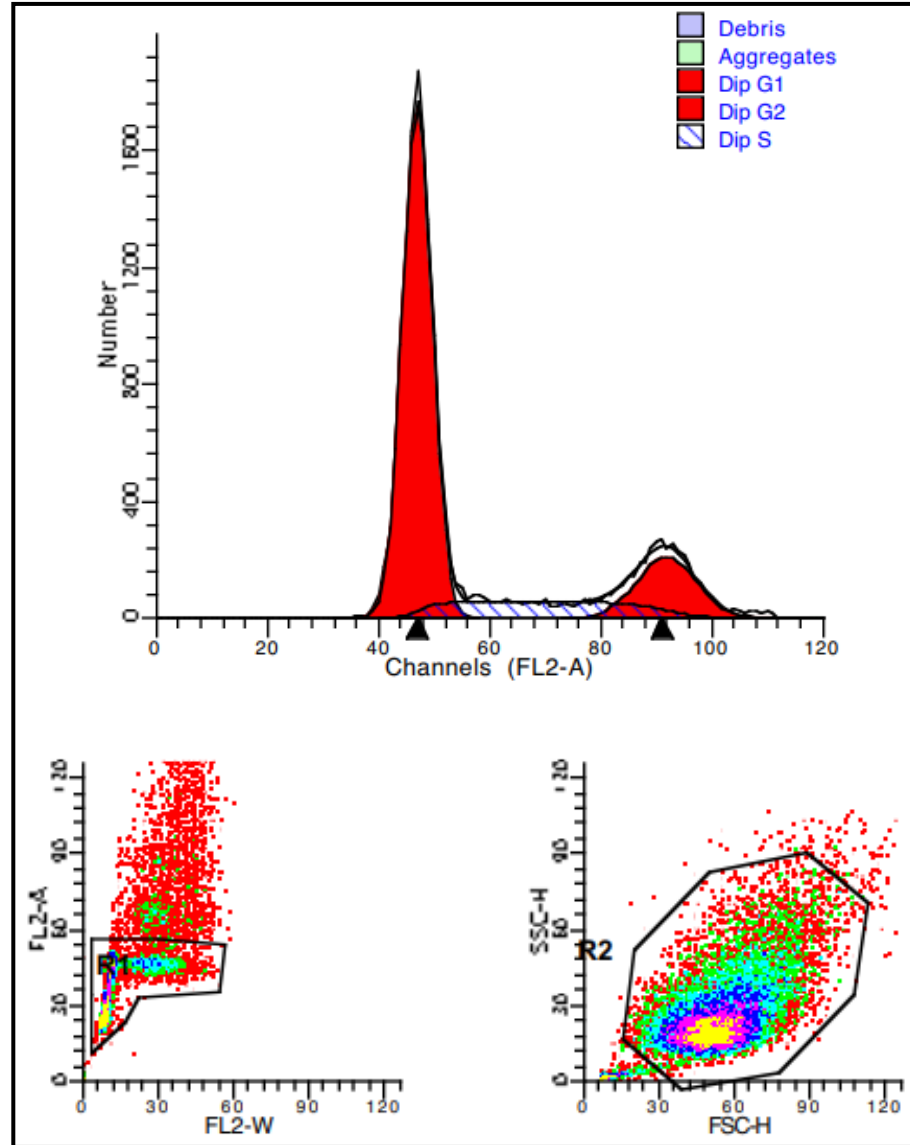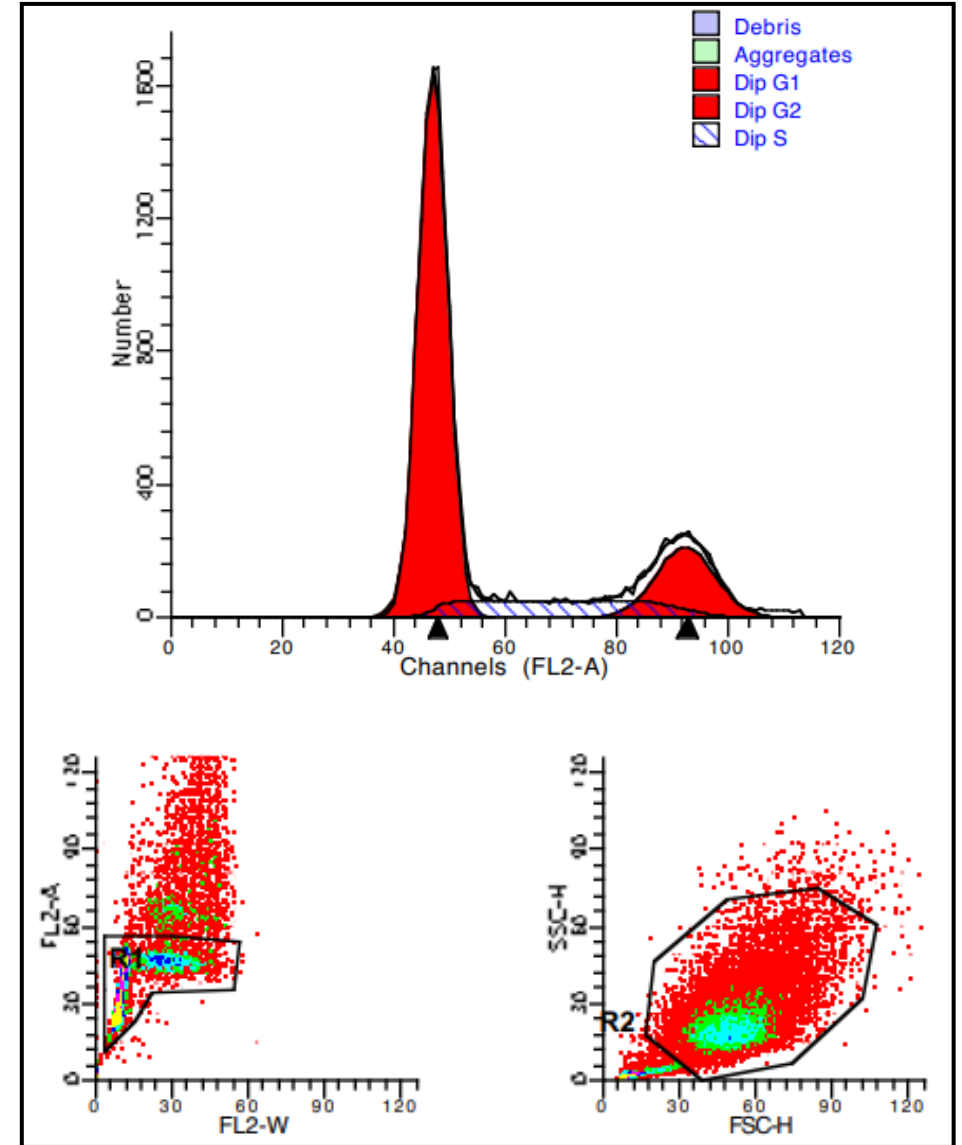

## 7,8-DHF: 5 $\mu$ M

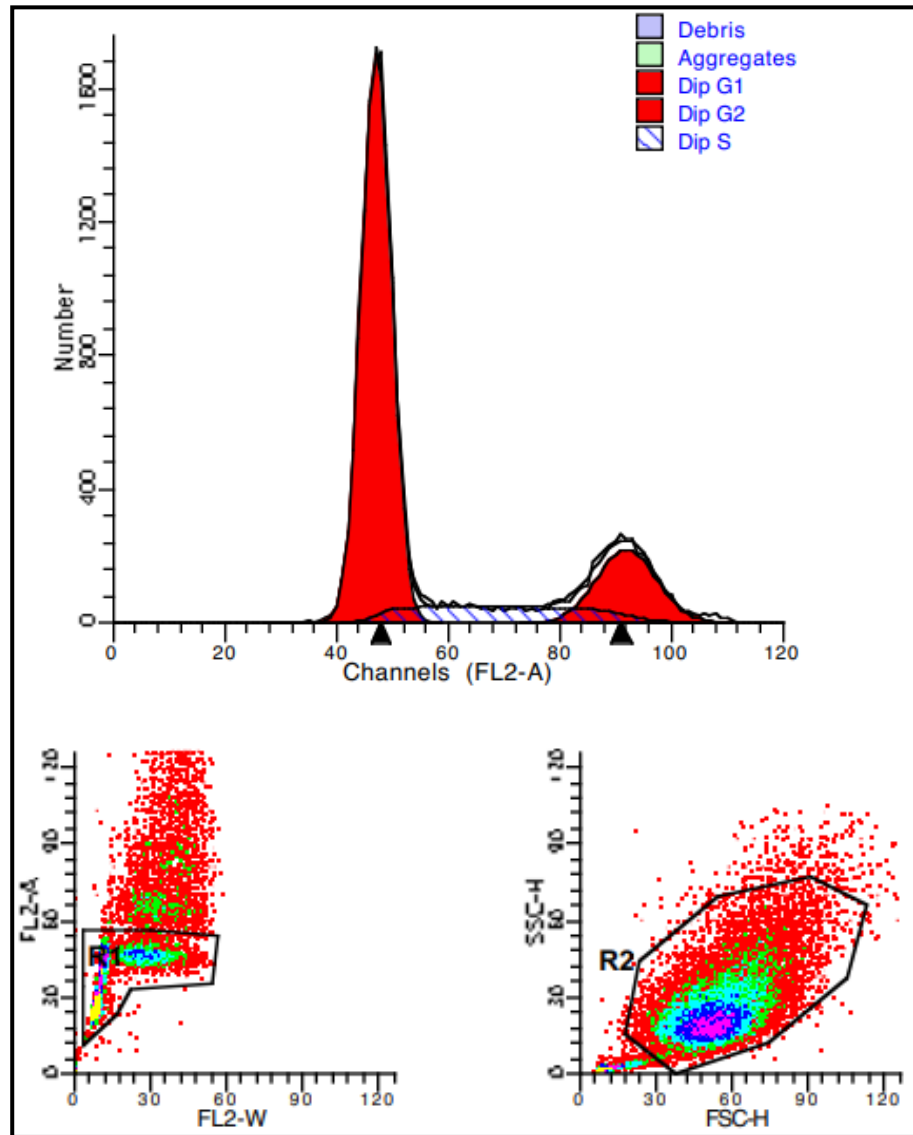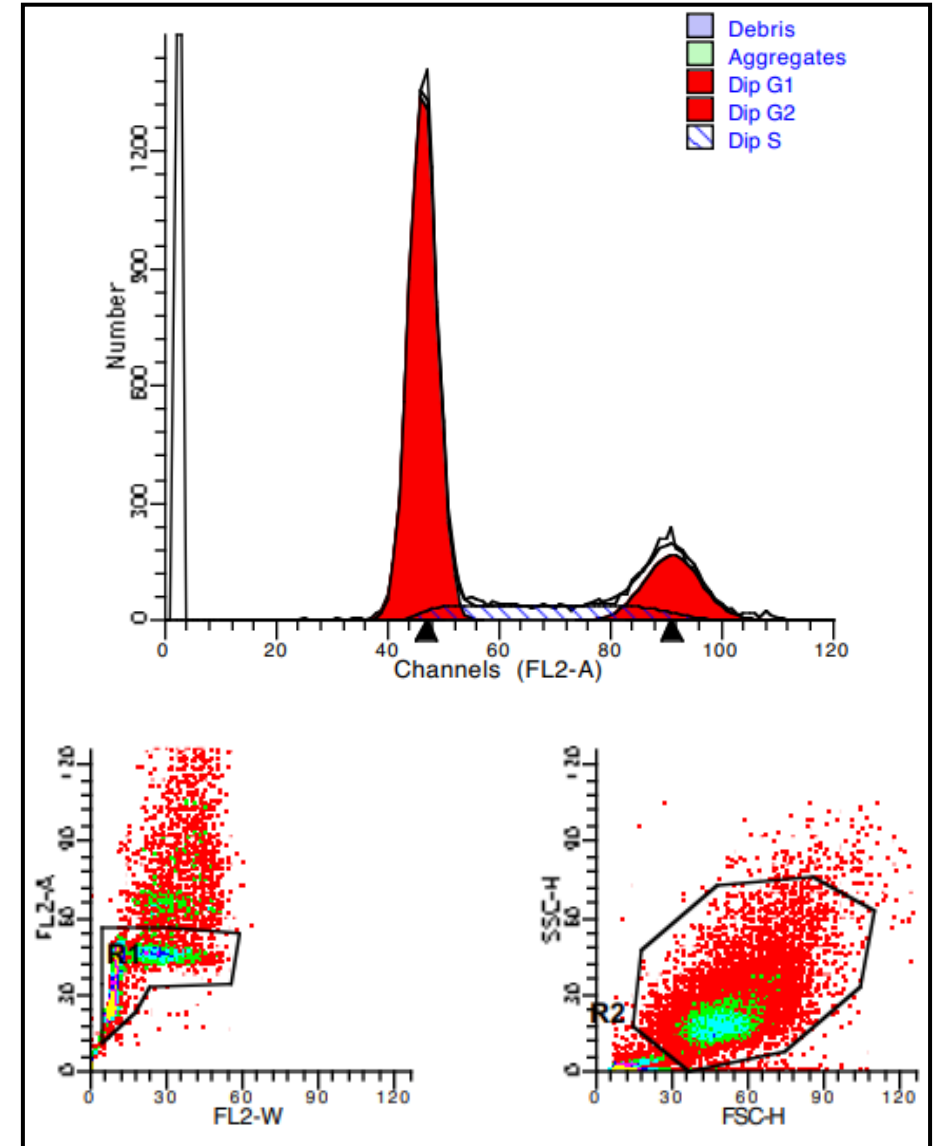

Supplement: Figure 1—source data 1. [file elife-64872-fig1-data1.pdf]

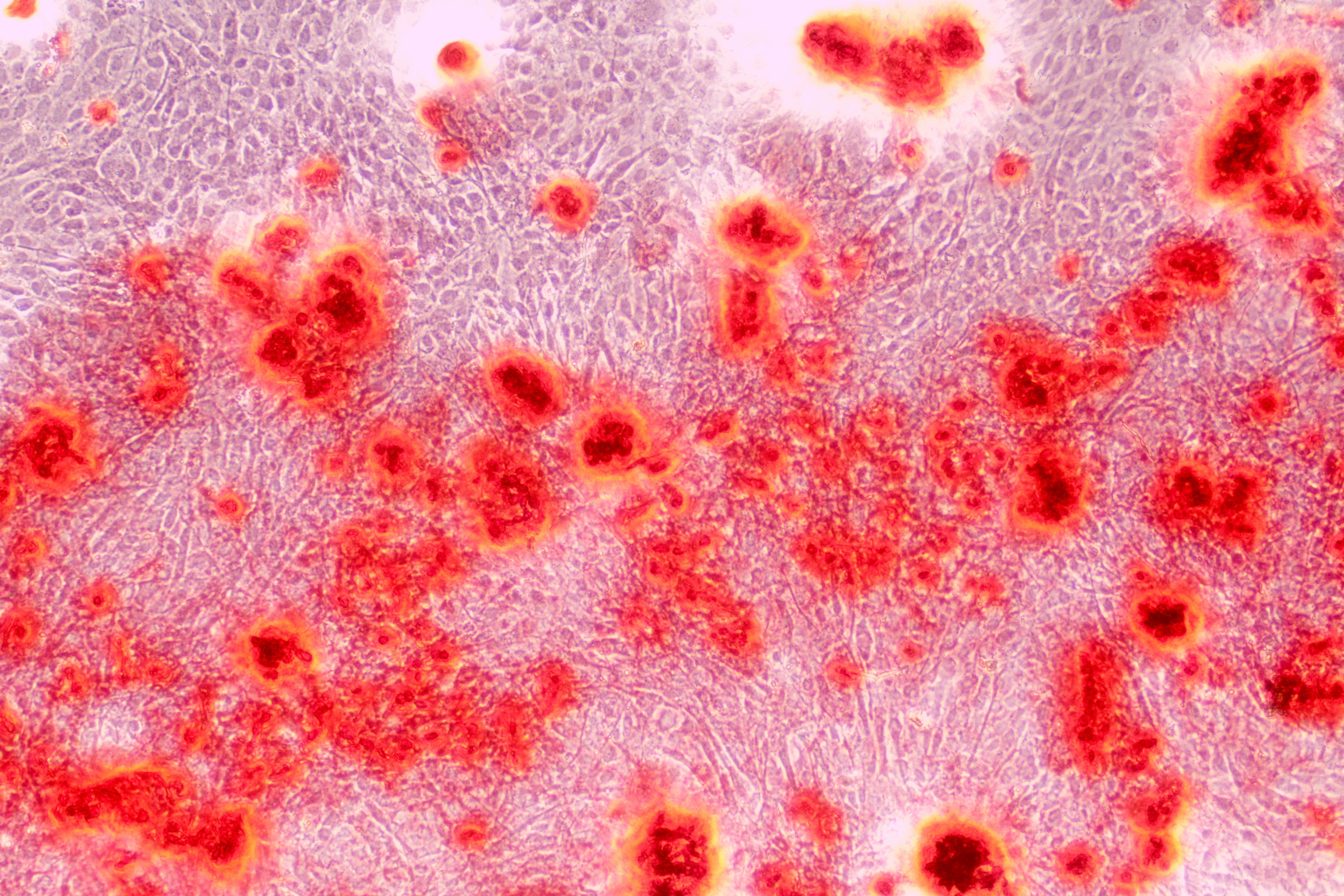

Supplement: Figure 1—source data 2. — The folder named ‘40×’ contains the images at a magnification of 40. The folder named ‘100×’ contains the images at a magnification of 100. [file elife-64872-fig1-data2.zip › Figure 1-source data 2/100×/0.5μM.png]

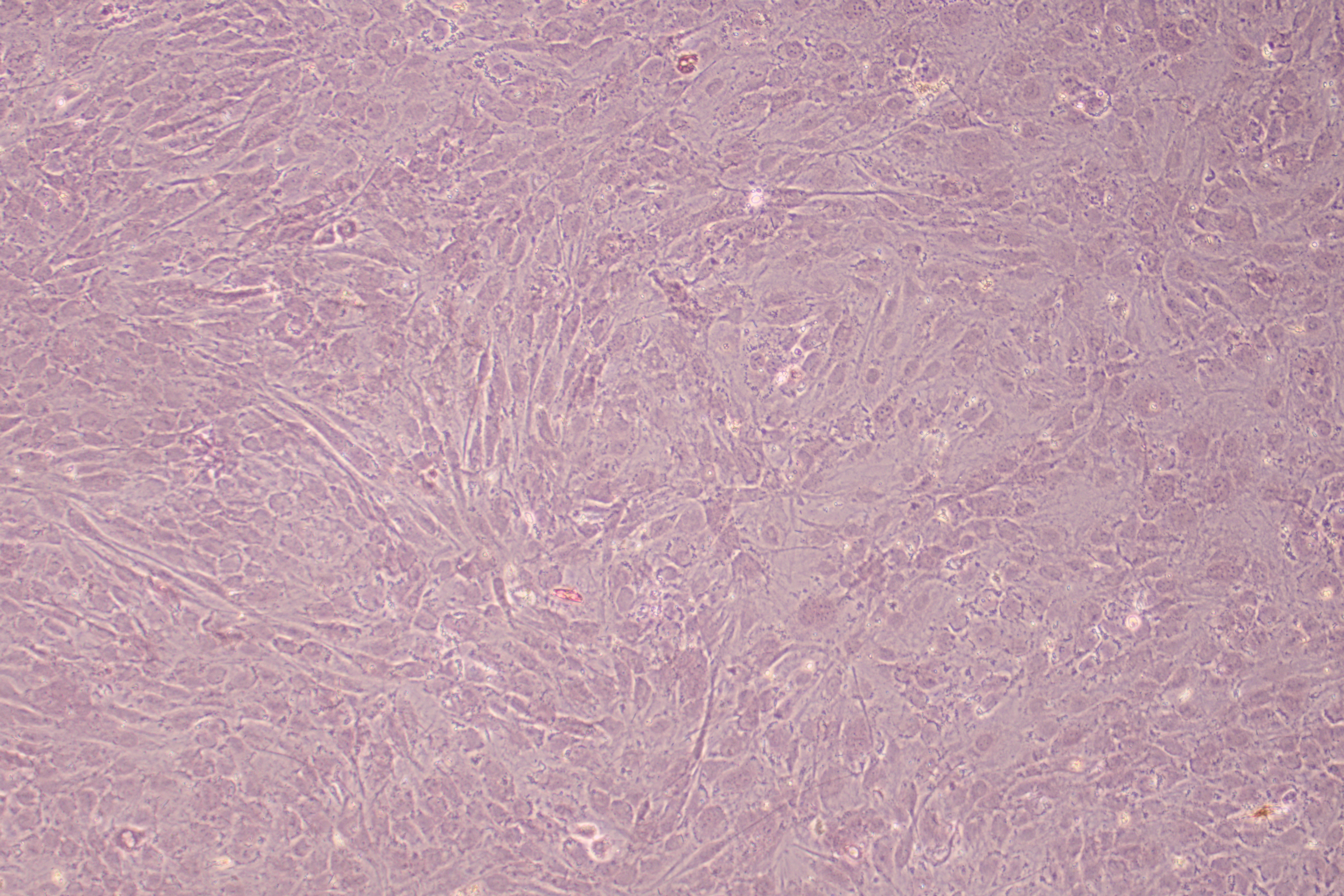

Supplement: Figure 1—source data 2. — The folder named ‘40×’ contains the images at a magnification of 40. The folder named ‘100×’ contains the images at a magnification of 100. [file elife-64872-fig1-data2.zip › Figure 1-source data 2/100×/0.png]

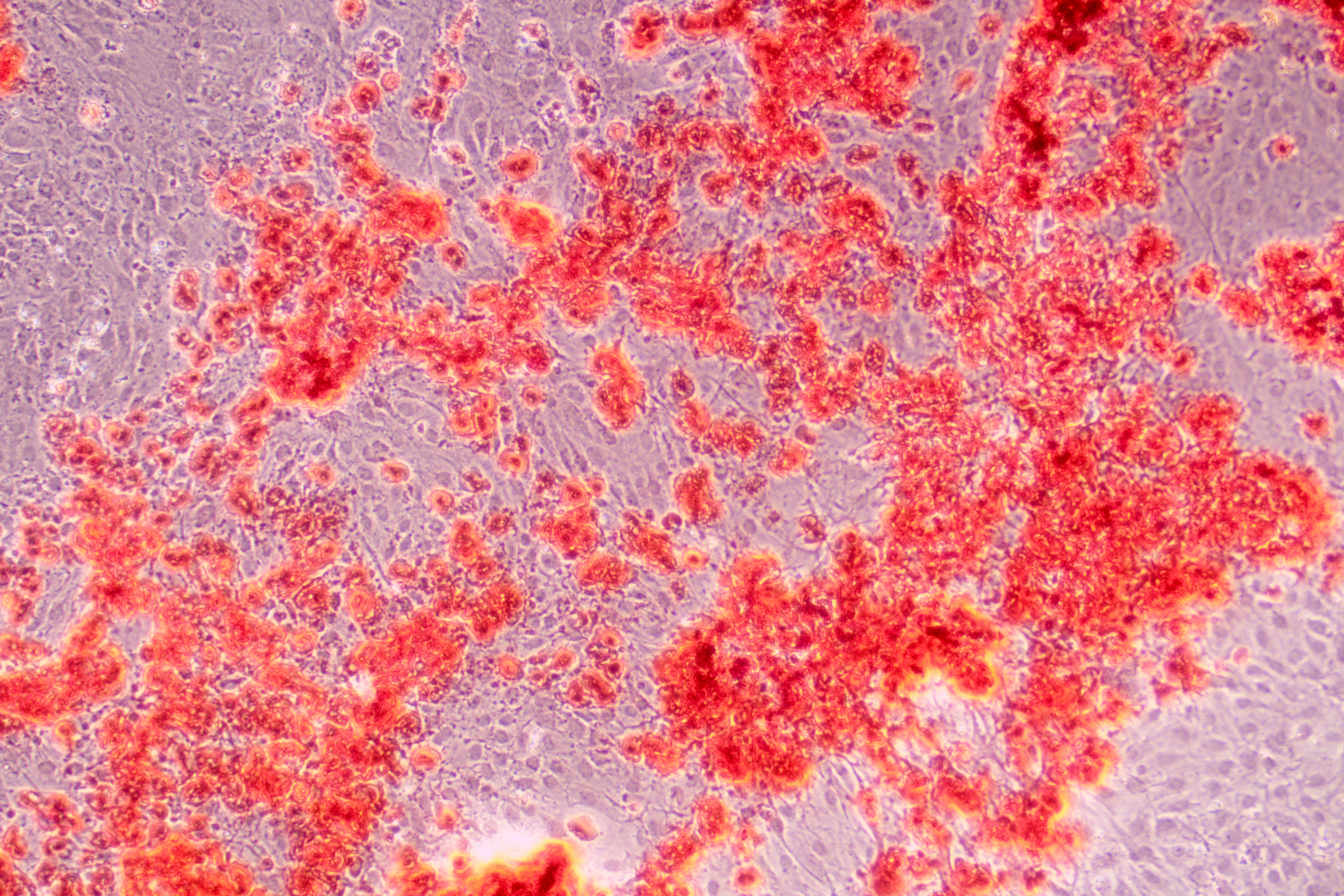

Supplement: Figure 1—source data 2. — The folder named ‘40×’ contains the images at a magnification of 40. The folder named ‘100×’ contains the images at a magnification of 100. [file elife-64872-fig1-data2.zip › Figure 1-source data 2/100×/1μM.png]

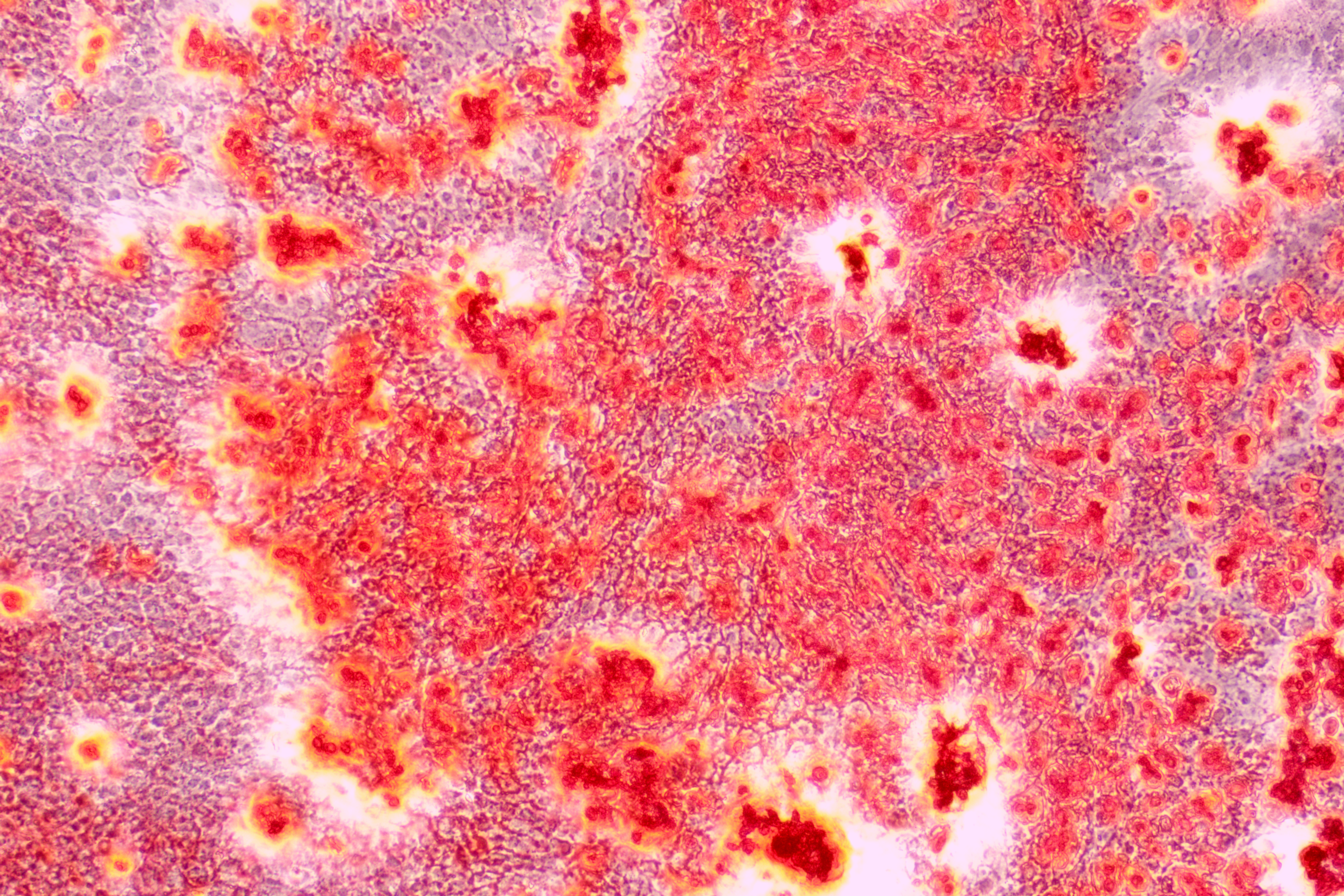

Supplement: Figure 1—source data 2. — The folder named ‘40×’ contains the images at a magnification of 40. The folder named ‘100×’ contains the images at a magnification of 100. [file elife-64872-fig1-data2.zip › Figure 1-source data 2/100×/5μM.png]

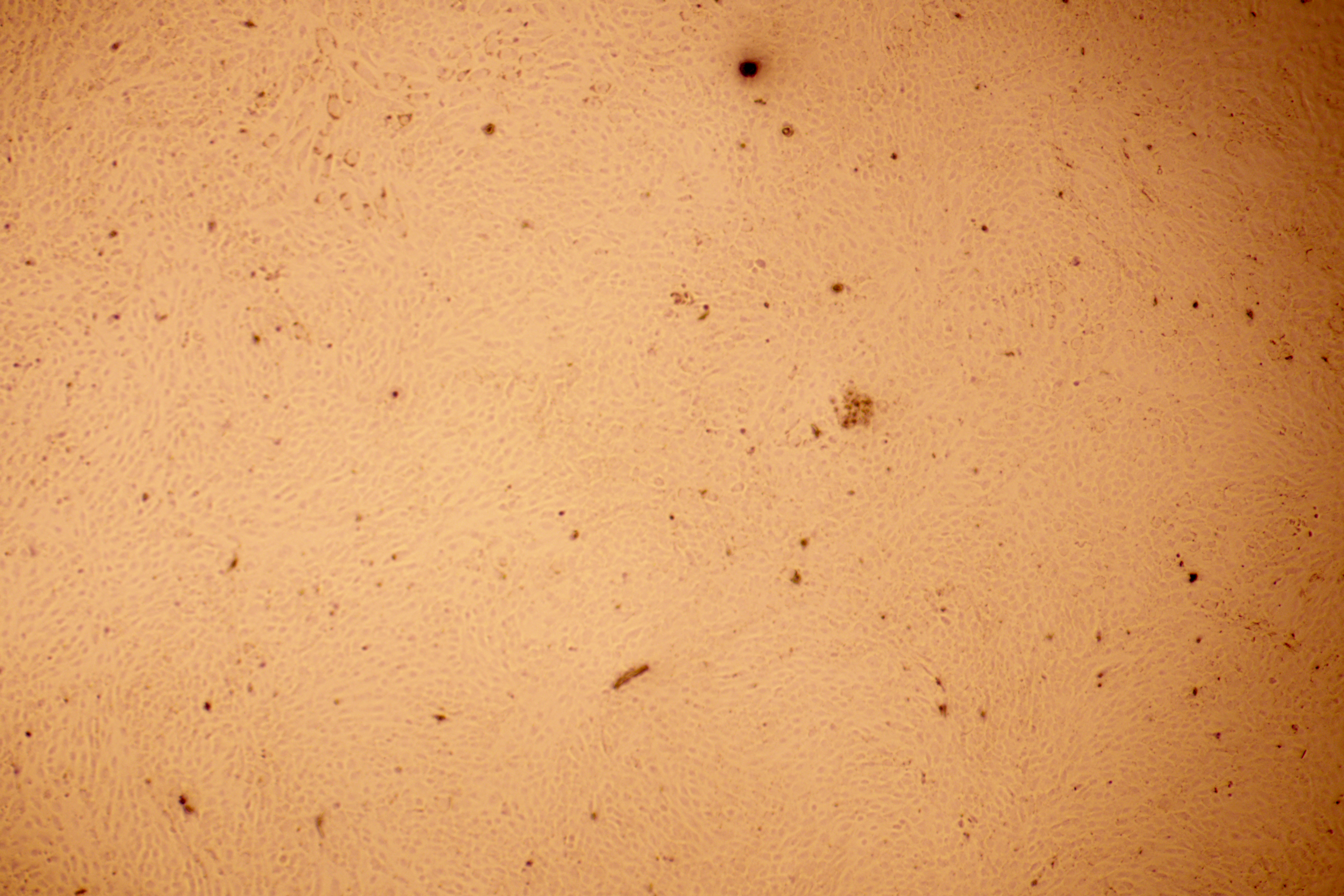

Supplement: Figure 1—source data 2. — The folder named ‘40×’ contains the images at a magnification of 40. The folder named ‘100×’ contains the images at a magnification of 100. [file elife-64872-fig1-data2.zip › Figure 1-source data 2/40×/0.5 μM.png]

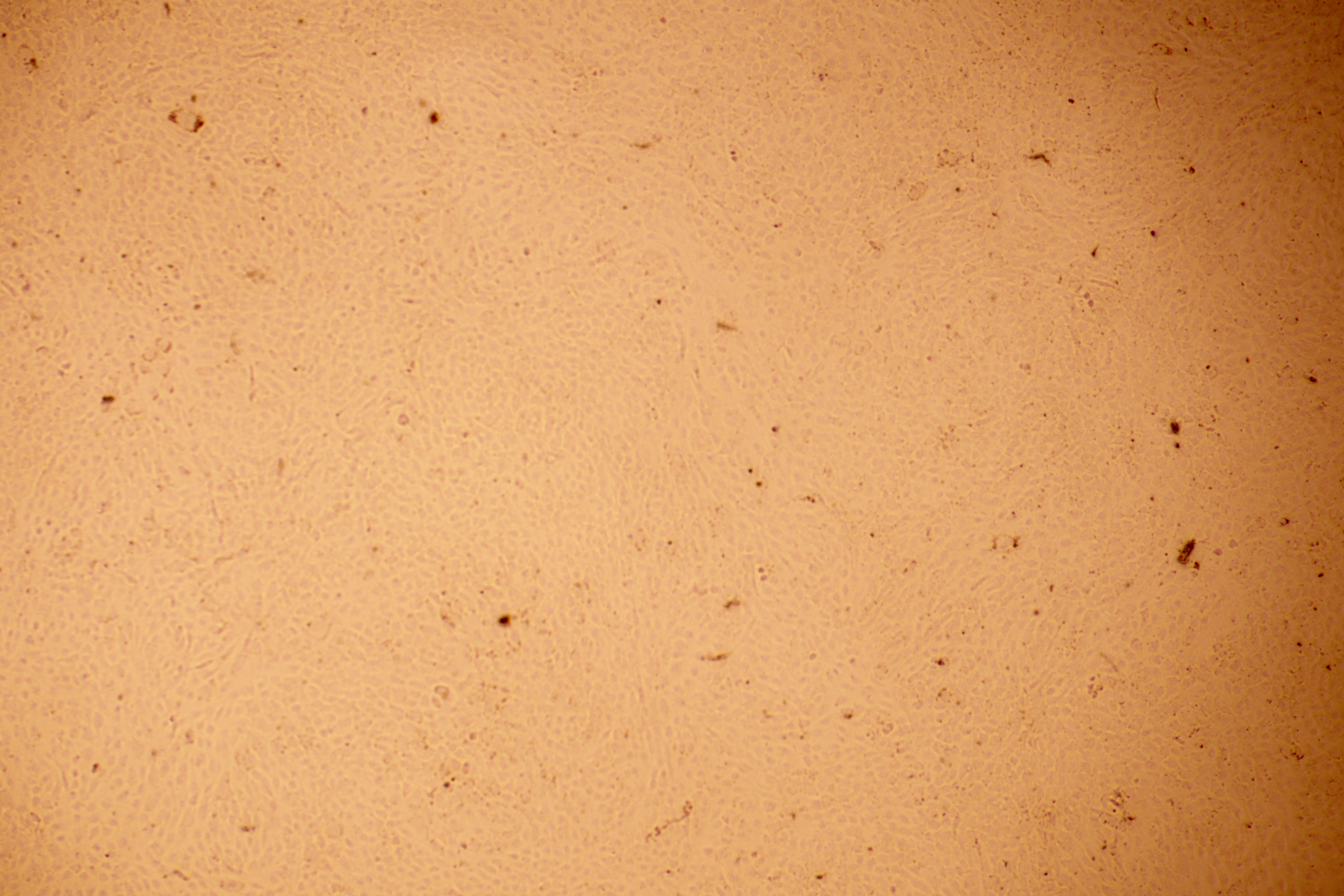

Supplement: Figure 1—source data 2. — The folder named ‘40×’ contains the images at a magnification of 40. The folder named ‘100×’ contains the images at a magnification of 100. [file elife-64872-fig1-data2.zip › Figure 1-source data 2/40×/0.png]

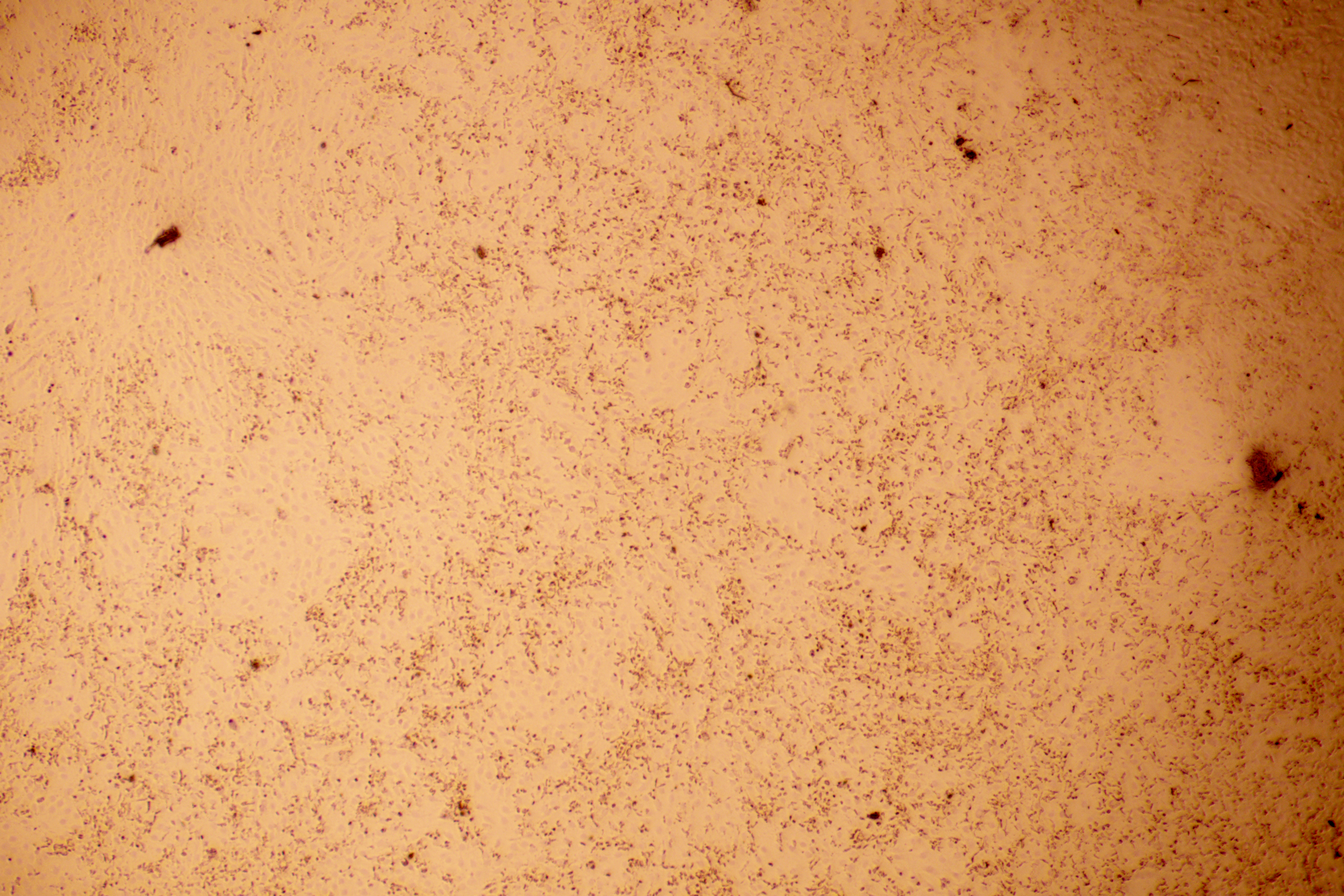

Supplement: Figure 1—source data 2. — The folder named ‘40×’ contains the images at a magnification of 40. The folder named ‘100×’ contains the images at a magnification of 100. [file elife-64872-fig1-data2.zip › Figure 1-source data 2/40×/1 μM.png]

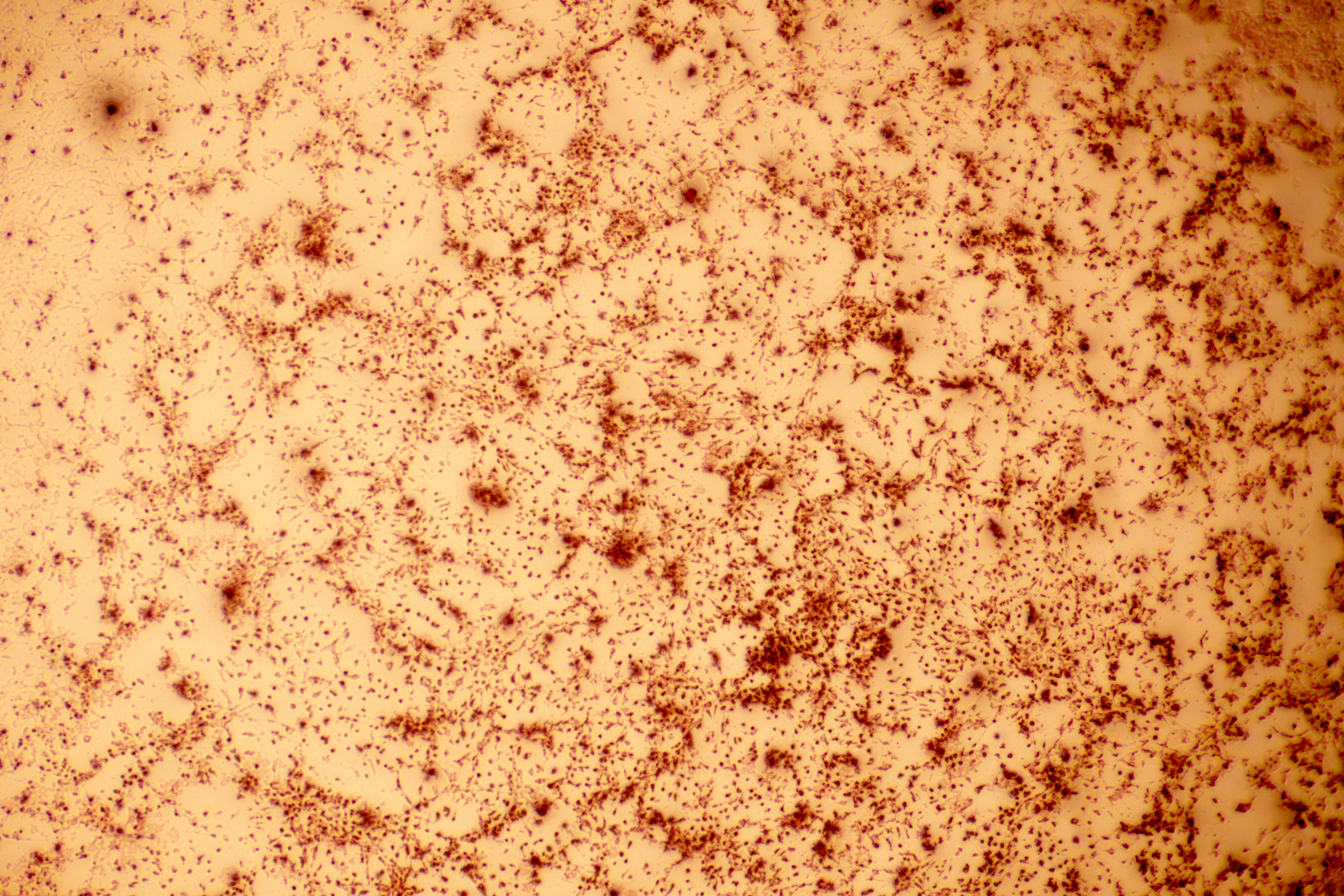

Supplement: Figure 1—source data 2. — The folder named ‘40×’ contains the images at a magnification of 40. The folder named ‘100×’ contains the images at a magnification of 100. [file elife-64872-fig1-data2.zip › Figure 1-source data 2/40×/5 μM.png]

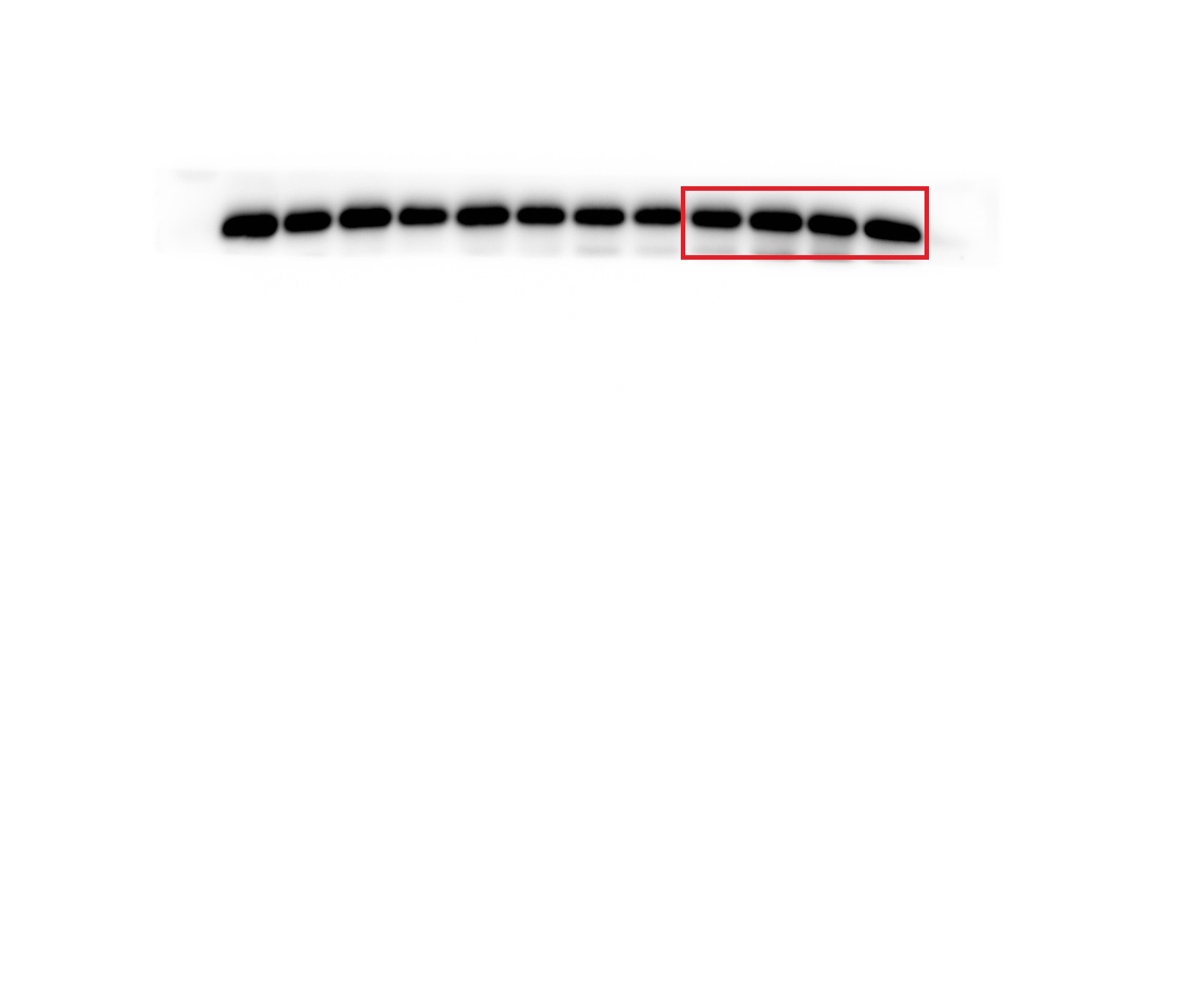

Supplement: Figure 2—source data 1. — The folders named ‘Figure 2A’, ‘Figure 2F ’, and ‘Figure 2J ’ contain the original images in Figure 2A, Figure 2F, and Figure 2J, respectively (the individual file name containing ‘(labeled)’ is blot with the relevant bands labeled by a red outline). [file elife-64872-fig2-data1.zip › Figure 2-source data 1/Figure 2A/GAPDH (labelled).jpg]

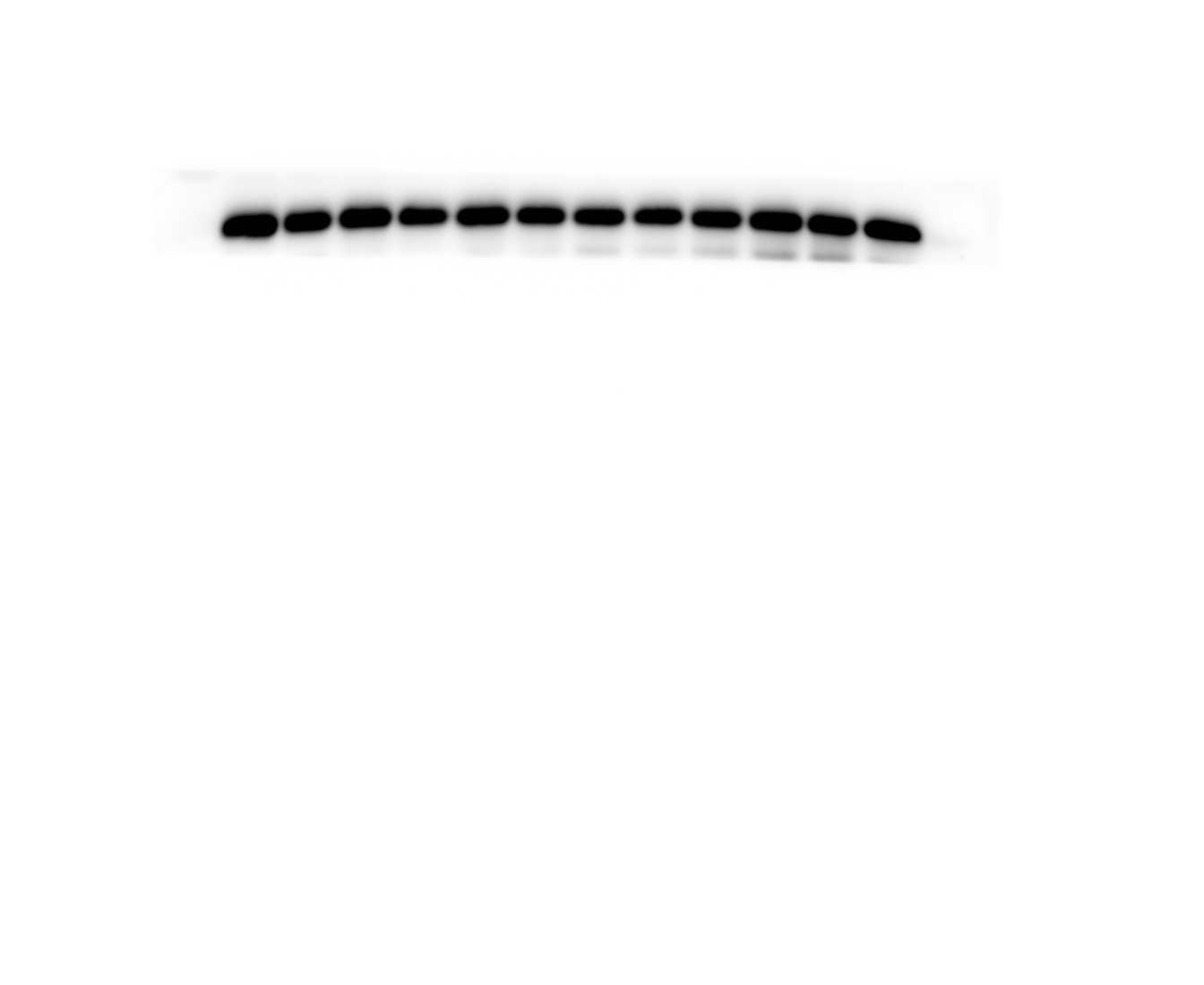

Supplement: Figure 2—source data 1. — The folders named ‘Figure 2A’, ‘Figure 2F ’, and ‘Figure 2J ’ contain the original images in Figure 2A, Figure 2F, and Figure 2J, respectively (the individual file name containing ‘(labeled)’ is blot with the relevant bands labeled by a red outline). [file elife-64872-fig2-data1.zip › Figure 2-source data 1/Figure 2A/GAPDH.jpg]

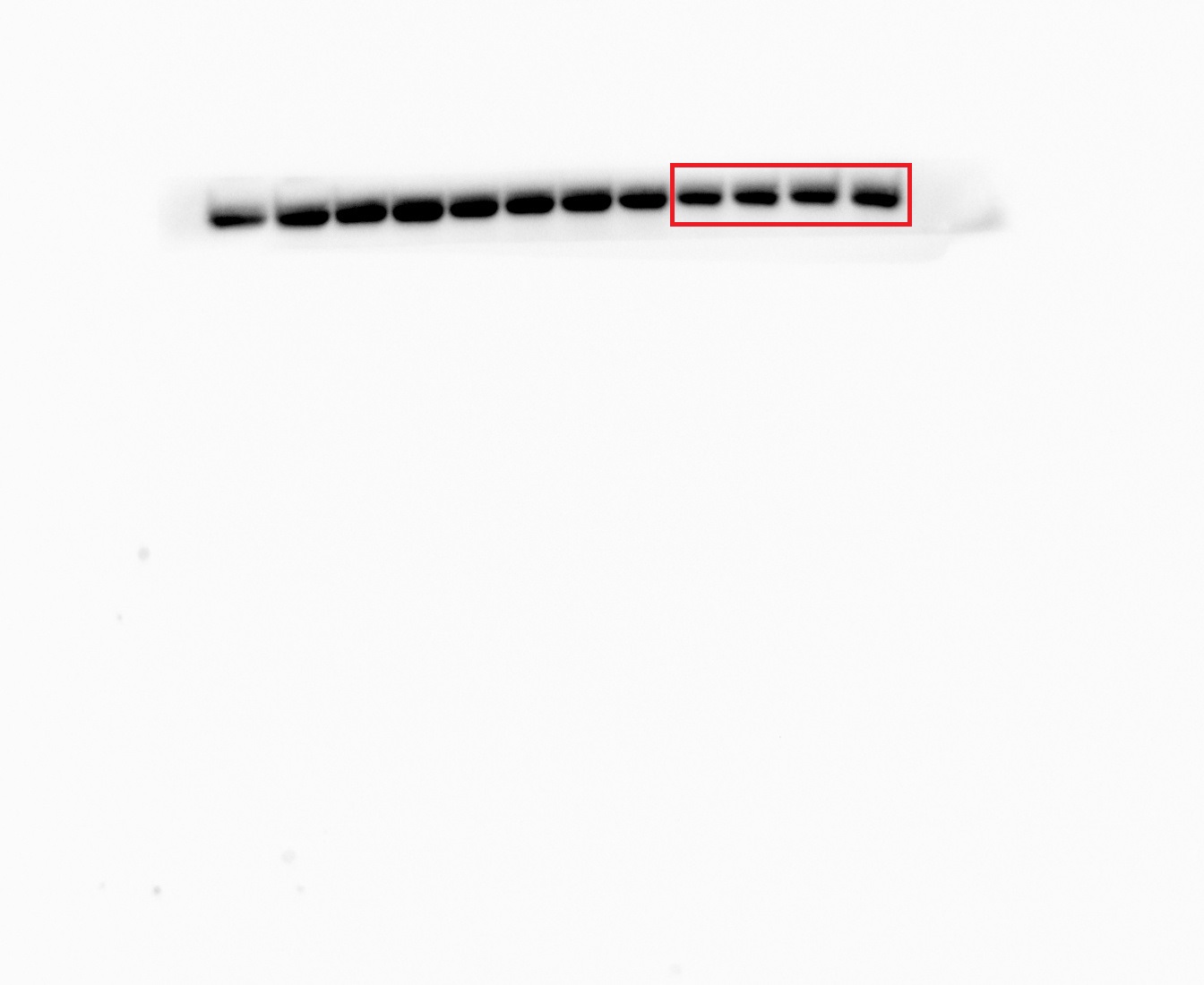

Supplement: Figure 2—source data 1. — The folders named ‘Figure 2A’, ‘Figure 2F ’, and ‘Figure 2J ’ contain the original images in Figure 2A, Figure 2F, and Figure 2J, respectively (the individual file name containing ‘(labeled)’ is blot with the relevant bands labeled by a red outline). [file elife-64872-fig2-data1.zip › Figure 2-source data 1/Figure 2A/GSK3β (labelled).jpg]

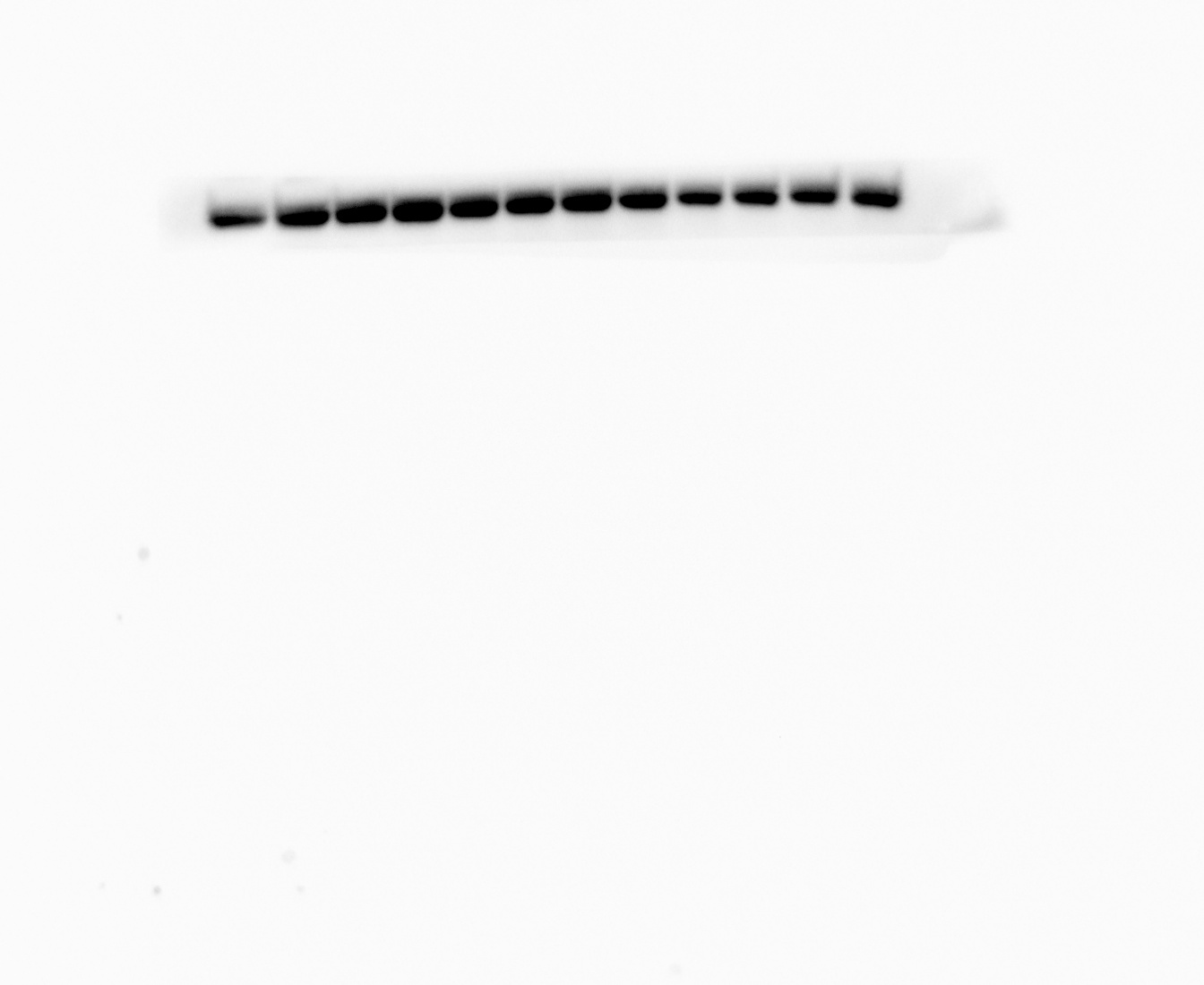

Supplement: Figure 2—source data 1. — The folders named ‘Figure 2A’, ‘Figure 2F ’, and ‘Figure 2J ’ contain the original images in Figure 2A, Figure 2F, and Figure 2J, respectively (the individual file name containing ‘(labeled)’ is blot with the relevant bands labeled by a red outline). [file elife-64872-fig2-data1.zip › Figure 2-source data 1/Figure 2A/GSK3β.jpg]

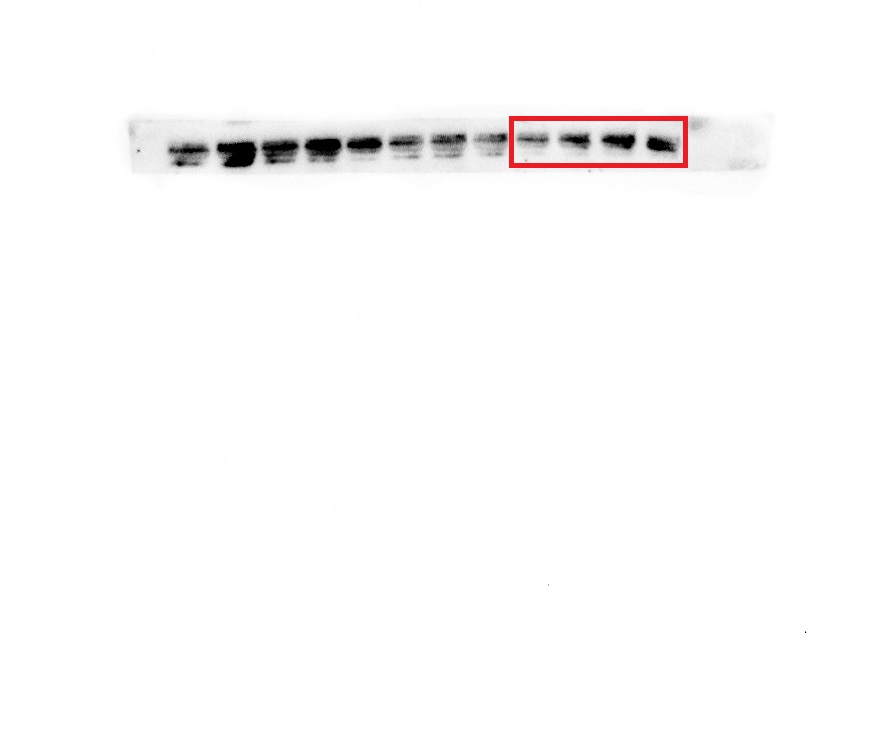

Supplement: Figure 2—source data 1. — The folders named ‘Figure 2A’, ‘Figure 2F ’, and ‘Figure 2J ’ contain the original images in Figure 2A, Figure 2F, and Figure 2J, respectively (the individual file name containing ‘(labeled)’ is blot with the relevant bands labeled by a red outline). [file elife-64872-fig2-data1.zip › Figure 2-source data 1/Figure 2A/p-GSK3β (labelled).jpg]

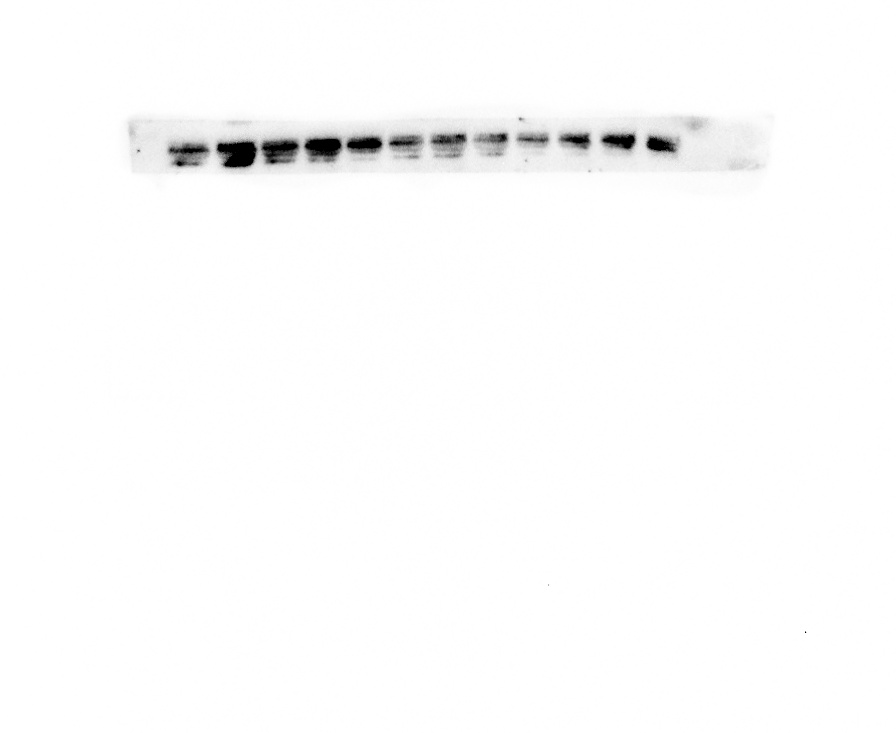

Supplement: Figure 2—source data 1. — The folders named ‘Figure 2A’, ‘Figure 2F ’, and ‘Figure 2J ’ contain the original images in Figure 2A, Figure 2F, and Figure 2J, respectively (the individual file name containing ‘(labeled)’ is blot with the relevant bands labeled by a red outline). [file elife-64872-fig2-data1.zip › Figure 2-source data 1/Figure 2A/p-GSK3β.jpg]

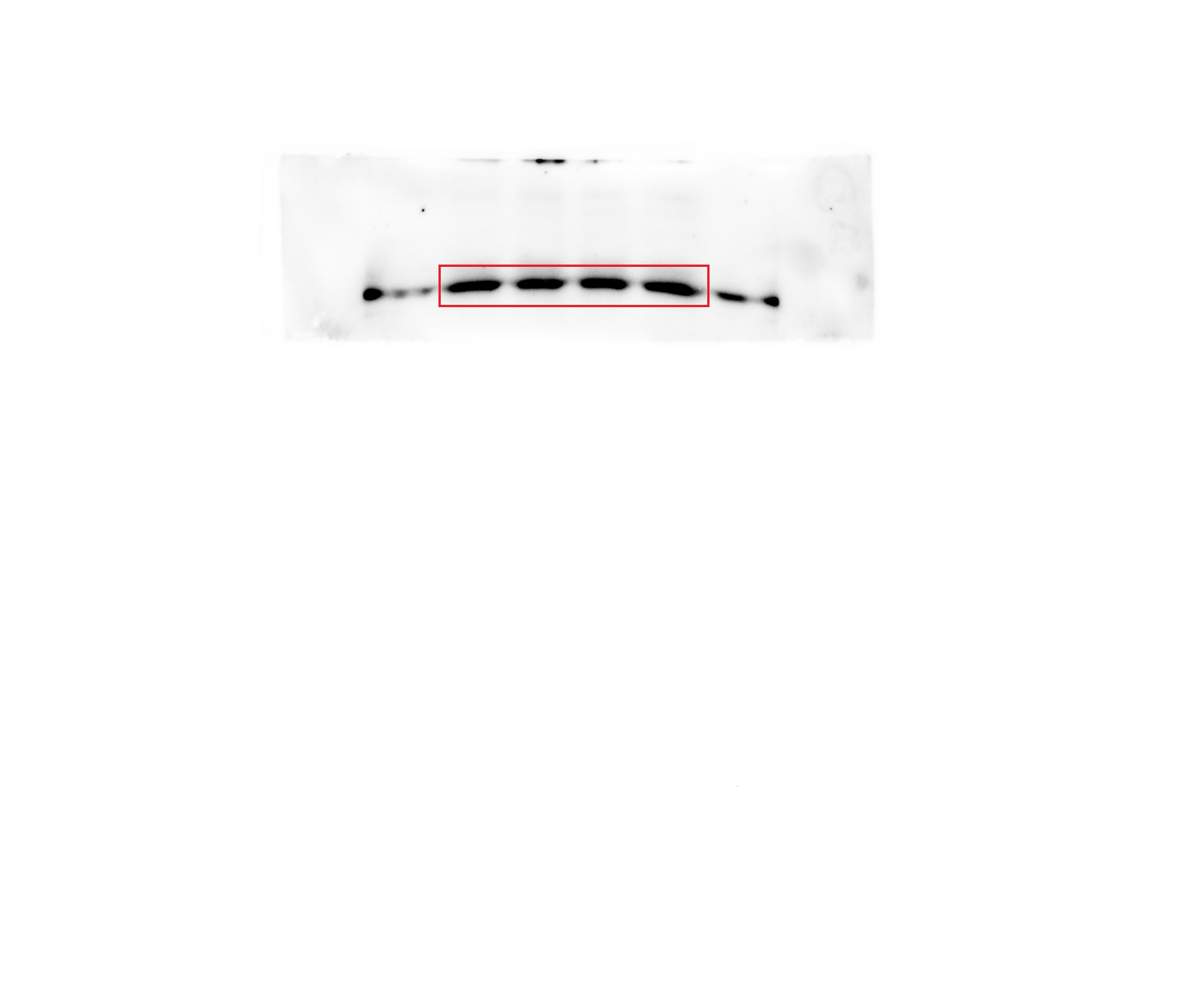

Supplement: Figure 2—source data 1. — The folders named ‘Figure 2A’, ‘Figure 2F ’, and ‘Figure 2J ’ contain the original images in Figure 2A, Figure 2F, and Figure 2J, respectively (the individual file name containing ‘(labeled)’ is blot with the relevant bands labeled by a red outline). [file elife-64872-fig2-data1.zip › Figure 2-source data 1/Figure 2F/GAPDH (labelled).jpg]

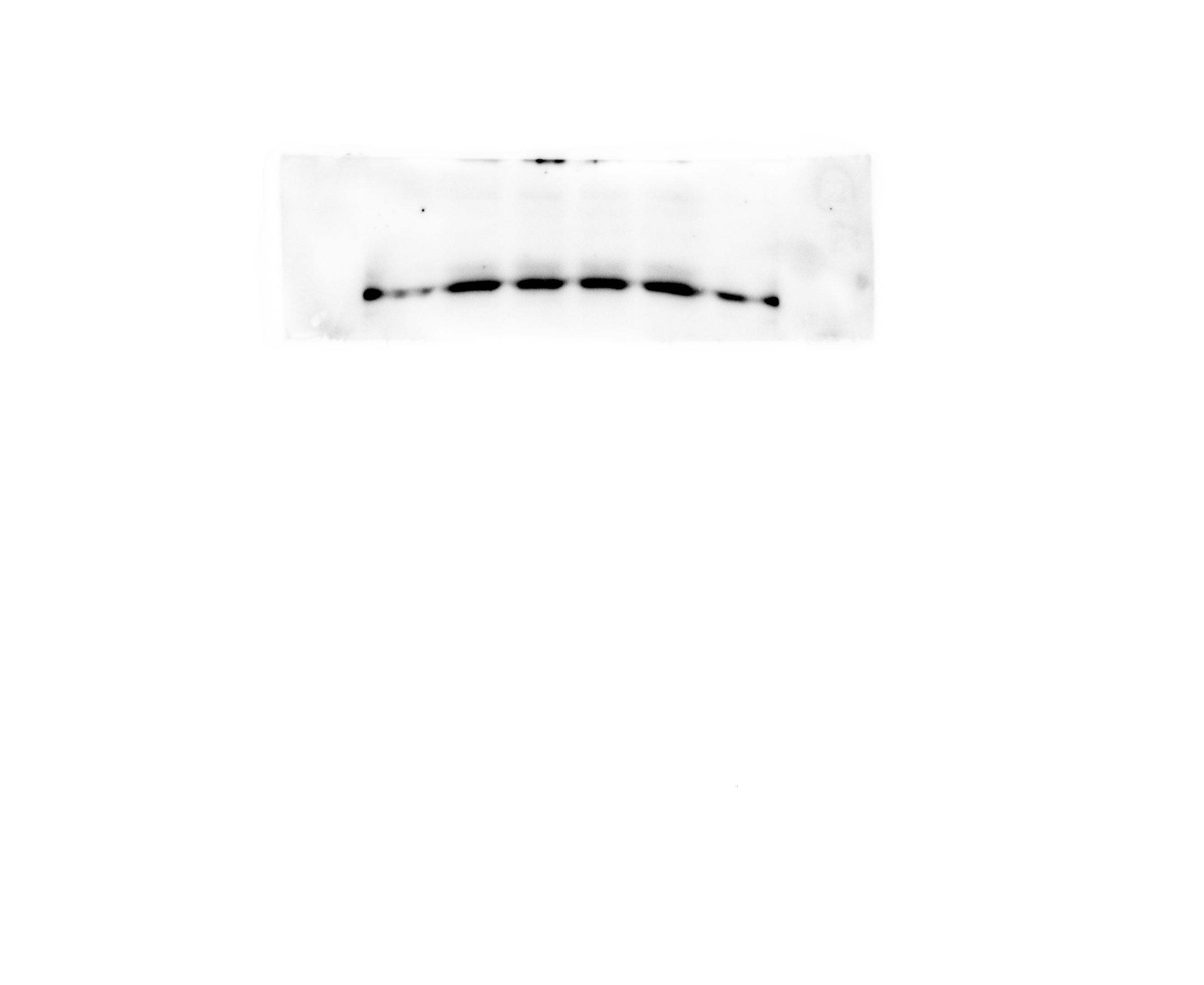

Supplement: Figure 2—source data 1. — The folders named ‘Figure 2A’, ‘Figure 2F ’, and ‘Figure 2J ’ contain the original images in Figure 2A, Figure 2F, and Figure 2J, respectively (the individual file name containing ‘(labeled)’ is blot with the relevant bands labeled by a red outline). [file elife-64872-fig2-data1.zip › Figure 2-source data 1/Figure 2F/GAPDH.jpg]

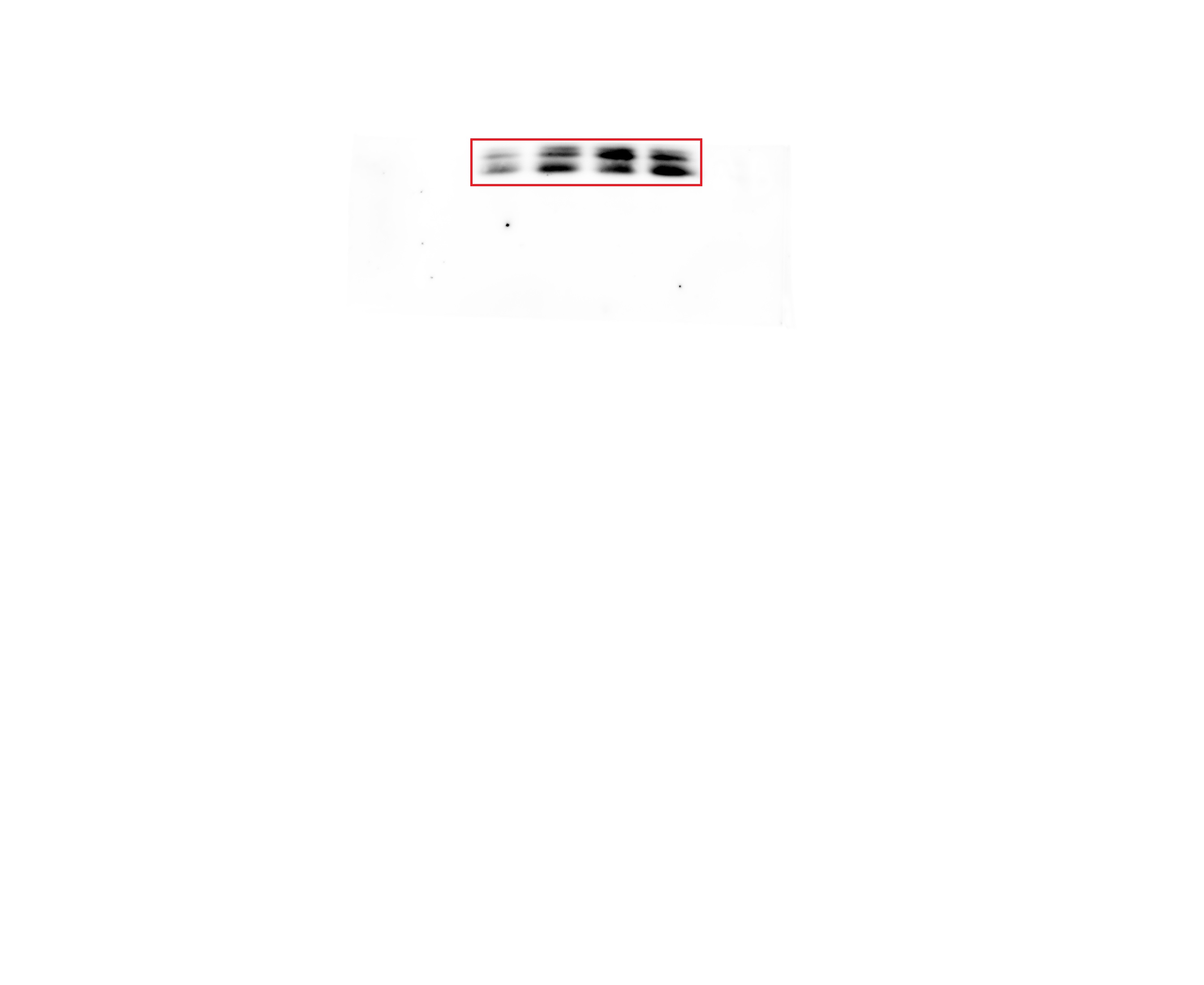

Supplement: Figure 2—source data 1. — The folders named ‘Figure 2A’, ‘Figure 2F ’, and ‘Figure 2J ’ contain the original images in Figure 2A, Figure 2F, and Figure 2J, respectively (the individual file name containing ‘(labeled)’ is blot with the relevant bands labeled by a red outline). [file elife-64872-fig2-data1.zip › Figure 2-source data 1/Figure 2F/Osterix (labelled).jpg]

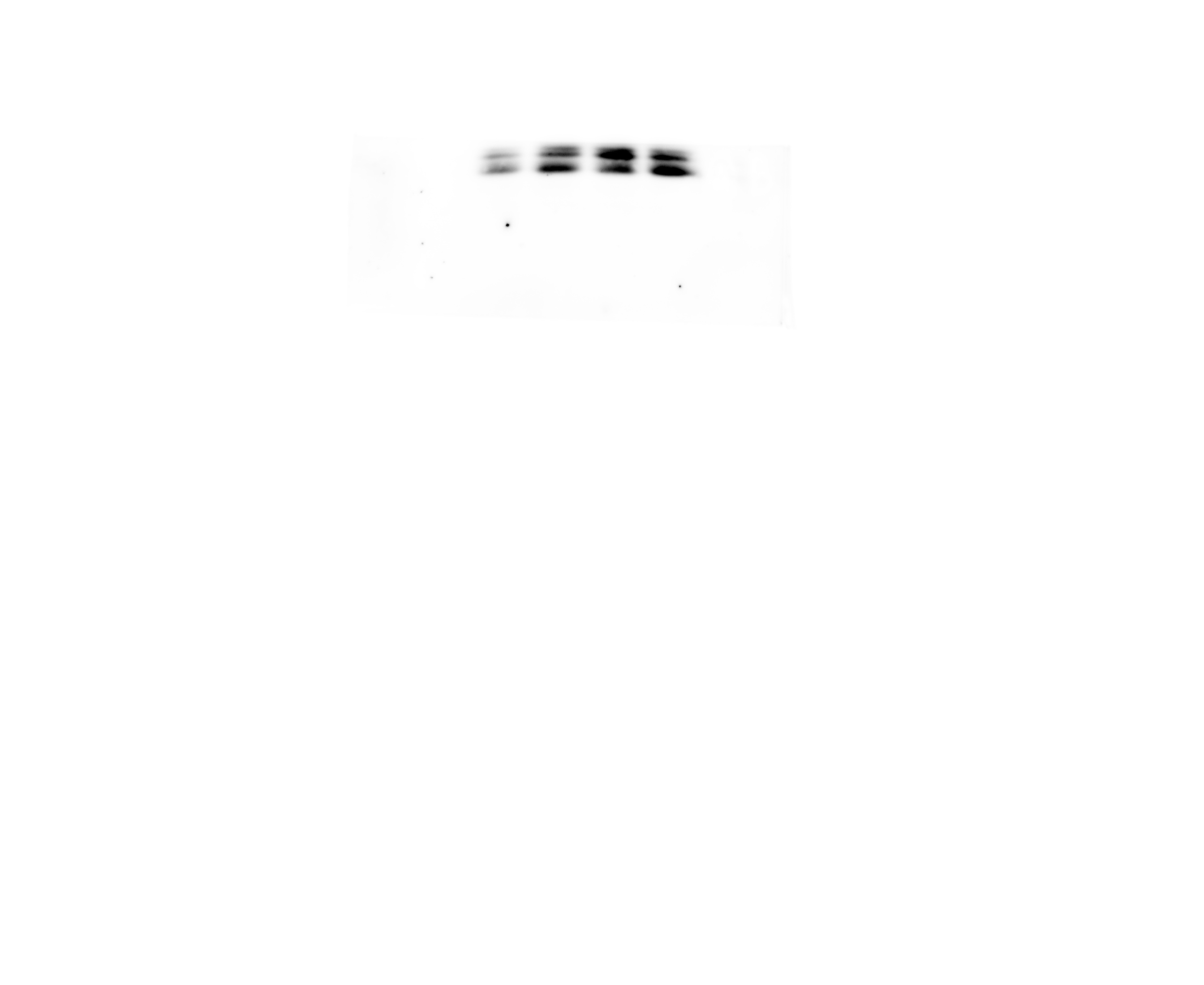

Supplement: Figure 2—source data 1. — The folders named ‘Figure 2A’, ‘Figure 2F ’, and ‘Figure 2J ’ contain the original images in Figure 2A, Figure 2F, and Figure 2J, respectively (the individual file name containing ‘(labeled)’ is blot with the relevant bands labeled by a red outline). [file elife-64872-fig2-data1.zip › Figure 2-source data 1/Figure 2F/Osterix.jpg]

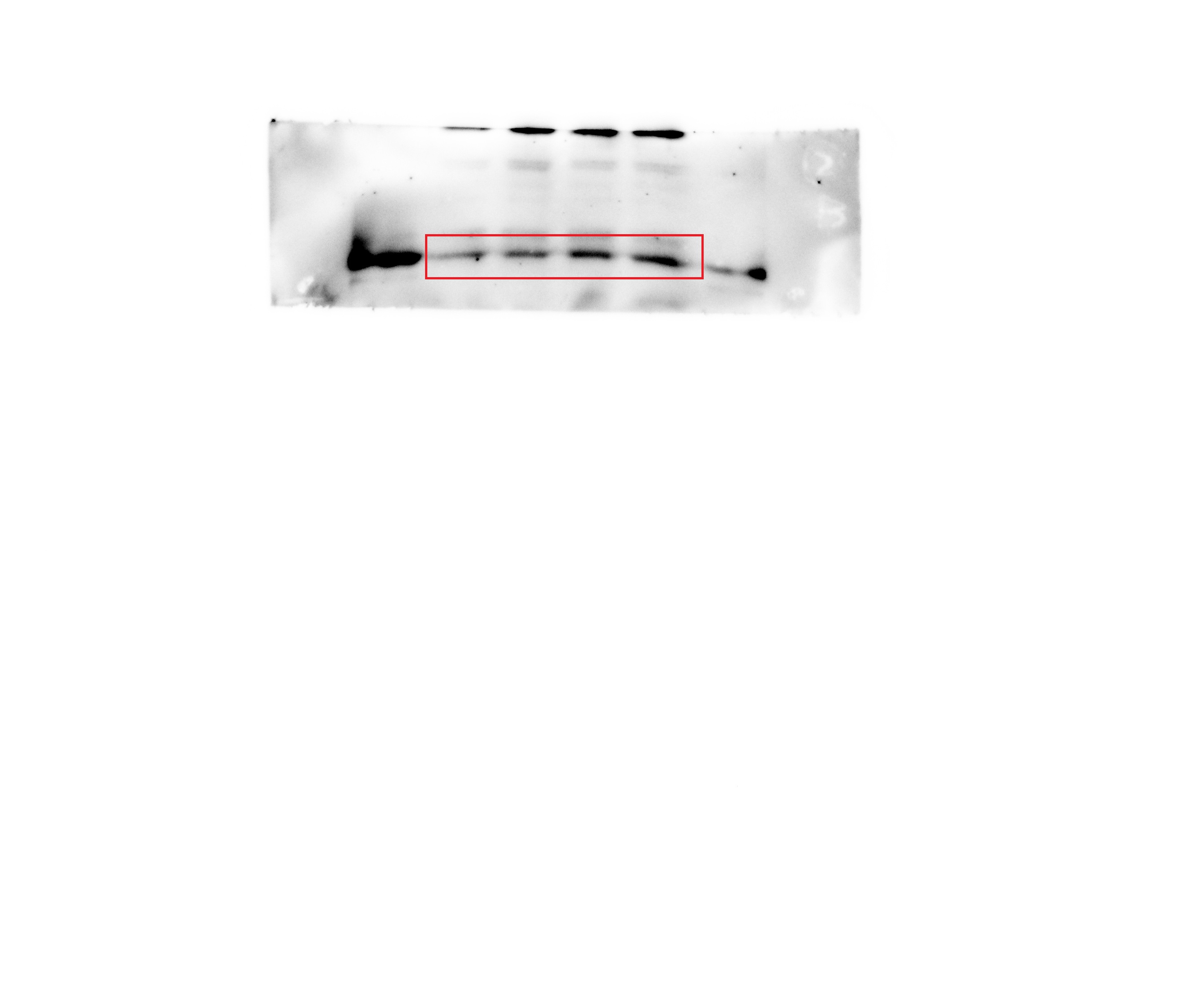

Supplement: Figure 2—source data 1. — The folders named ‘Figure 2A’, ‘Figure 2F ’, and ‘Figure 2J ’ contain the original images in Figure 2A, Figure 2F, and Figure 2J, respectively (the individual file name containing ‘(labeled)’ is blot with the relevant bands labeled by a red outline). [file elife-64872-fig2-data1.zip › Figure 2-source data 1/Figure 2F/Runx2 (labelled).jpg]

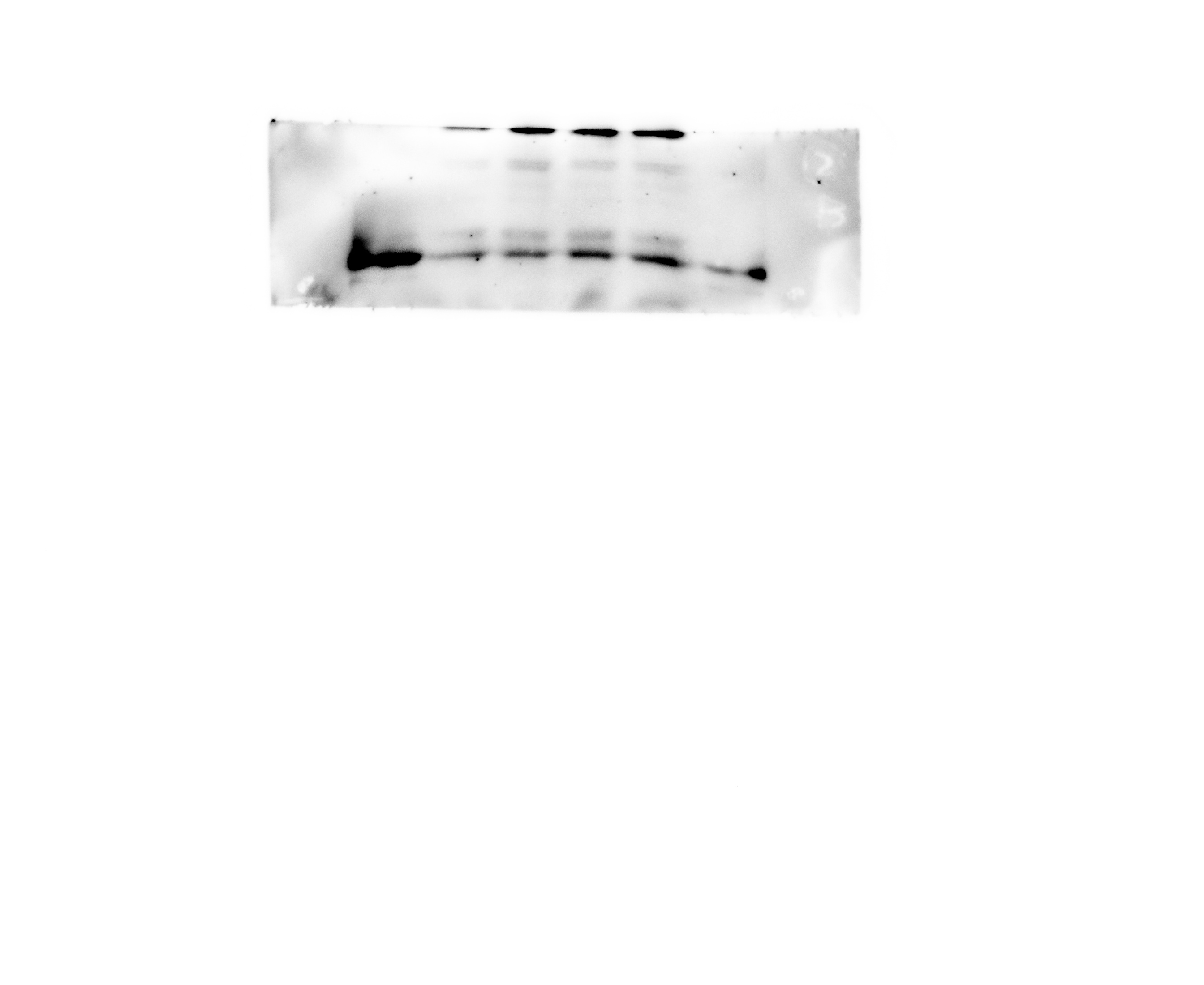

Supplement: Figure 2—source data 1. — The folders named ‘Figure 2A’, ‘Figure 2F ’, and ‘Figure 2J ’ contain the original images in Figure 2A, Figure 2F, and Figure 2J, respectively (the individual file name containing ‘(labeled)’ is blot with the relevant bands labeled by a red outline). [file elife-64872-fig2-data1.zip › Figure 2-source data 1/Figure 2F/Runx2.jpg]

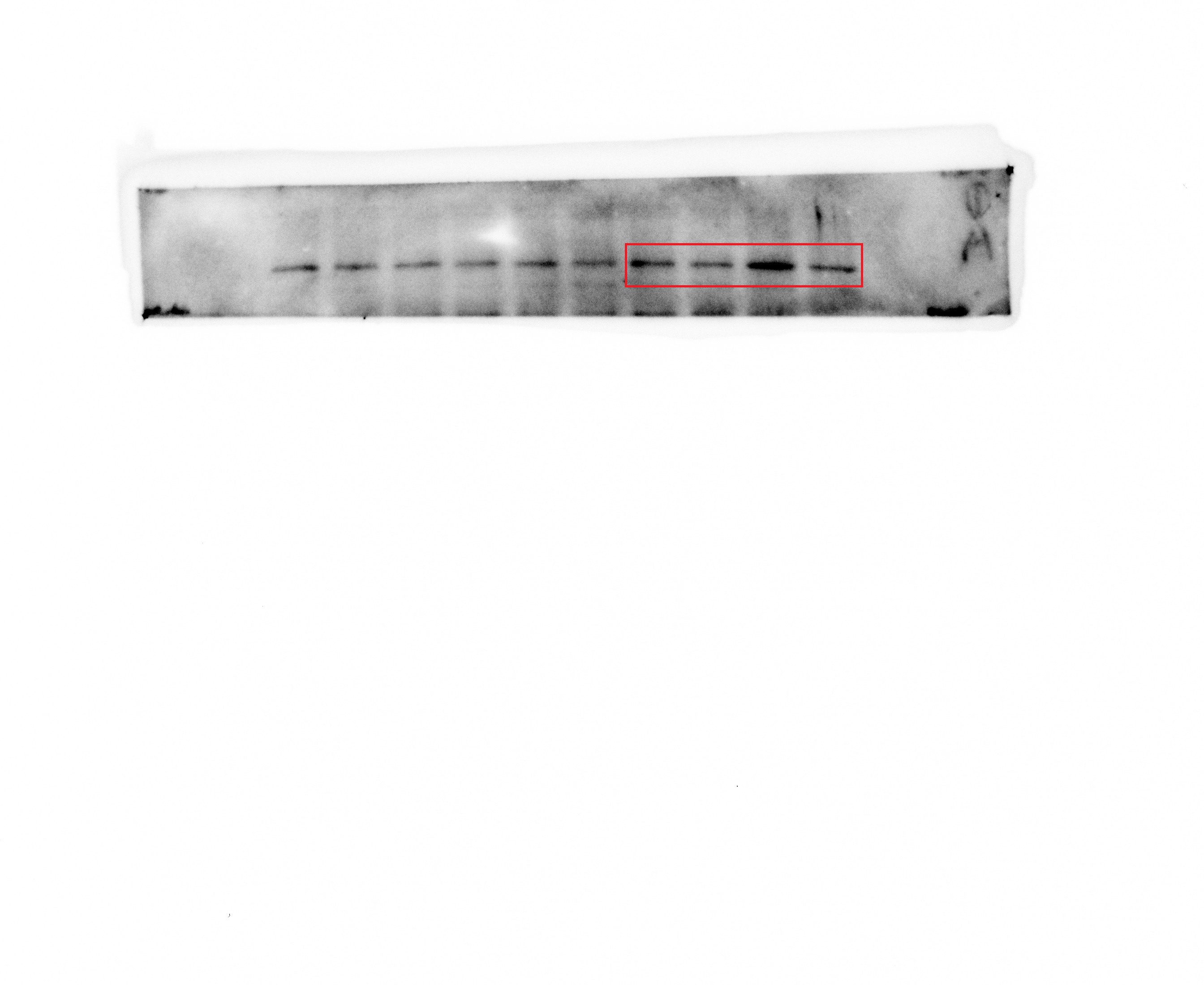

Supplement: Figure 2—source data 1. — The folders named ‘Figure 2A’, ‘Figure 2F ’, and ‘Figure 2J ’ contain the original images in Figure 2A, Figure 2F, and Figure 2J, respectively (the individual file name containing ‘(labeled)’ is blot with the relevant bands labeled by a red outline). [file elife-64872-fig2-data1.zip › Figure 2-source data 1/Figure 2F/β-catenin (labelled).jpg]

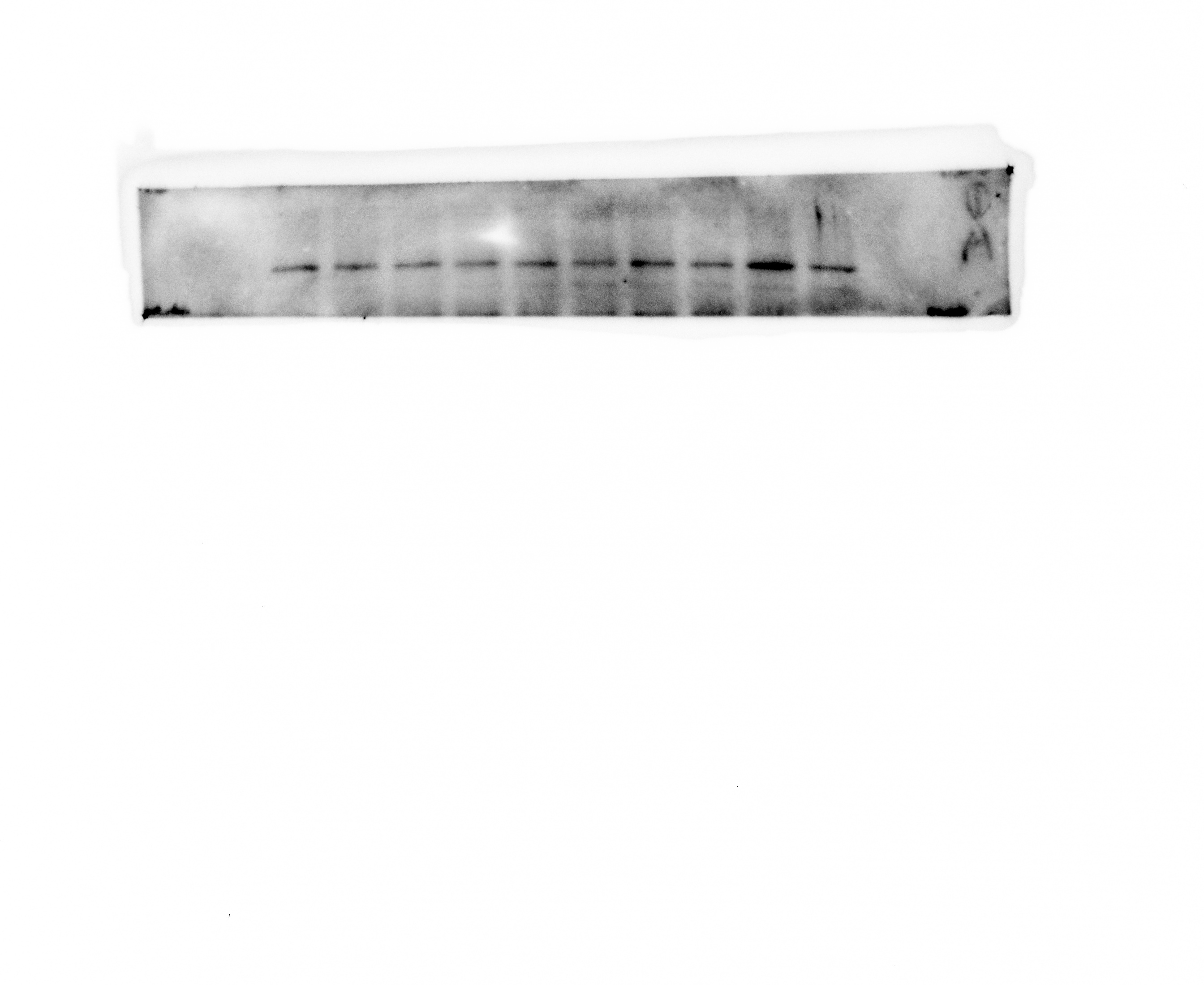

Supplement: Figure 2—source data 1. — The folders named ‘Figure 2A’, ‘Figure 2F ’, and ‘Figure 2J ’ contain the original images in Figure 2A, Figure 2F, and Figure 2J, respectively (the individual file name containing ‘(labeled)’ is blot with the relevant bands labeled by a red outline). [file elife-64872-fig2-data1.zip › Figure 2-source data 1/Figure 2F/β-catenin.jpg]

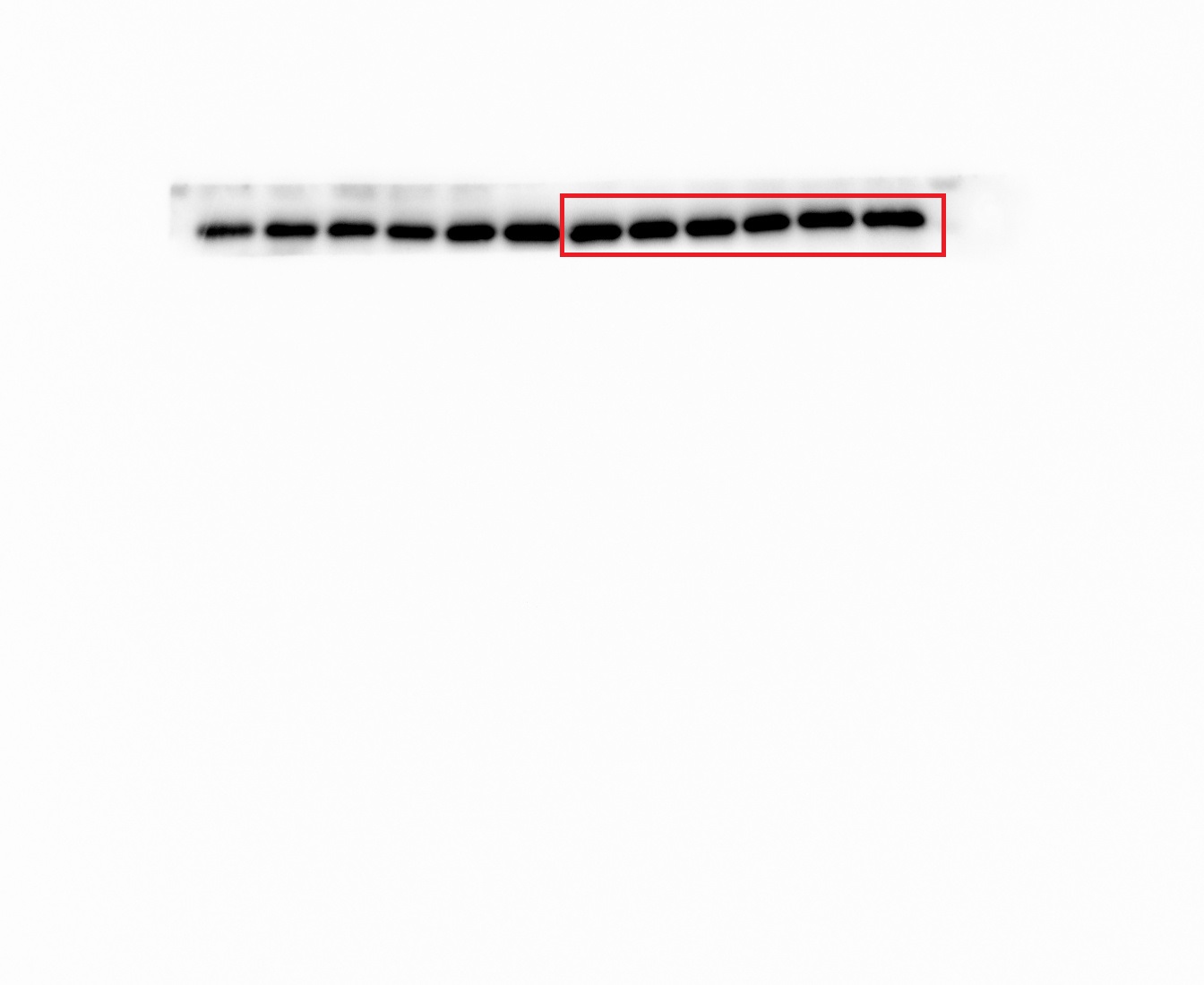

Supplement: Figure 2—source data 1. — The folders named ‘Figure 2A’, ‘Figure 2F ’, and ‘Figure 2J ’ contain the original images in Figure 2A, Figure 2F, and Figure 2J, respectively (the individual file name containing ‘(labeled)’ is blot with the relevant bands labeled by a red outline). [file elife-64872-fig2-data1.zip › Figure 2-source data 1/Figure 2J/GAPDH (labelled).jpg]

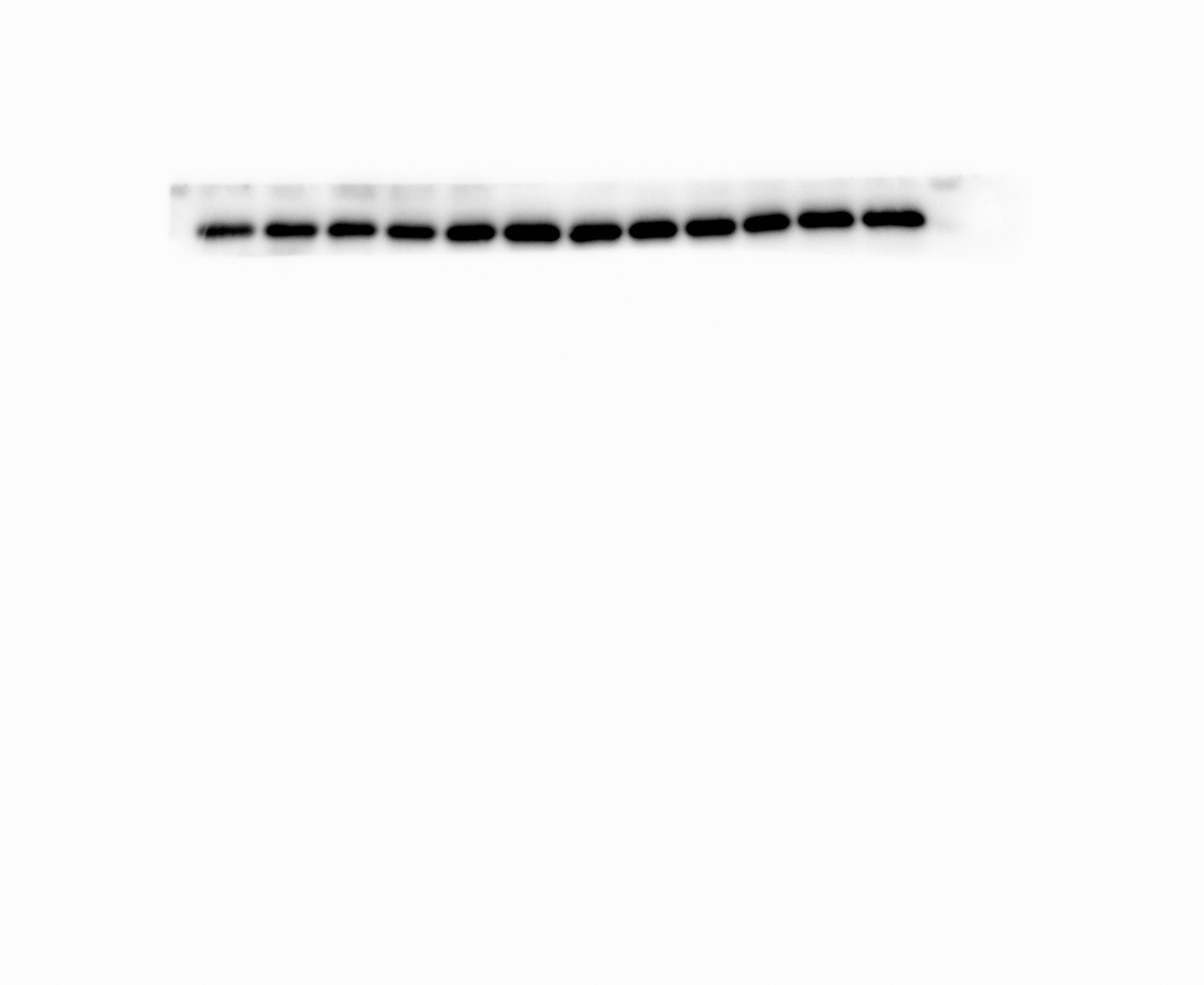

Supplement: Figure 2—source data 1. — The folders named ‘Figure 2A’, ‘Figure 2F ’, and ‘Figure 2J ’ contain the original images in Figure 2A, Figure 2F, and Figure 2J, respectively (the individual file name containing ‘(labeled)’ is blot with the relevant bands labeled by a red outline). [file elife-64872-fig2-data1.zip › Figure 2-source data 1/Figure 2J/GAPDH.jpg]

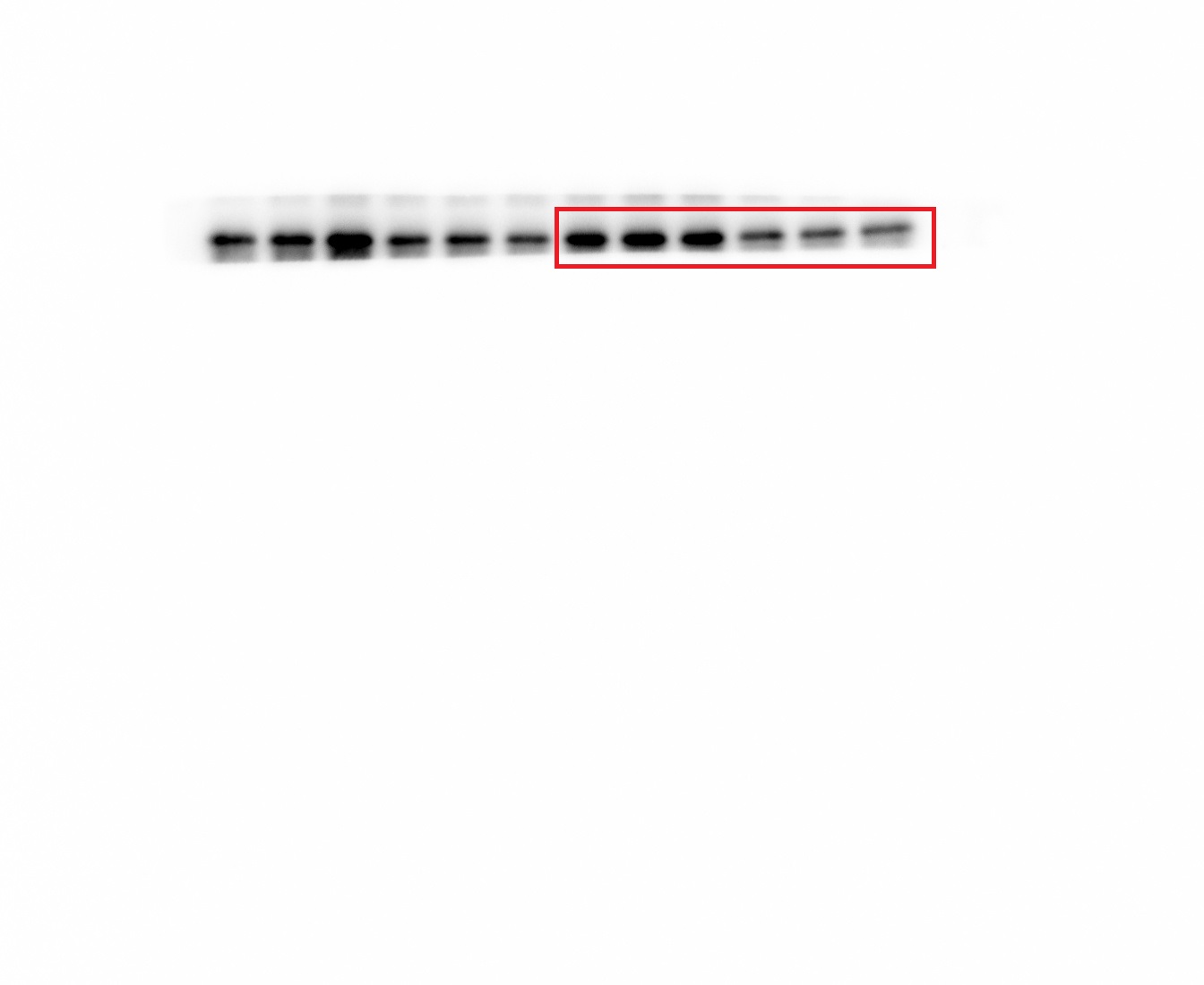

Supplement: Figure 2—source data 1. — The folders named ‘Figure 2A’, ‘Figure 2F ’, and ‘Figure 2J ’ contain the original images in Figure 2A, Figure 2F, and Figure 2J, respectively (the individual file name containing ‘(labeled)’ is blot with the relevant bands labeled by a red outline). [file elife-64872-fig2-data1.zip › Figure 2-source data 1/Figure 2J/Osterix (labelled).jpg]

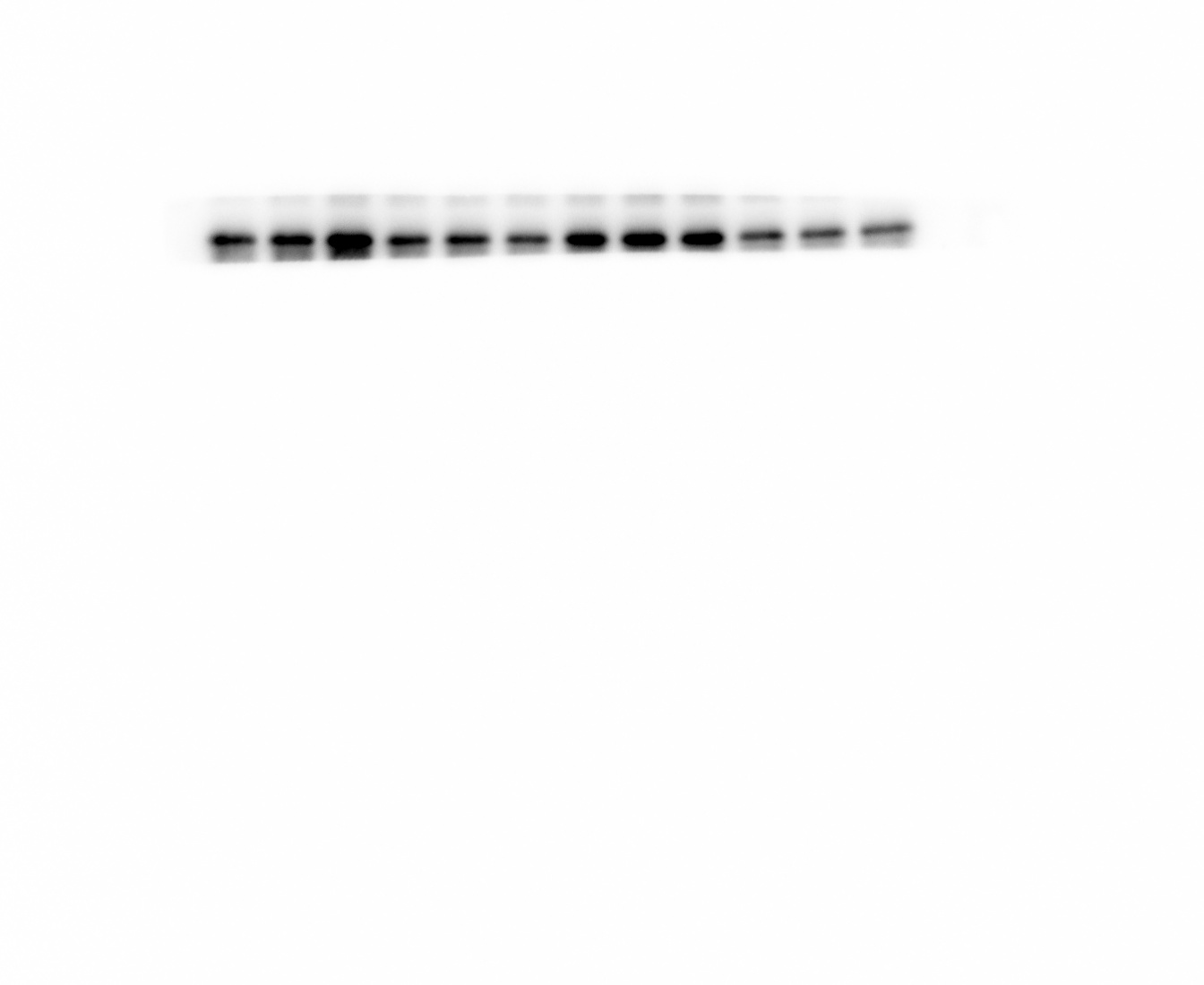

Supplement: Figure 2—source data 1. — The folders named ‘Figure 2A’, ‘Figure 2F ’, and ‘Figure 2J ’ contain the original images in Figure 2A, Figure 2F, and Figure 2J, respectively (the individual file name containing ‘(labeled)’ is blot with the relevant bands labeled by a red outline). [file elife-64872-fig2-data1.zip › Figure 2-source data 1/Figure 2J/Osterix.jpg]

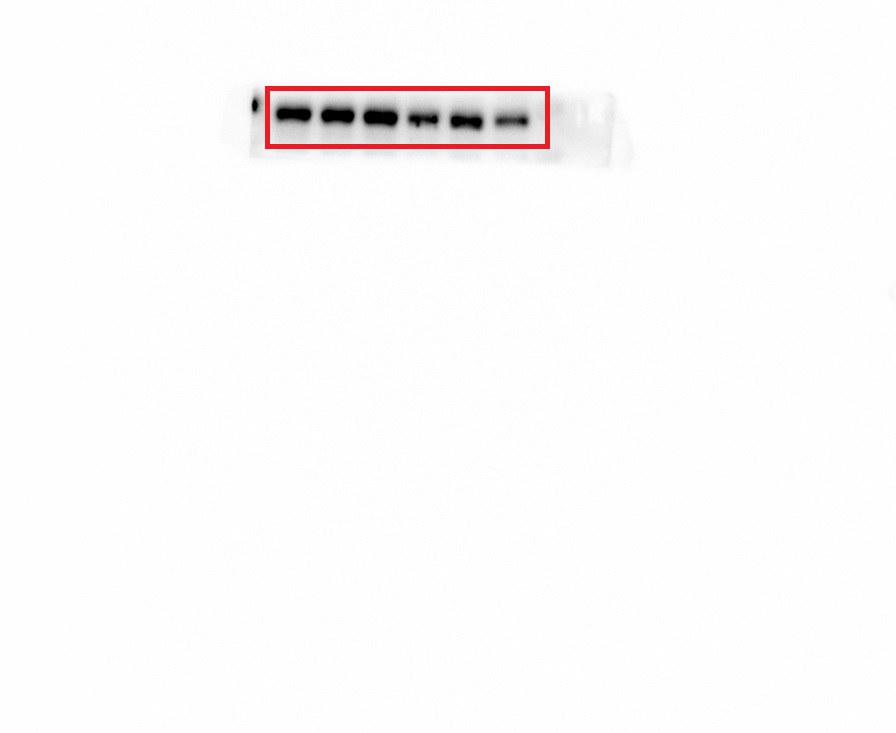

Supplement: Figure 2—source data 1. — The folders named ‘Figure 2A’, ‘Figure 2F ’, and ‘Figure 2J ’ contain the original images in Figure 2A, Figure 2F, and Figure 2J, respectively (the individual file name containing ‘(labeled)’ is blot with the relevant bands labeled by a red outline). [file elife-64872-fig2-data1.zip › Figure 2-source data 1/Figure 2J/RUNX2 (labelled).jpg]

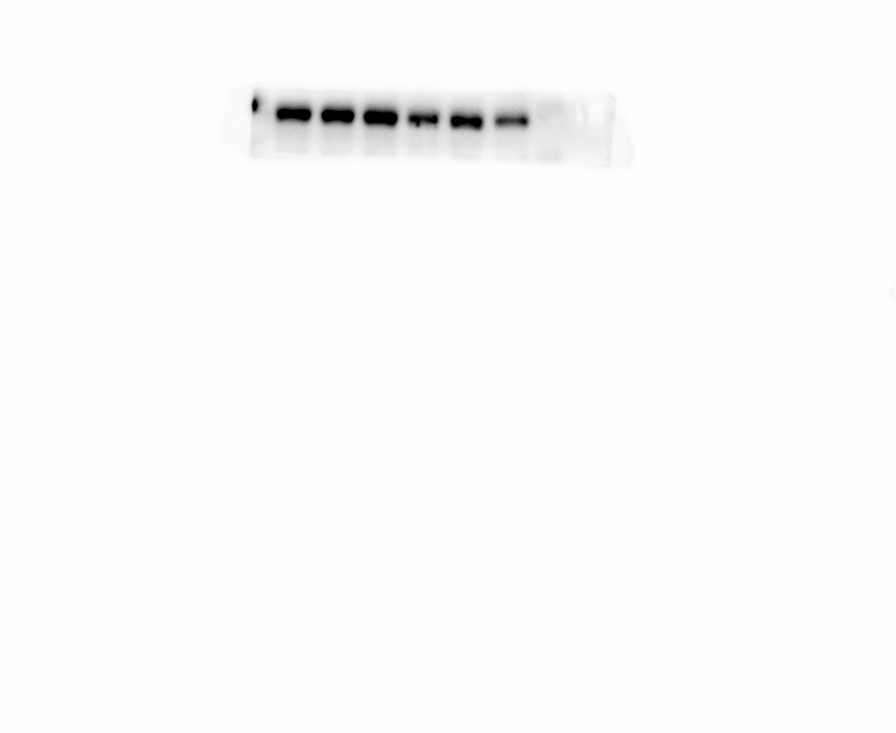

Supplement: Figure 2—source data 1. — The folders named ‘Figure 2A’, ‘Figure 2F ’, and ‘Figure 2J ’ contain the original images in Figure 2A, Figure 2F, and Figure 2J, respectively (the individual file name containing ‘(labeled)’ is blot with the relevant bands labeled by a red outline). [file elife-64872-fig2-data1.zip › Figure 2-source data 1/Figure 2J/RUNX2.jpg]

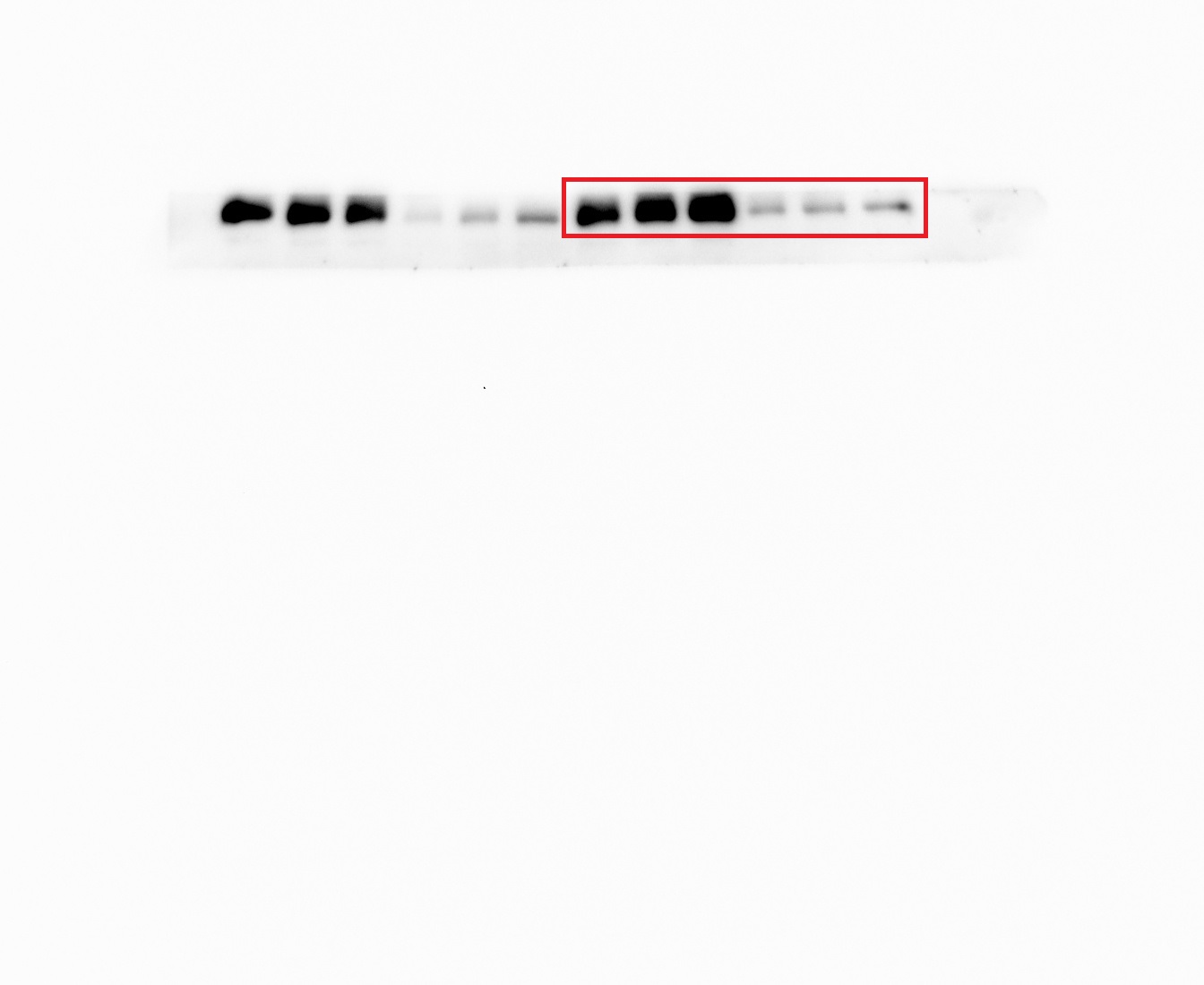

Supplement: Figure 2—source data 1. — The folders named ‘Figure 2A’, ‘Figure 2F ’, and ‘Figure 2J ’ contain the original images in Figure 2A, Figure 2F, and Figure 2J, respectively (the individual file name containing ‘(labeled)’ is blot with the relevant bands labeled by a red outline). [file elife-64872-fig2-data1.zip › Figure 2-source data 1/Figure 2J/β-catenin (labelled).jpg]

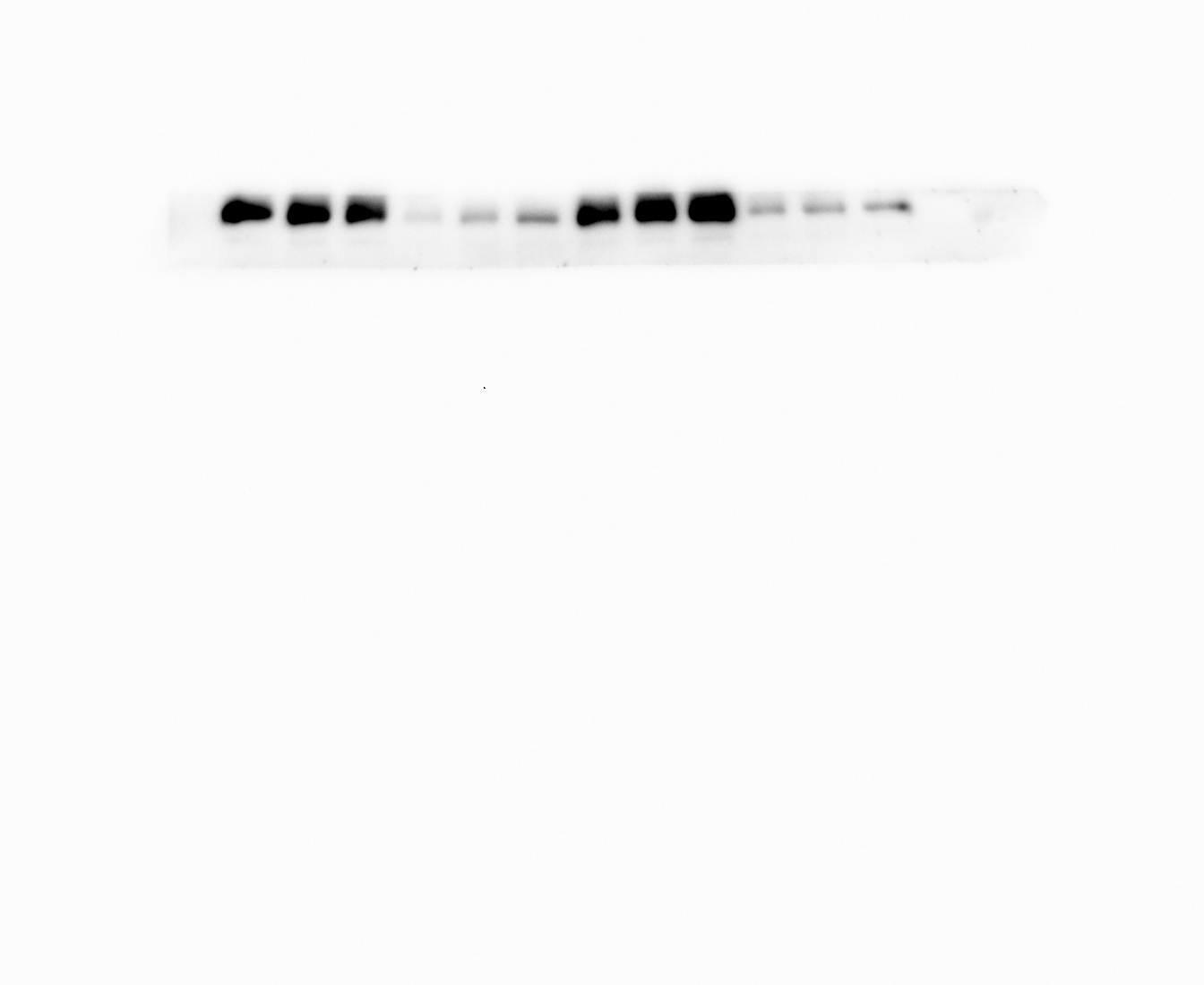

Supplement: Figure 2—source data 1. — The folders named ‘Figure 2A’, ‘Figure 2F ’, and ‘Figure 2J ’ contain the original images in Figure 2A, Figure 2F, and Figure 2J, respectively (the individual file name containing ‘(labeled)’ is blot with the relevant bands labeled by a red outline). [file elife-64872-fig2-data1.zip › Figure 2-source data 1/Figure 2J/β-catenin.jpg]

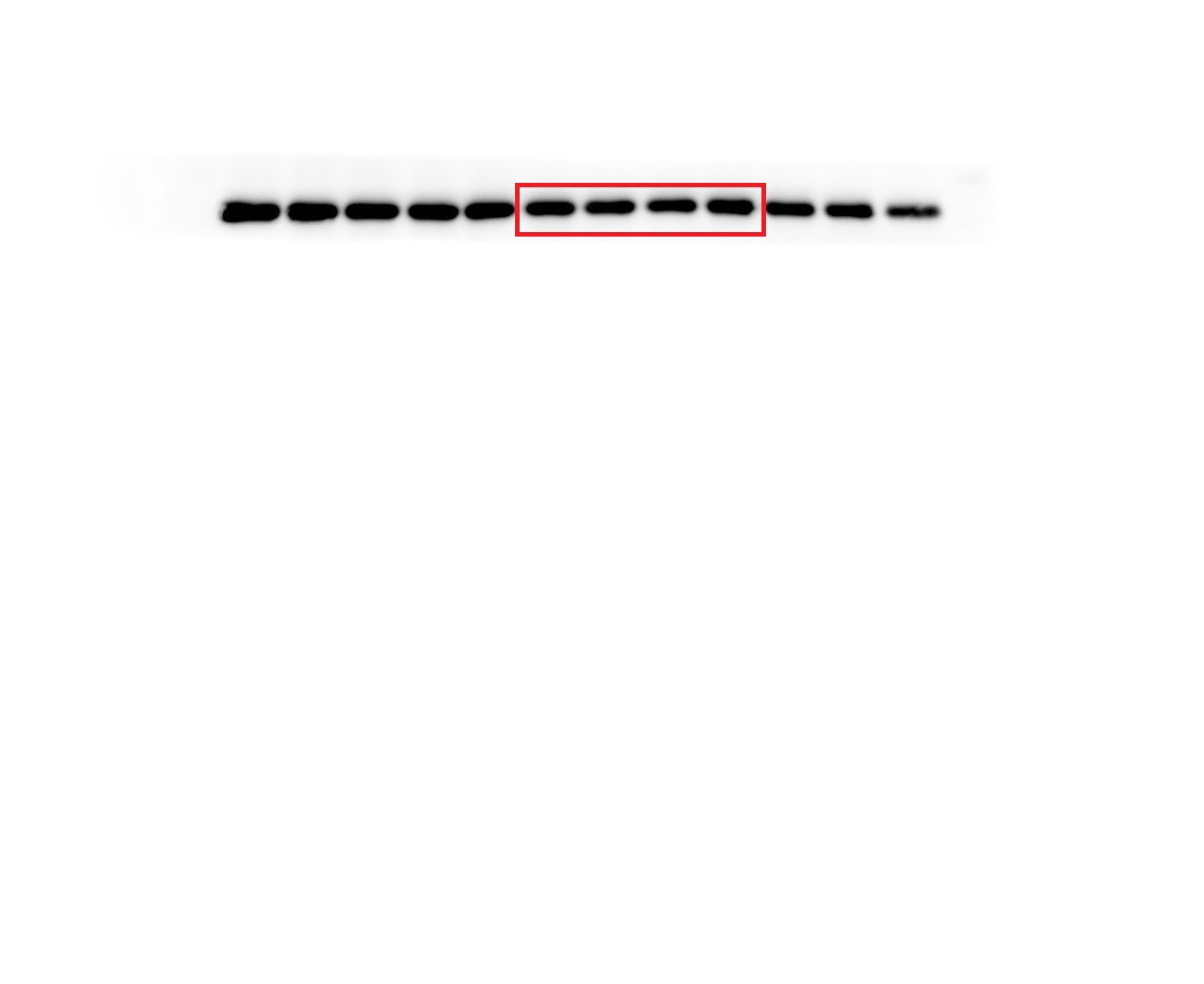

Supplement: Figure 2—figure supplement 2—source data 1. — The individual file name containing ‘(labeled)’ is blot with the relevant bands labeled by a red outline. [file elife-64872-fig2-figsupp2-data1.zip › Figure 2-figure supplement 2-source data 1/GAPDH (labelled).jpg]

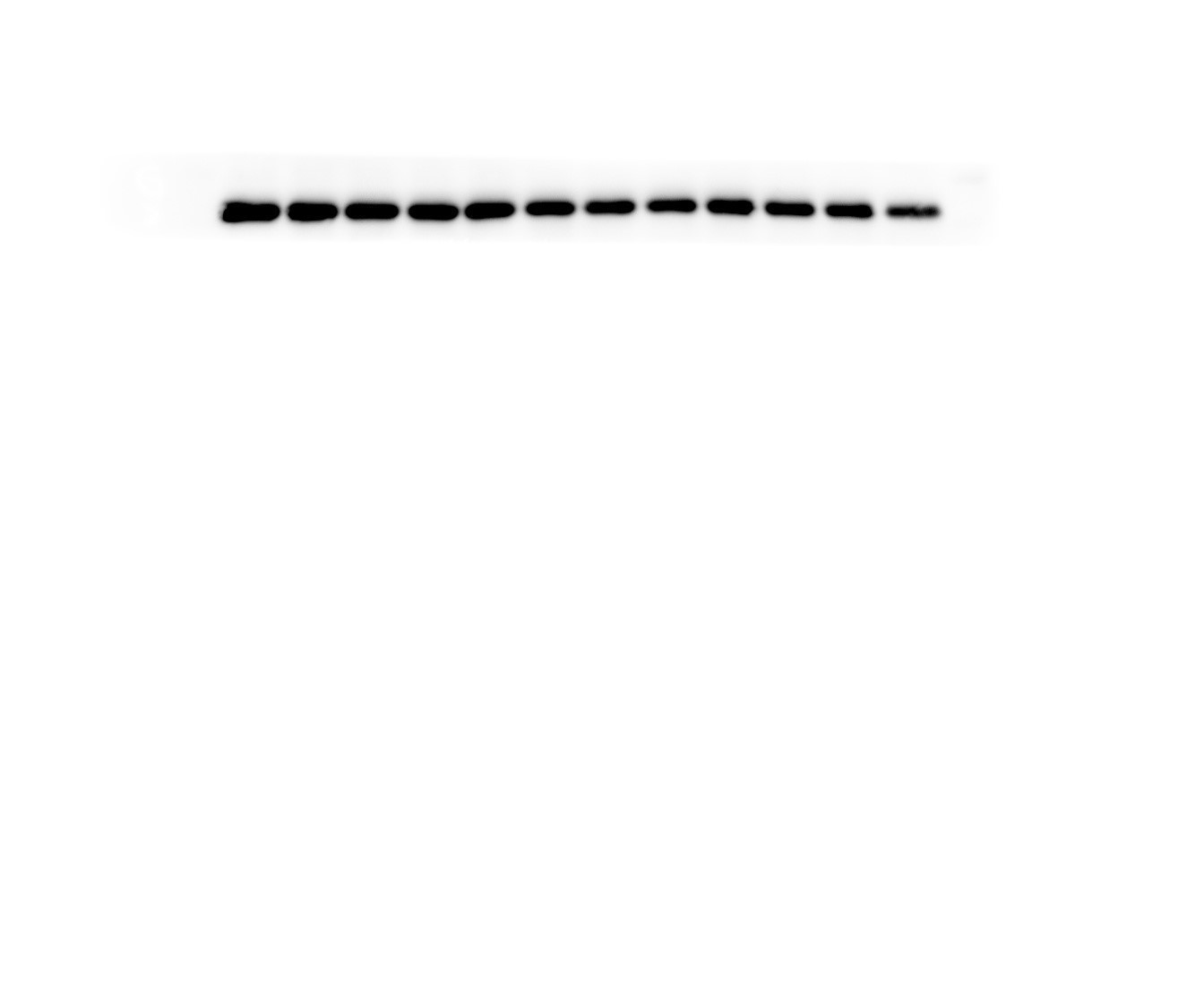

Supplement: Figure 2—figure supplement 2—source data 1. — The individual file name containing ‘(labeled)’ is blot with the relevant bands labeled by a red outline. [file elife-64872-fig2-figsupp2-data1.zip › Figure 2-figure supplement 2-source data 1/GAPDH.jpg]

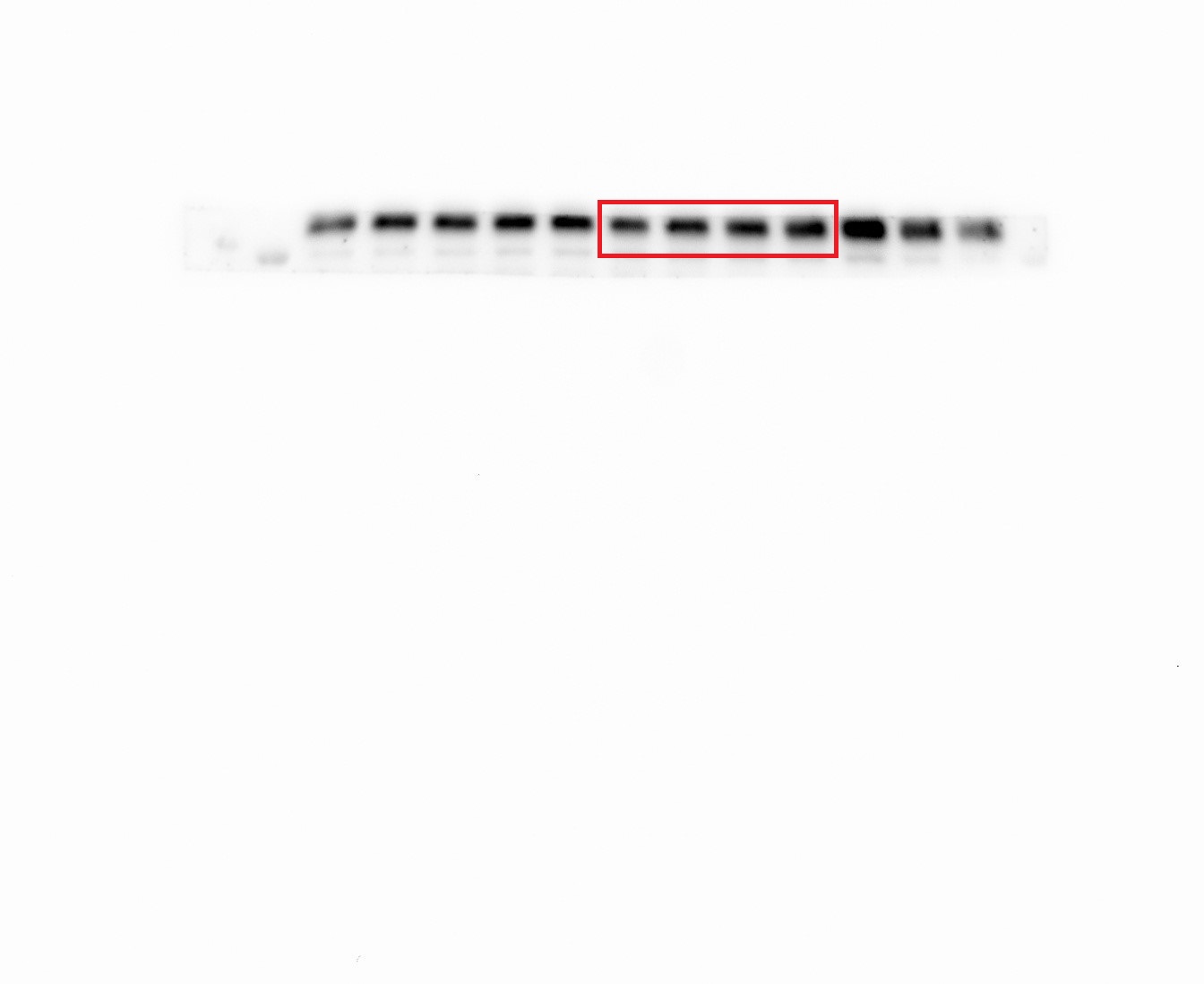

Supplement: Figure 2—figure supplement 2—source data 1. — The individual file name containing ‘(labeled)’ is blot with the relevant bands labeled by a red outline. [file elife-64872-fig2-figsupp2-data1.zip › Figure 2-figure supplement 2-source data 1/p-Smad2 (labelled).jpg]

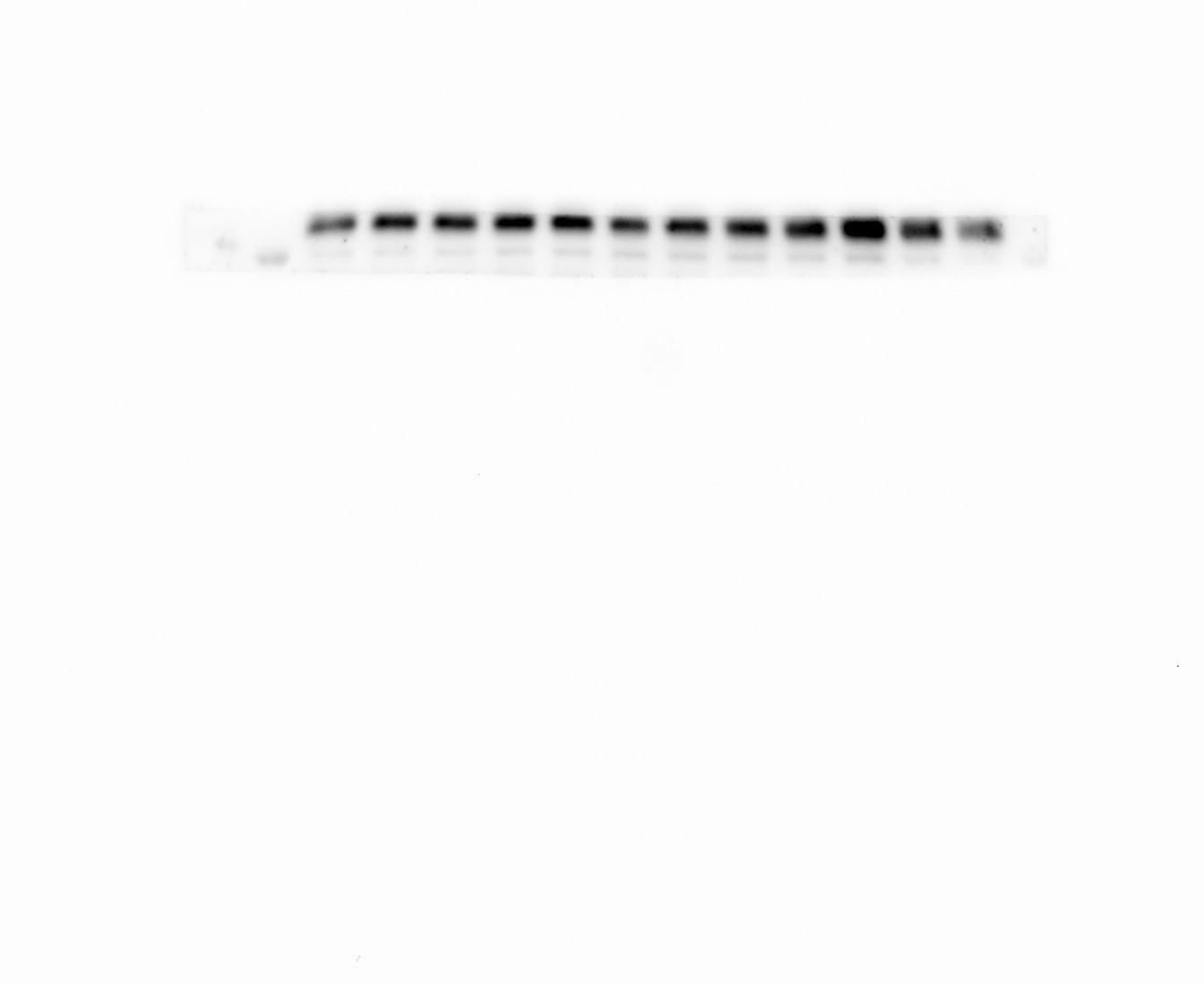

Supplement: Figure 2—figure supplement 2—source data 1. — The individual file name containing ‘(labeled)’ is blot with the relevant bands labeled by a red outline. [file elife-64872-fig2-figsupp2-data1.zip › Figure 2-figure supplement 2-source data 1/p-Smad2.jpg]

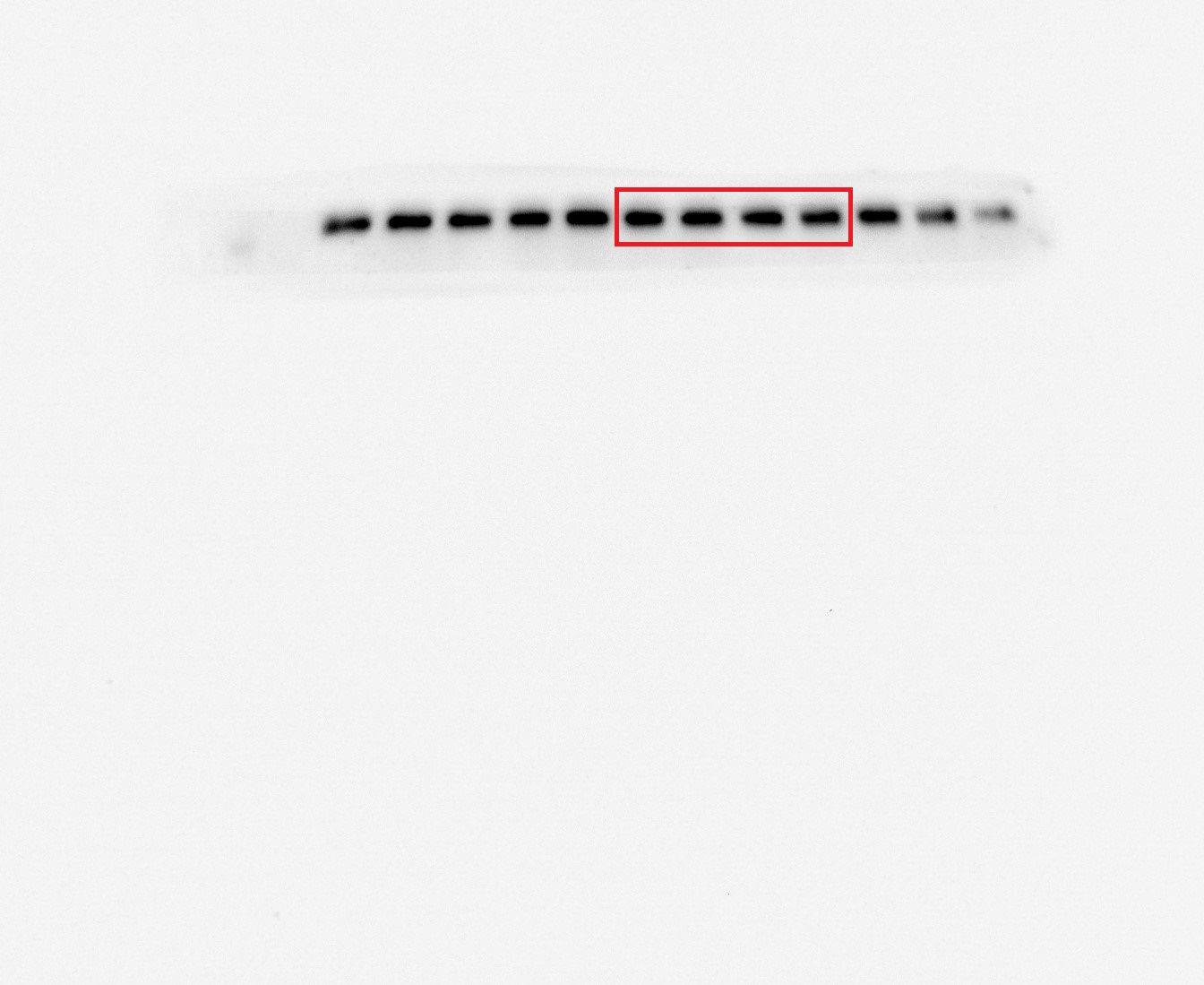

Supplement: Figure 2—figure supplement 2—source data 1. — The individual file name containing ‘(labeled)’ is blot with the relevant bands labeled by a red outline. [file elife-64872-fig2-figsupp2-data1.zip › Figure 2-figure supplement 2-source data 1/Smad2 (labelled).jpg]

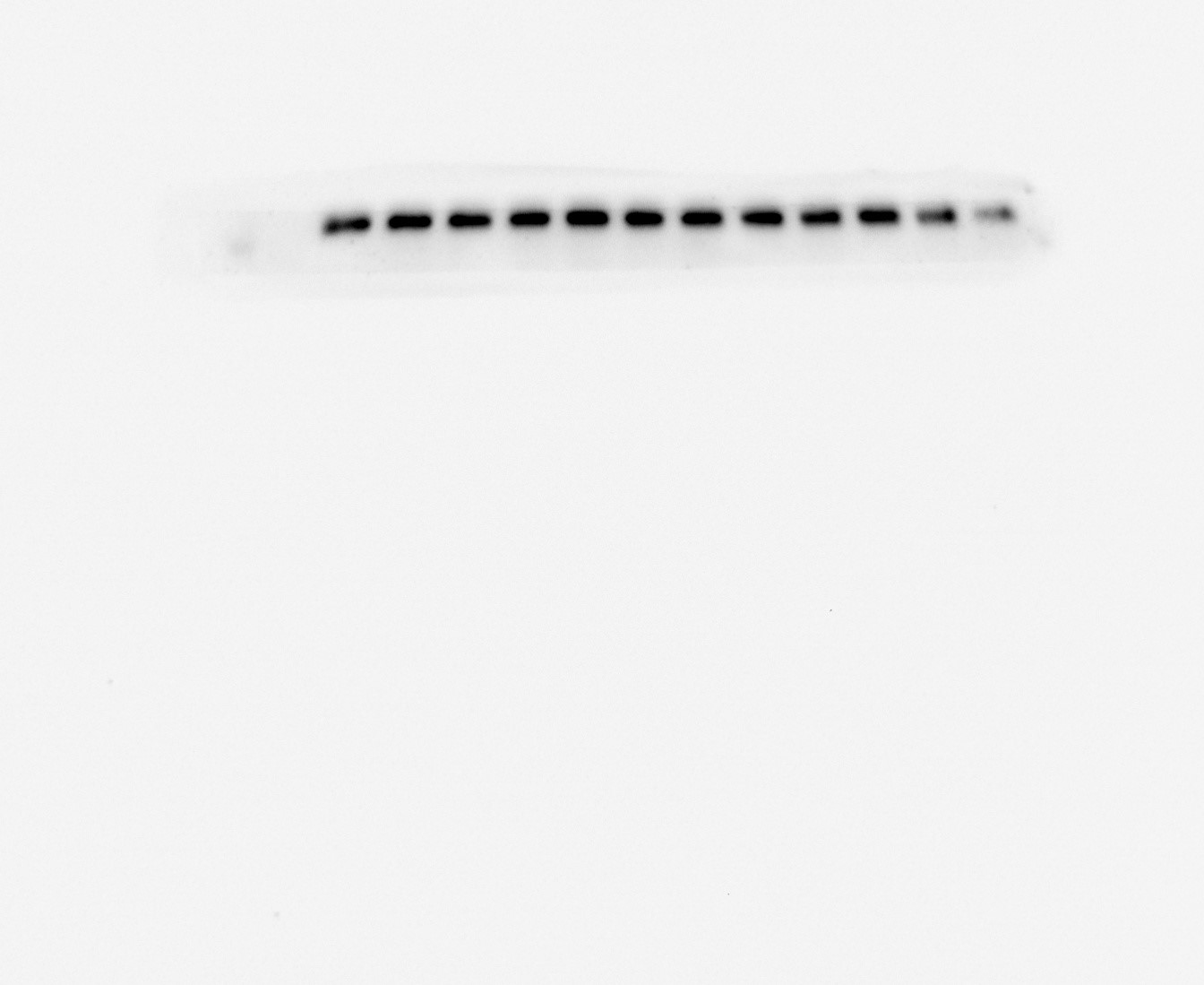

Supplement: Figure 2—figure supplement 2—source data 1. — The individual file name containing ‘(labeled)’ is blot with the relevant bands labeled by a red outline. [file elife-64872-fig2-figsupp2-data1.zip › Figure 2-figure supplement 2-source data 1/Smad2.jpg]

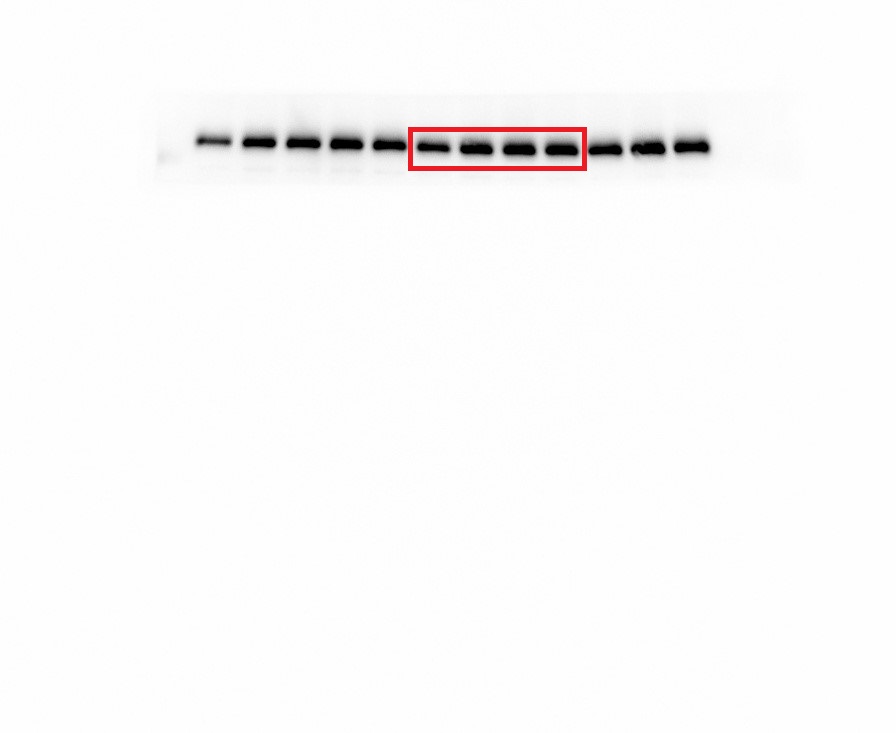

Supplement: Figure 2—figure supplement 2—source data 1. — The individual file name containing ‘(labeled)’ is blot with the relevant bands labeled by a red outline. [file elife-64872-fig2-figsupp2-data1.zip › Figure 2-figure supplement 2-source data 1/TAK1 (labelled).jpg]

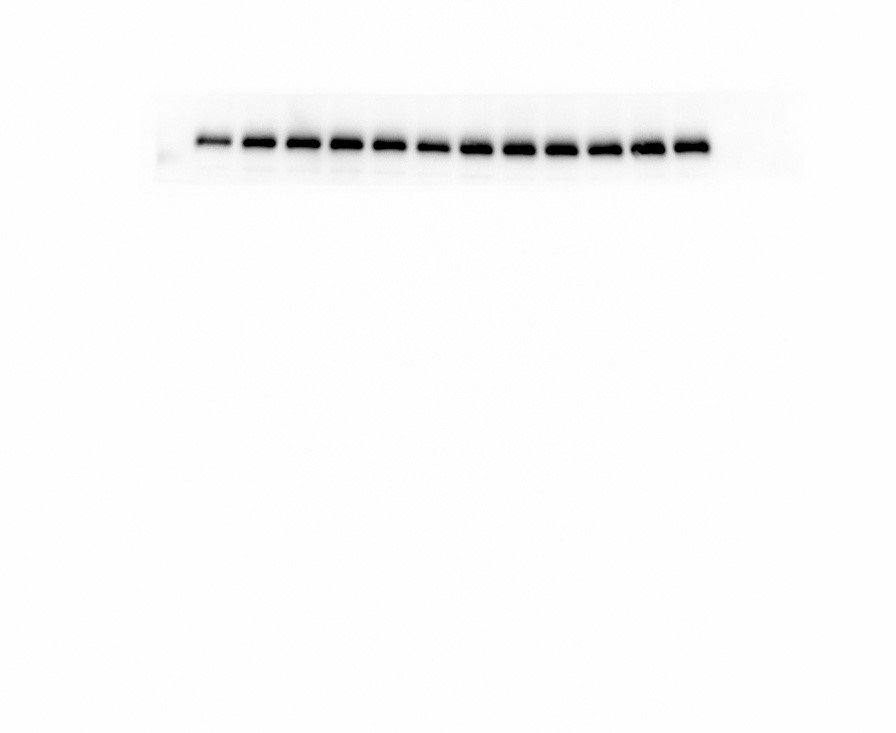

Supplement: Figure 2—figure supplement 2—source data 1. — The individual file name containing ‘(labeled)’ is blot with the relevant bands labeled by a red outline. [file elife-64872-fig2-figsupp2-data1.zip › Figure 2-figure supplement 2-source data 1/TAK1.jpg]

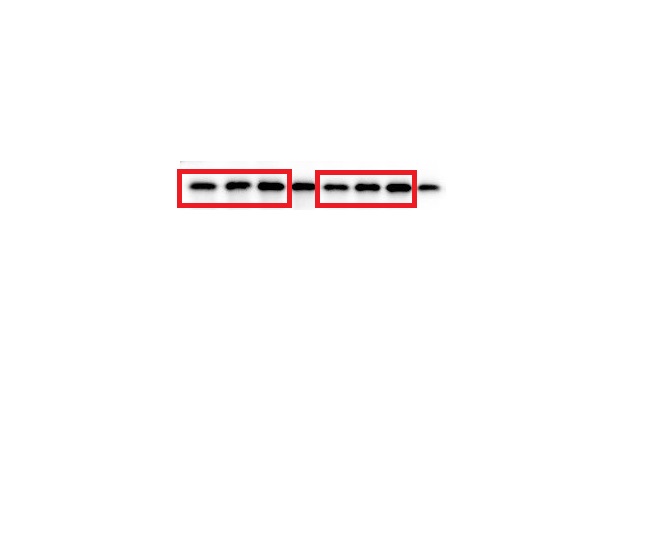

Supplement: Figure 3—source data 1. — The folders named ‘Figure 3C’, ‘Figure 3D’, ‘Figure 3E’, ‘Figure 3F’ and ‘Figure 3G’ contain the original images in Figure 3C, Figure 3D, Figure 3E, Figure 3F and Figure 3G, respectively (the individual file name containing ‘(labeled)’ is blot with the relevant bands labeled by a red outline). [file elife-64872-fig3-data1.zip › Figure 3-source data 1/Figure 3C/GAPDH (labelled).jpg]

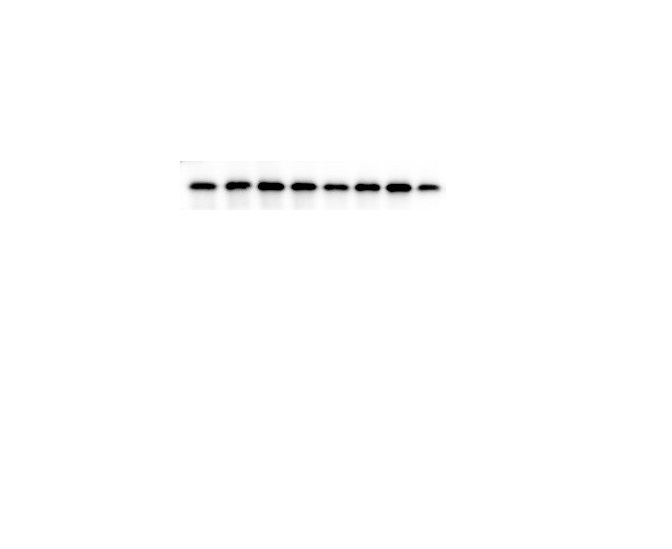

Supplement: Figure 3—source data 1. — The folders named ‘Figure 3C’, ‘Figure 3D’, ‘Figure 3E’, ‘Figure 3F’ and ‘Figure 3G’ contain the original images in Figure 3C, Figure 3D, Figure 3E, Figure 3F and Figure 3G, respectively (the individual file name containing ‘(labeled)’ is blot with the relevant bands labeled by a red outline). [file elife-64872-fig3-data1.zip › Figure 3-source data 1/Figure 3C/GAPDH.jpg]

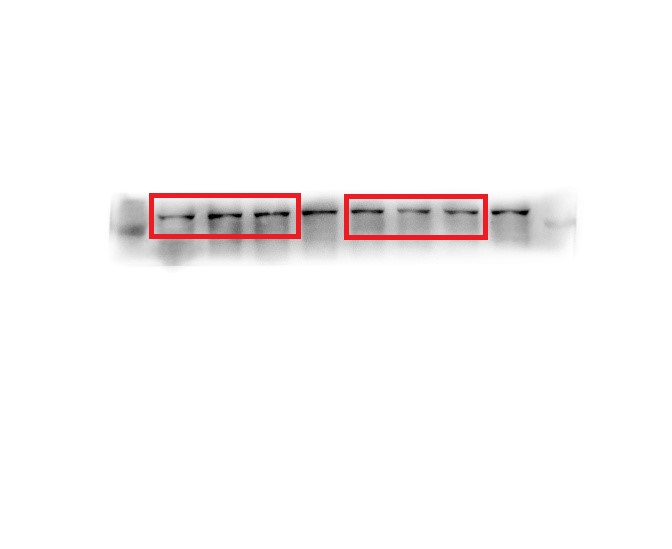

Supplement: Figure 3—source data 1. — The folders named ‘Figure 3C’, ‘Figure 3D’, ‘Figure 3E’, ‘Figure 3F’ and ‘Figure 3G’ contain the original images in Figure 3C, Figure 3D, Figure 3E, Figure 3F and Figure 3G, respectively (the individual file name containing ‘(labeled)’ is blot with the relevant bands labeled by a red outline). [file elife-64872-fig3-data1.zip › Figure 3-source data 1/Figure 3C/p-TrkB (labelled).jpg]

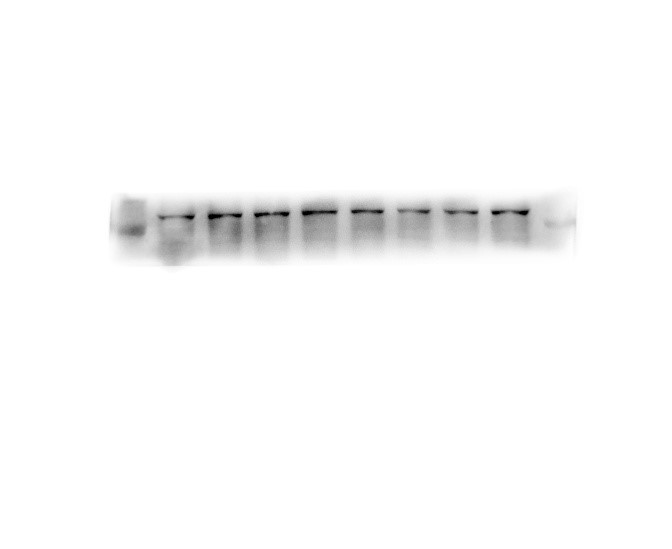

Supplement: Figure 3—source data 1. — The folders named ‘Figure 3C’, ‘Figure 3D’, ‘Figure 3E’, ‘Figure 3F’ and ‘Figure 3G’ contain the original images in Figure 3C, Figure 3D, Figure 3E, Figure 3F and Figure 3G, respectively (the individual file name containing ‘(labeled)’ is blot with the relevant bands labeled by a red outline). [file elife-64872-fig3-data1.zip › Figure 3-source data 1/Figure 3C/p-TrkB.jpg]

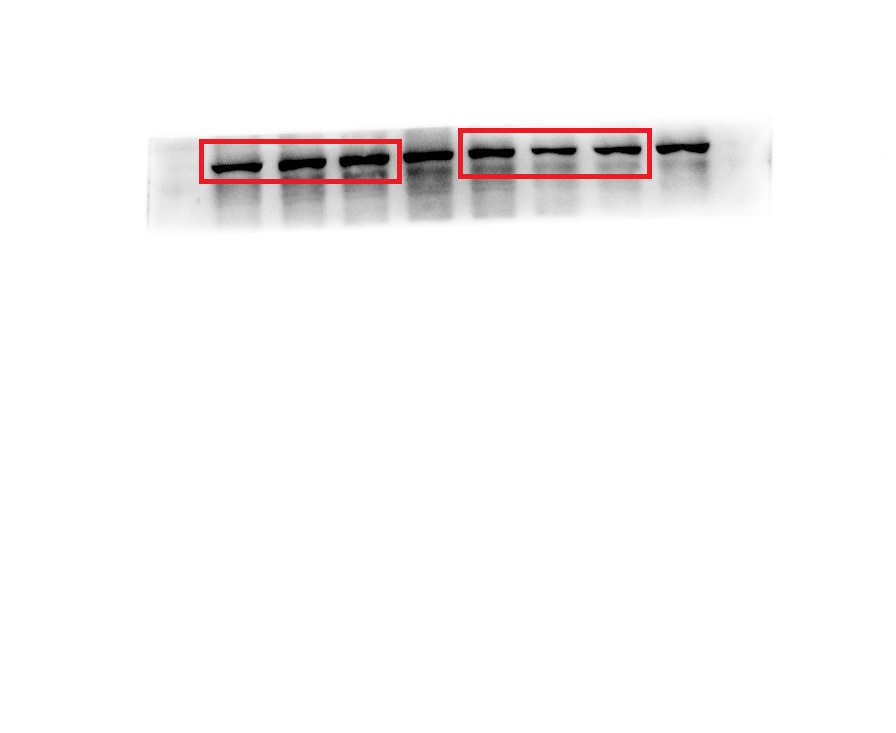

Supplement: Figure 3—source data 1. — The folders named ‘Figure 3C’, ‘Figure 3D’, ‘Figure 3E’, ‘Figure 3F’ and ‘Figure 3G’ contain the original images in Figure 3C, Figure 3D, Figure 3E, Figure 3F and Figure 3G, respectively (the individual file name containing ‘(labeled)’ is blot with the relevant bands labeled by a red outline). [file elife-64872-fig3-data1.zip › Figure 3-source data 1/Figure 3C/TrkB (labelled).jpg]

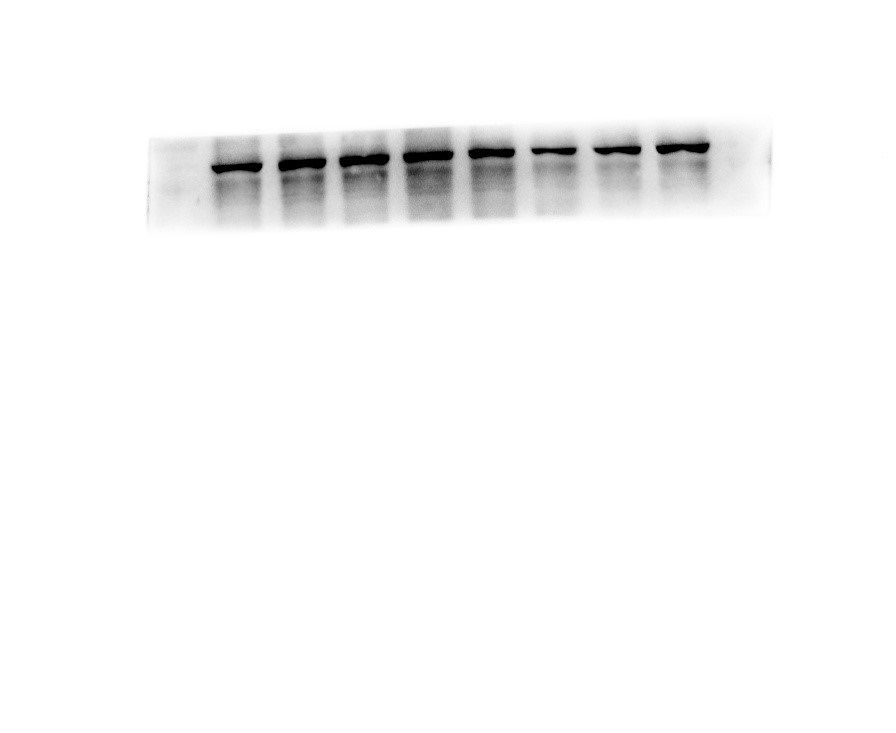

Supplement: Figure 3—source data 1. — The folders named ‘Figure 3C’, ‘Figure 3D’, ‘Figure 3E’, ‘Figure 3F’ and ‘Figure 3G’ contain the original images in Figure 3C, Figure 3D, Figure 3E, Figure 3F and Figure 3G, respectively (the individual file name containing ‘(labeled)’ is blot with the relevant bands labeled by a red outline). [file elife-64872-fig3-data1.zip › Figure 3-source data 1/Figure 3C/TrkB.jpg]

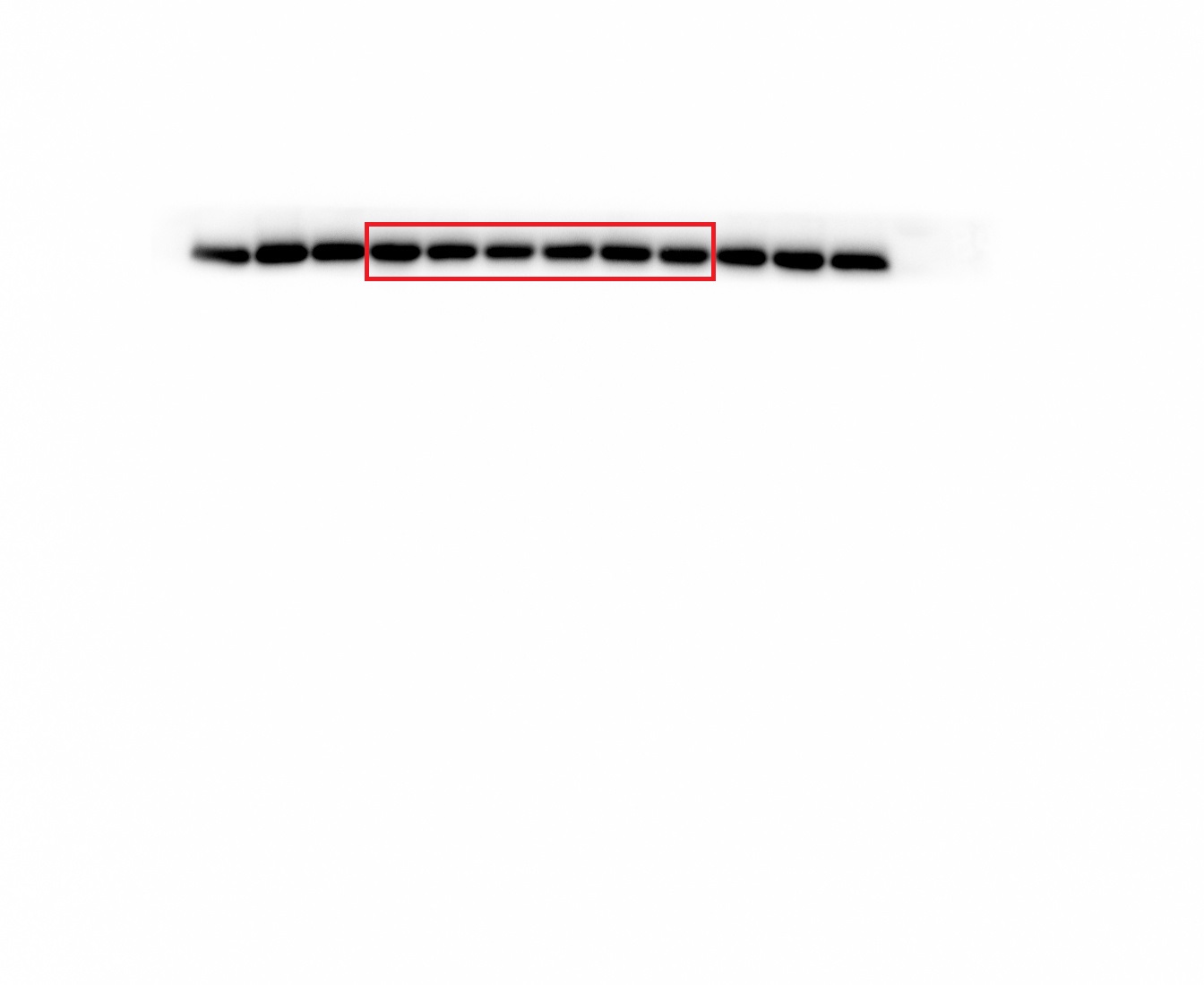

Supplement: Figure 3—source data 1. — The folders named ‘Figure 3C’, ‘Figure 3D’, ‘Figure 3E’, ‘Figure 3F’ and ‘Figure 3G’ contain the original images in Figure 3C, Figure 3D, Figure 3E, Figure 3F and Figure 3G, respectively (the individual file name containing ‘(labeled)’ is blot with the relevant bands labeled by a red outline). [file elife-64872-fig3-data1.zip › Figure 3-source data 1/Figure 3D/GAPDH (labelled).jpg]

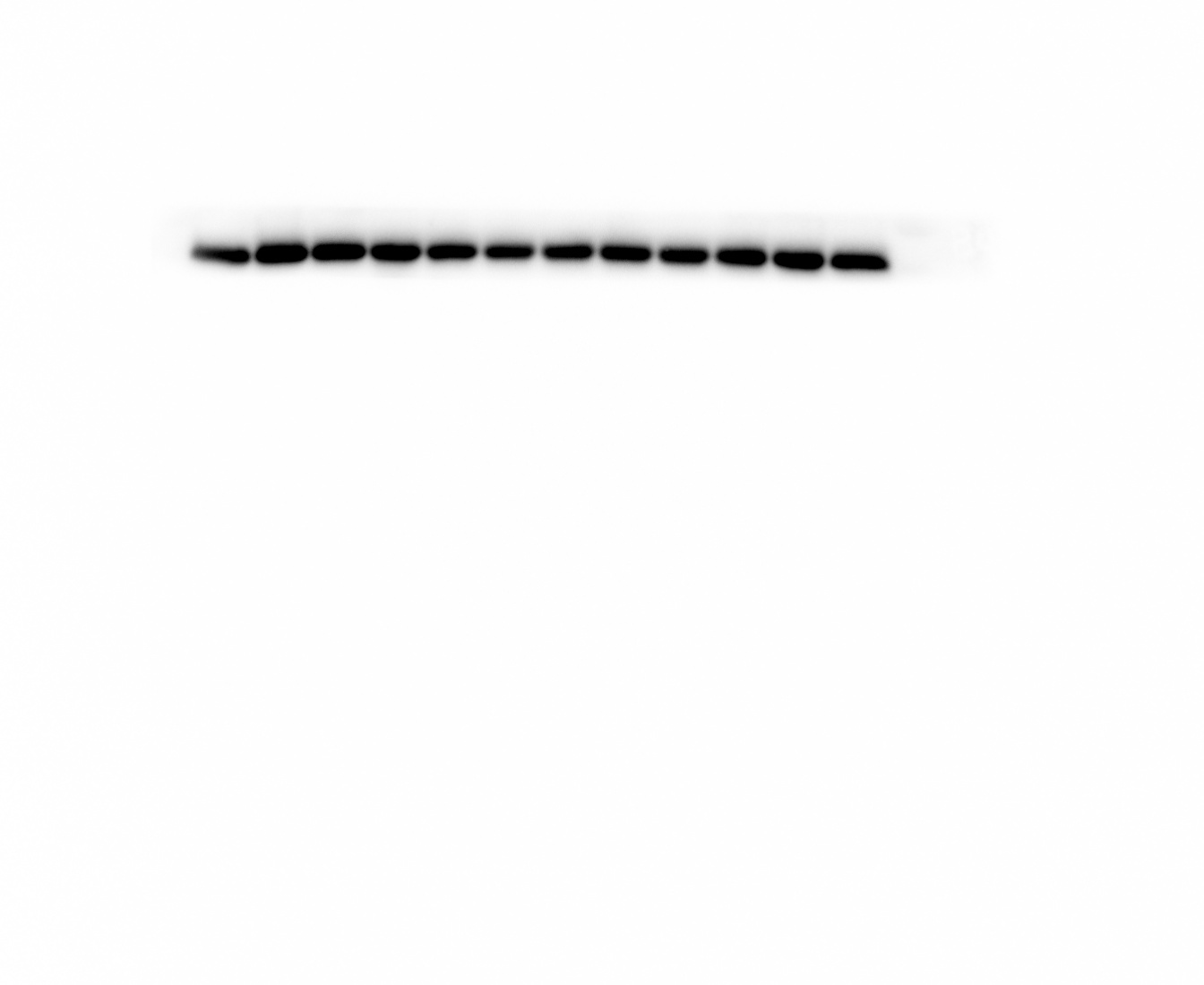

Supplement: Figure 3—source data 1. — The folders named ‘Figure 3C’, ‘Figure 3D’, ‘Figure 3E’, ‘Figure 3F’ and ‘Figure 3G’ contain the original images in Figure 3C, Figure 3D, Figure 3E, Figure 3F and Figure 3G, respectively (the individual file name containing ‘(labeled)’ is blot with the relevant bands labeled by a red outline). [file elife-64872-fig3-data1.zip › Figure 3-source data 1/Figure 3D/GAPDH.jpg]

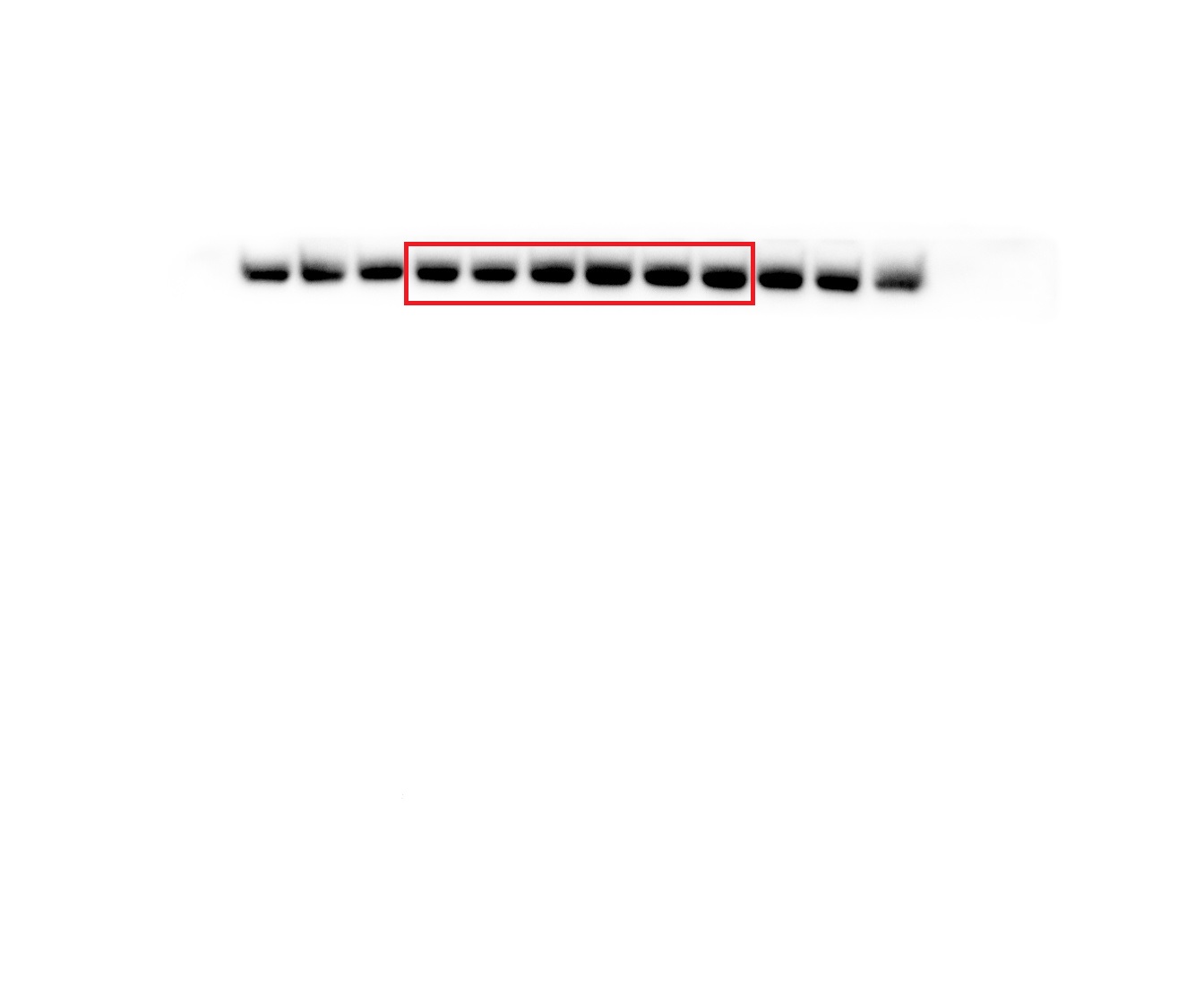

Supplement: Figure 3—source data 1. — The folders named ‘Figure 3C’, ‘Figure 3D’, ‘Figure 3E’, ‘Figure 3F’ and ‘Figure 3G’ contain the original images in Figure 3C, Figure 3D, Figure 3E, Figure 3F and Figure 3G, respectively (the individual file name containing ‘(labeled)’ is blot with the relevant bands labeled by a red outline). [file elife-64872-fig3-data1.zip › Figure 3-source data 1/Figure 3D/GSK3β (labelled).jpg]

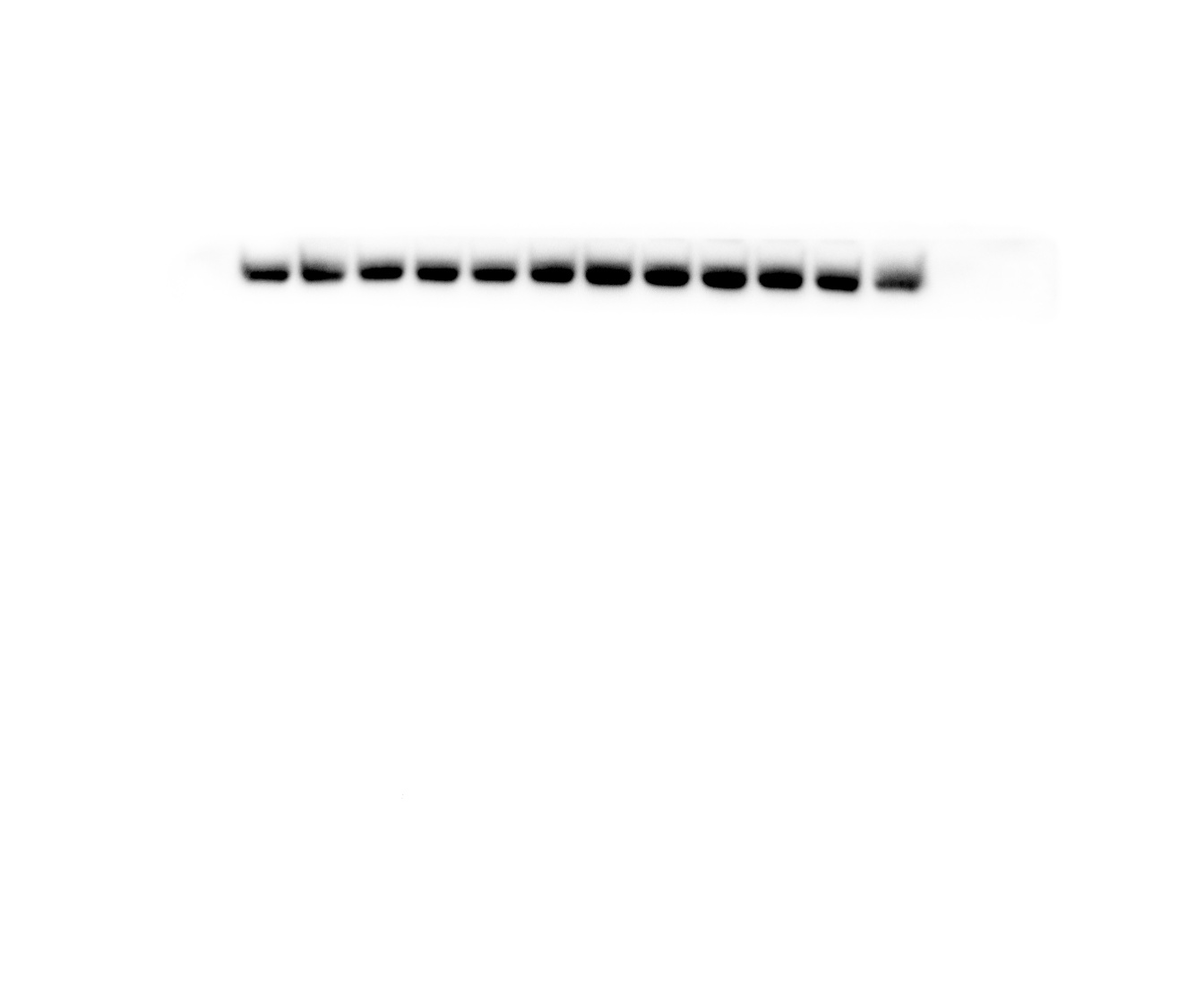

Supplement: Figure 3—source data 1. — The folders named ‘Figure 3C’, ‘Figure 3D’, ‘Figure 3E’, ‘Figure 3F’ and ‘Figure 3G’ contain the original images in Figure 3C, Figure 3D, Figure 3E, Figure 3F and Figure 3G, respectively (the individual file name containing ‘(labeled)’ is blot with the relevant bands labeled by a red outline). [file elife-64872-fig3-data1.zip › Figure 3-source data 1/Figure 3D/GSK3β.jpg]

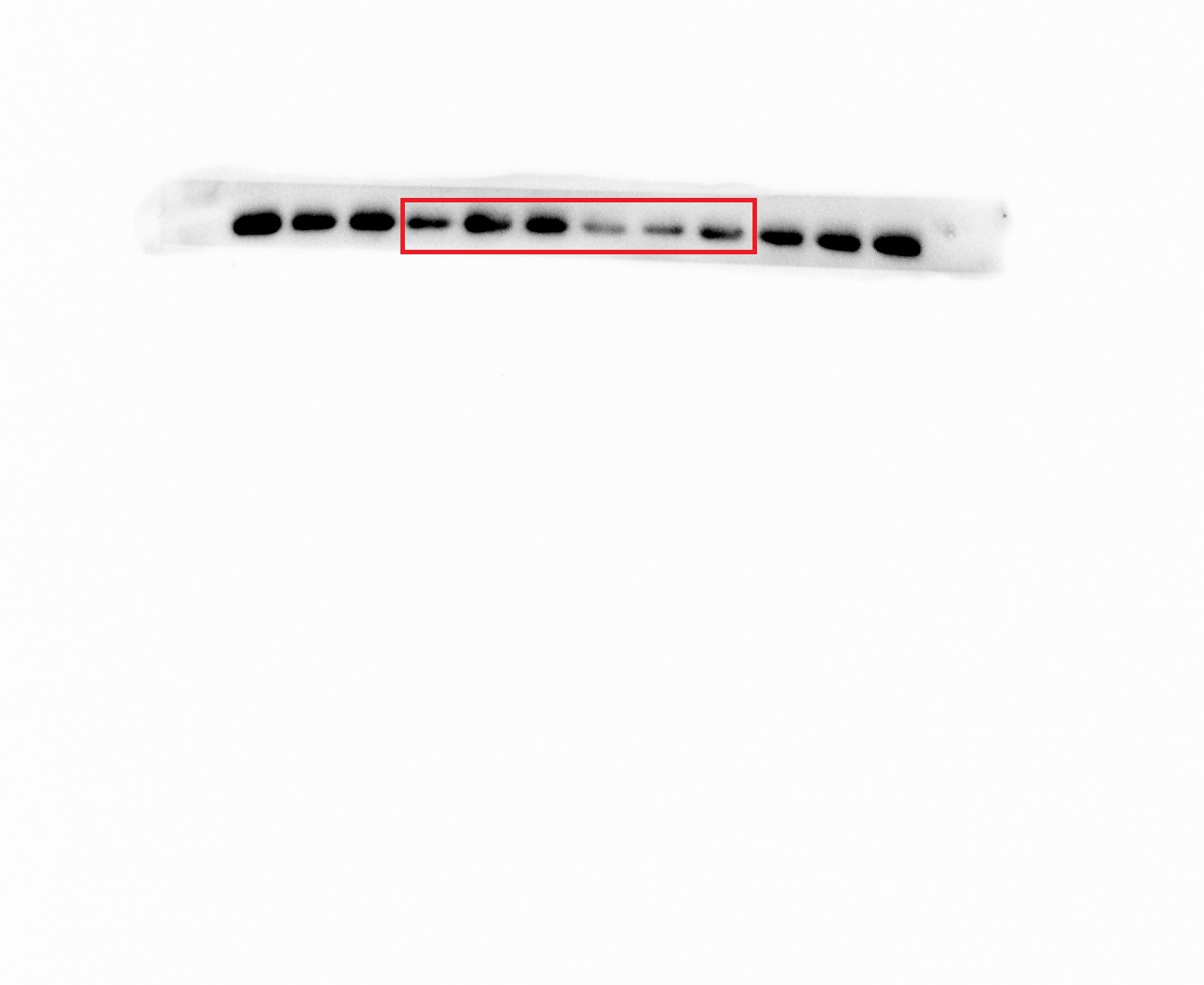

Supplement: Figure 3—source data 1. — The folders named ‘Figure 3C’, ‘Figure 3D’, ‘Figure 3E’, ‘Figure 3F’ and ‘Figure 3G’ contain the original images in Figure 3C, Figure 3D, Figure 3E, Figure 3F and Figure 3G, respectively (the individual file name containing ‘(labeled)’ is blot with the relevant bands labeled by a red outline). [file elife-64872-fig3-data1.zip › Figure 3-source data 1/Figure 3D/p-GSK3β (labelled).jpg]

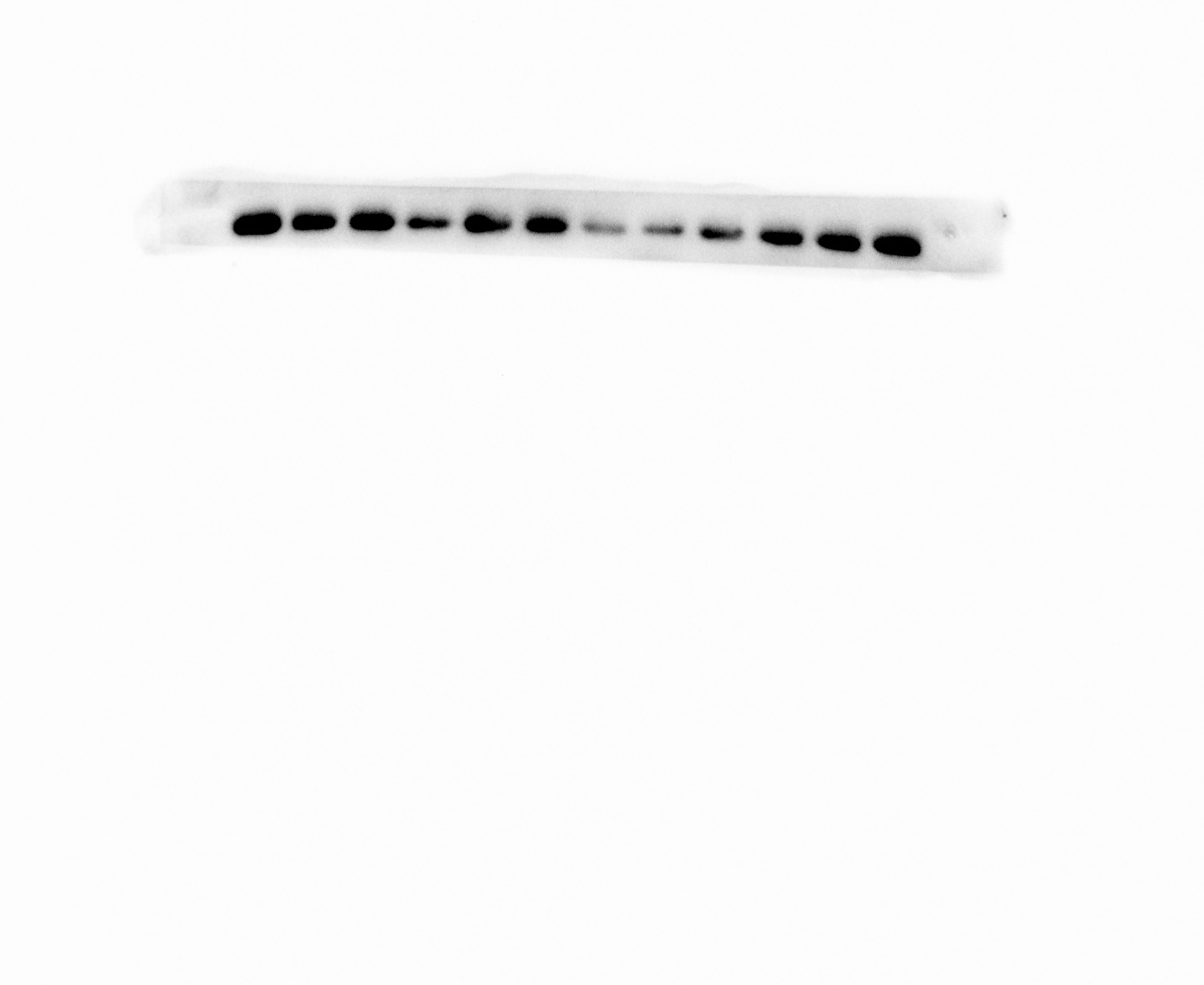

Supplement: Figure 3—source data 1. — The folders named ‘Figure 3C’, ‘Figure 3D’, ‘Figure 3E’, ‘Figure 3F’ and ‘Figure 3G’ contain the original images in Figure 3C, Figure 3D, Figure 3E, Figure 3F and Figure 3G, respectively (the individual file name containing ‘(labeled)’ is blot with the relevant bands labeled by a red outline). [file elife-64872-fig3-data1.zip › Figure 3-source data 1/Figure 3D/p-GSK3β.jpg]

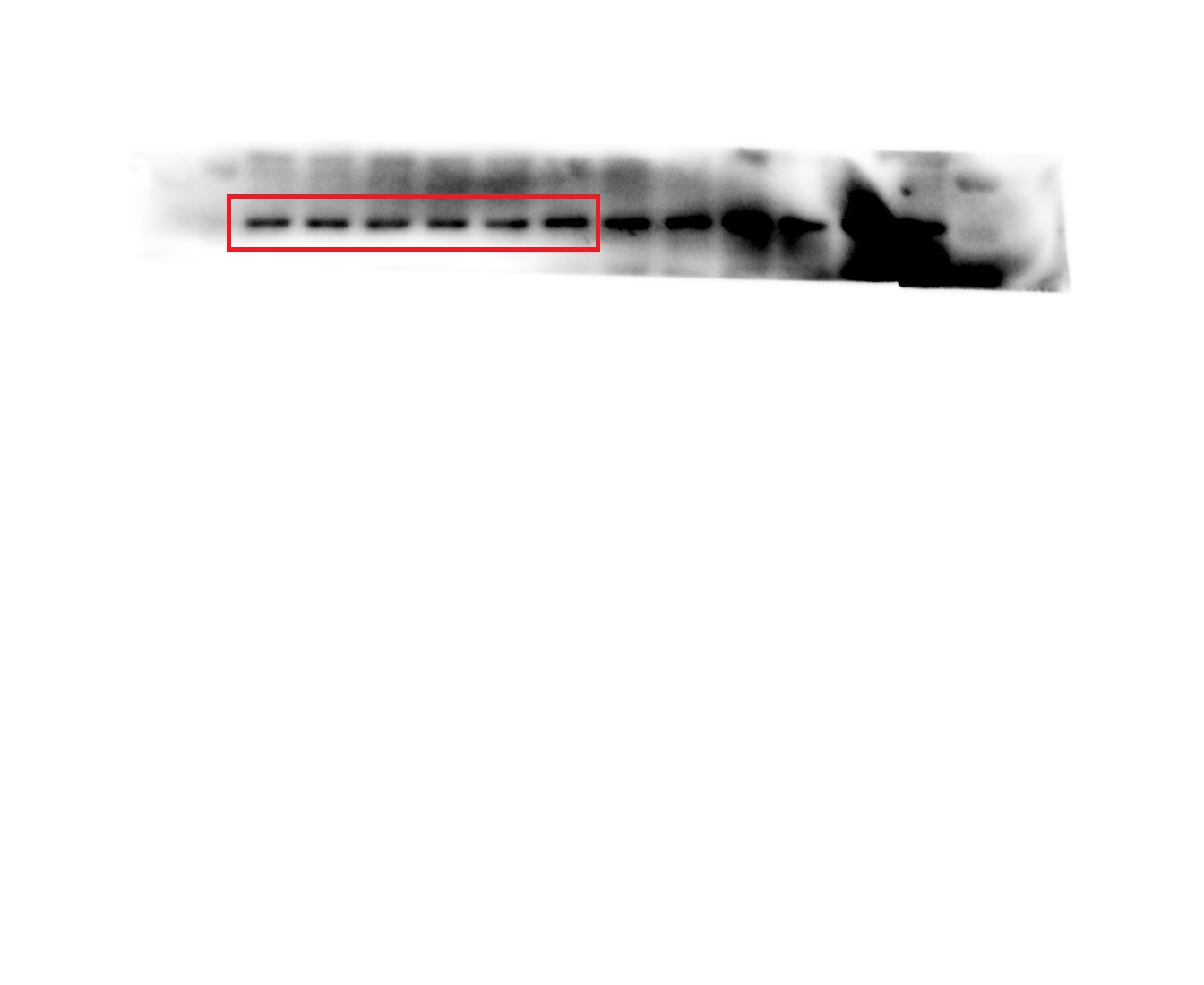

Supplement: Figure 3—source data 1. — The folders named ‘Figure 3C’, ‘Figure 3D’, ‘Figure 3E’, ‘Figure 3F’ and ‘Figure 3G’ contain the original images in Figure 3C, Figure 3D, Figure 3E, Figure 3F and Figure 3G, respectively (the individual file name containing ‘(labeled)’ is blot with the relevant bands labeled by a red outline). [file elife-64872-fig3-data1.zip › Figure 3-source data 1/Figure 3E/GAPDH (labelled).jpg]

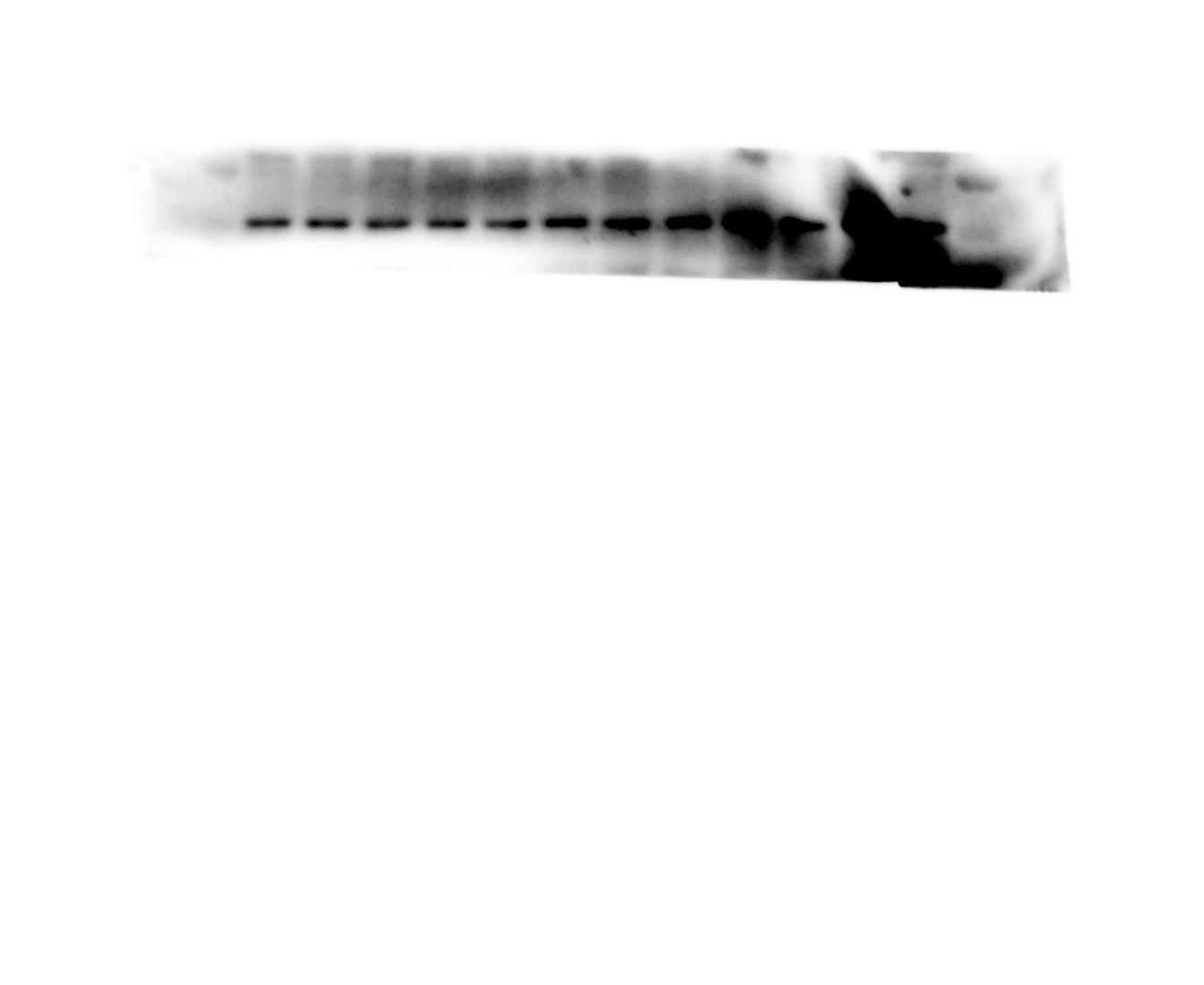

Supplement: Figure 3—source data 1. — The folders named ‘Figure 3C’, ‘Figure 3D’, ‘Figure 3E’, ‘Figure 3F’ and ‘Figure 3G’ contain the original images in Figure 3C, Figure 3D, Figure 3E, Figure 3F and Figure 3G, respectively (the individual file name containing ‘(labeled)’ is blot with the relevant bands labeled by a red outline). [file elife-64872-fig3-data1.zip › Figure 3-source data 1/Figure 3E/GAPDH.jpg]

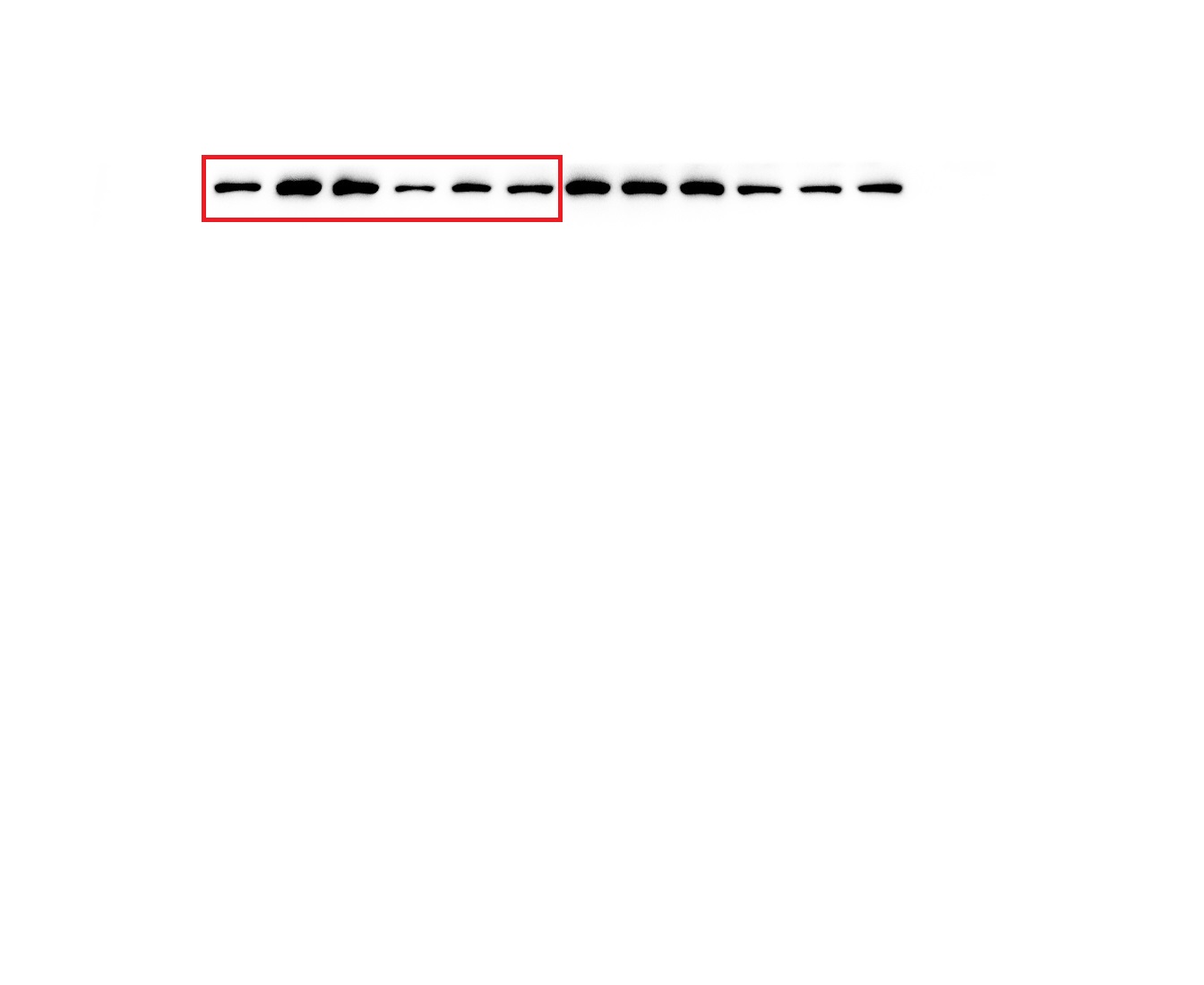

Supplement: Figure 3—source data 1. — The folders named ‘Figure 3C’, ‘Figure 3D’, ‘Figure 3E’, ‘Figure 3F’ and ‘Figure 3G’ contain the original images in Figure 3C, Figure 3D, Figure 3E, Figure 3F and Figure 3G, respectively (the individual file name containing ‘(labeled)’ is blot with the relevant bands labeled by a red outline). [file elife-64872-fig3-data1.zip › Figure 3-source data 1/Figure 3E/β-catenin (labelled).jpg]

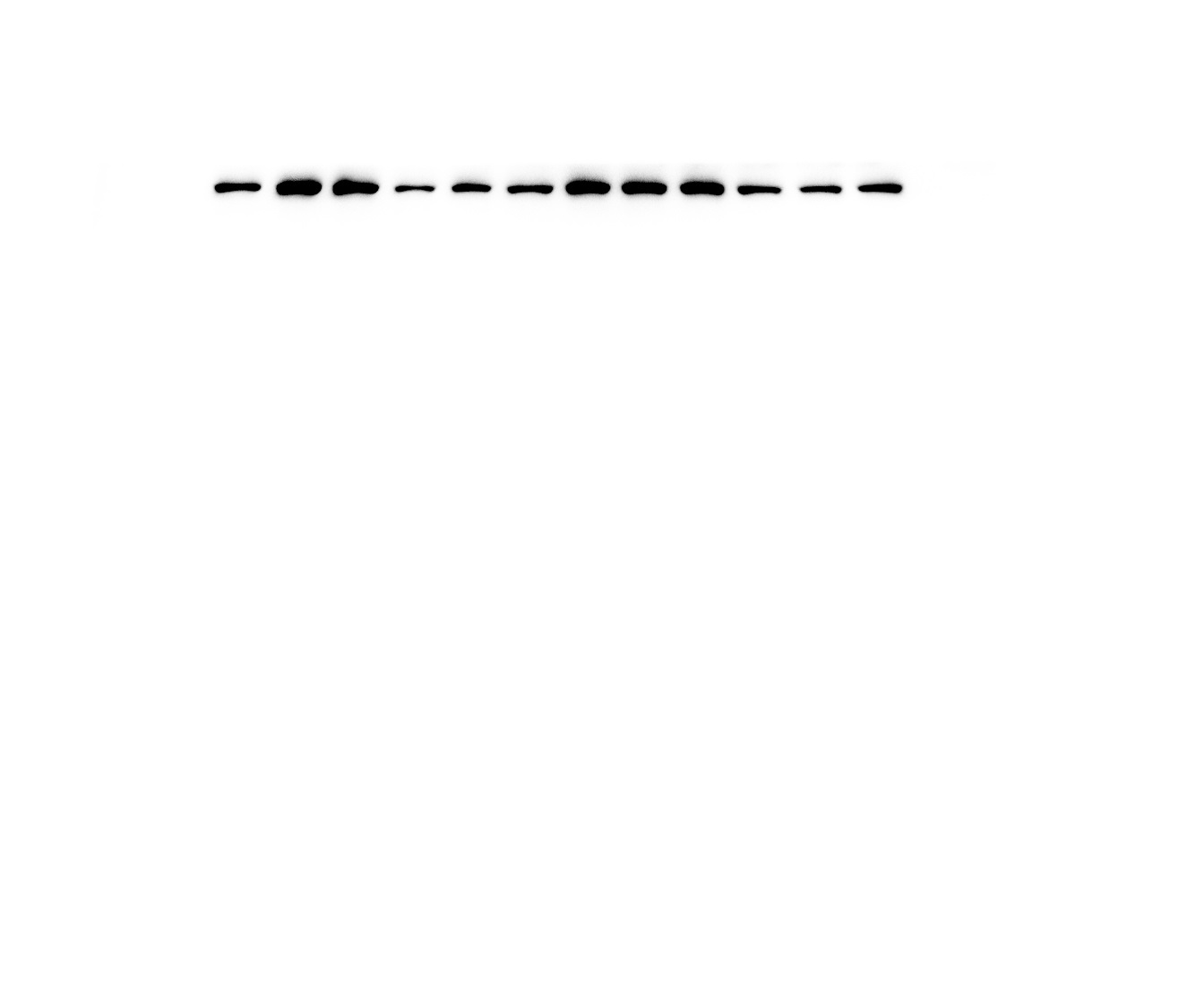

Supplement: Figure 3—source data 1. — The folders named ‘Figure 3C’, ‘Figure 3D’, ‘Figure 3E’, ‘Figure 3F’ and ‘Figure 3G’ contain the original images in Figure 3C, Figure 3D, Figure 3E, Figure 3F and Figure 3G, respectively (the individual file name containing ‘(labeled)’ is blot with the relevant bands labeled by a red outline). [file elife-64872-fig3-data1.zip › Figure 3-source data 1/Figure 3E/β-catenin.jpg]

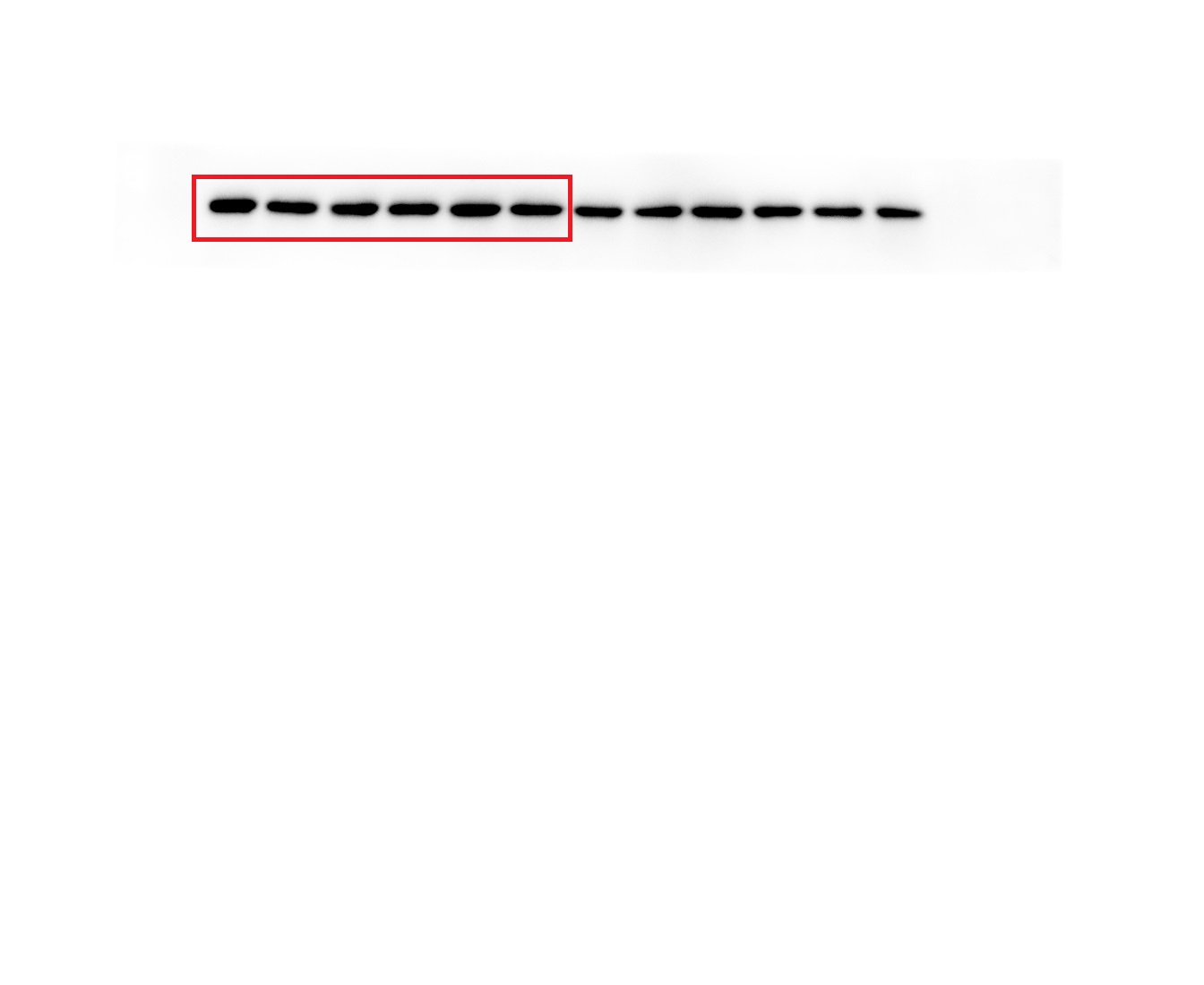

Supplement: Figure 3—source data 1. — The folders named ‘Figure 3C’, ‘Figure 3D’, ‘Figure 3E’, ‘Figure 3F’ and ‘Figure 3G’ contain the original images in Figure 3C, Figure 3D, Figure 3E, Figure 3F and Figure 3G, respectively (the individual file name containing ‘(labeled)’ is blot with the relevant bands labeled by a red outline). [file elife-64872-fig3-data1.zip › Figure 3-source data 1/Figure 3F/GAPDH (labelled).jpg]

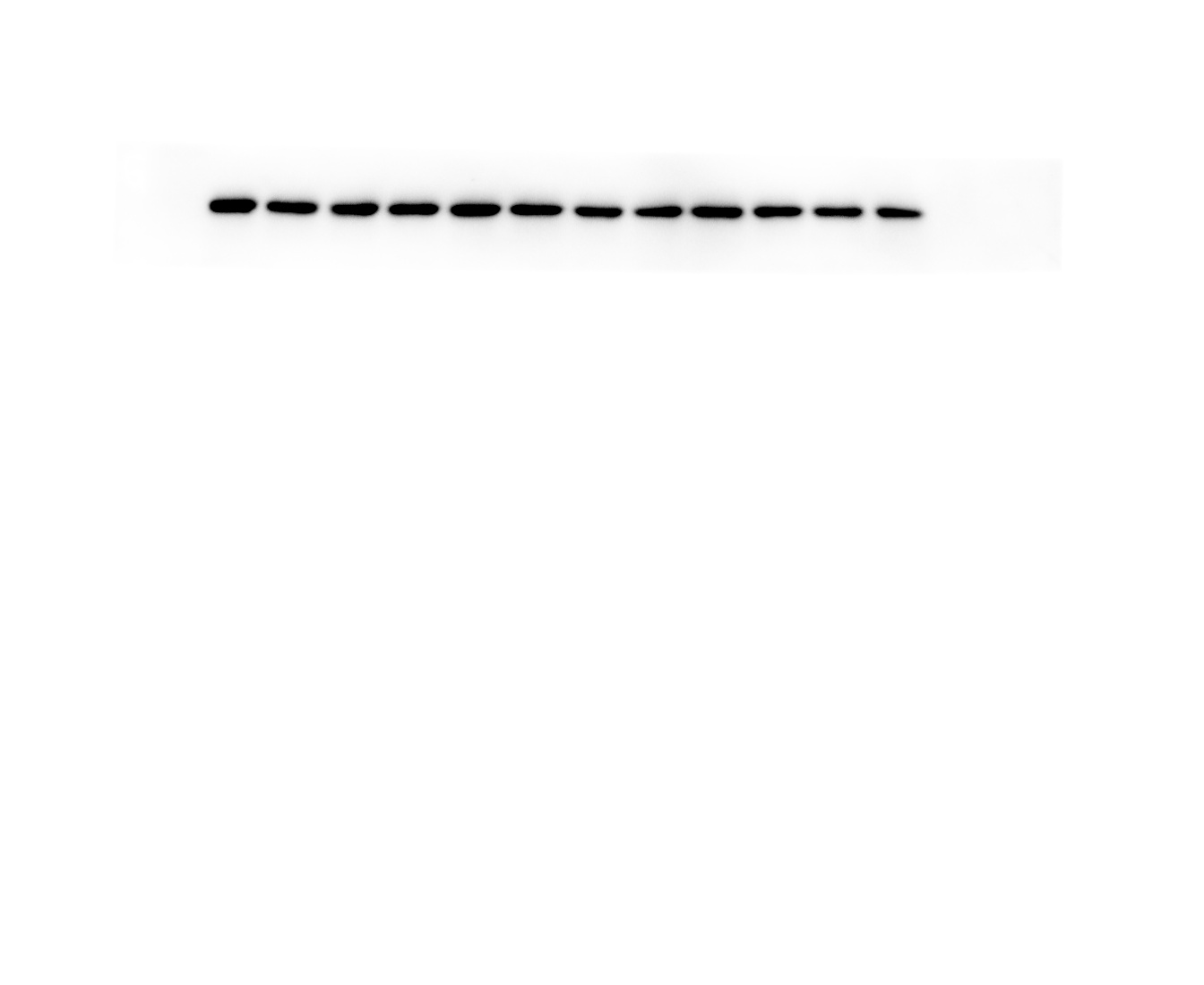

Supplement: Figure 3—source data 1. — The folders named ‘Figure 3C’, ‘Figure 3D’, ‘Figure 3E’, ‘Figure 3F’ and ‘Figure 3G’ contain the original images in Figure 3C, Figure 3D, Figure 3E, Figure 3F and Figure 3G, respectively (the individual file name containing ‘(labeled)’ is blot with the relevant bands labeled by a red outline). [file elife-64872-fig3-data1.zip › Figure 3-source data 1/Figure 3F/GAPDH.jpg]

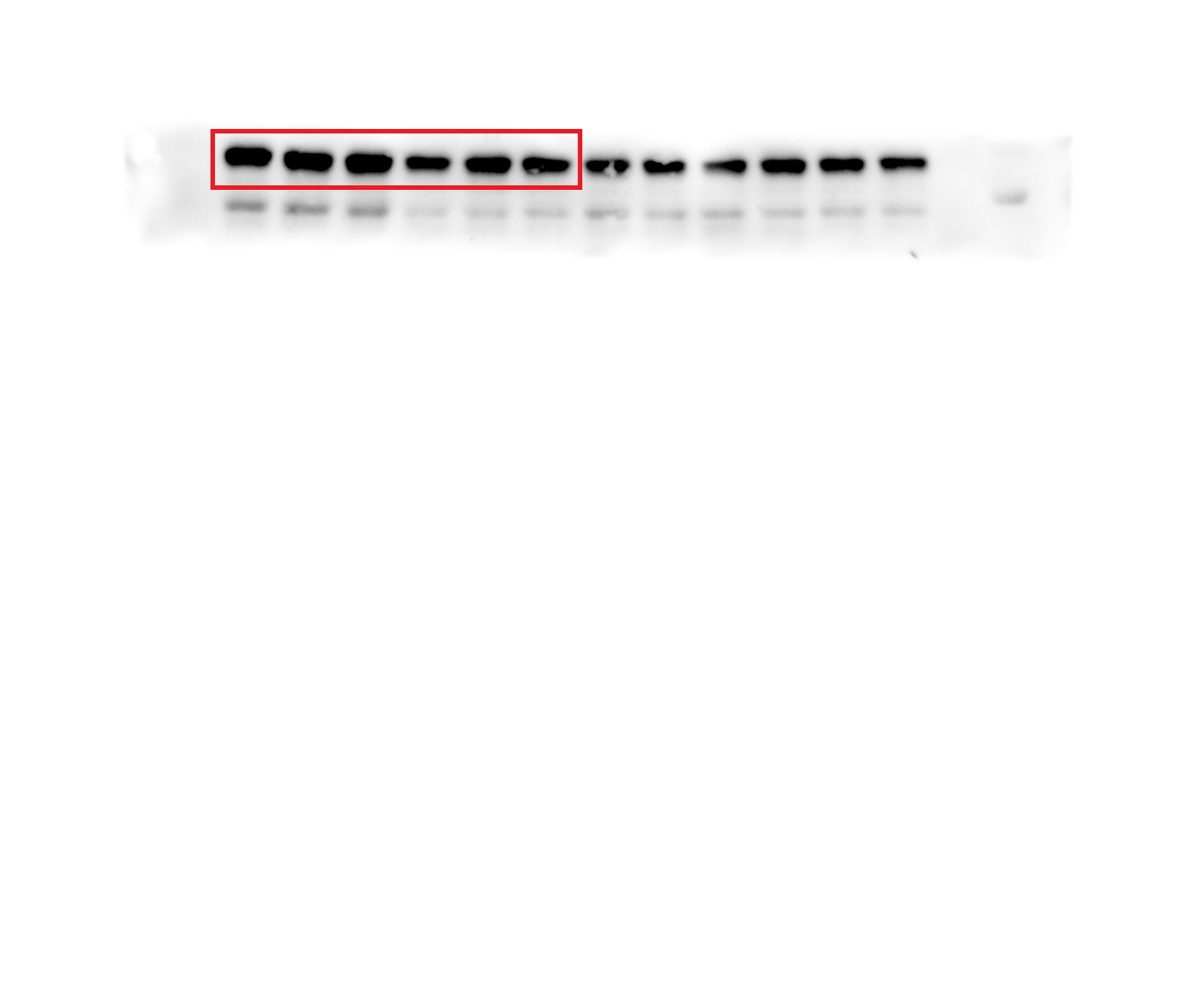

Supplement: Figure 3—source data 1. — The folders named ‘Figure 3C’, ‘Figure 3D’, ‘Figure 3E’, ‘Figure 3F’ and ‘Figure 3G’ contain the original images in Figure 3C, Figure 3D, Figure 3E, Figure 3F and Figure 3G, respectively (the individual file name containing ‘(labeled)’ is blot with the relevant bands labeled by a red outline). [file elife-64872-fig3-data1.zip › Figure 3-source data 1/Figure 3F/Runx2 (labelled).jpg]

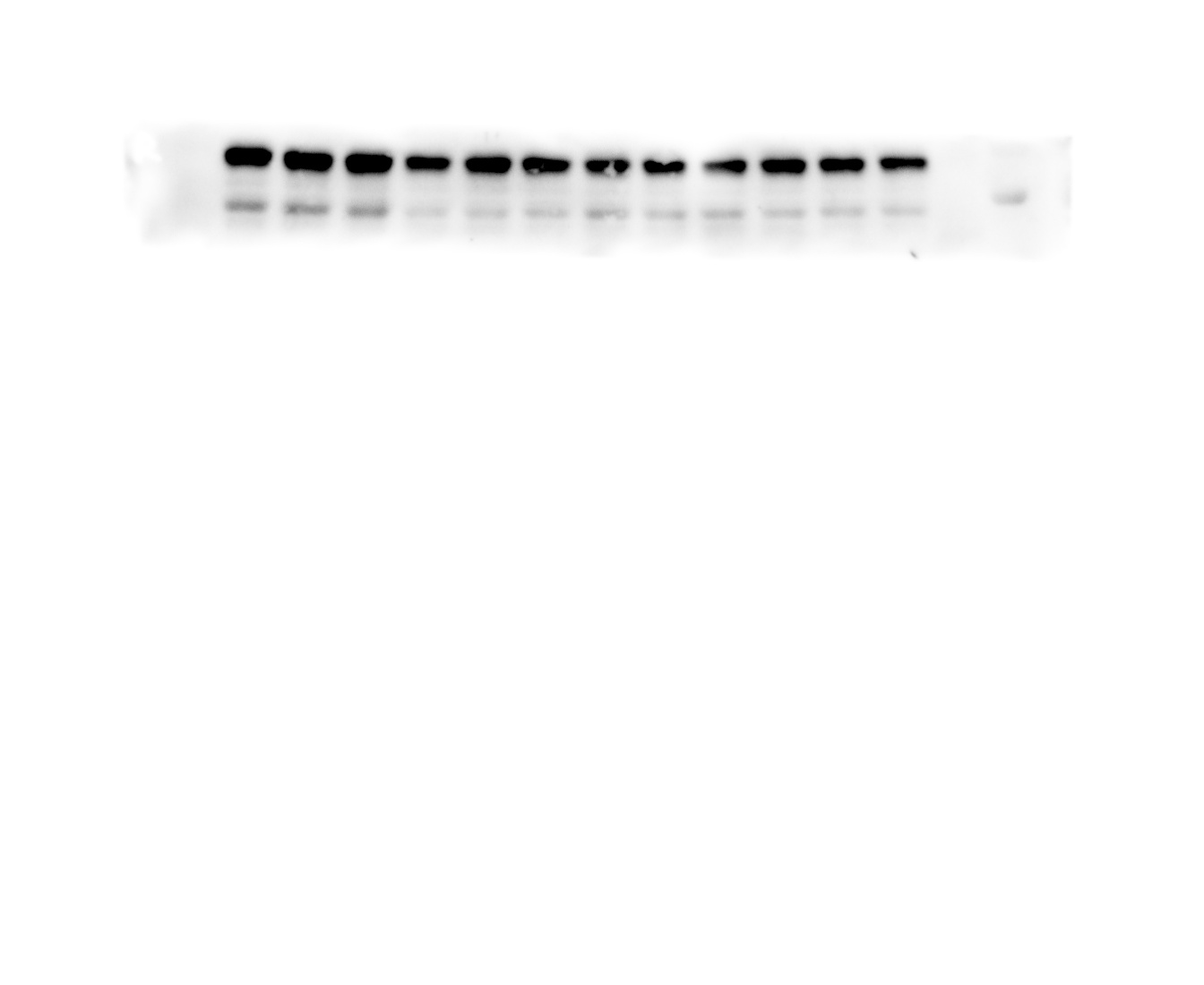

Supplement: Figure 3—source data 1. — The folders named ‘Figure 3C’, ‘Figure 3D’, ‘Figure 3E’, ‘Figure 3F’ and ‘Figure 3G’ contain the original images in Figure 3C, Figure 3D, Figure 3E, Figure 3F and Figure 3G, respectively (the individual file name containing ‘(labeled)’ is blot with the relevant bands labeled by a red outline). [file elife-64872-fig3-data1.zip › Figure 3-source data 1/Figure 3F/Runx2.jpg]

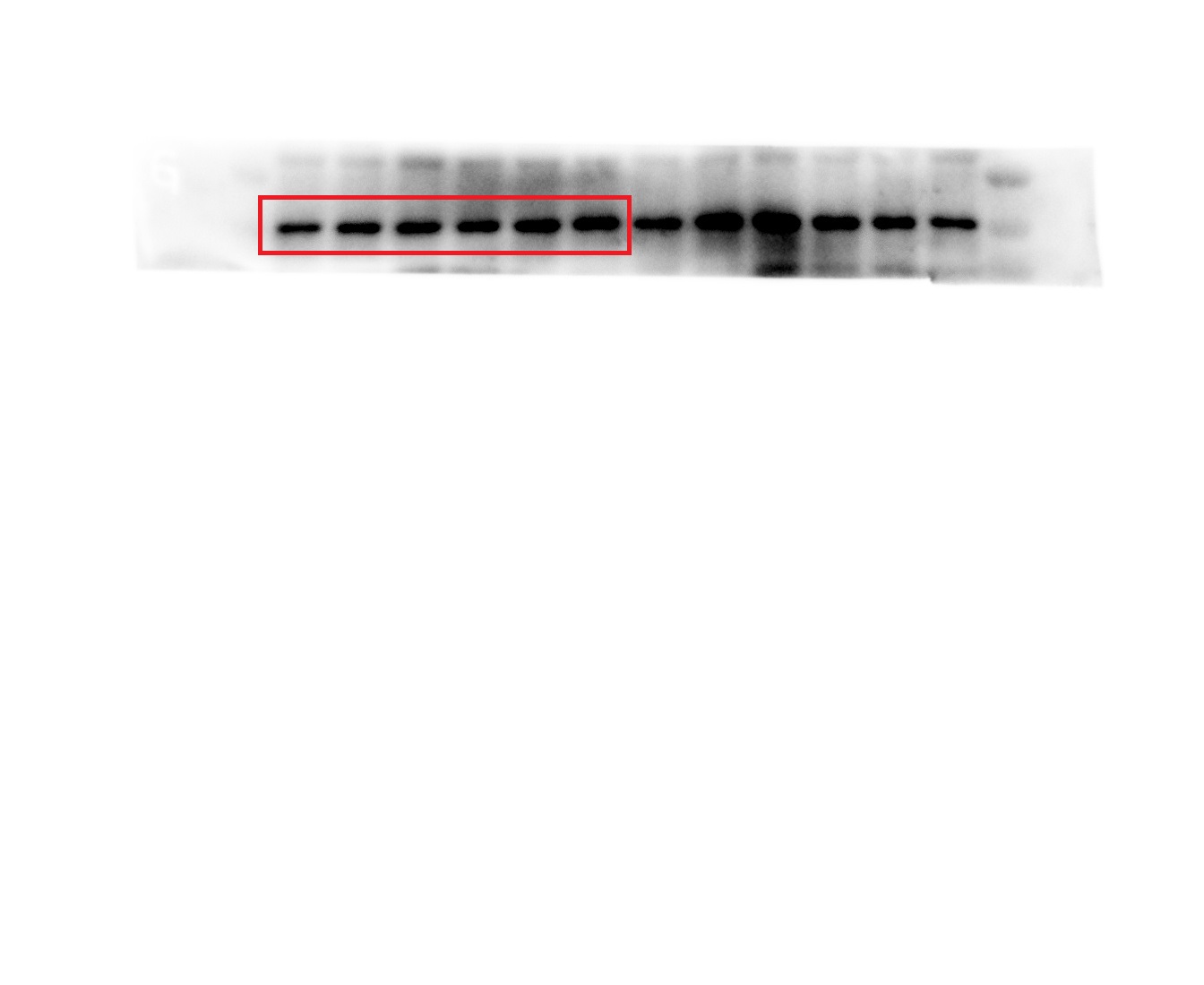

Supplement: Figure 3—source data 1. — The folders named ‘Figure 3C’, ‘Figure 3D’, ‘Figure 3E’, ‘Figure 3F’ and ‘Figure 3G’ contain the original images in Figure 3C, Figure 3D, Figure 3E, Figure 3F and Figure 3G, respectively (the individual file name containing ‘(labeled)’ is blot with the relevant bands labeled by a red outline). [file elife-64872-fig3-data1.zip › Figure 3-source data 1/Figure 3G/GAPDH (labelled).jpg]

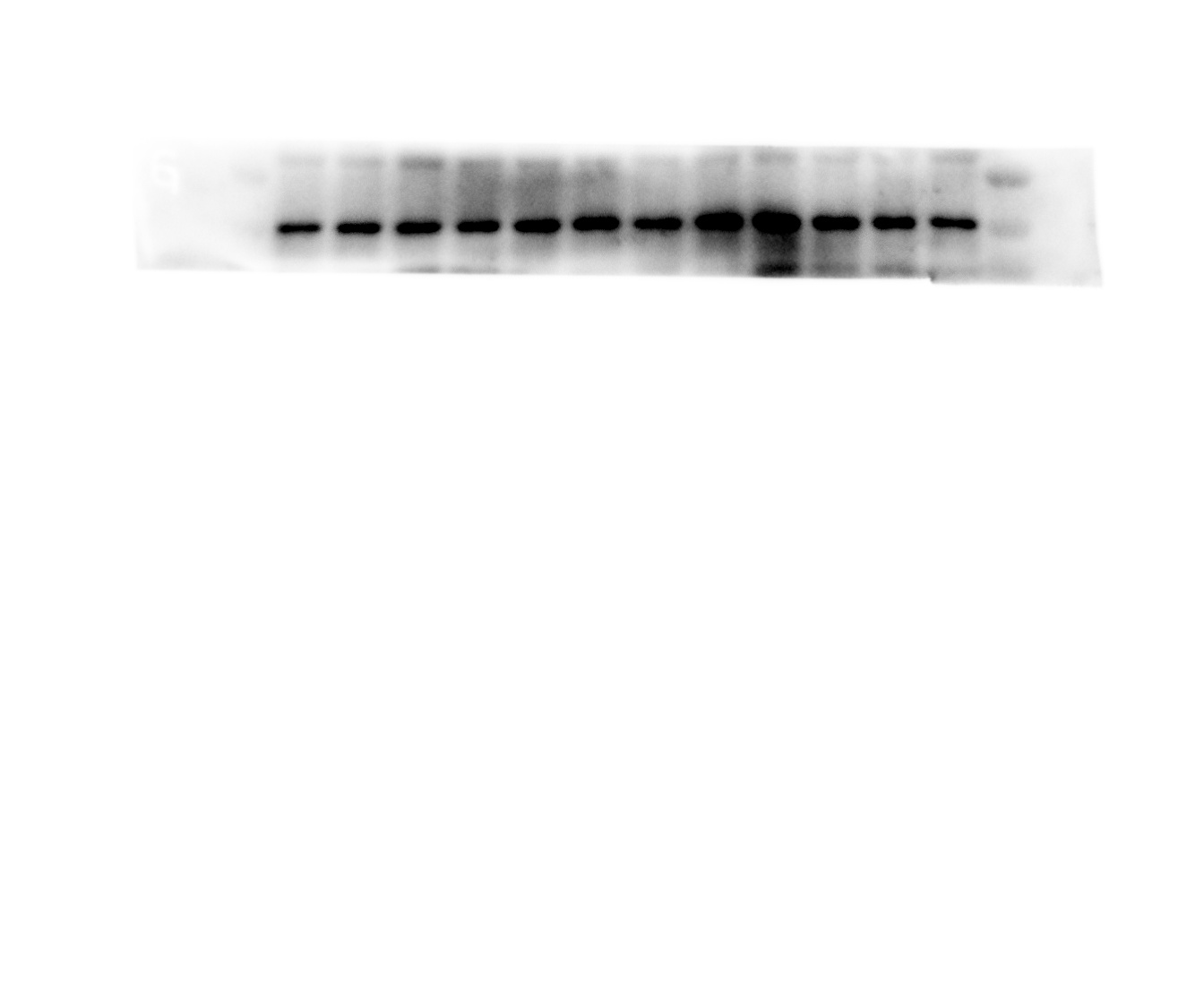

Supplement: Figure 3—source data 1. — The folders named ‘Figure 3C’, ‘Figure 3D’, ‘Figure 3E’, ‘Figure 3F’ and ‘Figure 3G’ contain the original images in Figure 3C, Figure 3D, Figure 3E, Figure 3F and Figure 3G, respectively (the individual file name containing ‘(labeled)’ is blot with the relevant bands labeled by a red outline). [file elife-64872-fig3-data1.zip › Figure 3-source data 1/Figure 3G/GAPDH.jpg]

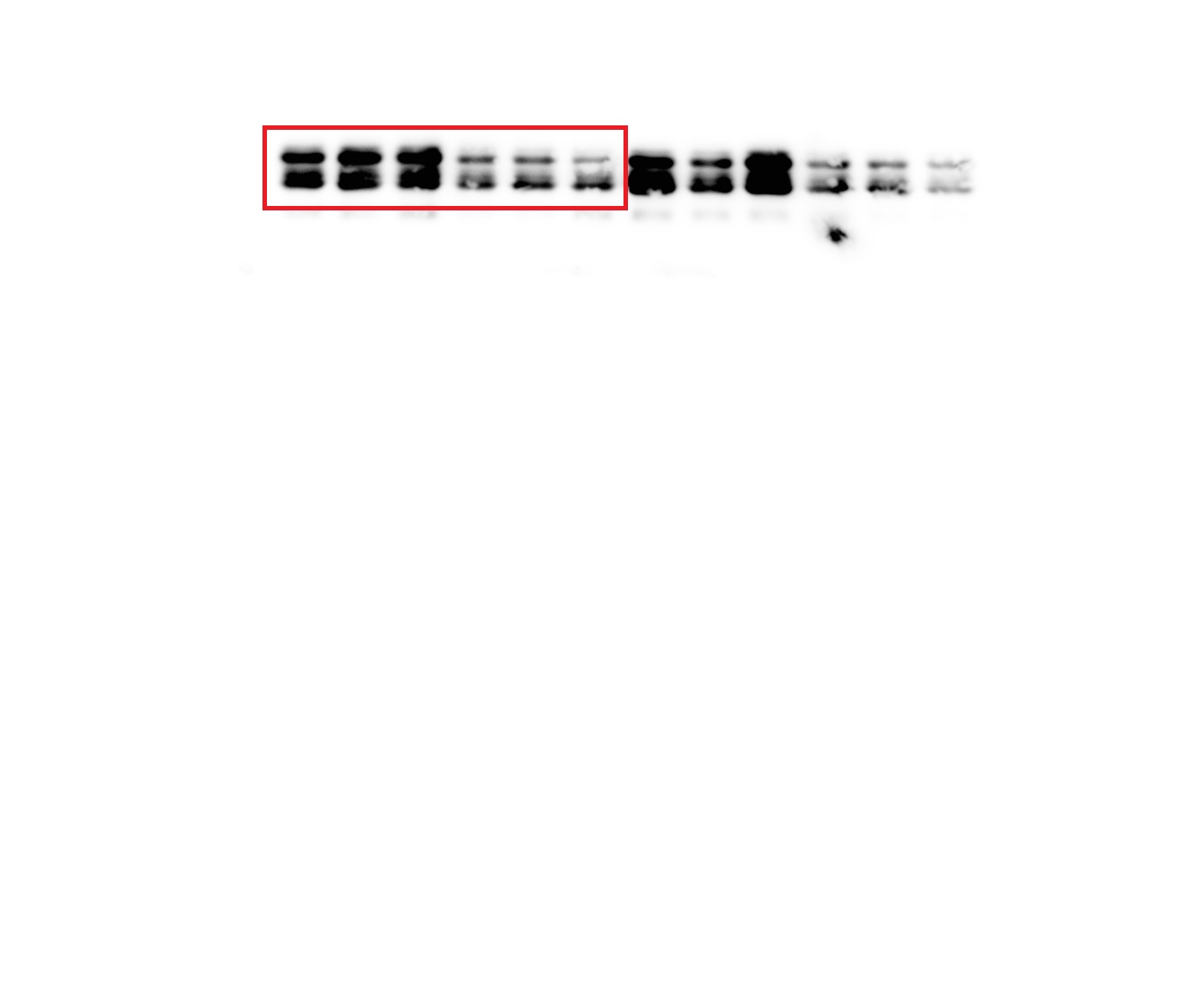

Supplement: Figure 3—source data 1. — The folders named ‘Figure 3C’, ‘Figure 3D’, ‘Figure 3E’, ‘Figure 3F’ and ‘Figure 3G’ contain the original images in Figure 3C, Figure 3D, Figure 3E, Figure 3F and Figure 3G, respectively (the individual file name containing ‘(labeled)’ is blot with the relevant bands labeled by a red outline). [file elife-64872-fig3-data1.zip › Figure 3-source data 1/Figure 3G/Osterix (labelled).jpg]

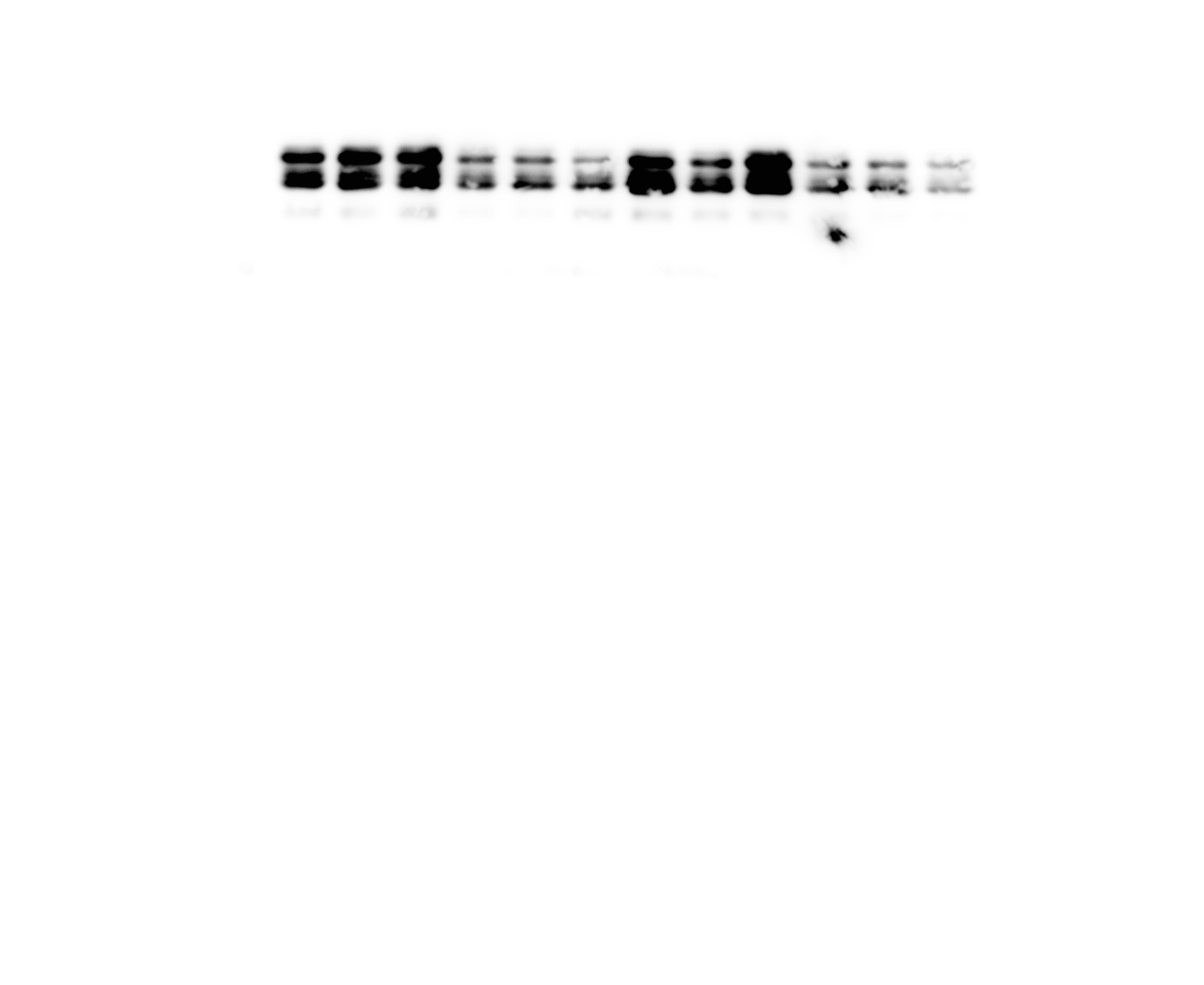

Supplement: Figure 3—source data 1. — The folders named ‘Figure 3C’, ‘Figure 3D’, ‘Figure 3E’, ‘Figure 3F’ and ‘Figure 3G’ contain the original images in Figure 3C, Figure 3D, Figure 3E, Figure 3F and Figure 3G, respectively (the individual file name containing ‘(labeled)’ is blot with the relevant bands labeled by a red outline). [file elife-64872-fig3-data1.zip › Figure 3-source data 1/Figure 3G/Osterix.jpg]

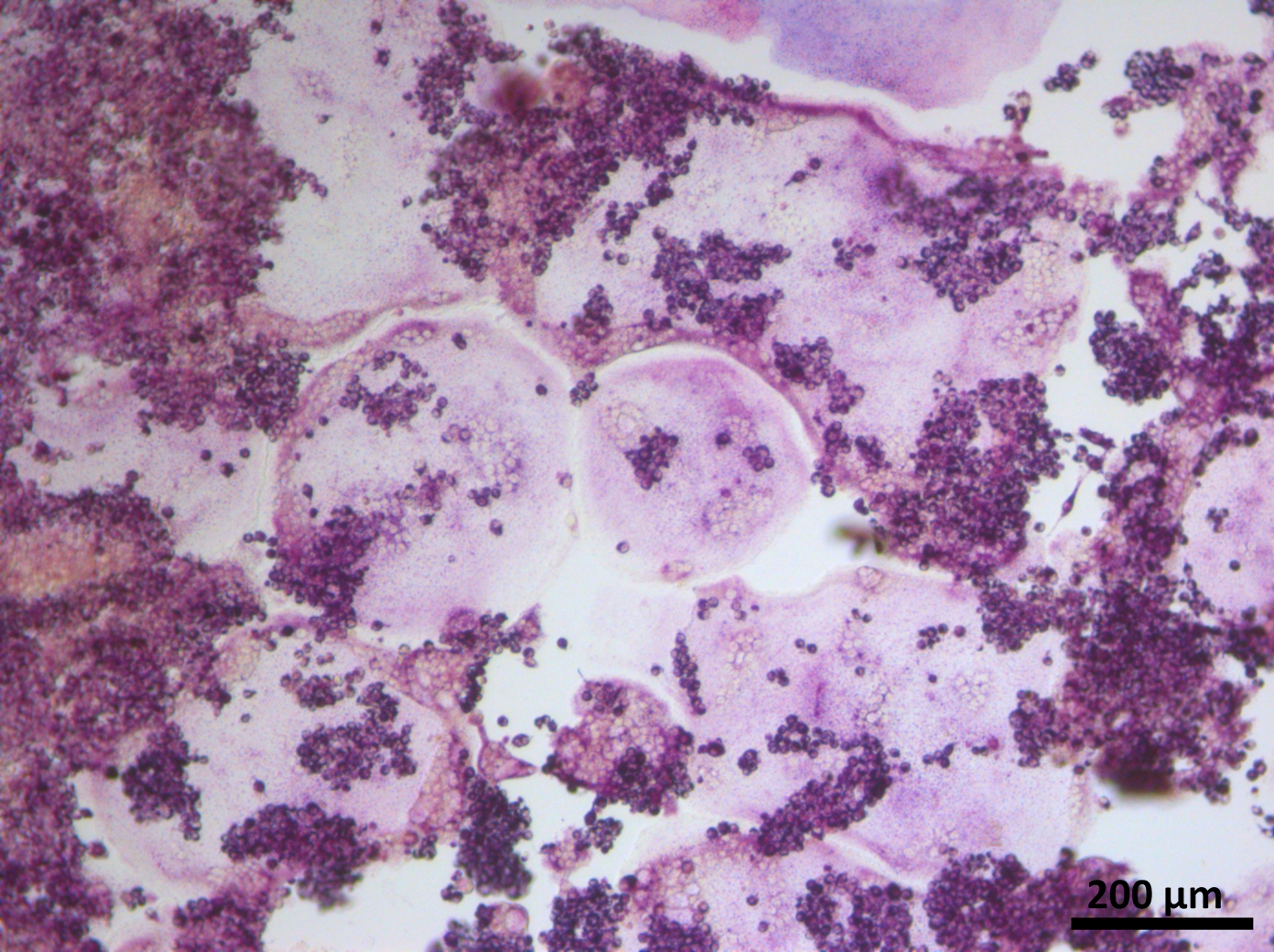

Supplement: Figure 4—source data 1. — The folder named ‘7,8-DHF’ contains micrographs of cells with or without 7,8-DHF treatment (individual files are named by the concentration of 7,8-DHF). The folder named ‘BDNF’ contains micrographs of cells with or without BDNF treatment (individual files are named by the concentration of BDNF). [file elife-64872-fig4-data1.zip › Figure 4-source data 1/7,8-DHF/0 (1).jpg]

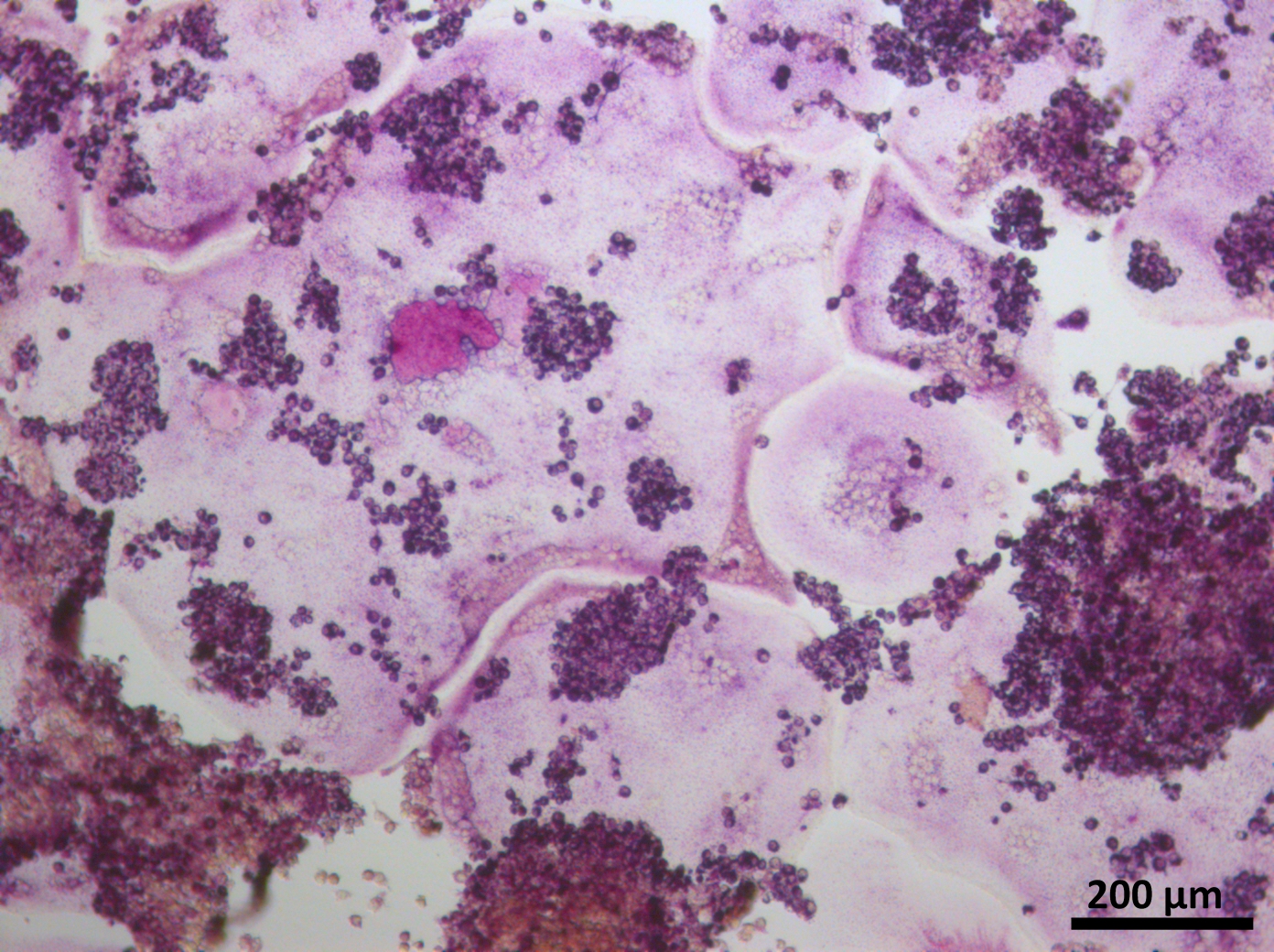

Supplement: Figure 4—source data 1. — The folder named ‘7,8-DHF’ contains micrographs of cells with or without 7,8-DHF treatment (individual files are named by the concentration of 7,8-DHF). The folder named ‘BDNF’ contains micrographs of cells with or without BDNF treatment (individual files are named by the concentration of BDNF). [file elife-64872-fig4-data1.zip › Figure 4-source data 1/7,8-DHF/0 (2).jpg]

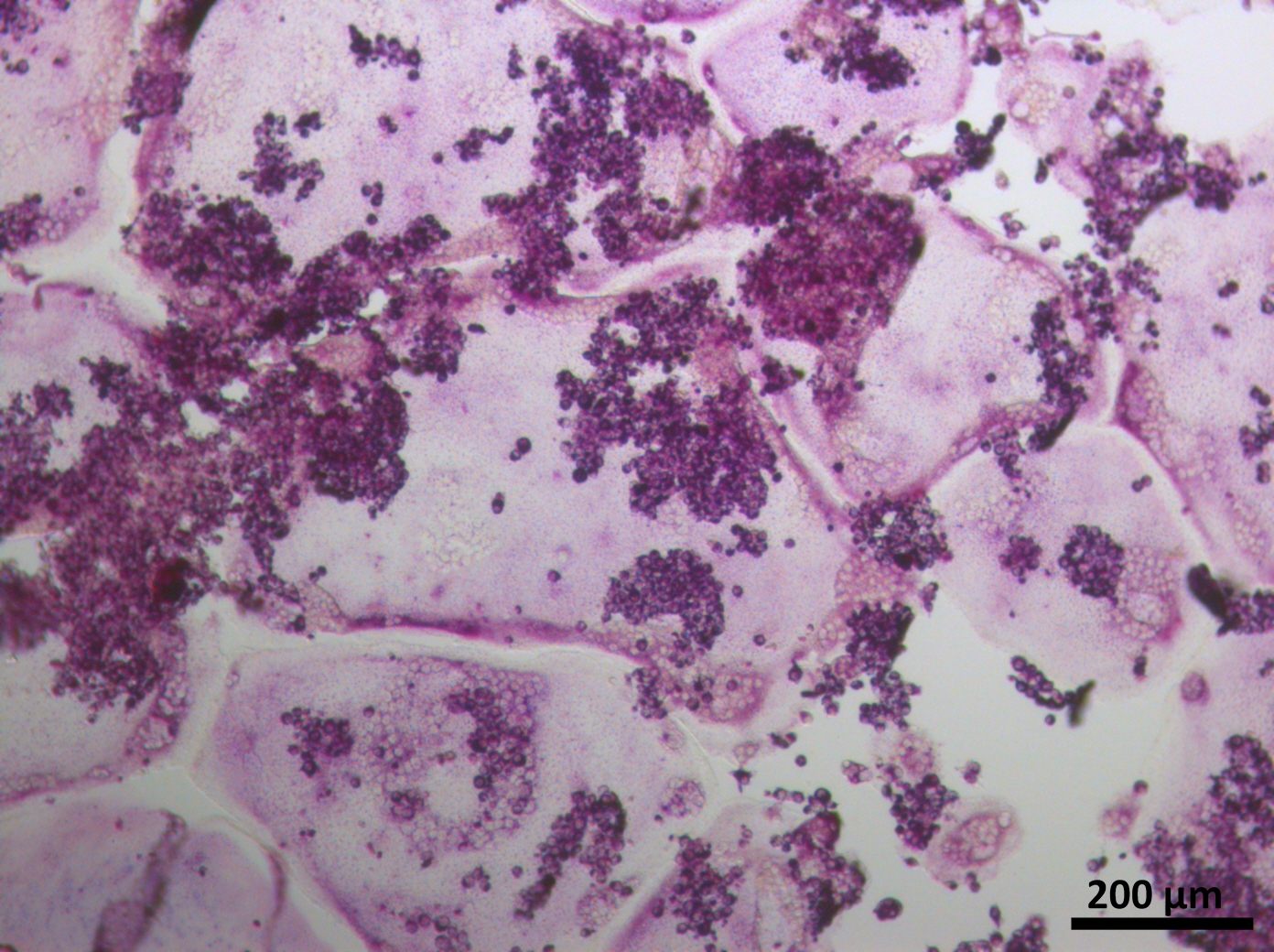

Supplement: Figure 4—source data 1. — The folder named ‘7,8-DHF’ contains micrographs of cells with or without 7,8-DHF treatment (individual files are named by the concentration of 7,8-DHF). The folder named ‘BDNF’ contains micrographs of cells with or without BDNF treatment (individual files are named by the concentration of BDNF). [file elife-64872-fig4-data1.zip › Figure 4-source data 1/7,8-DHF/0 (3).jpg]

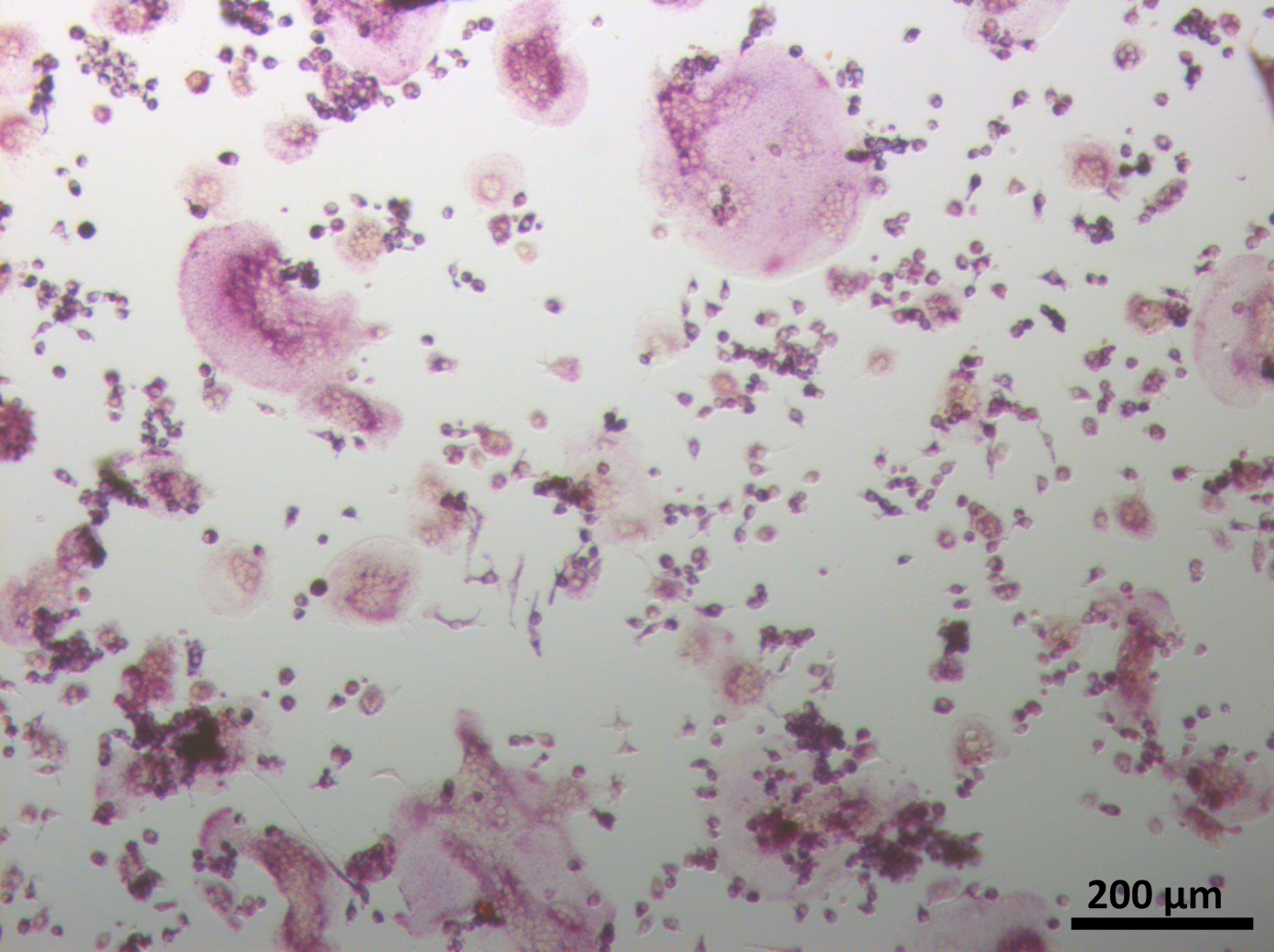

Supplement: Figure 4—source data 1. — The folder named ‘7,8-DHF’ contains micrographs of cells with or without 7,8-DHF treatment (individual files are named by the concentration of 7,8-DHF). The folder named ‘BDNF’ contains micrographs of cells with or without BDNF treatment (individual files are named by the concentration of BDNF). [file elife-64872-fig4-data1.zip › Figure 4-source data 1/7,8-DHF/0.5 μM (2).jpg]

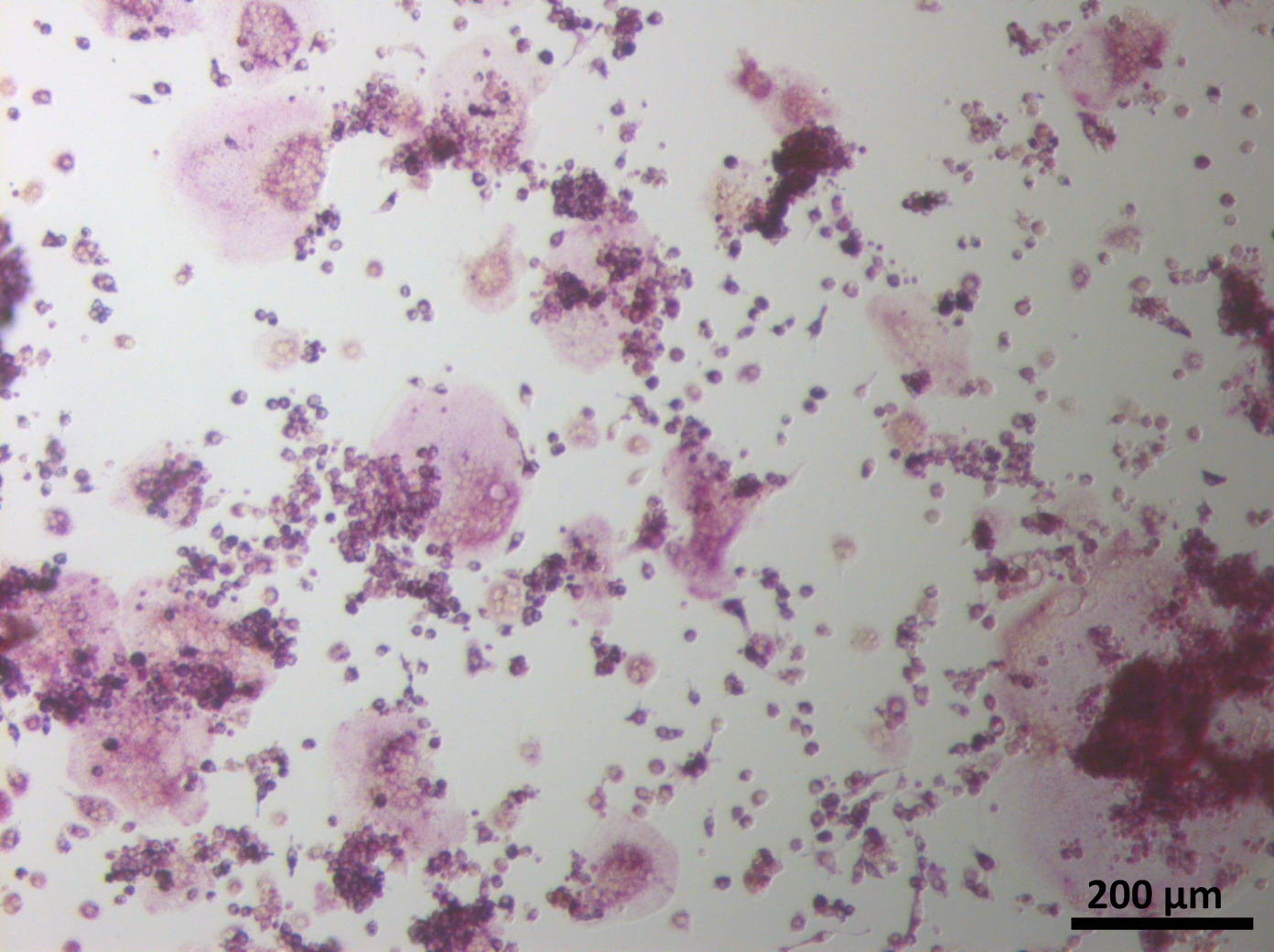

Supplement: Figure 4—source data 1. — The folder named ‘7,8-DHF’ contains micrographs of cells with or without 7,8-DHF treatment (individual files are named by the concentration of 7,8-DHF). The folder named ‘BDNF’ contains micrographs of cells with or without BDNF treatment (individual files are named by the concentration of BDNF). [file elife-64872-fig4-data1.zip › Figure 4-source data 1/7,8-DHF/0.5 μM (3).jpg]

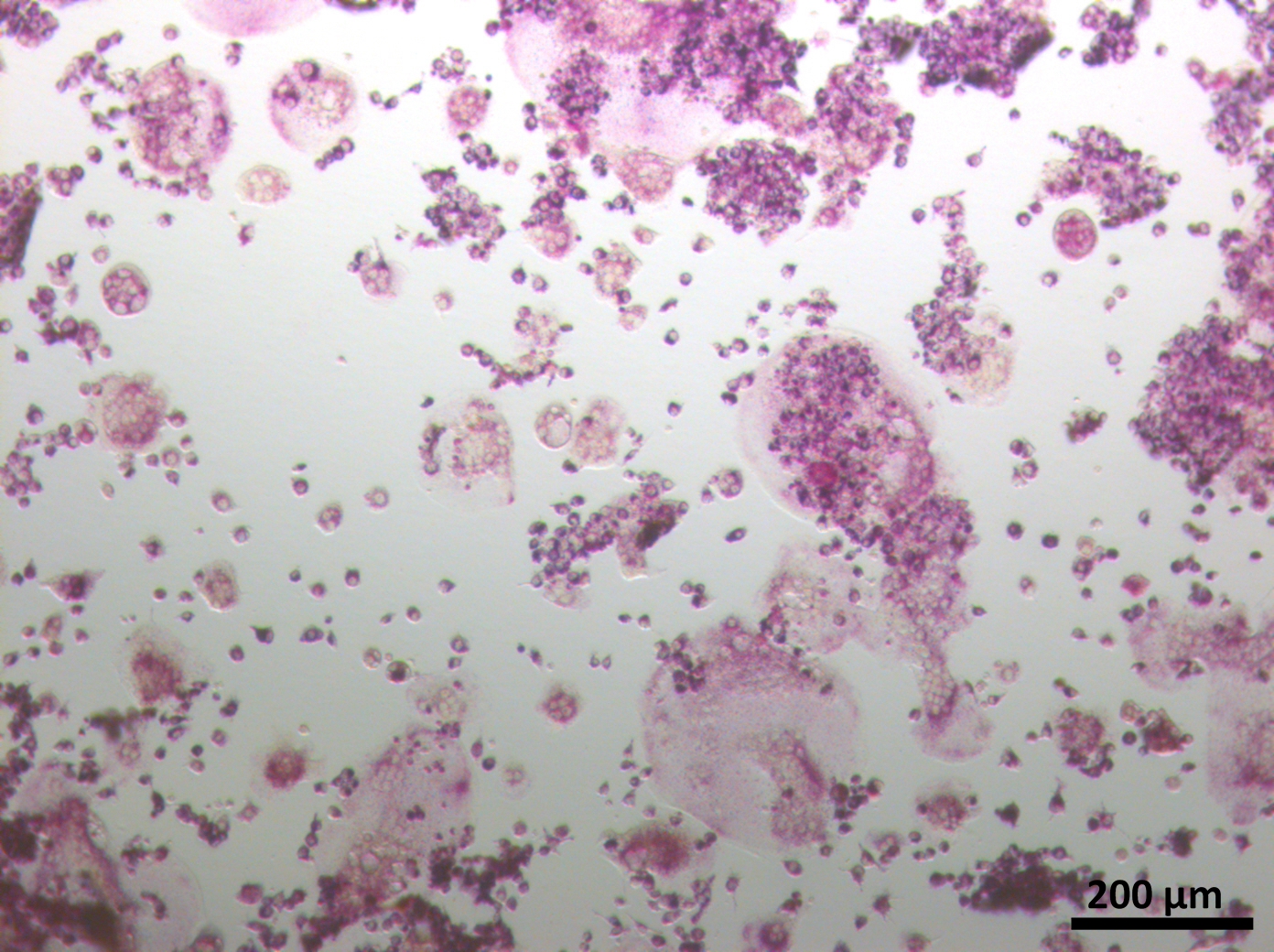

Supplement: Figure 4—source data 1. — The folder named ‘7,8-DHF’ contains micrographs of cells with or without 7,8-DHF treatment (individual files are named by the concentration of 7,8-DHF). The folder named ‘BDNF’ contains micrographs of cells with or without BDNF treatment (individual files are named by the concentration of BDNF). [file elife-64872-fig4-data1.zip › Figure 4-source data 1/7,8-DHF/0.5 μM (1).jpg]

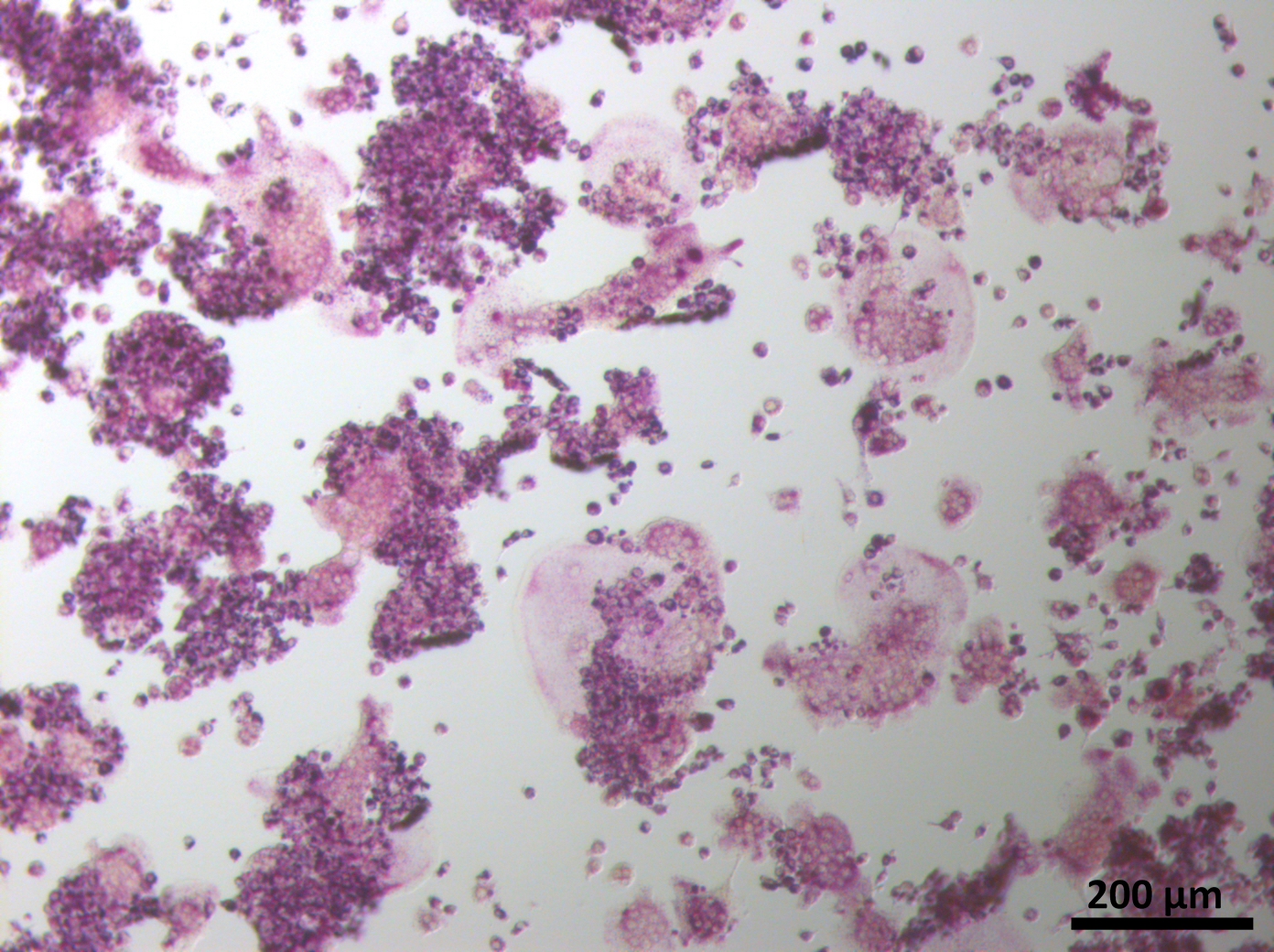

Supplement: Figure 4—source data 1. — The folder named ‘7,8-DHF’ contains micrographs of cells with or without 7,8-DHF treatment (individual files are named by the concentration of 7,8-DHF). The folder named ‘BDNF’ contains micrographs of cells with or without BDNF treatment (individual files are named by the concentration of BDNF). [file elife-64872-fig4-data1.zip › Figure 4-source data 1/7,8-DHF/1 μM (1).jpg]

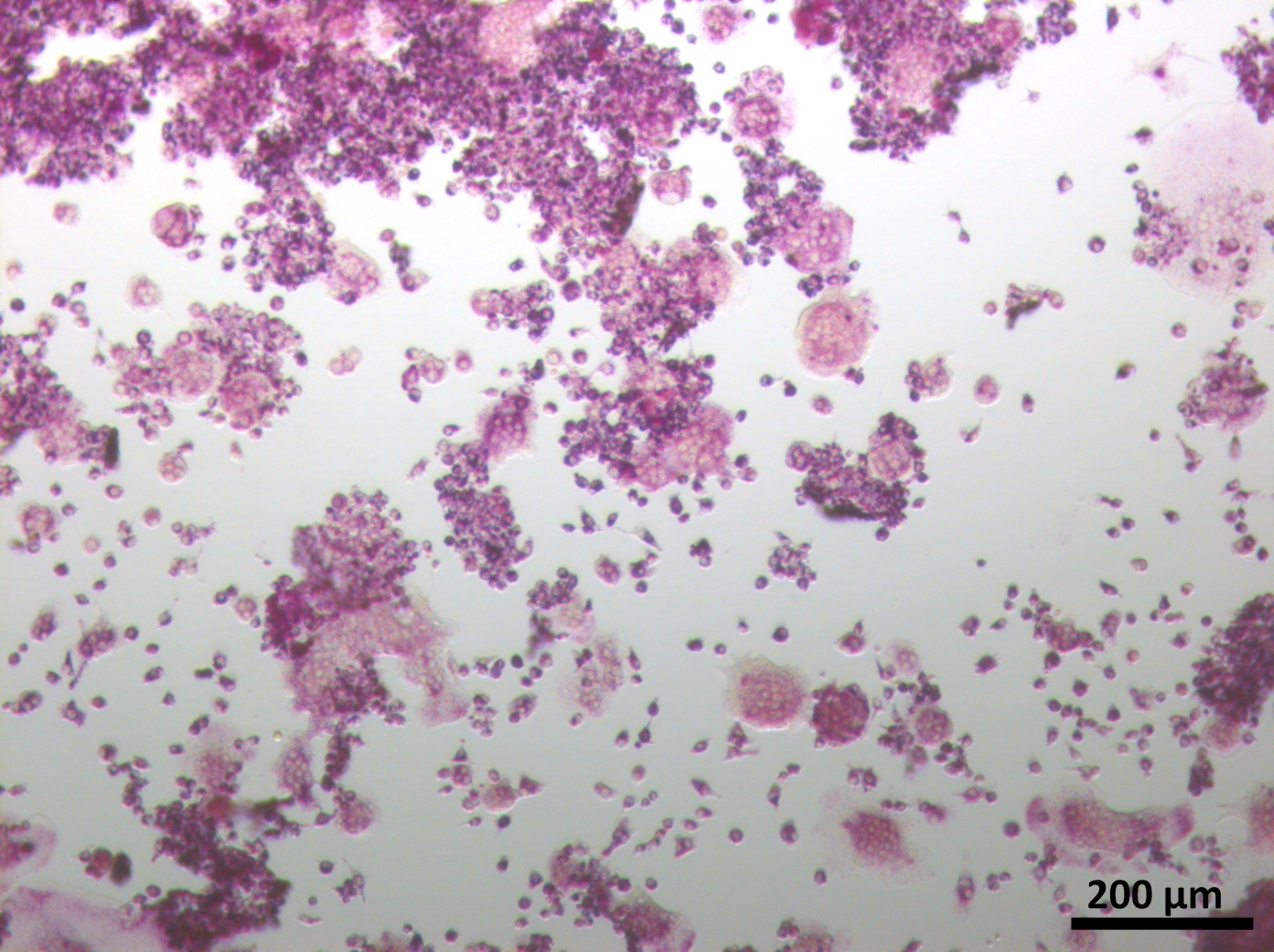

Supplement: Figure 4—source data 1. — The folder named ‘7,8-DHF’ contains micrographs of cells with or without 7,8-DHF treatment (individual files are named by the concentration of 7,8-DHF). The folder named ‘BDNF’ contains micrographs of cells with or without BDNF treatment (individual files are named by the concentration of BDNF). [file elife-64872-fig4-data1.zip › Figure 4-source data 1/7,8-DHF/1 μM (2).jpg]

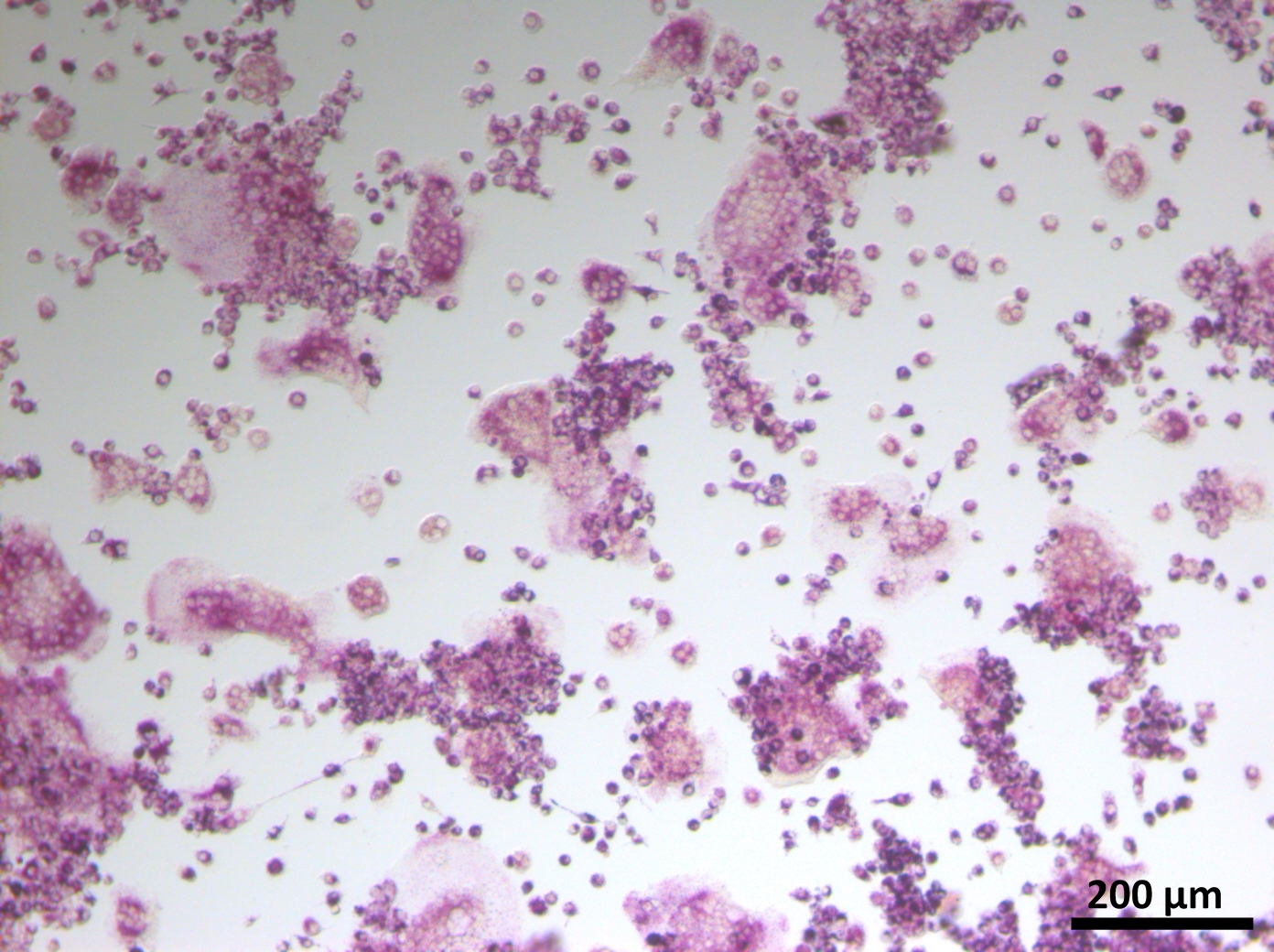

Supplement: Figure 4—source data 1. — The folder named ‘7,8-DHF’ contains micrographs of cells with or without 7,8-DHF treatment (individual files are named by the concentration of 7,8-DHF). The folder named ‘BDNF’ contains micrographs of cells with or without BDNF treatment (individual files are named by the concentration of BDNF). [file elife-64872-fig4-data1.zip › Figure 4-source data 1/7,8-DHF/1 μM (3).jpg]

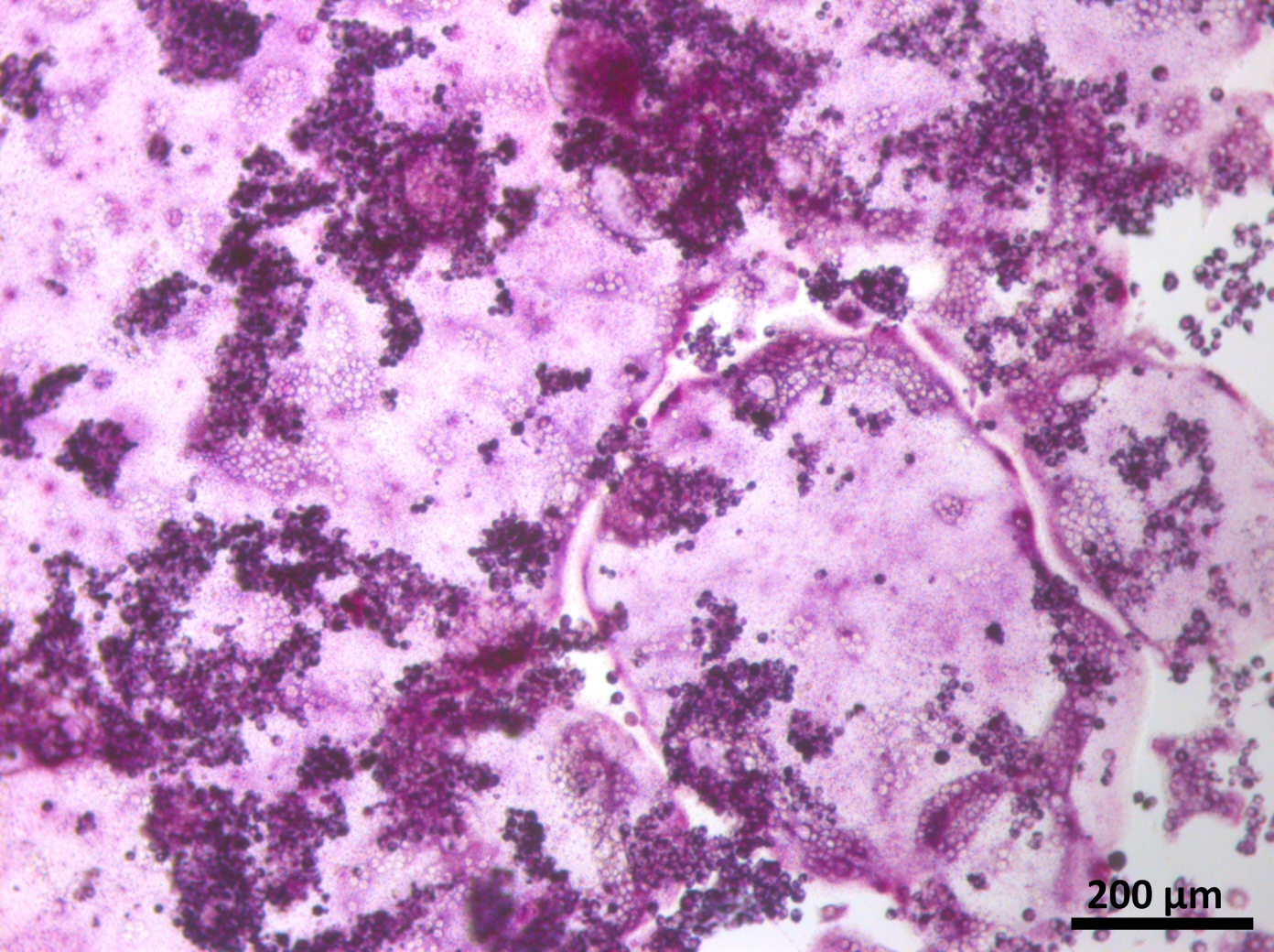

Supplement: Figure 4—source data 1. — The folder named ‘7,8-DHF’ contains micrographs of cells with or without 7,8-DHF treatment (individual files are named by the concentration of 7,8-DHF). The folder named ‘BDNF’ contains micrographs of cells with or without BDNF treatment (individual files are named by the concentration of BDNF). [file elife-64872-fig4-data1.zip › Figure 4-source data 1/7,8-DHF/5 μM (1).jpg]

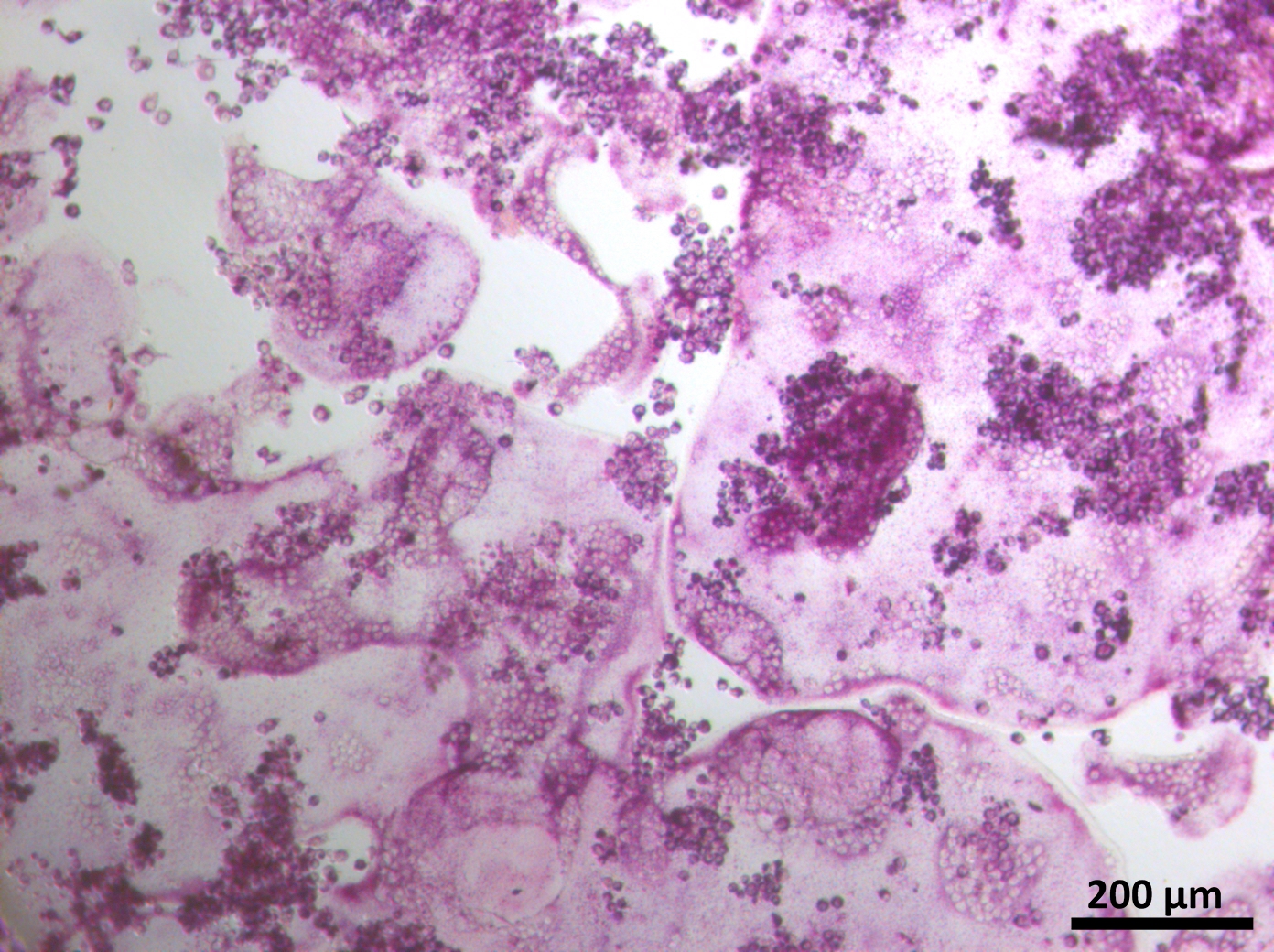

Supplement: Figure 4—source data 1. — The folder named ‘7,8-DHF’ contains micrographs of cells with or without 7,8-DHF treatment (individual files are named by the concentration of 7,8-DHF). The folder named ‘BDNF’ contains micrographs of cells with or without BDNF treatment (individual files are named by the concentration of BDNF). [file elife-64872-fig4-data1.zip › Figure 4-source data 1/7,8-DHF/5 μM (2).jpg]

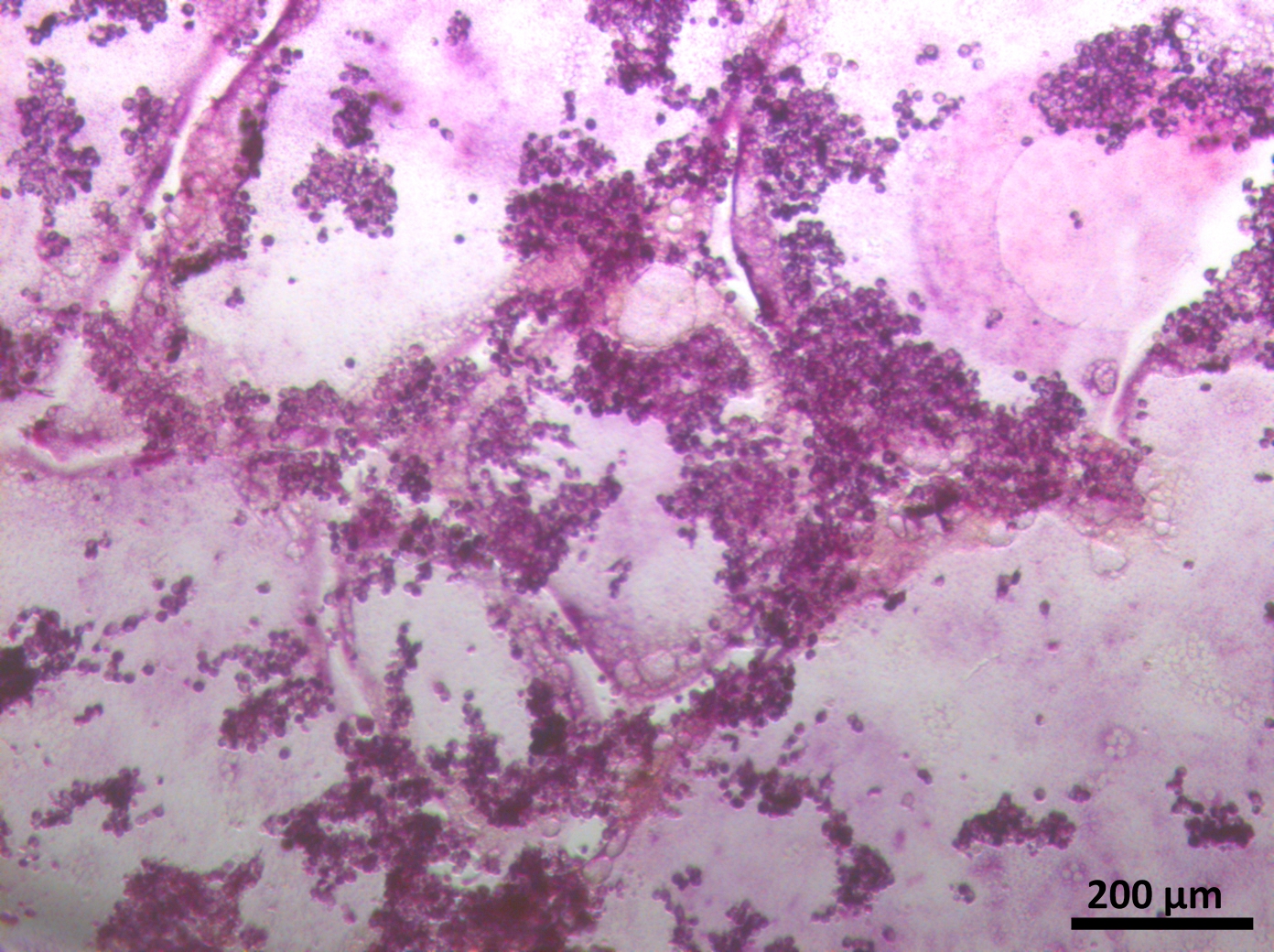

Supplement: Figure 4—source data 1. — The folder named ‘7,8-DHF’ contains micrographs of cells with or without 7,8-DHF treatment (individual files are named by the concentration of 7,8-DHF). The folder named ‘BDNF’ contains micrographs of cells with or without BDNF treatment (individual files are named by the concentration of BDNF). [file elife-64872-fig4-data1.zip › Figure 4-source data 1/7,8-DHF/5 μM (3).jpg]

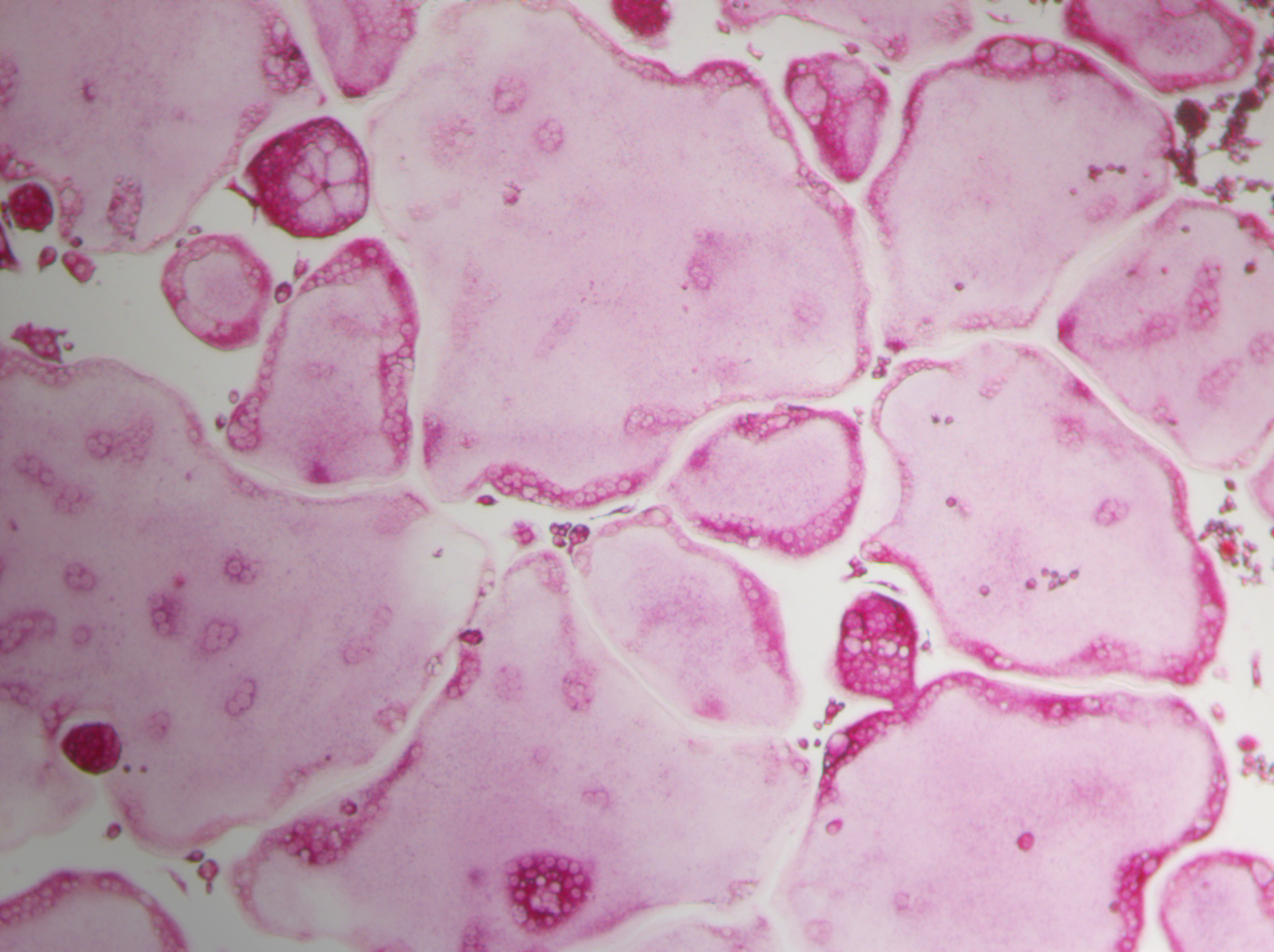

Supplement: Figure 4—source data 1. — The folder named ‘7,8-DHF’ contains micrographs of cells with or without 7,8-DHF treatment (individual files are named by the concentration of 7,8-DHF). The folder named ‘BDNF’ contains micrographs of cells with or without BDNF treatment (individual files are named by the concentration of BDNF). [file elife-64872-fig4-data1.zip › Figure 4-source data 1/BDNF/0 (1).jpg]

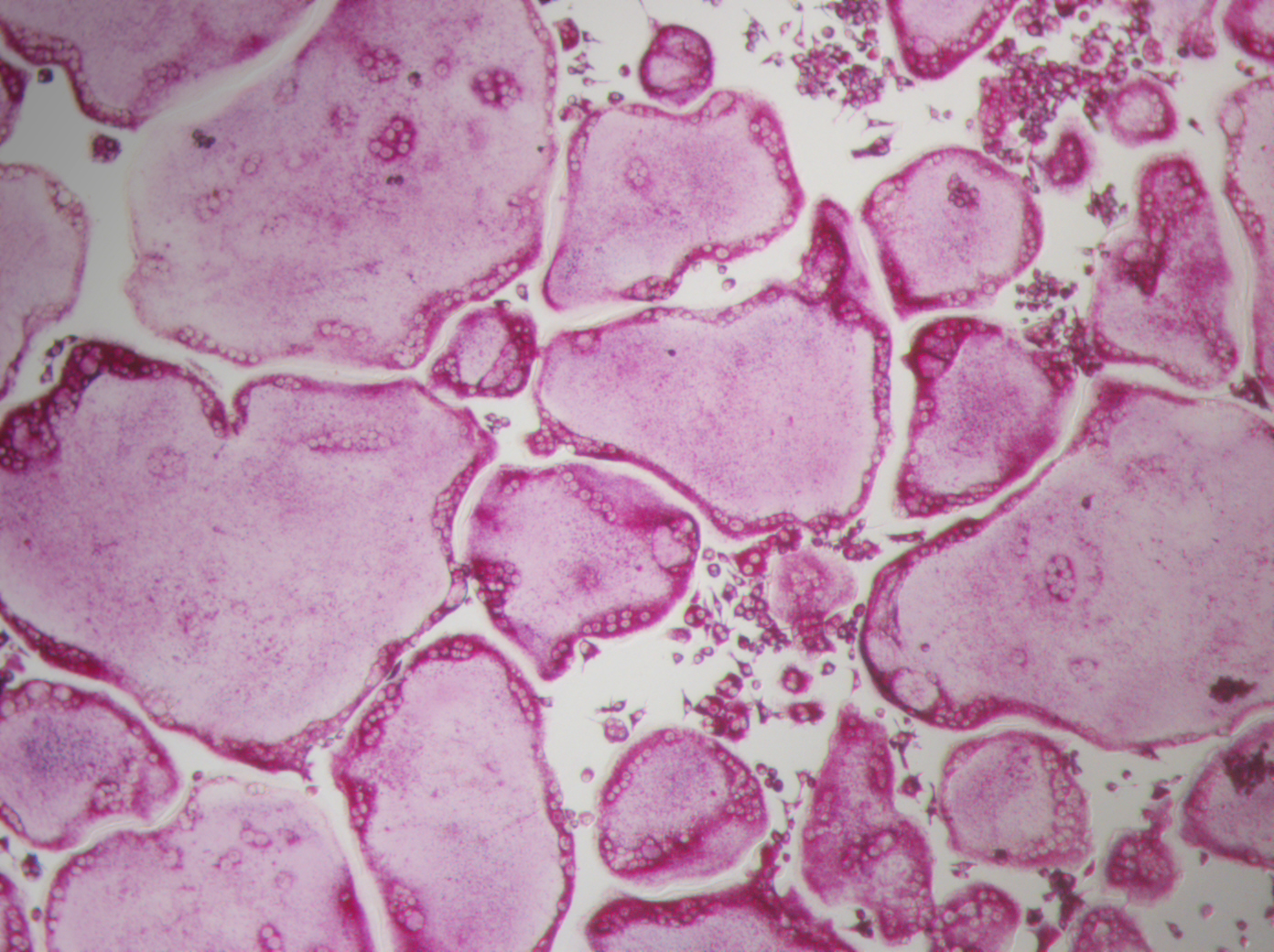

Supplement: Figure 4—source data 1. — The folder named ‘7,8-DHF’ contains micrographs of cells with or without 7,8-DHF treatment (individual files are named by the concentration of 7,8-DHF). The folder named ‘BDNF’ contains micrographs of cells with or without BDNF treatment (individual files are named by the concentration of BDNF). [file elife-64872-fig4-data1.zip › Figure 4-source data 1/BDNF/0 (2).jpg]

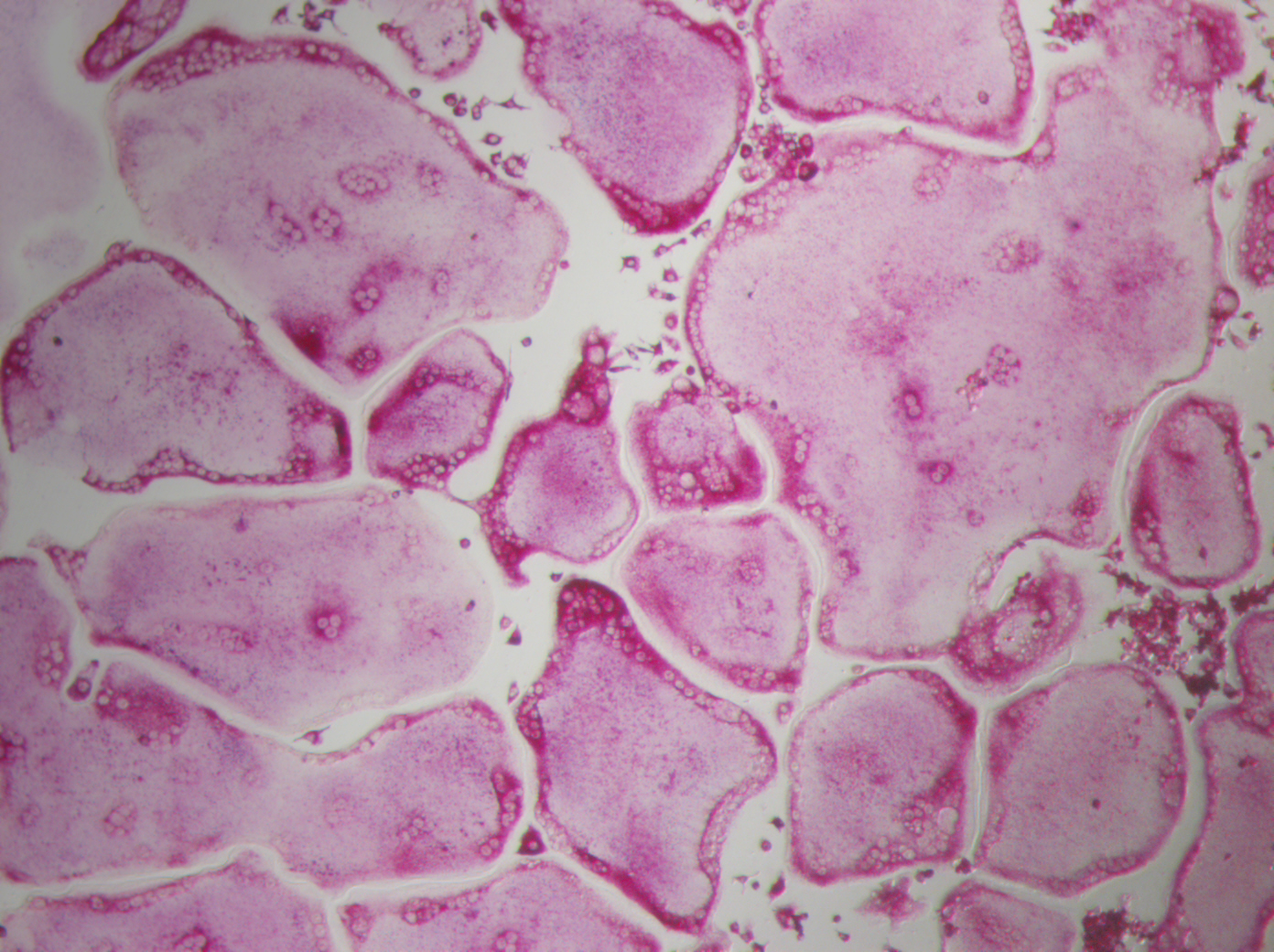

Supplement: Figure 4—source data 1. — The folder named ‘7,8-DHF’ contains micrographs of cells with or without 7,8-DHF treatment (individual files are named by the concentration of 7,8-DHF). The folder named ‘BDNF’ contains micrographs of cells with or without BDNF treatment (individual files are named by the concentration of BDNF). [file elife-64872-fig4-data1.zip › Figure 4-source data 1/BDNF/0 (3).jpg]

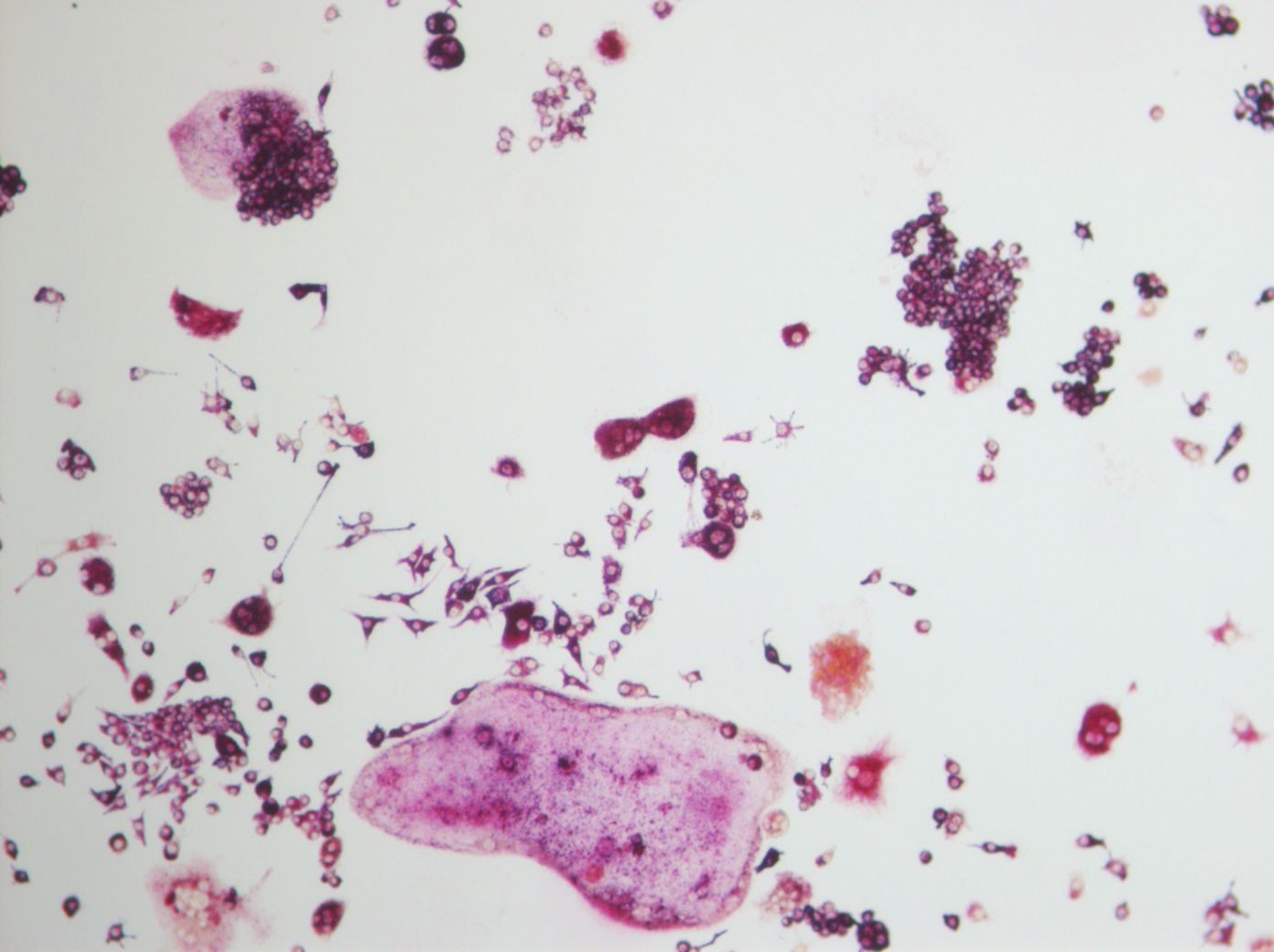

Supplement: Figure 4—source data 1. — The folder named ‘7,8-DHF’ contains micrographs of cells with or without 7,8-DHF treatment (individual files are named by the concentration of 7,8-DHF). The folder named ‘BDNF’ contains micrographs of cells with or without BDNF treatment (individual files are named by the concentration of BDNF). [file elife-64872-fig4-data1.zip › Figure 4-source data 1/BDNF/100 ng·mL-1 (1).jpg]

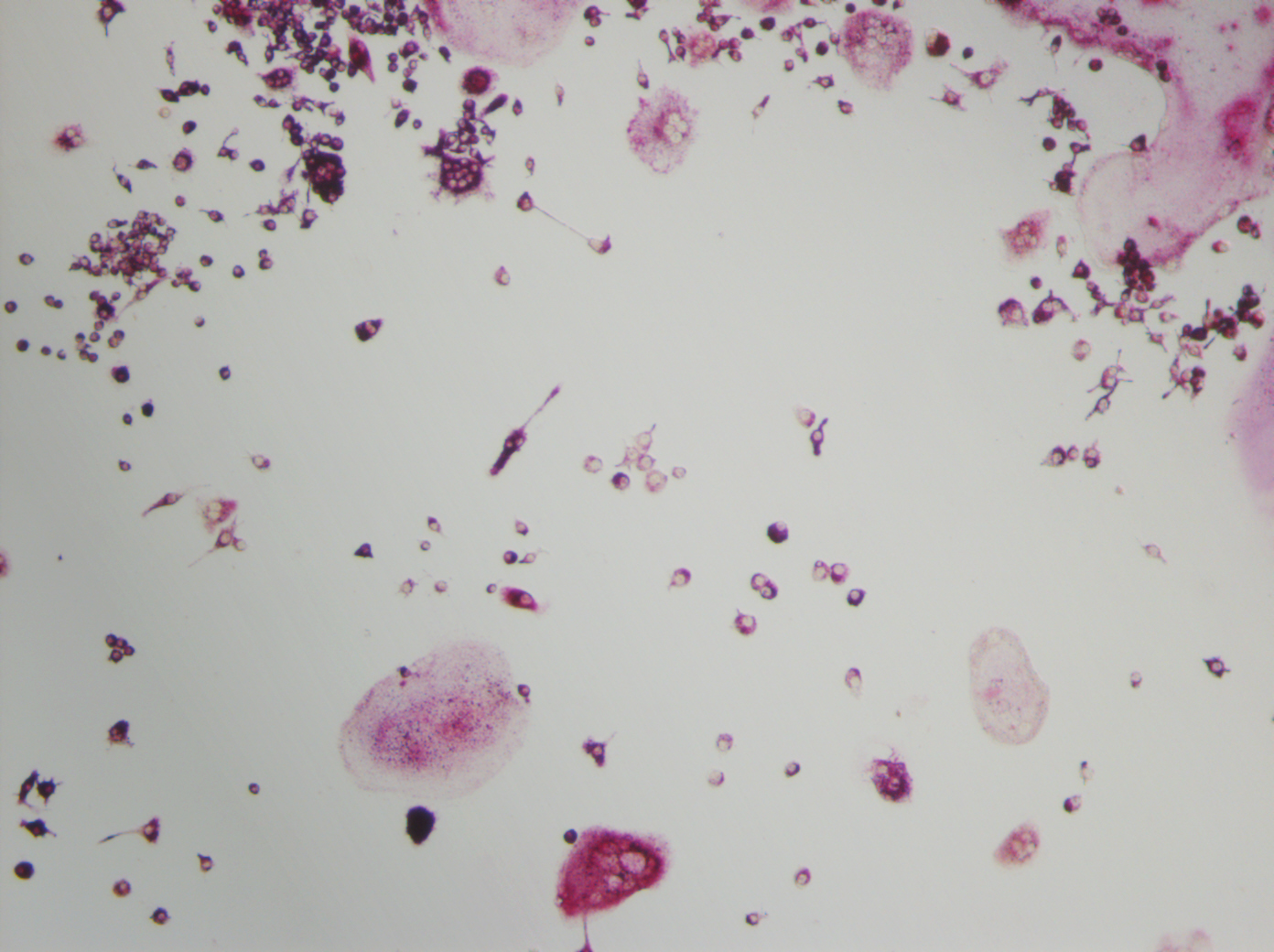

Supplement: Figure 4—source data 1. — The folder named ‘7,8-DHF’ contains micrographs of cells with or without 7,8-DHF treatment (individual files are named by the concentration of 7,8-DHF). The folder named ‘BDNF’ contains micrographs of cells with or without BDNF treatment (individual files are named by the concentration of BDNF). [file elife-64872-fig4-data1.zip › Figure 4-source data 1/BDNF/100 ng·mL-1 (2).jpg]

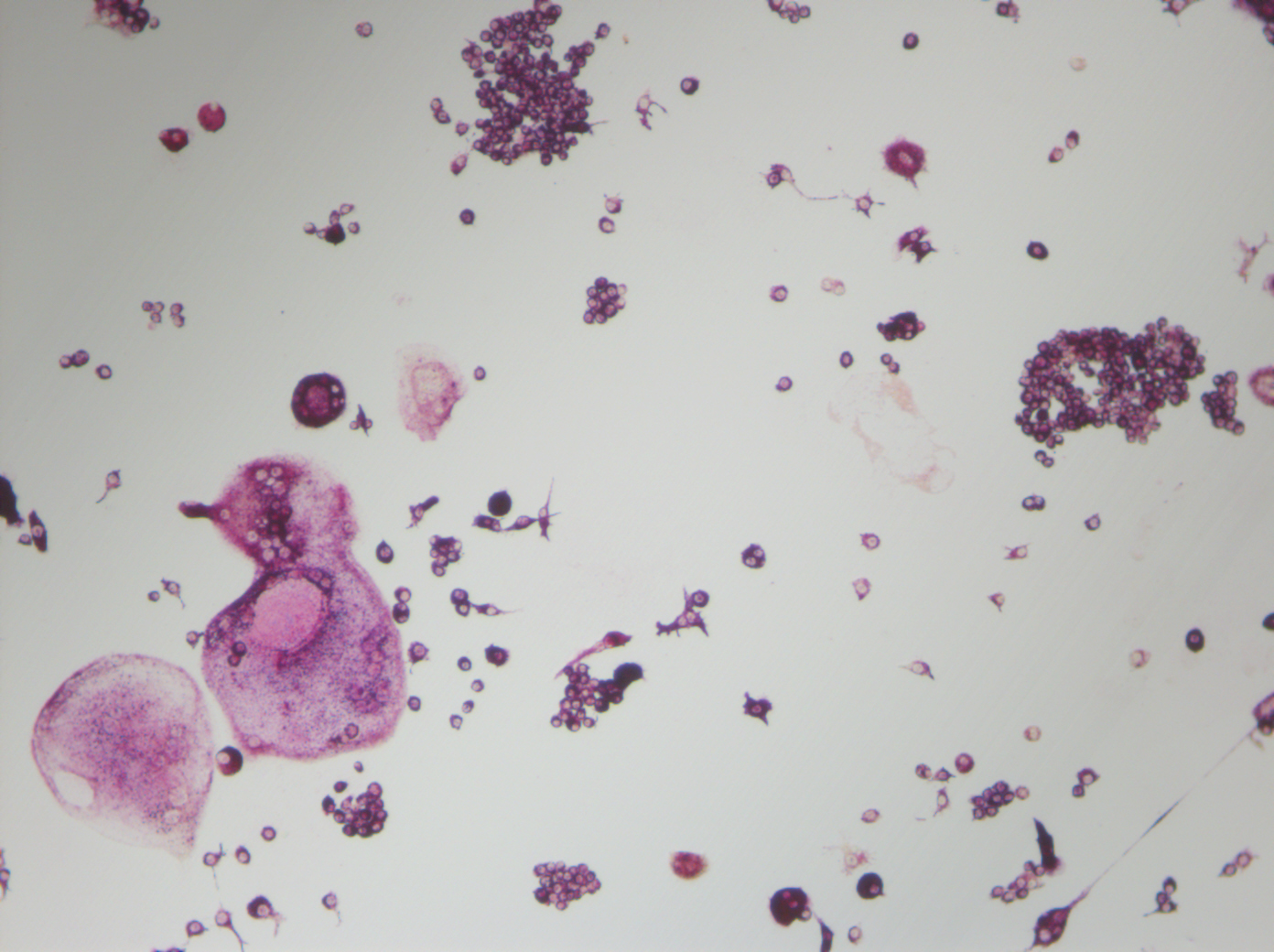

Supplement: Figure 4—source data 1. — The folder named ‘7,8-DHF’ contains micrographs of cells with or without 7,8-DHF treatment (individual files are named by the concentration of 7,8-DHF). The folder named ‘BDNF’ contains micrographs of cells with or without BDNF treatment (individual files are named by the concentration of BDNF). [file elife-64872-fig4-data1.zip › Figure 4-source data 1/BDNF/100 ng·mL-1 (3).jpg]

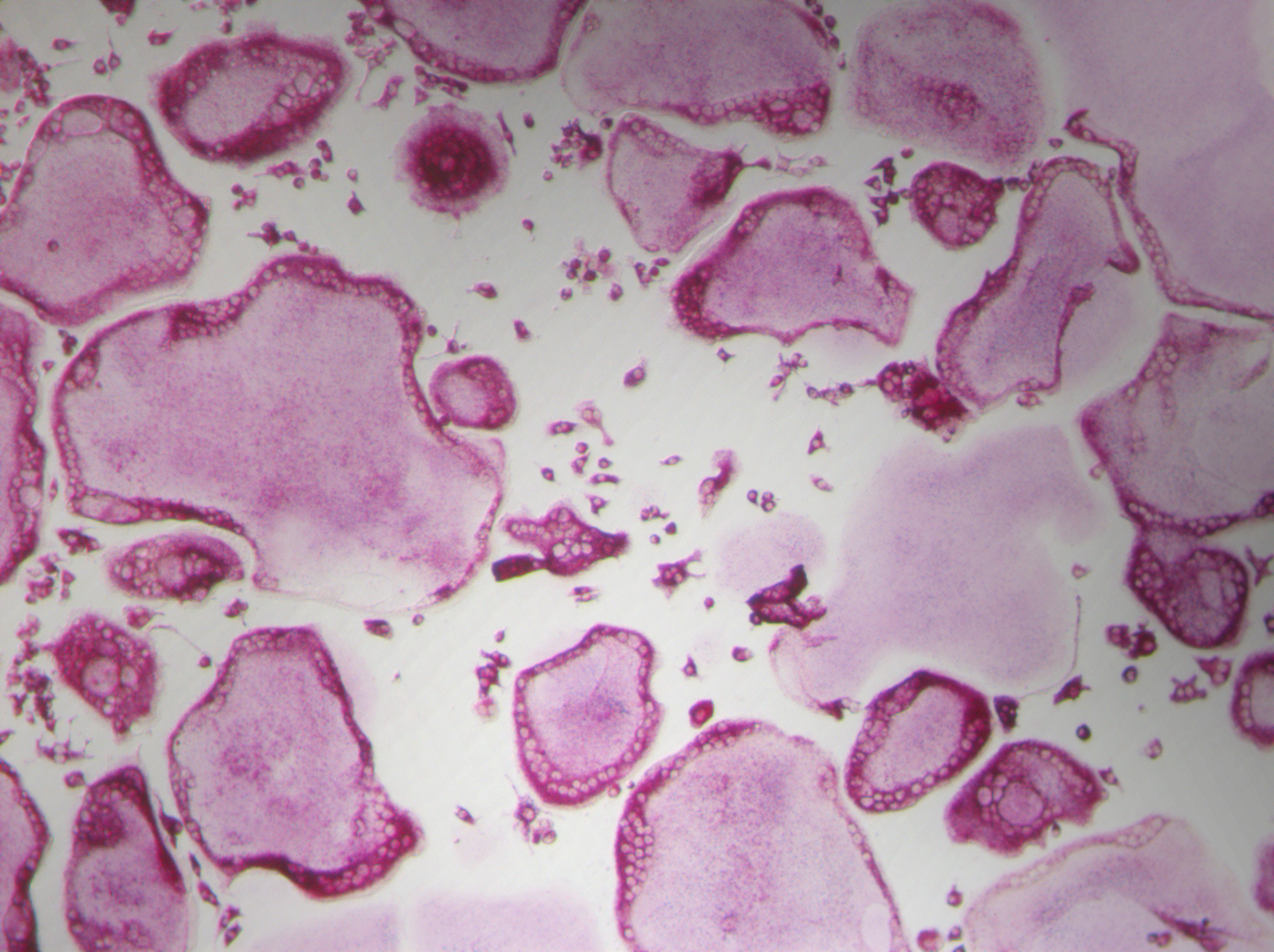

Supplement: Figure 4—source data 1. — The folder named ‘7,8-DHF’ contains micrographs of cells with or without 7,8-DHF treatment (individual files are named by the concentration of 7,8-DHF). The folder named ‘BDNF’ contains micrographs of cells with or without BDNF treatment (individual files are named by the concentration of BDNF). [file elife-64872-fig4-data1.zip › Figure 4-source data 1/BDNF/25 ng·mL-1 (1).jpg]

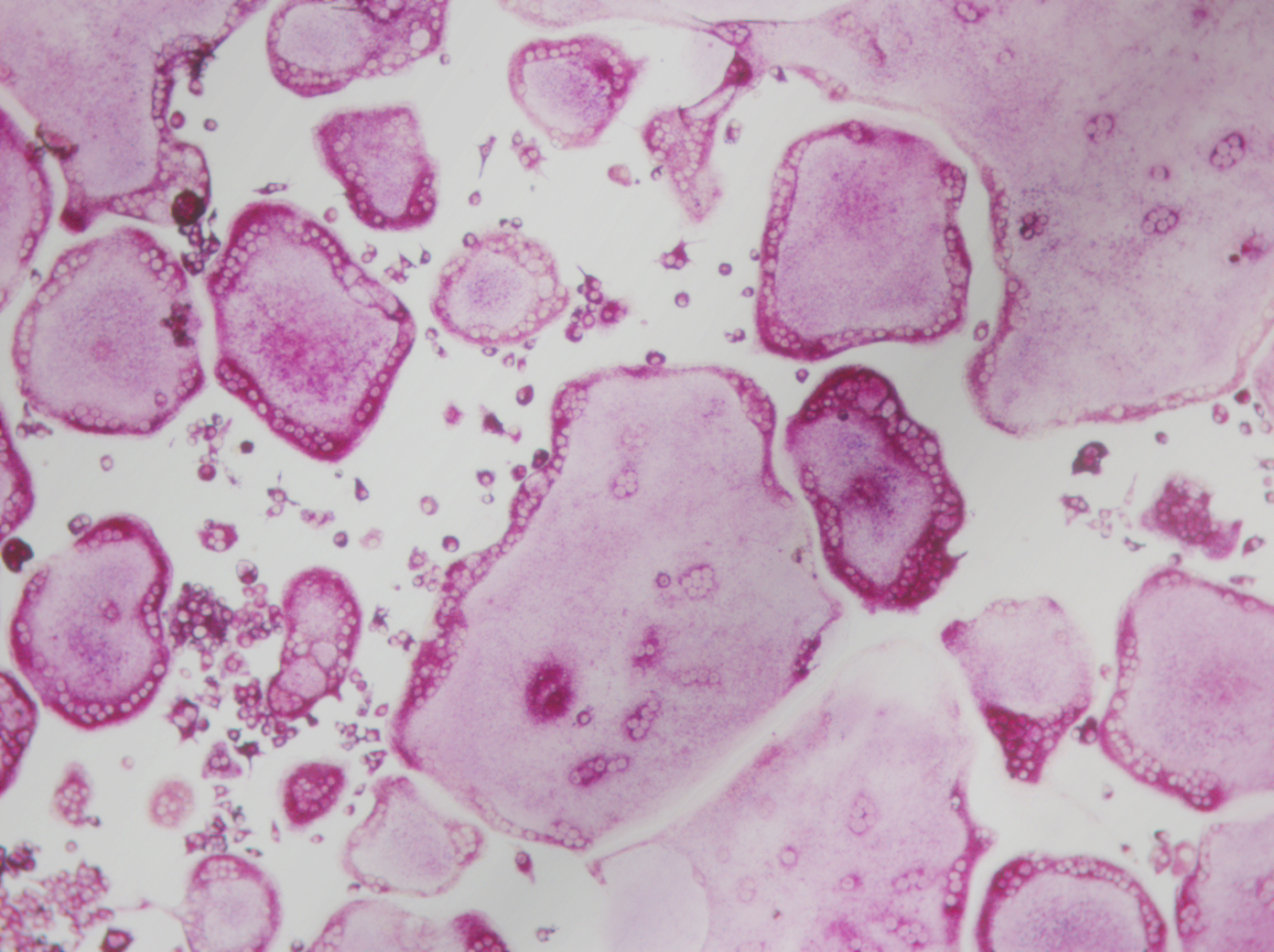

Supplement: Figure 4—source data 1. — The folder named ‘7,8-DHF’ contains micrographs of cells with or without 7,8-DHF treatment (individual files are named by the concentration of 7,8-DHF). The folder named ‘BDNF’ contains micrographs of cells with or without BDNF treatment (individual files are named by the concentration of BDNF). [file elife-64872-fig4-data1.zip › Figure 4-source data 1/BDNF/25 ng·mL-1 (2).jpg]

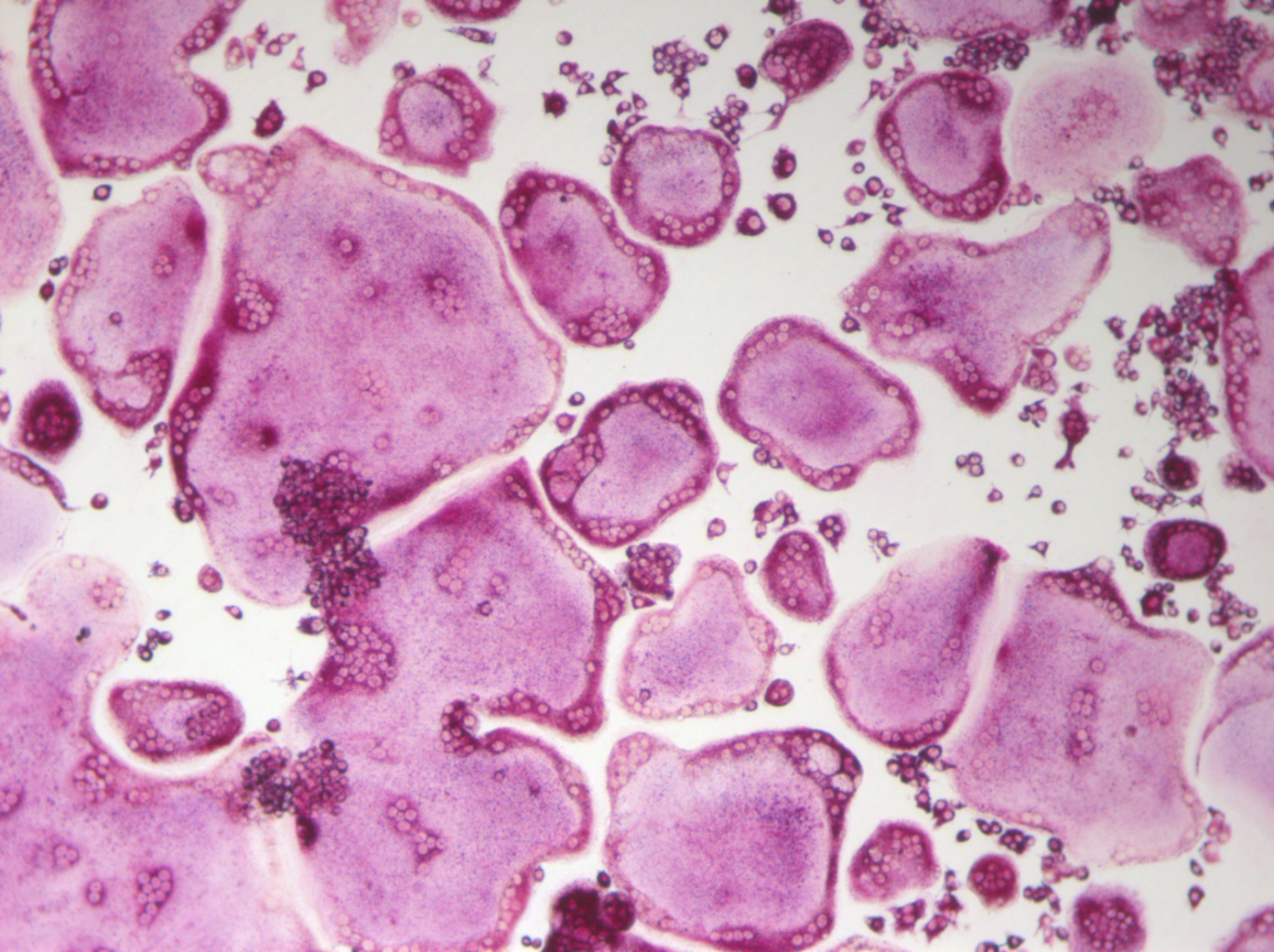

Supplement: Figure 4—source data 1. — The folder named ‘7,8-DHF’ contains micrographs of cells with or without 7,8-DHF treatment (individual files are named by the concentration of 7,8-DHF). The folder named ‘BDNF’ contains micrographs of cells with or without BDNF treatment (individual files are named by the concentration of BDNF). [file elife-64872-fig4-data1.zip › Figure 4-source data 1/BDNF/25 ng·mL-1 (3).jpg]

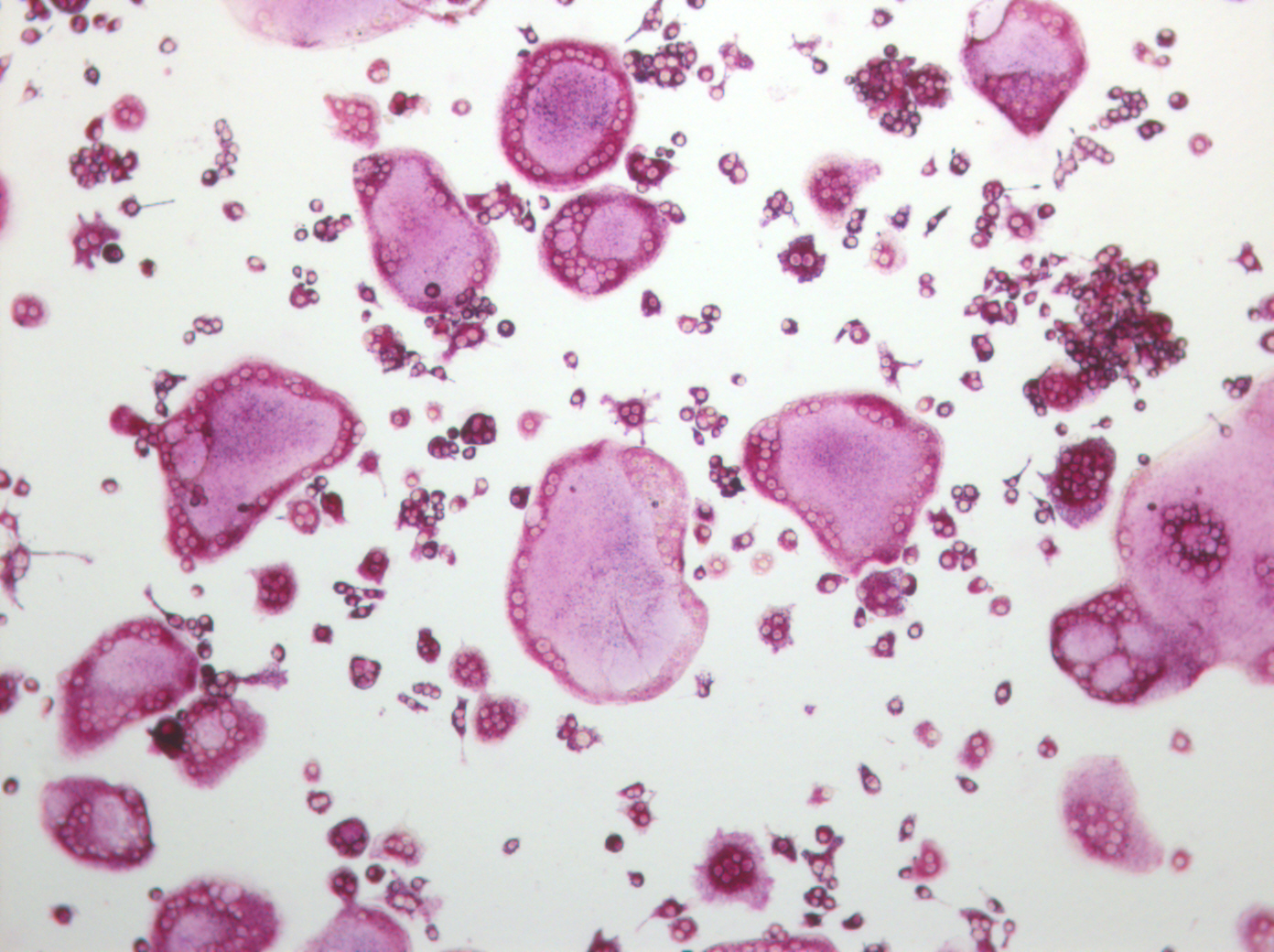

Supplement: Figure 4—source data 1. — The folder named ‘7,8-DHF’ contains micrographs of cells with or without 7,8-DHF treatment (individual files are named by the concentration of 7,8-DHF). The folder named ‘BDNF’ contains micrographs of cells with or without BDNF treatment (individual files are named by the concentration of BDNF). [file elife-64872-fig4-data1.zip › Figure 4-source data 1/BDNF/50 ng·mL-1 (1).jpg]

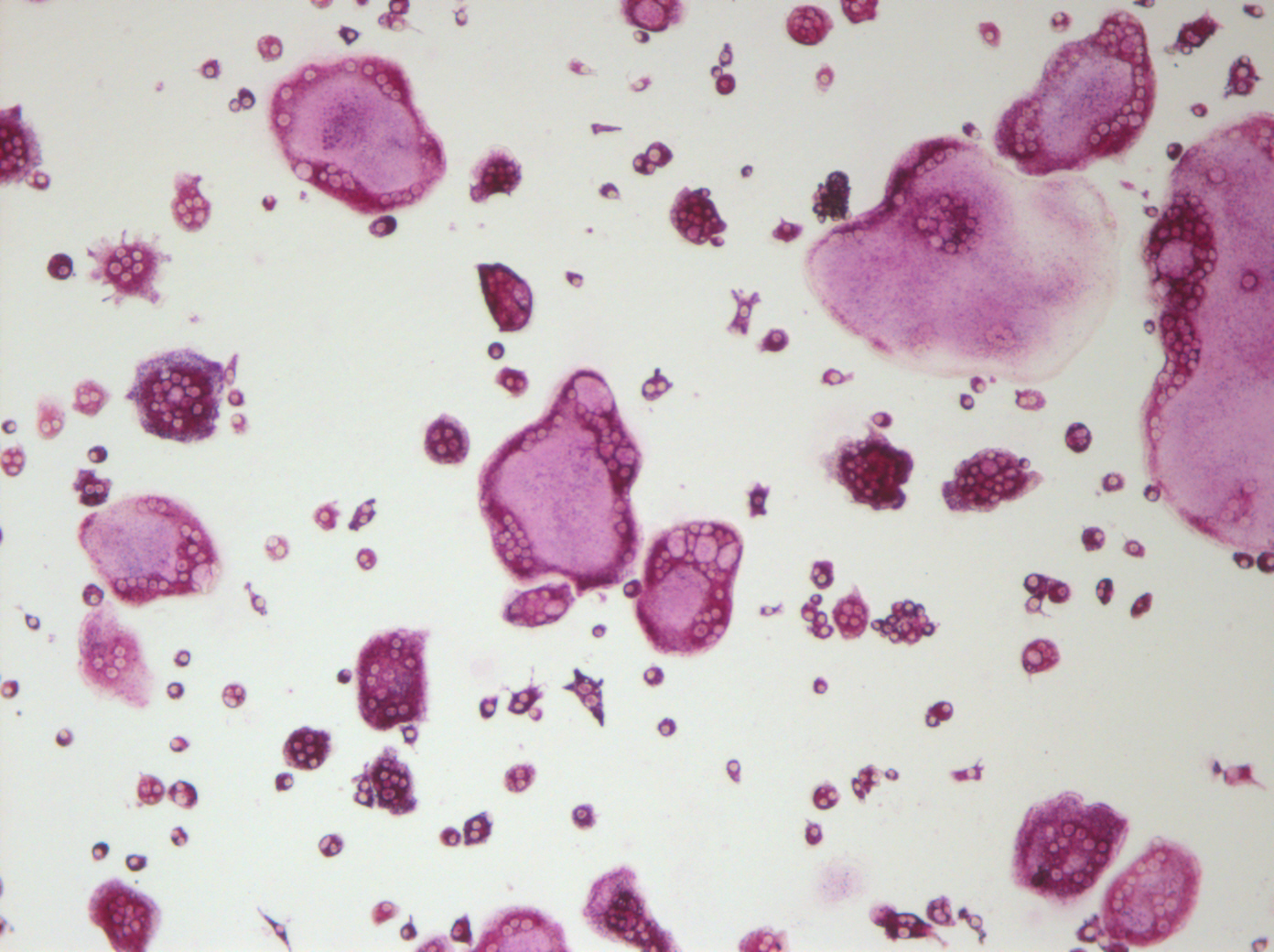

Supplement: Figure 4—source data 1. — The folder named ‘7,8-DHF’ contains micrographs of cells with or without 7,8-DHF treatment (individual files are named by the concentration of 7,8-DHF). The folder named ‘BDNF’ contains micrographs of cells with or without BDNF treatment (individual files are named by the concentration of BDNF). [file elife-64872-fig4-data1.zip › Figure 4-source data 1/BDNF/50 ng·mL-1 (2).jpg]

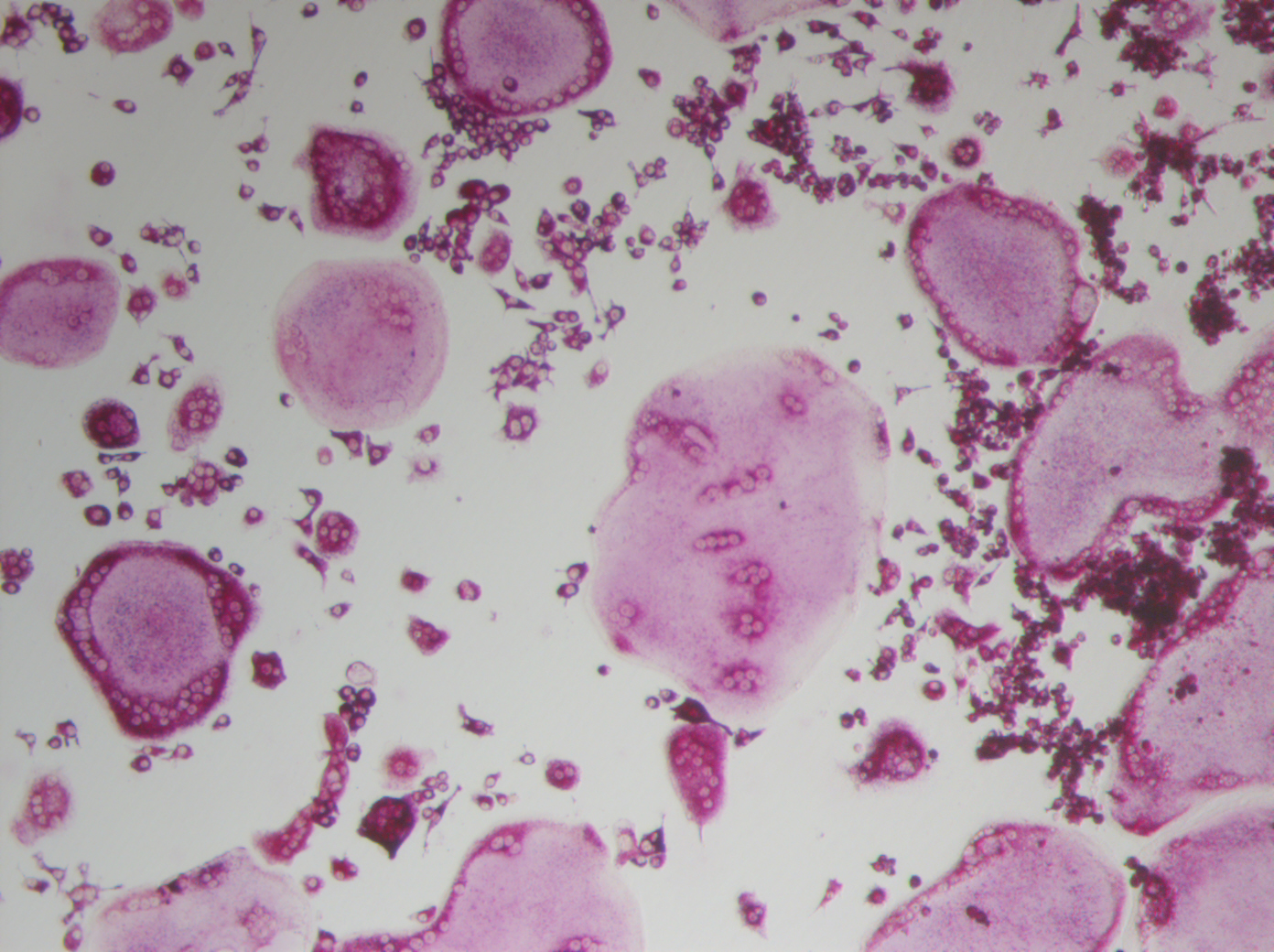

Supplement: Figure 4—source data 1. — The folder named ‘7,8-DHF’ contains micrographs of cells with or without 7,8-DHF treatment (individual files are named by the concentration of 7,8-DHF). The folder named ‘BDNF’ contains micrographs of cells with or without BDNF treatment (individual files are named by the concentration of BDNF). [file elife-64872-fig4-data1.zip › Figure 4-source data 1/BDNF/50 ng·mL-1 (3).jpg]

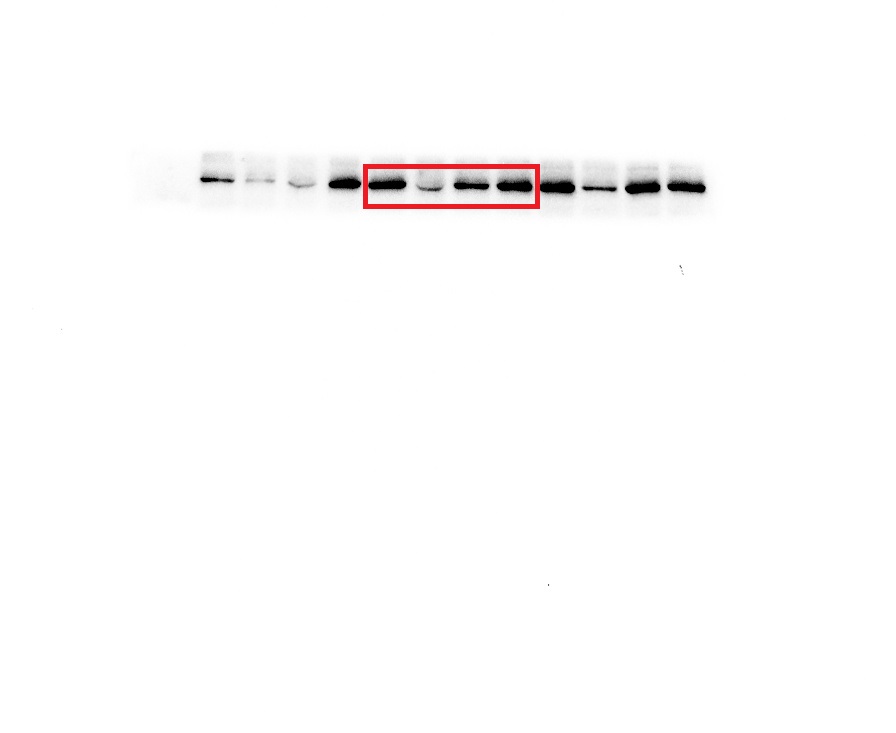

Supplement: Figure 4—source data 2. — The individual file name containing ‘(labeled)’ is blot with the relevant bands labeled by a red outline. [file elife-64872-fig4-data2.zip › Figure 4-source data 2/Adamts5 (labelled).jpg]

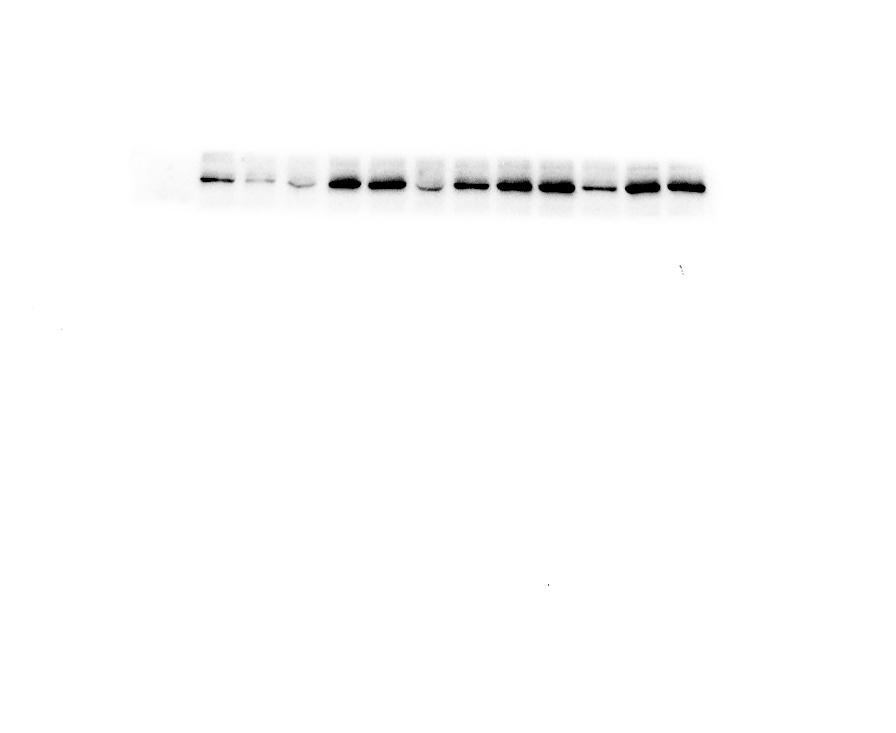

Supplement: Figure 4—source data 2. — The individual file name containing ‘(labeled)’ is blot with the relevant bands labeled by a red outline. [file elife-64872-fig4-data2.zip › Figure 4-source data 2/Adamts5.jpg]

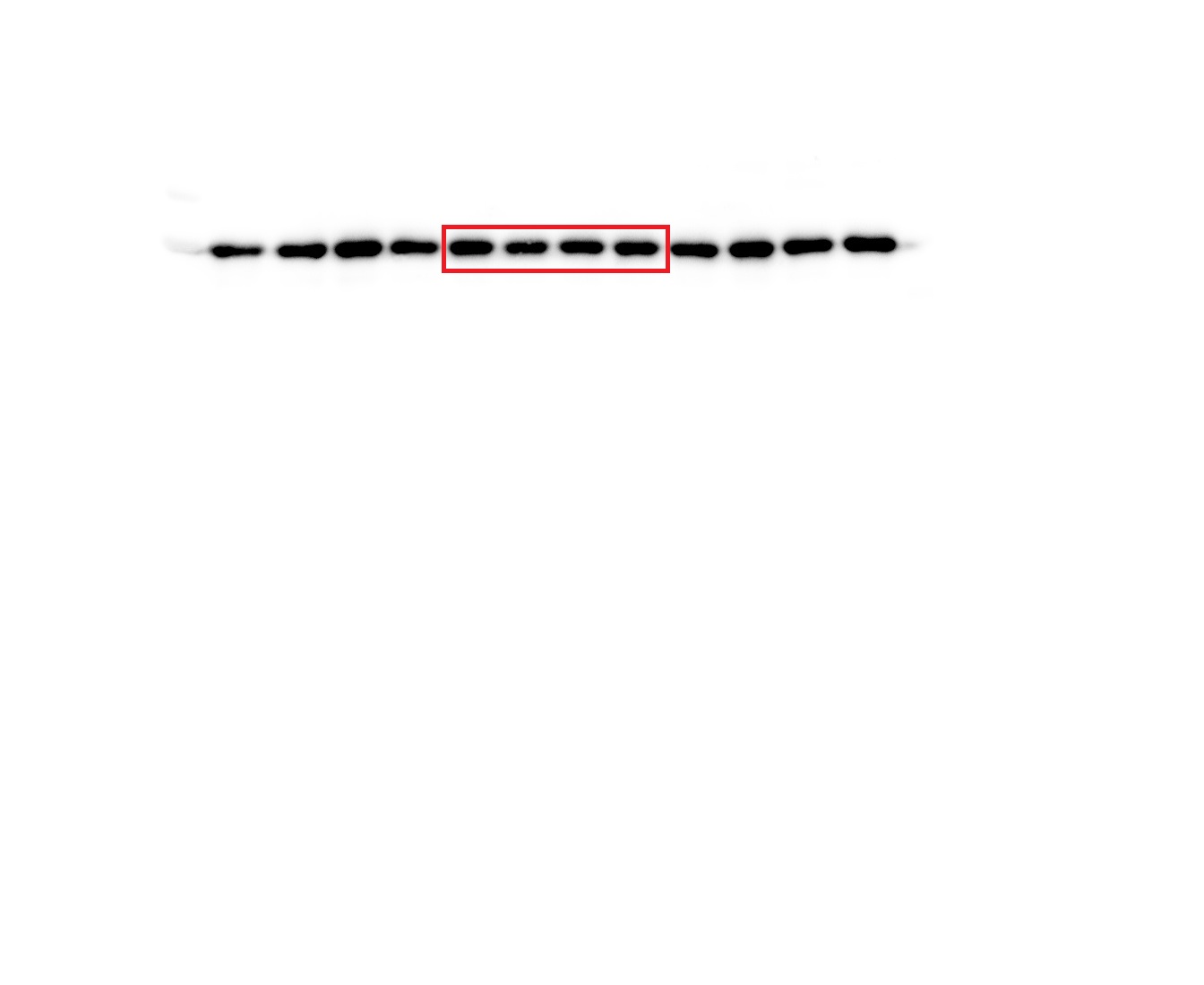

Supplement: Figure 4—source data 2. — The individual file name containing ‘(labeled)’ is blot with the relevant bands labeled by a red outline. [file elife-64872-fig4-data2.zip › Figure 4-source data 2/GAPDH (labelled).jpg]

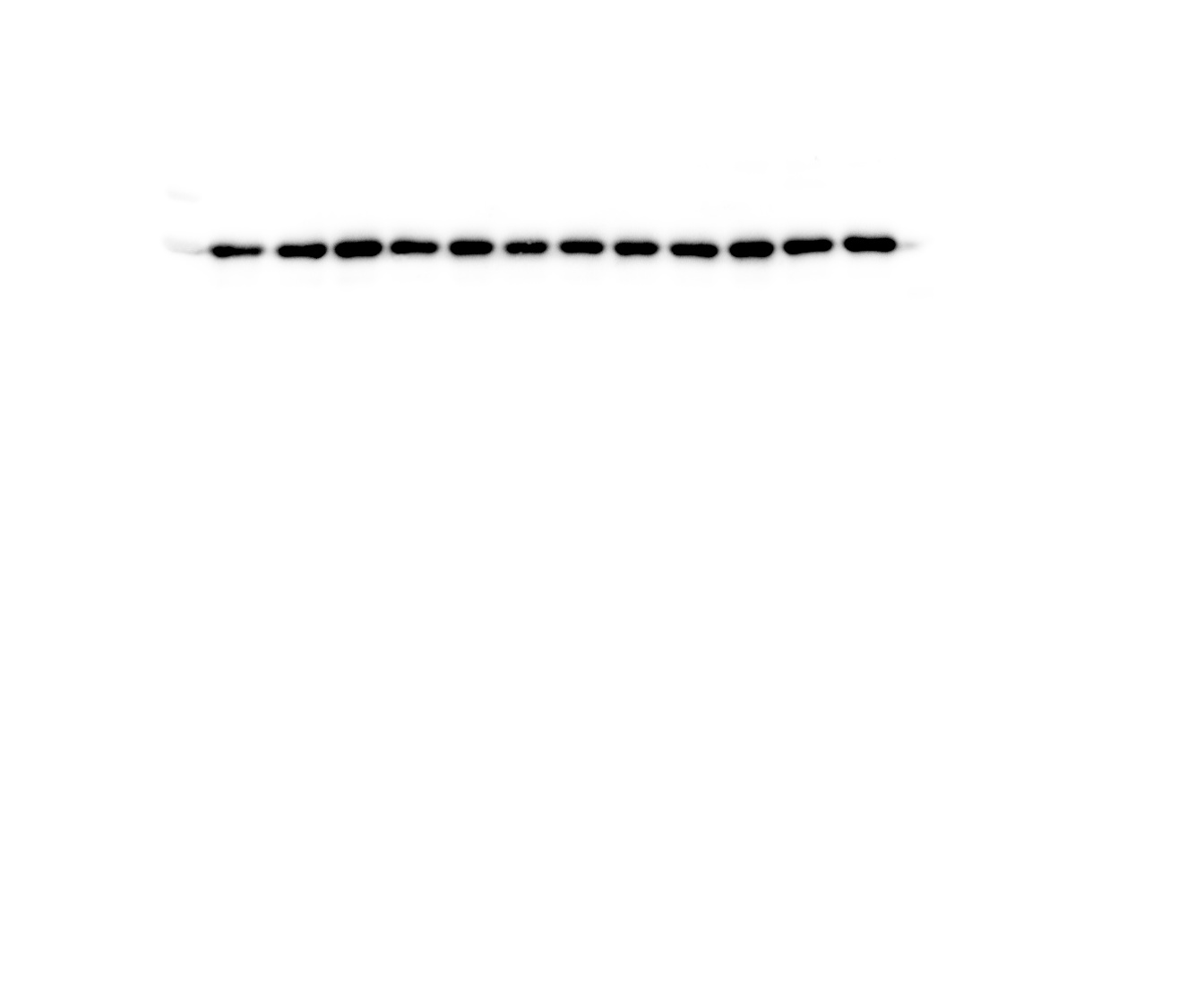

Supplement: Figure 4—source data 2. — The individual file name containing ‘(labeled)’ is blot with the relevant bands labeled by a red outline. [file elife-64872-fig4-data2.zip › Figure 4-source data 2/GAPDH.jpg]

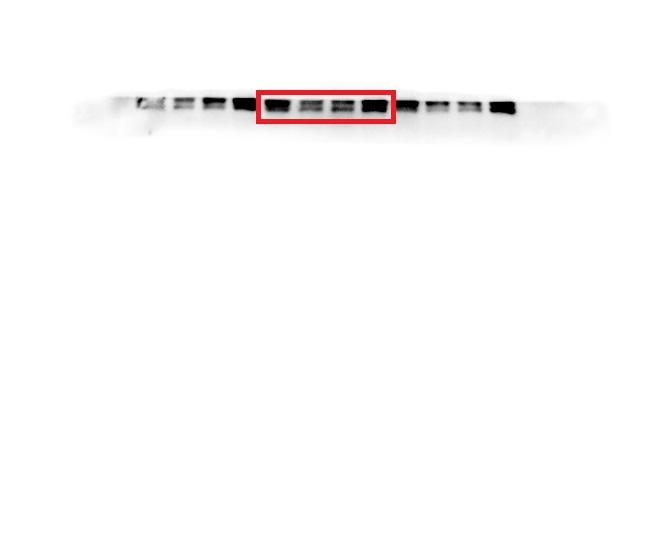

Supplement: Figure 4—source data 2. — The individual file name containing ‘(labeled)’ is blot with the relevant bands labeled by a red outline. [file elife-64872-fig4-data2.zip › Figure 4-source data 2/MMP-9 (labelled).jpg]

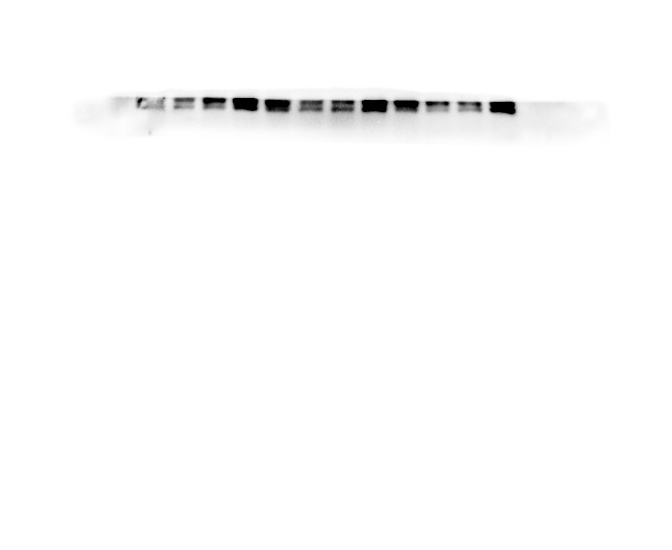

Supplement: Figure 4—source data 2. — The individual file name containing ‘(labeled)’ is blot with the relevant bands labeled by a red outline. [file elife-64872-fig4-data2.zip › Figure 4-source data 2/MMP-9.jpg]

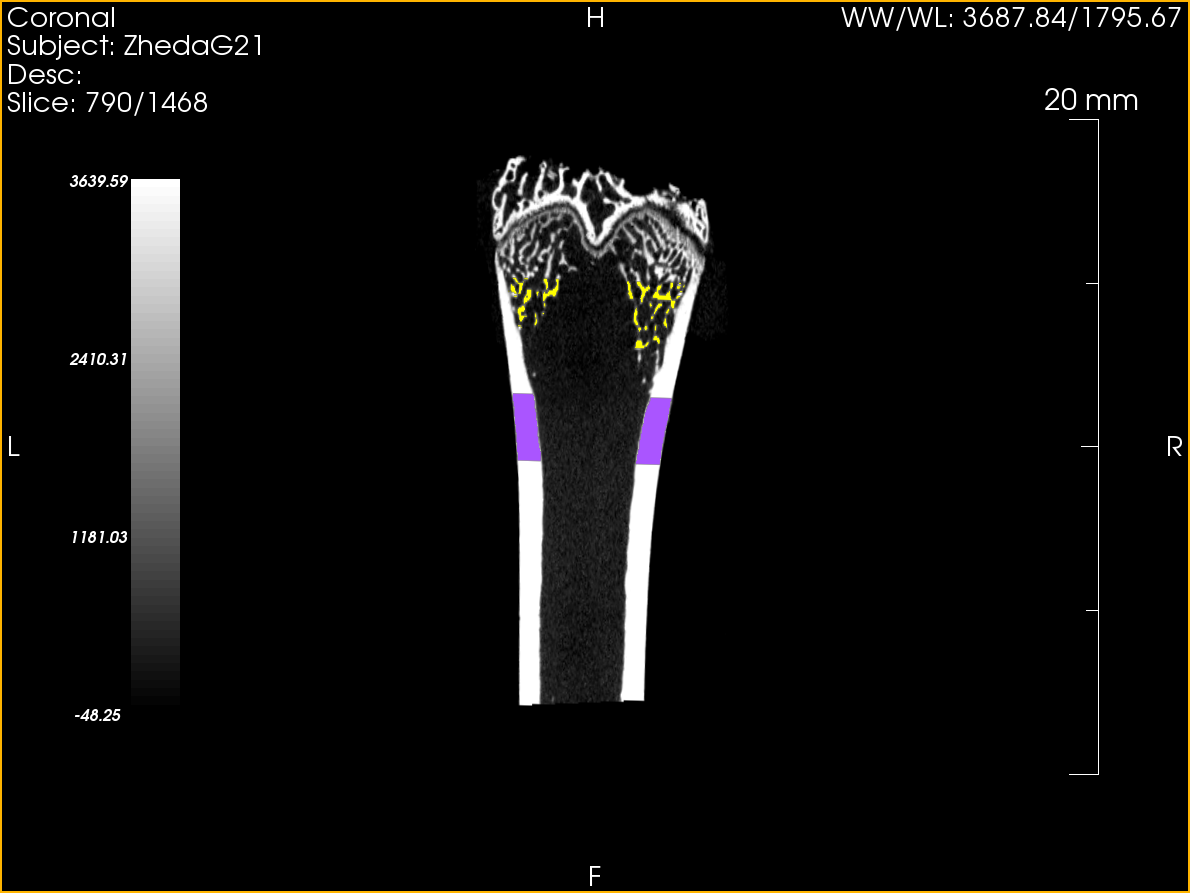

Supplement: Figure 5—source data 2. — The folders named ‘SHAM’, ‘OVX’, ‘OVX-L’, and ‘OVX-H’ contain the original images in Figure 5D. [file elife-64872-fig5-data2.zip › Figure 5-source data 2/OVX/OVX-1.png]

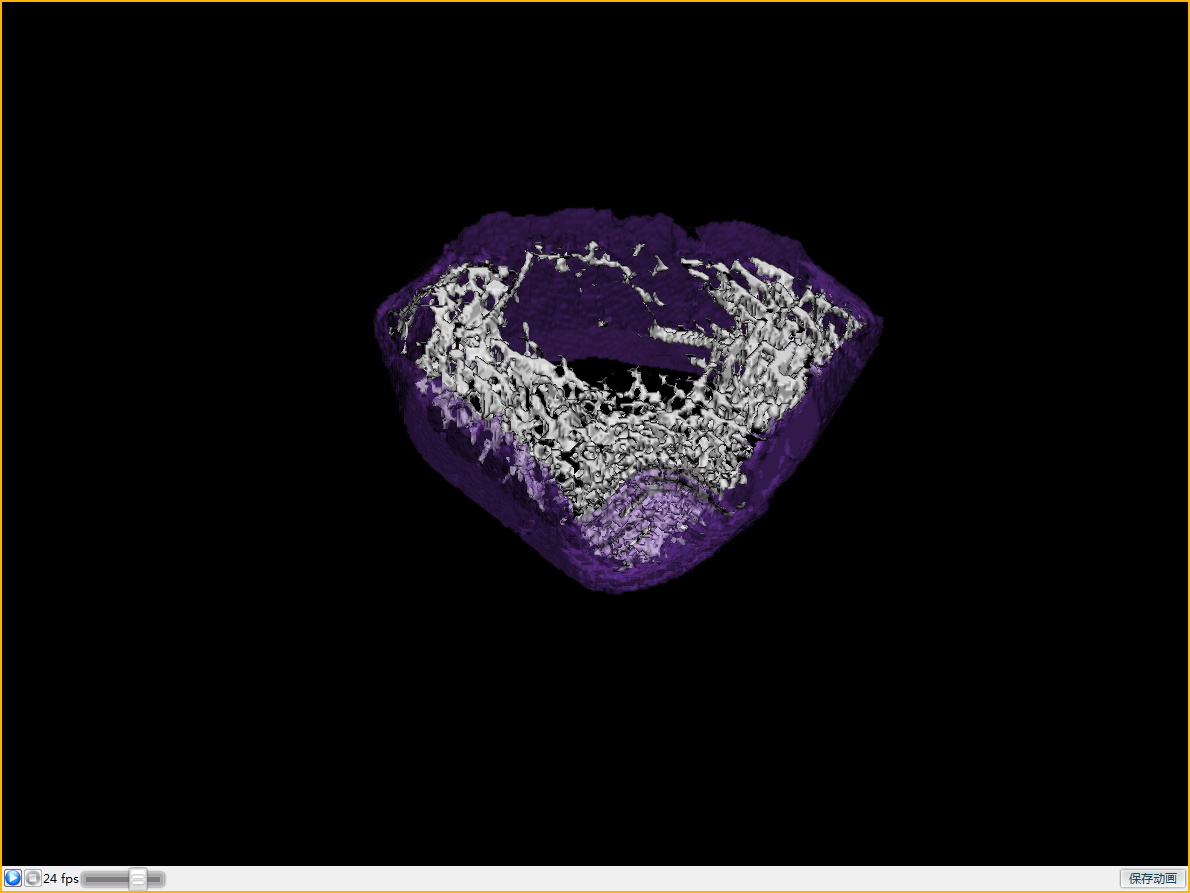

Supplement: Figure 5—source data 2. — The folders named ‘SHAM’, ‘OVX’, ‘OVX-L’, and ‘OVX-H’ contain the original images in Figure 5D. [file elife-64872-fig5-data2.zip › Figure 5-source data 2/OVX/OVX-2.png]

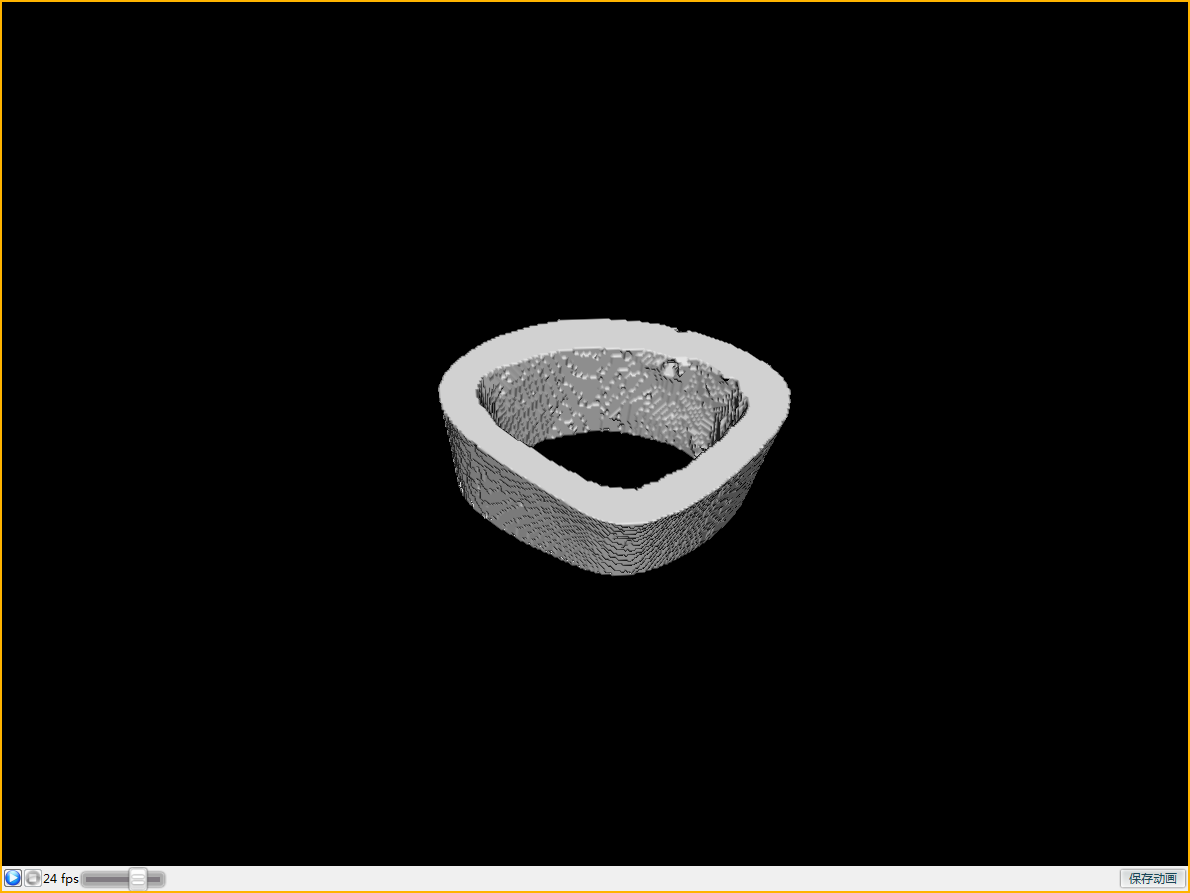

Supplement: Figure 5—source data 2. — The folders named ‘SHAM’, ‘OVX’, ‘OVX-L’, and ‘OVX-H’ contain the original images in Figure 5D. [file elife-64872-fig5-data2.zip › Figure 5-source data 2/OVX/OVX-3.png]

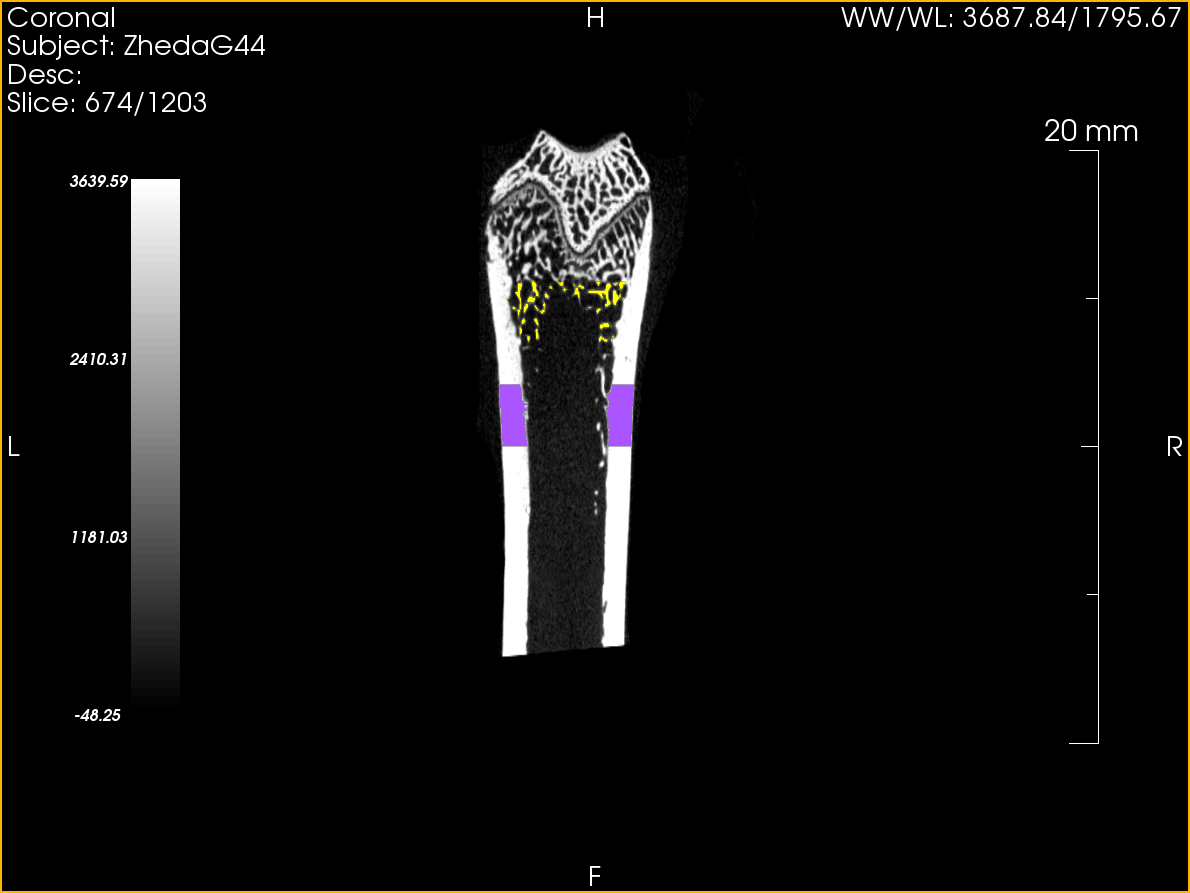

Supplement: Figure 5—source data 2. — The folders named ‘SHAM’, ‘OVX’, ‘OVX-L’, and ‘OVX-H’ contain the original images in Figure 5D. [file elife-64872-fig5-data2.zip › Figure 5-source data 2/OVX-H/OVX-H-1.png]

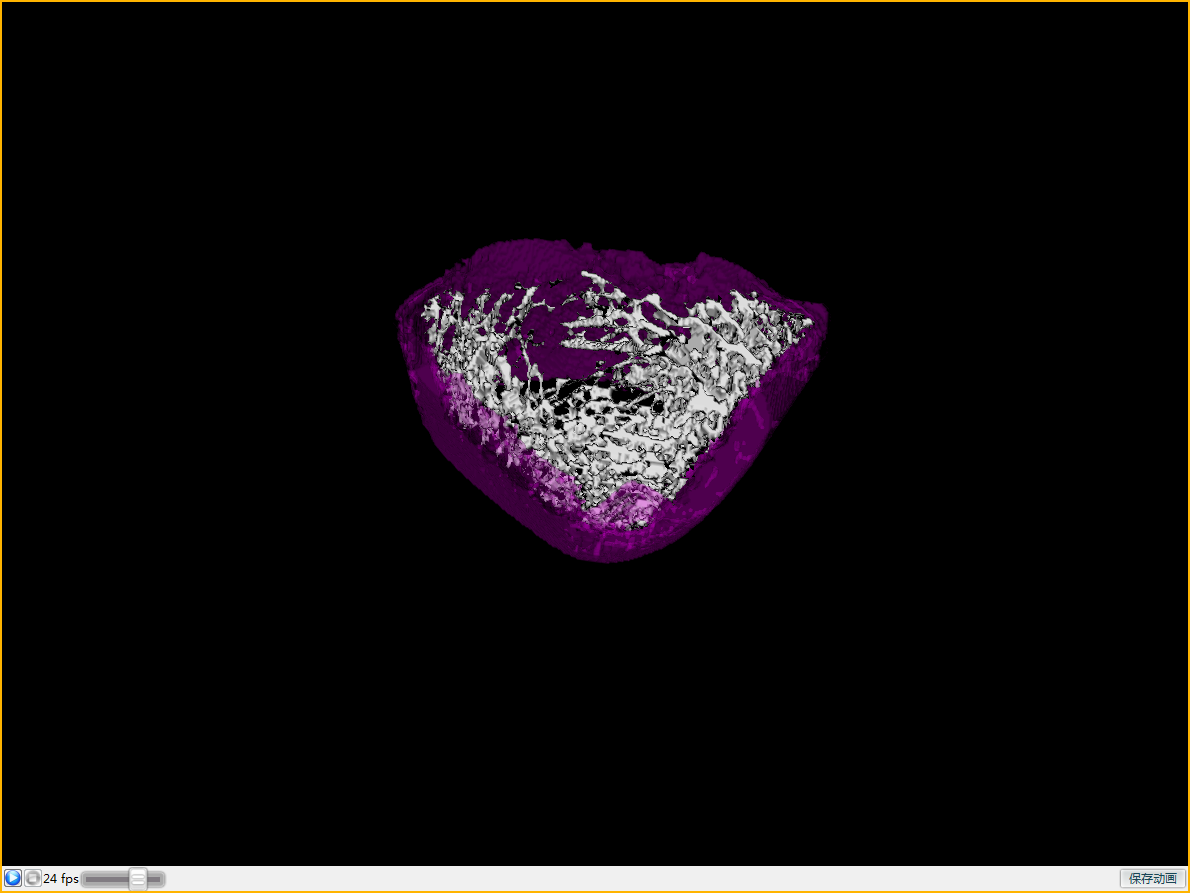

Supplement: Figure 5—source data 2. — The folders named ‘SHAM’, ‘OVX’, ‘OVX-L’, and ‘OVX-H’ contain the original images in Figure 5D. [file elife-64872-fig5-data2.zip › Figure 5-source data 2/OVX-H/OVX-H-2.png]

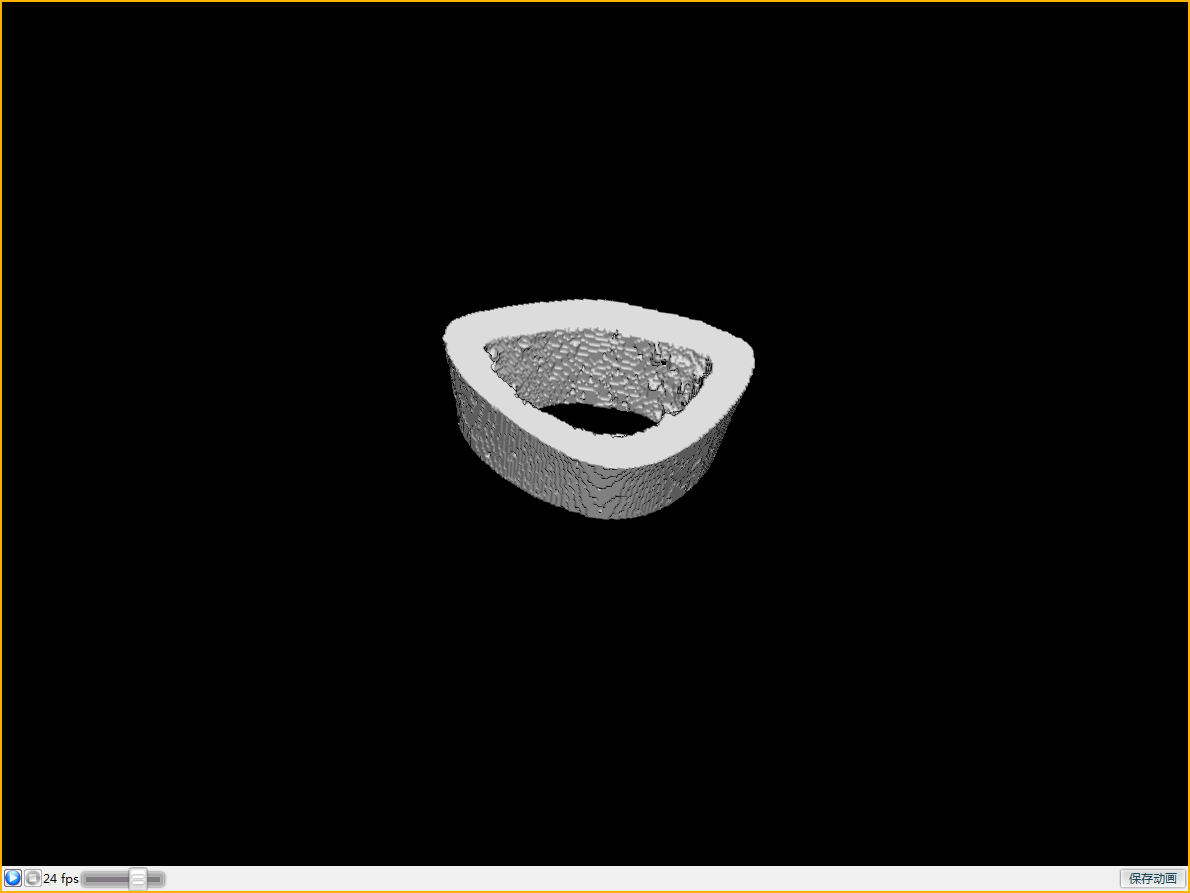

Supplement: Figure 5—source data 2. — The folders named ‘SHAM’, ‘OVX’, ‘OVX-L’, and ‘OVX-H’ contain the original images in Figure 5D. [file elife-64872-fig5-data2.zip › Figure 5-source data 2/OVX-H/OVX-H-3.png]

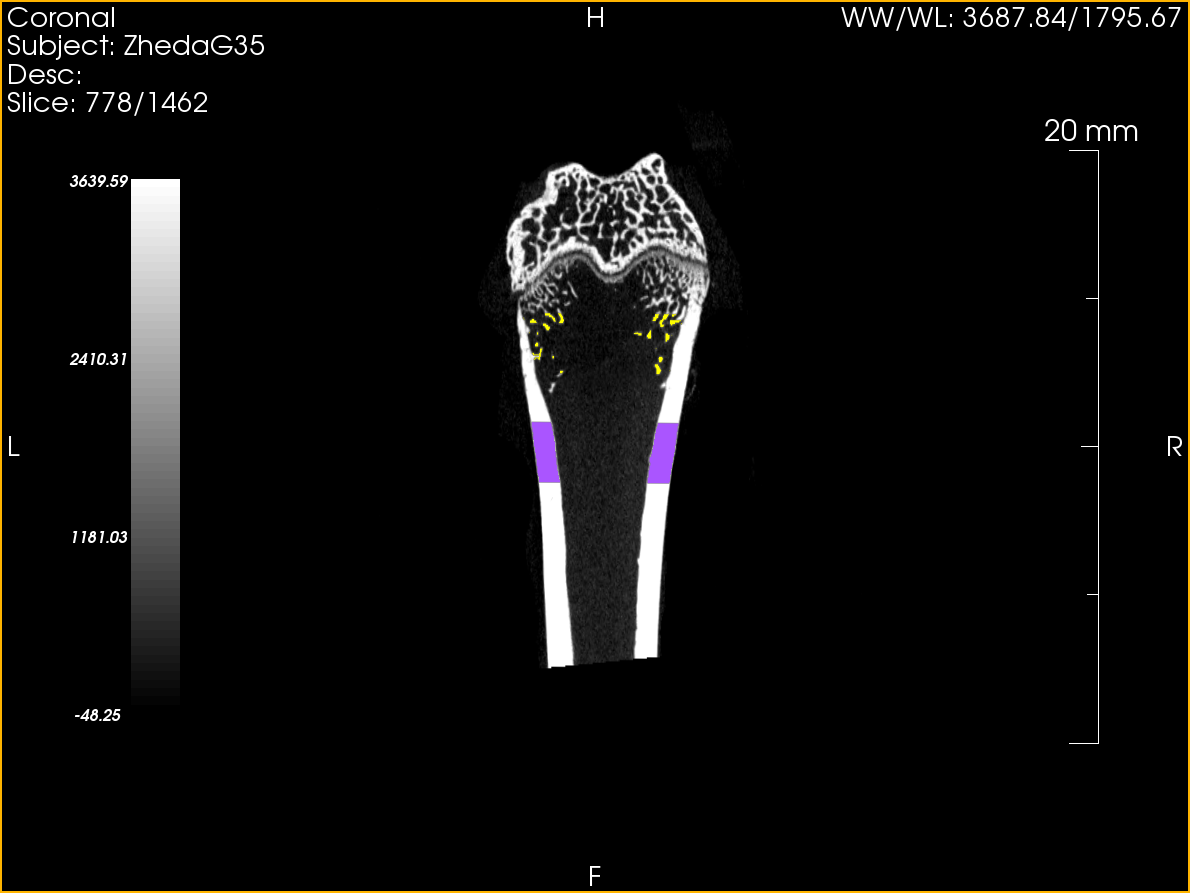

Supplement: Figure 5—source data 2. — The folders named ‘SHAM’, ‘OVX’, ‘OVX-L’, and ‘OVX-H’ contain the original images in Figure 5D. [file elife-64872-fig5-data2.zip › Figure 5-source data 2/OVX-L/OVX-L-1.png]
