# Supplementary figures and images for: 7,8-Dihydroxyflavone modulates bone formation and resorption and ameliorates ovariectomy-induced osteoporosis (part 2 of 2)
Source: eLife. 2021 Jul 6;10:e64872. doi: 10.7554/eLife.64872 (PMC8285109; doi:10.7554/eLife.64872)

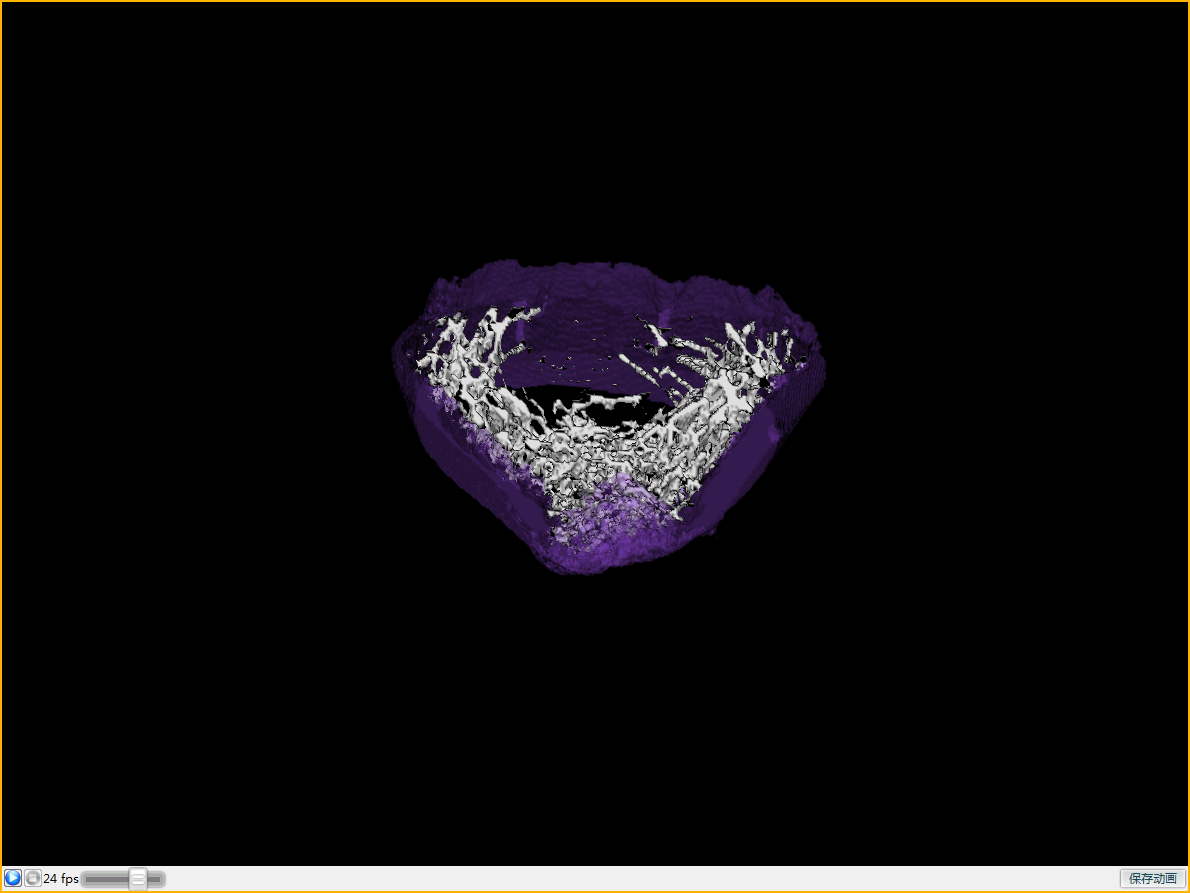

Supplement: Figure 5—source data 2. — The folders named ‘SHAM’, ‘OVX’, ‘OVX-L’, and ‘OVX-H’ contain the original images in Figure 5D. [file elife-64872-fig5-data2.zip › Figure 5-source data 2/OVX-L/OVX-L-2.png]

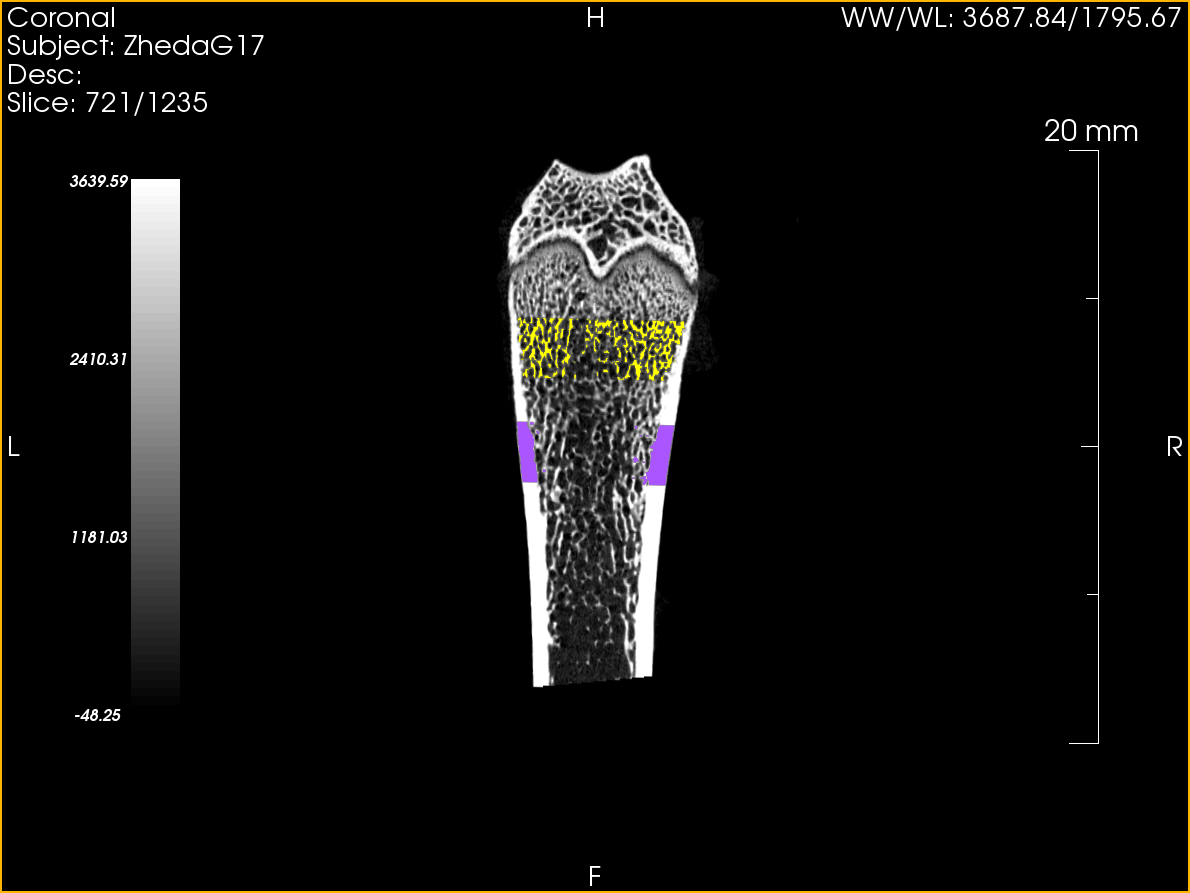

Supplement: Figure 5—source data 2. — The folders named ‘SHAM’, ‘OVX’, ‘OVX-L’, and ‘OVX-H’ contain the original images in Figure 5D. [file elife-64872-fig5-data2.zip › Figure 5-source data 2/SHAM/SHAM-1.png]

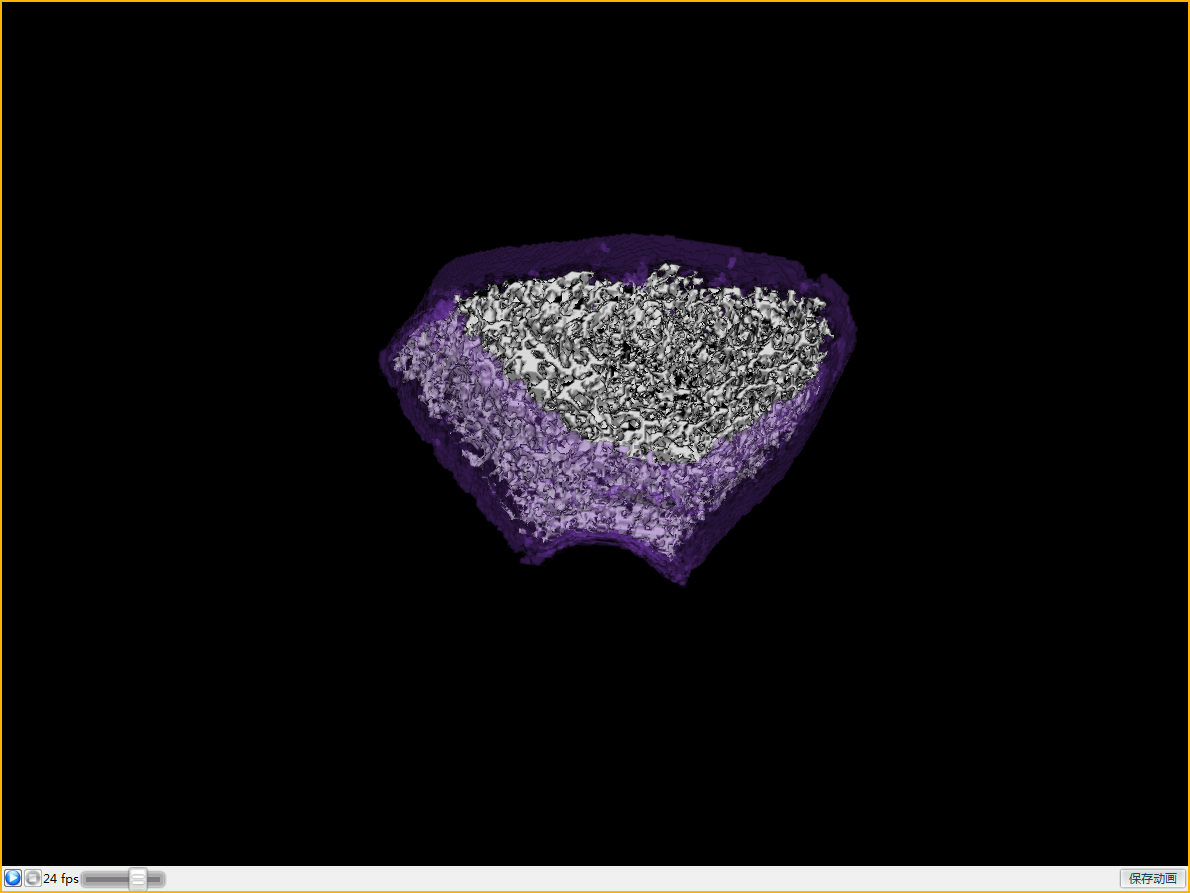

Supplement: Figure 5—source data 2. — The folders named ‘SHAM’, ‘OVX’, ‘OVX-L’, and ‘OVX-H’ contain the original images in Figure 5D. [file elife-64872-fig5-data2.zip › Figure 5-source data 2/SHAM/SHAM-2.png]

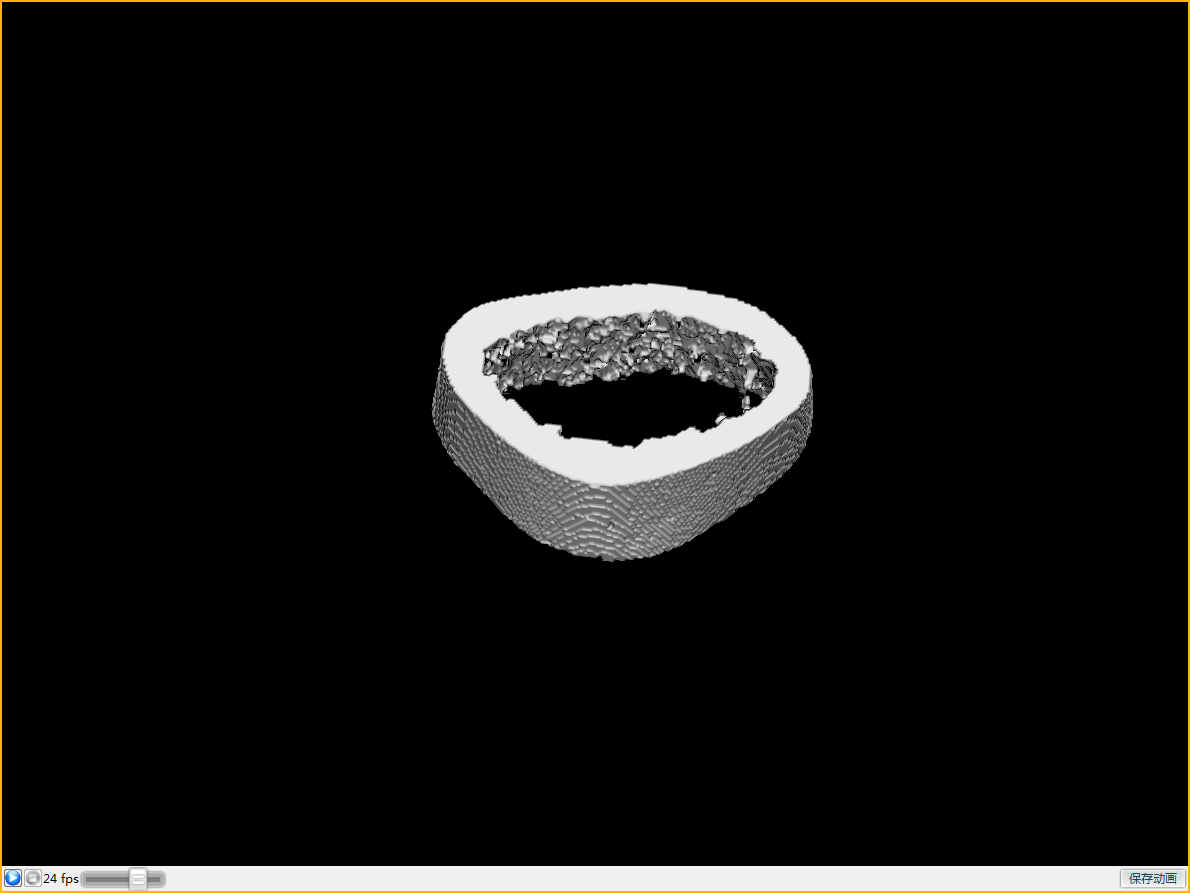

Supplement: Figure 5—source data 2. — The folders named ‘SHAM’, ‘OVX’, ‘OVX-L’, and ‘OVX-H’ contain the original images in Figure 5D. [file elife-64872-fig5-data2.zip › Figure 5-source data 2/SHAM/SHAM-3.png]

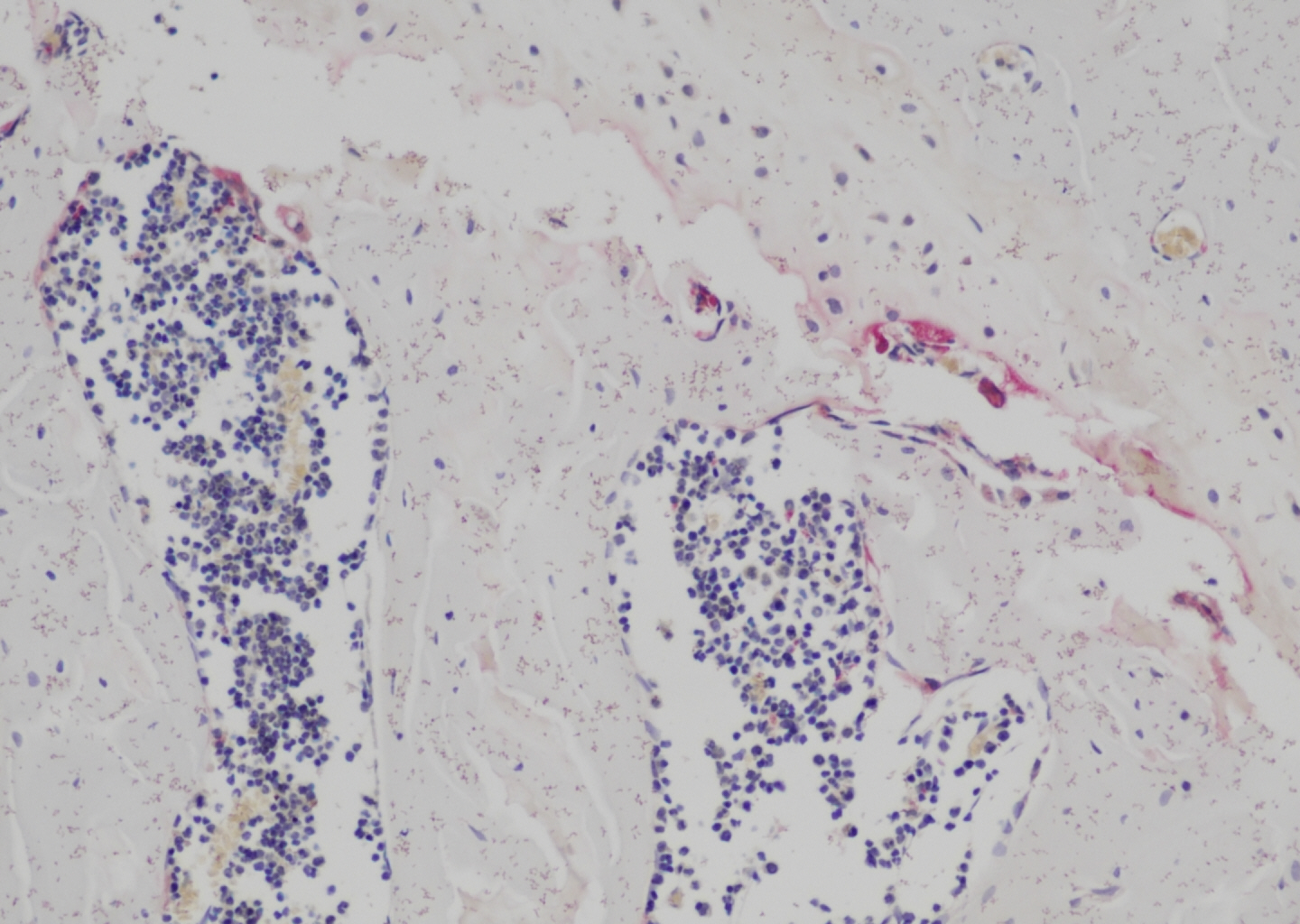

Supplement: Figure 6—source data 1. — The folders named ‘SHAM’, ‘OVX’, ‘OVX-L’, and ‘OVX-H’ contain micrographs of ‘SHAM’, ‘OVX’, ‘OVX-L’, and ‘OVX-H’ groups, respectively. [file elife-64872-fig6-data1.zip › Figure 6-source data 1/OVX/OVX-1.jpg]

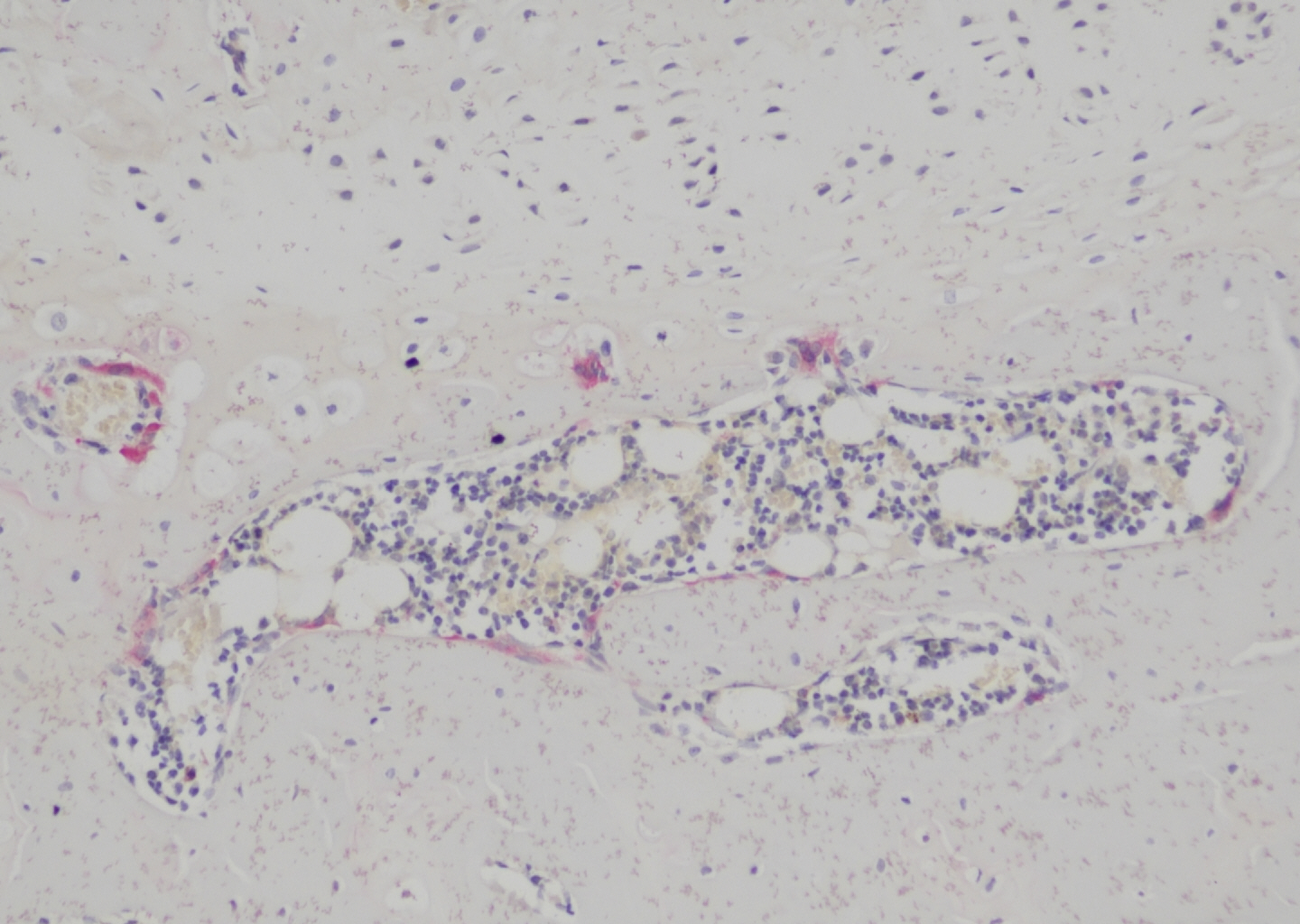

Supplement: Figure 6—source data 1. — The folders named ‘SHAM’, ‘OVX’, ‘OVX-L’, and ‘OVX-H’ contain micrographs of ‘SHAM’, ‘OVX’, ‘OVX-L’, and ‘OVX-H’ groups, respectively. [file elife-64872-fig6-data1.zip › Figure 6-source data 1/OVX/OVX-2.jpg]

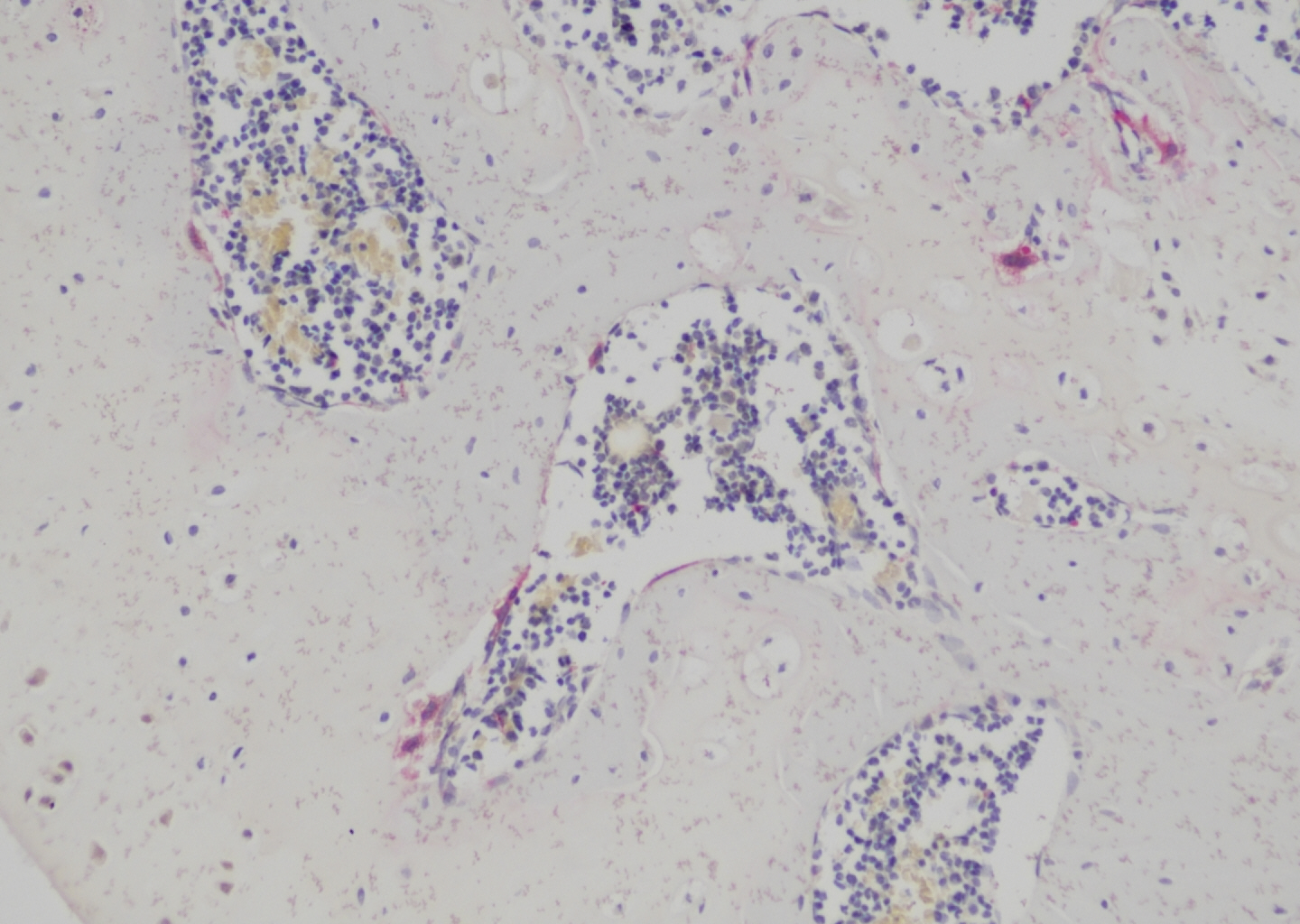

Supplement: Figure 6—source data 1. — The folders named ‘SHAM’, ‘OVX’, ‘OVX-L’, and ‘OVX-H’ contain micrographs of ‘SHAM’, ‘OVX’, ‘OVX-L’, and ‘OVX-H’ groups, respectively. [file elife-64872-fig6-data1.zip › Figure 6-source data 1/OVX/OVX-3.jpg]

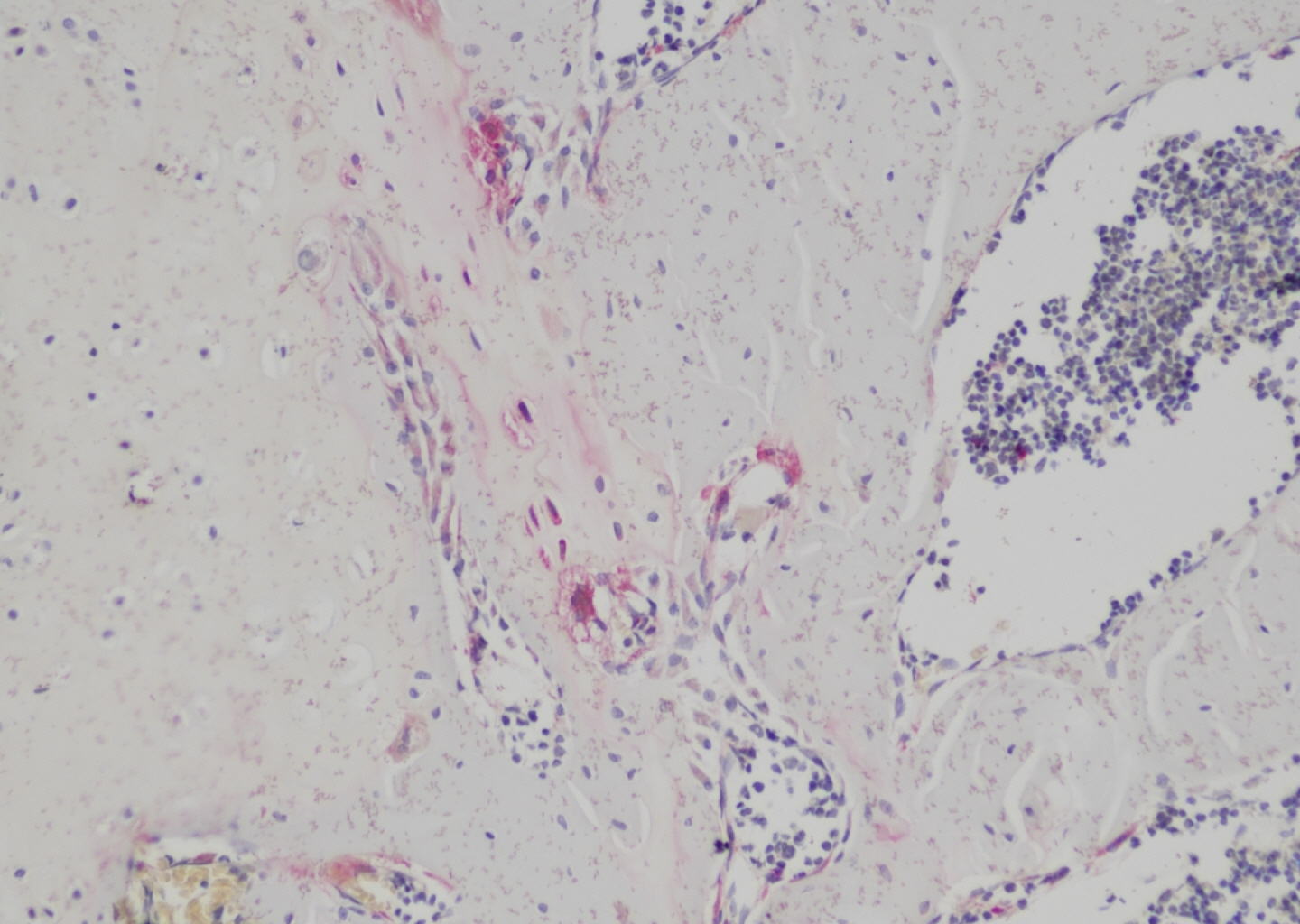

Supplement: Figure 6—source data 1. — The folders named ‘SHAM’, ‘OVX’, ‘OVX-L’, and ‘OVX-H’ contain micrographs of ‘SHAM’, ‘OVX’, ‘OVX-L’, and ‘OVX-H’ groups, respectively. [file elife-64872-fig6-data1.zip › Figure 6-source data 1/OVX/OVX-4.jpg]

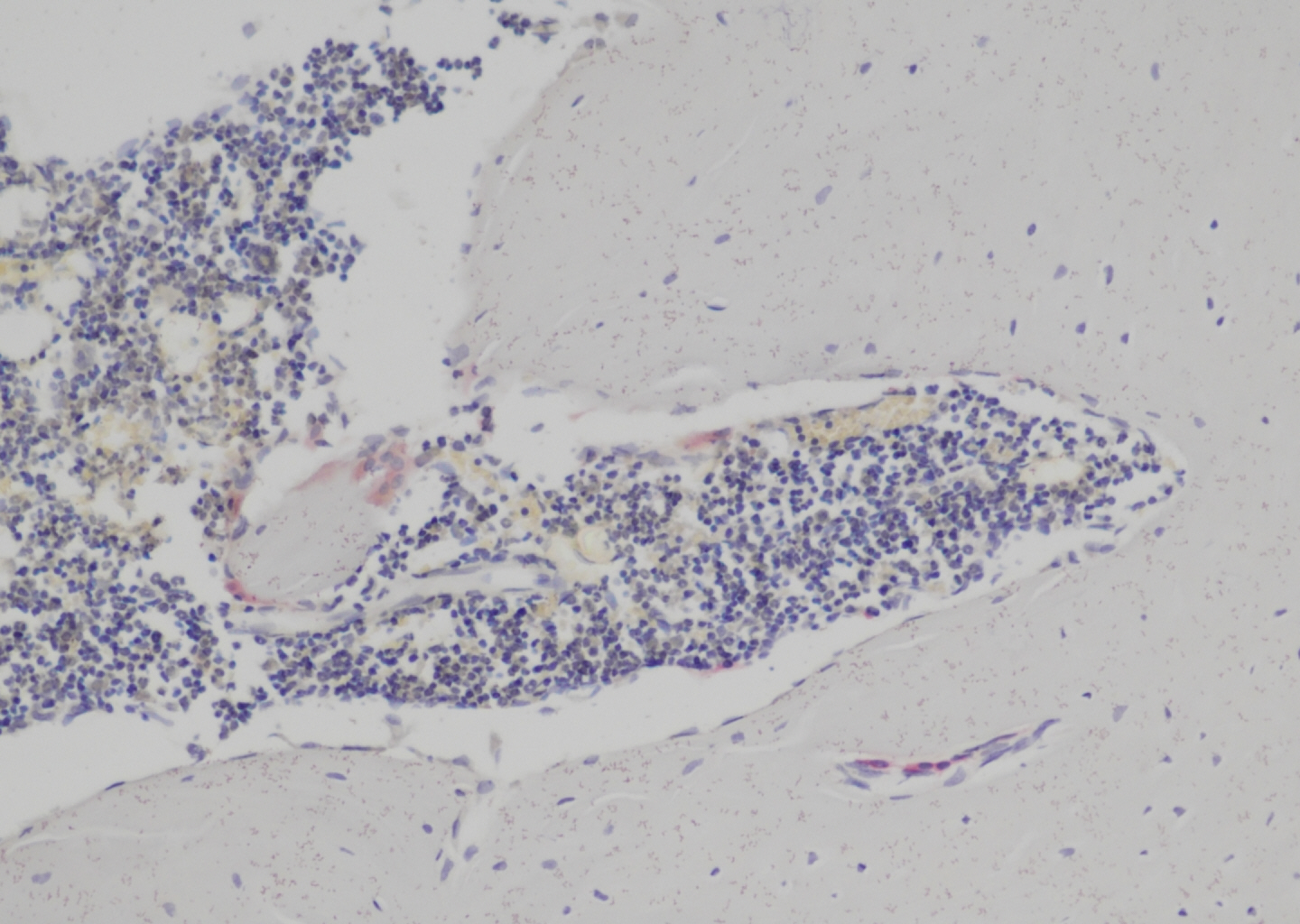

Supplement: Figure 6—source data 1. — The folders named ‘SHAM’, ‘OVX’, ‘OVX-L’, and ‘OVX-H’ contain micrographs of ‘SHAM’, ‘OVX’, ‘OVX-L’, and ‘OVX-H’ groups, respectively. [file elife-64872-fig6-data1.zip › Figure 6-source data 1/OVX/OVX-5.jpg]

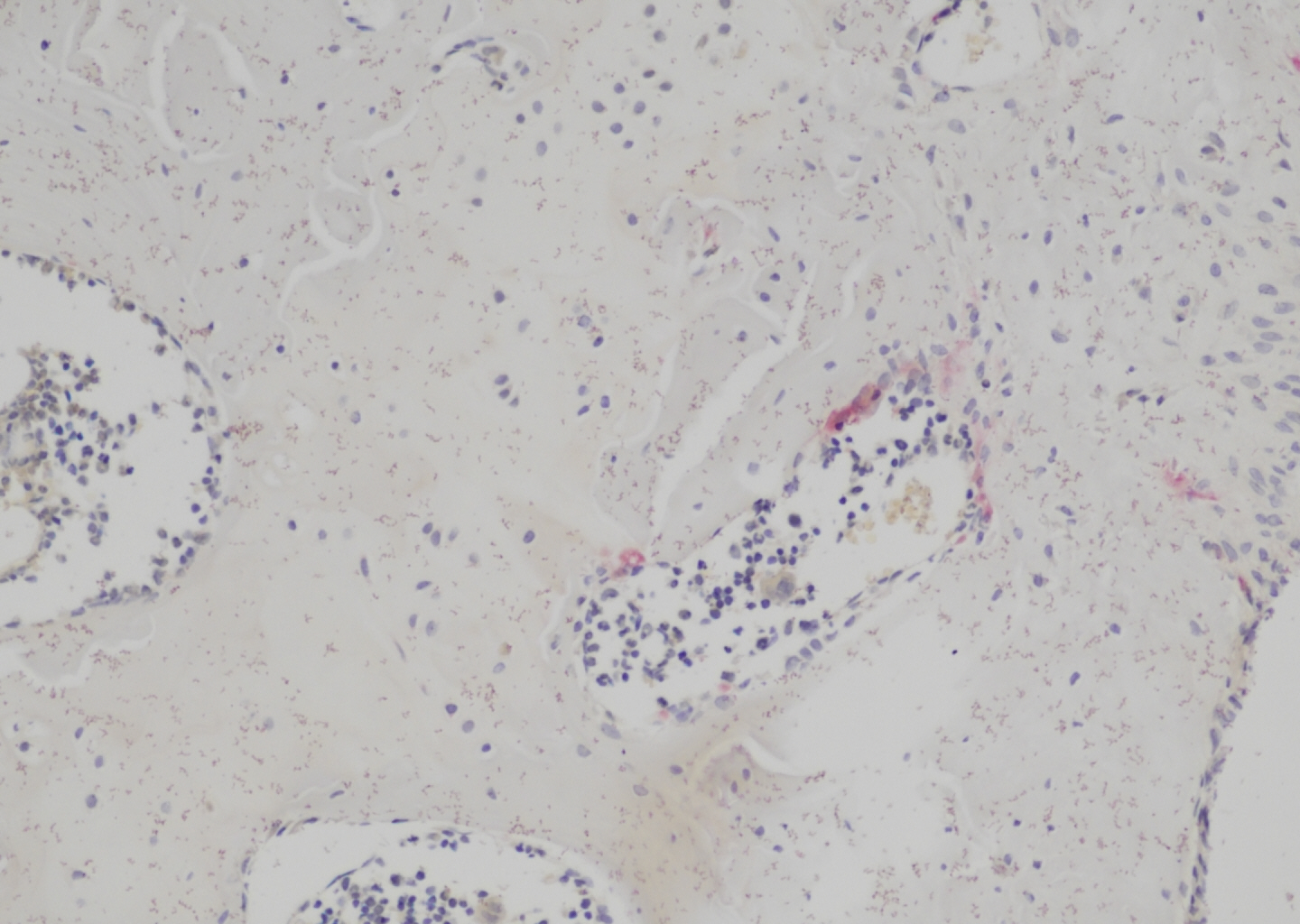

Supplement: Figure 6—source data 1. — The folders named ‘SHAM’, ‘OVX’, ‘OVX-L’, and ‘OVX-H’ contain micrographs of ‘SHAM’, ‘OVX’, ‘OVX-L’, and ‘OVX-H’ groups, respectively. [file elife-64872-fig6-data1.zip › Figure 6-source data 1/OVX-H/OVX-H-1.jpg]

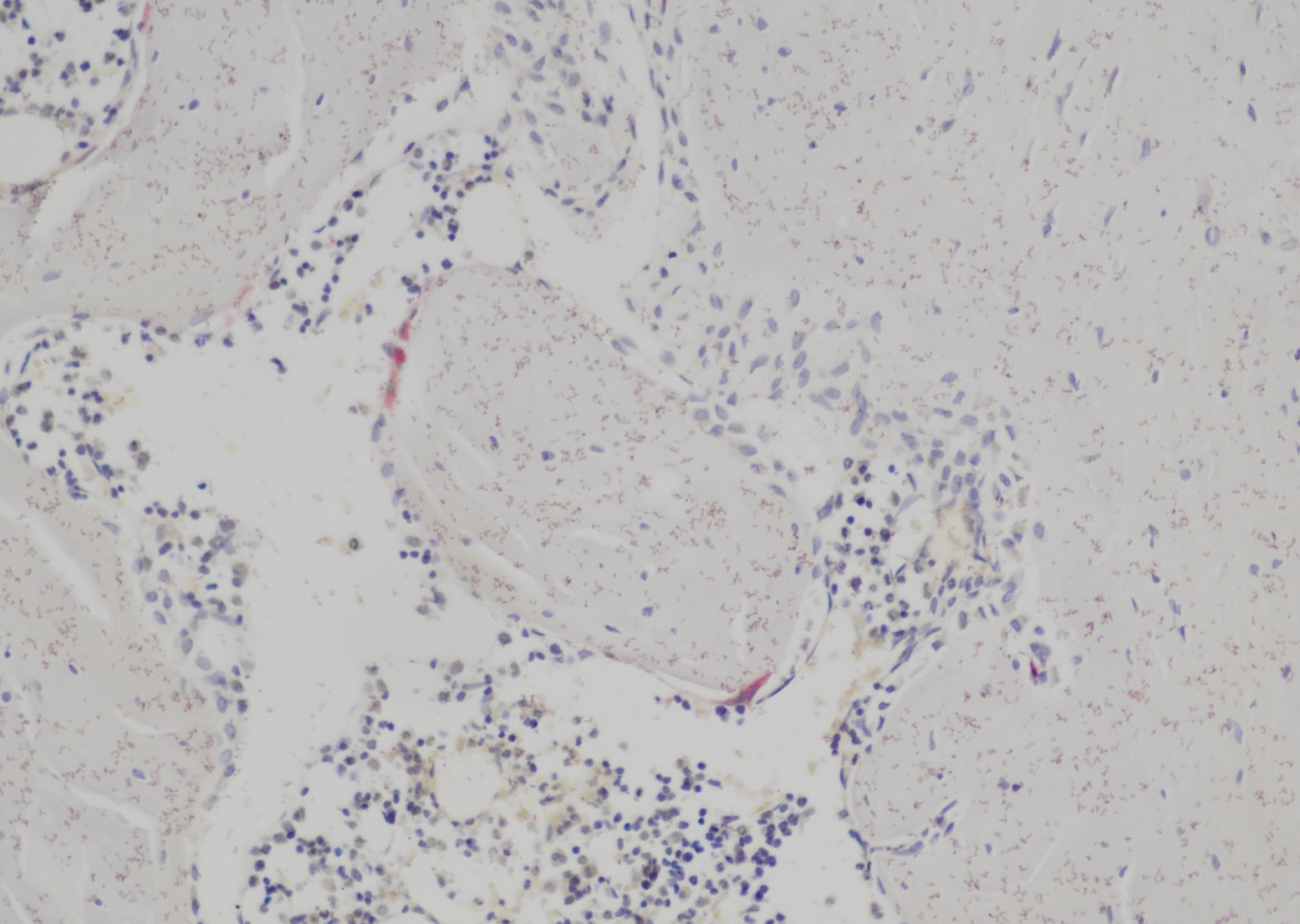

Supplement: Figure 6—source data 1. — The folders named ‘SHAM’, ‘OVX’, ‘OVX-L’, and ‘OVX-H’ contain micrographs of ‘SHAM’, ‘OVX’, ‘OVX-L’, and ‘OVX-H’ groups, respectively. [file elife-64872-fig6-data1.zip › Figure 6-source data 1/OVX-H/OVX-H-2.jpg]

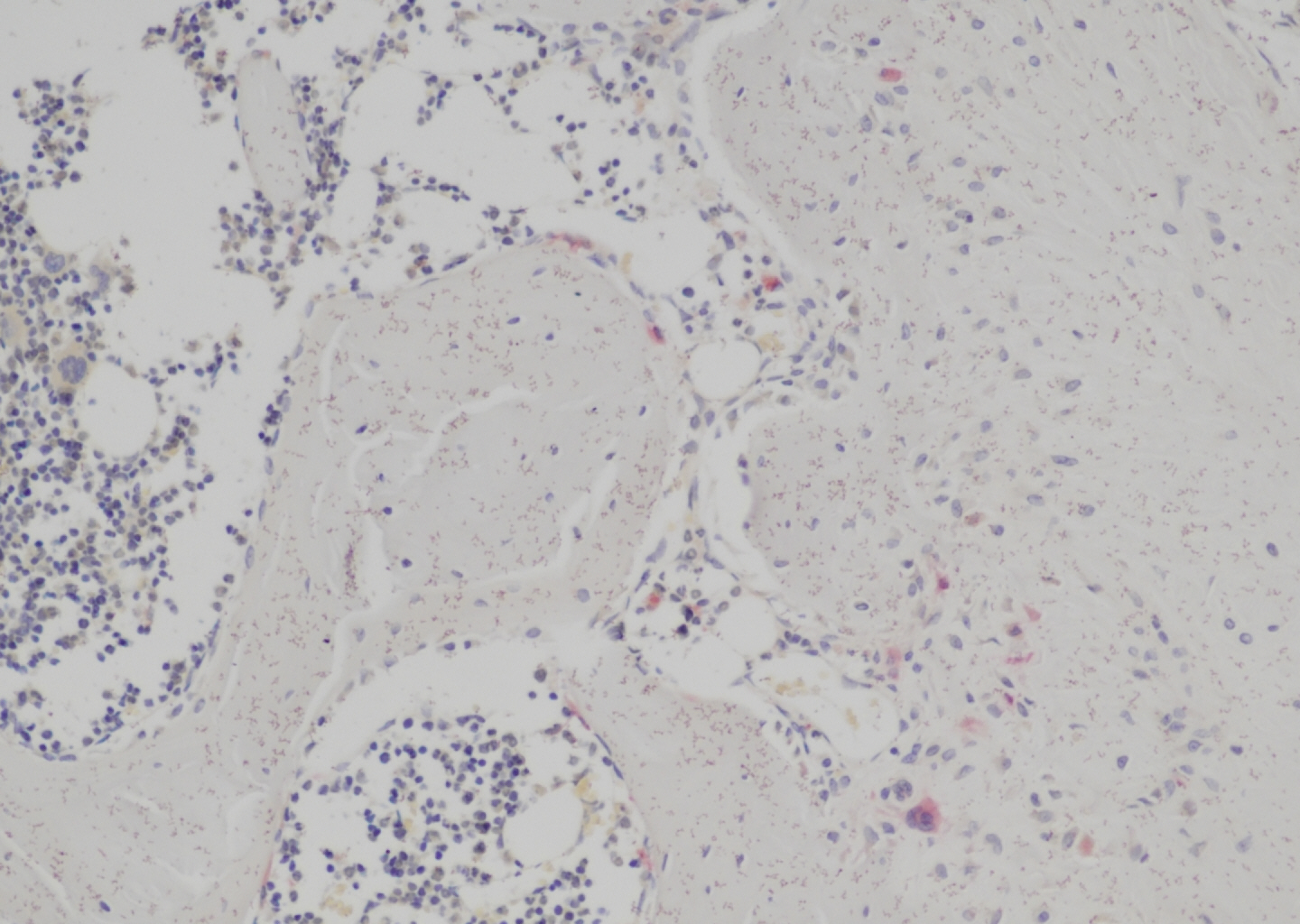

Supplement: Figure 6—source data 1. — The folders named ‘SHAM’, ‘OVX’, ‘OVX-L’, and ‘OVX-H’ contain micrographs of ‘SHAM’, ‘OVX’, ‘OVX-L’, and ‘OVX-H’ groups, respectively. [file elife-64872-fig6-data1.zip › Figure 6-source data 1/OVX-H/OVX-H-3.jpg]

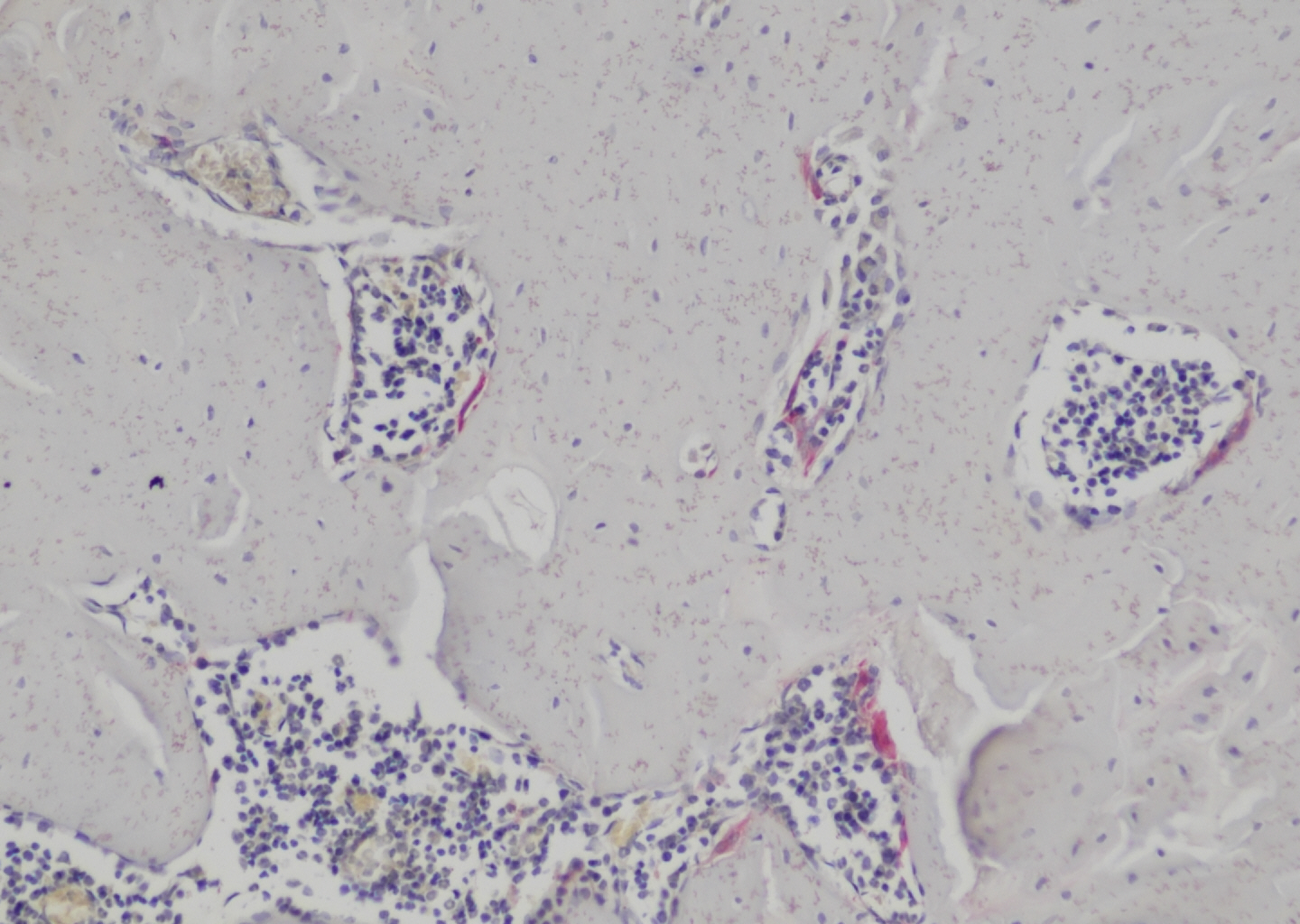

Supplement: Figure 6—source data 1. — The folders named ‘SHAM’, ‘OVX’, ‘OVX-L’, and ‘OVX-H’ contain micrographs of ‘SHAM’, ‘OVX’, ‘OVX-L’, and ‘OVX-H’ groups, respectively. [file elife-64872-fig6-data1.zip › Figure 6-source data 1/OVX-H/OVX-H-4.jpg]

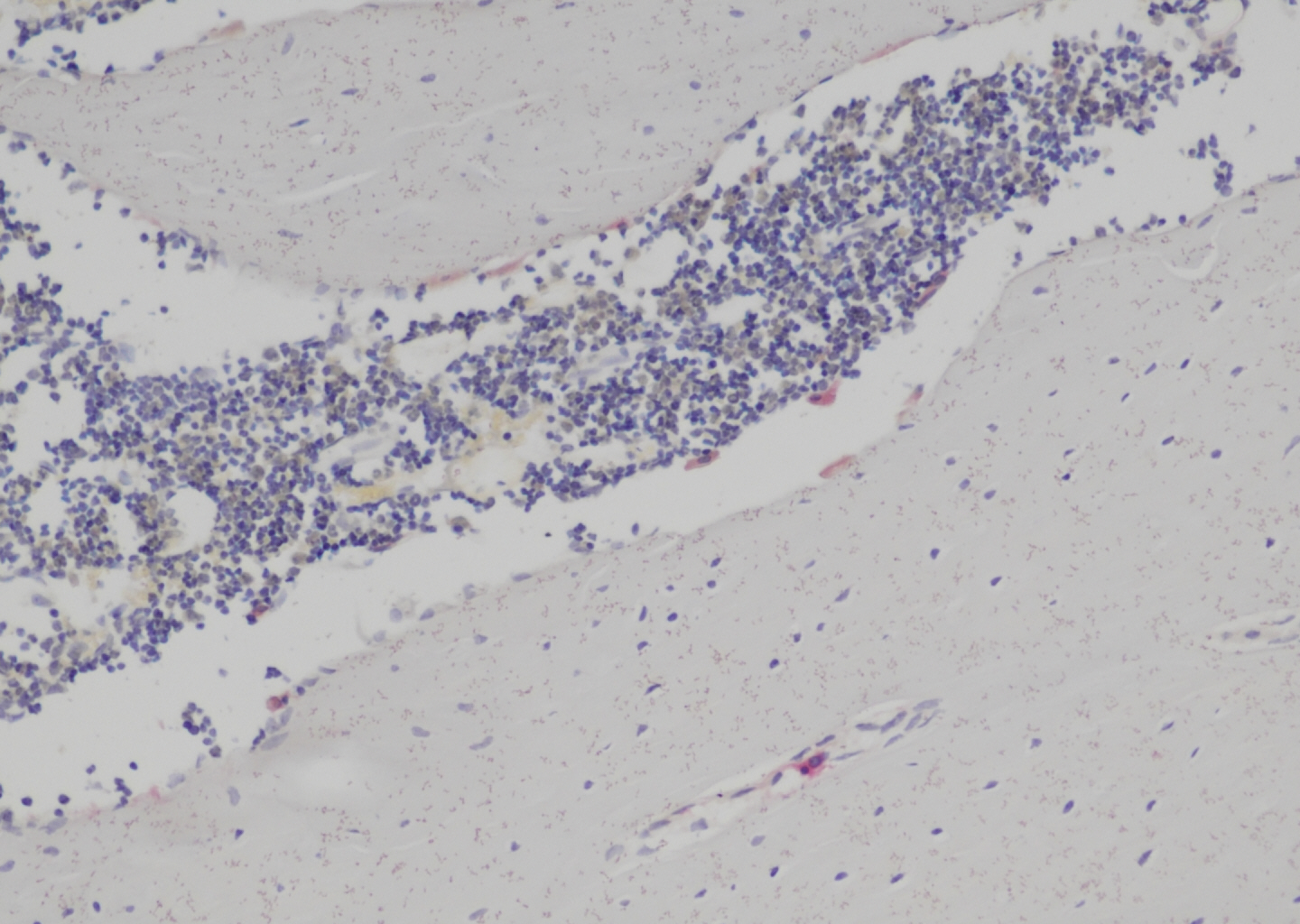

Supplement: Figure 6—source data 1. — The folders named ‘SHAM’, ‘OVX’, ‘OVX-L’, and ‘OVX-H’ contain micrographs of ‘SHAM’, ‘OVX’, ‘OVX-L’, and ‘OVX-H’ groups, respectively. [file elife-64872-fig6-data1.zip › Figure 6-source data 1/OVX-H/OVX-H-5.jpg]

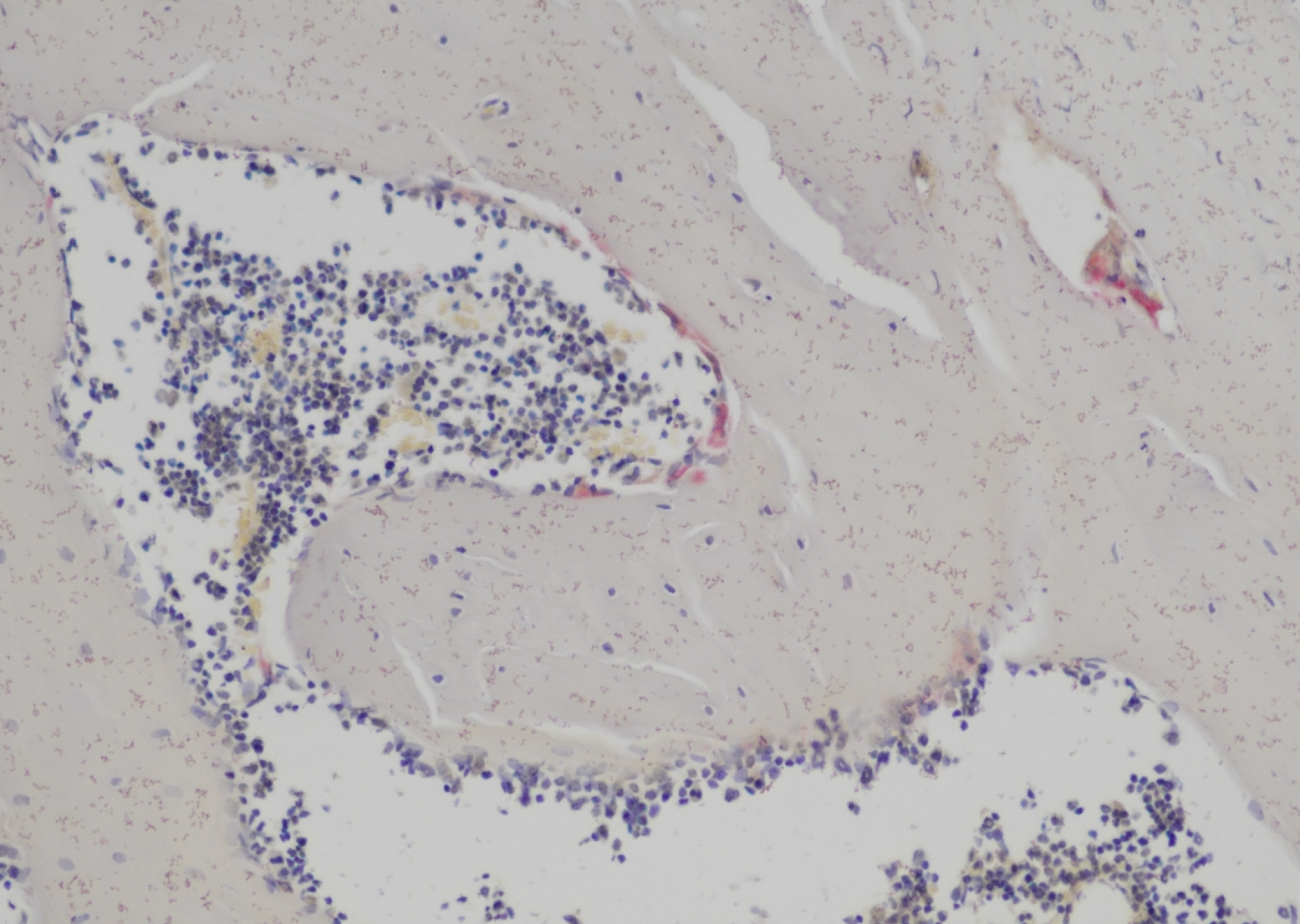

Supplement: Figure 6—source data 1. — The folders named ‘SHAM’, ‘OVX’, ‘OVX-L’, and ‘OVX-H’ contain micrographs of ‘SHAM’, ‘OVX’, ‘OVX-L’, and ‘OVX-H’ groups, respectively. [file elife-64872-fig6-data1.zip › Figure 6-source data 1/OVX-L/OVX-L-1.jpg]

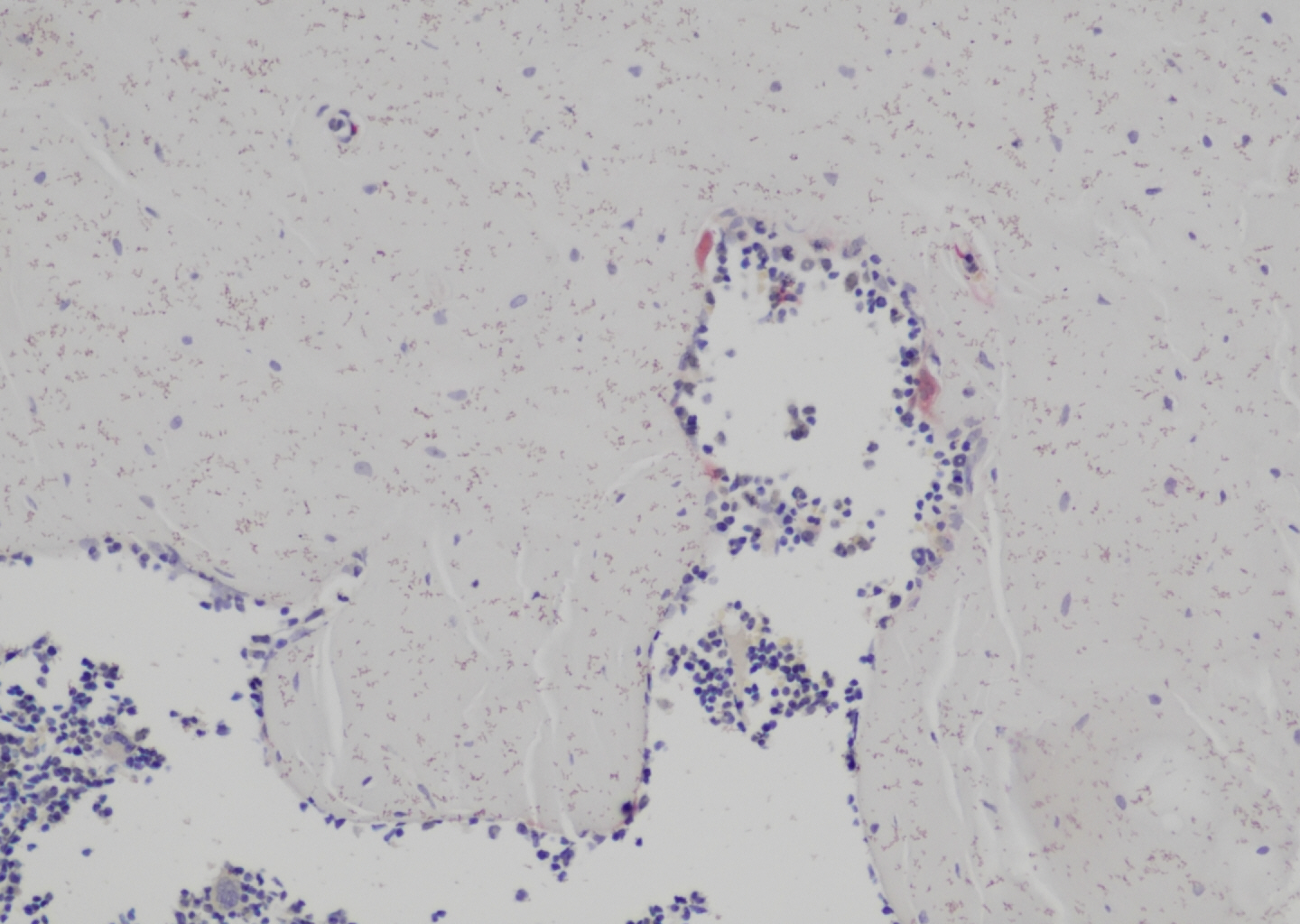

Supplement: Figure 6—source data 1. — The folders named ‘SHAM’, ‘OVX’, ‘OVX-L’, and ‘OVX-H’ contain micrographs of ‘SHAM’, ‘OVX’, ‘OVX-L’, and ‘OVX-H’ groups, respectively. [file elife-64872-fig6-data1.zip › Figure 6-source data 1/OVX-L/OVX-L-2.jpg]

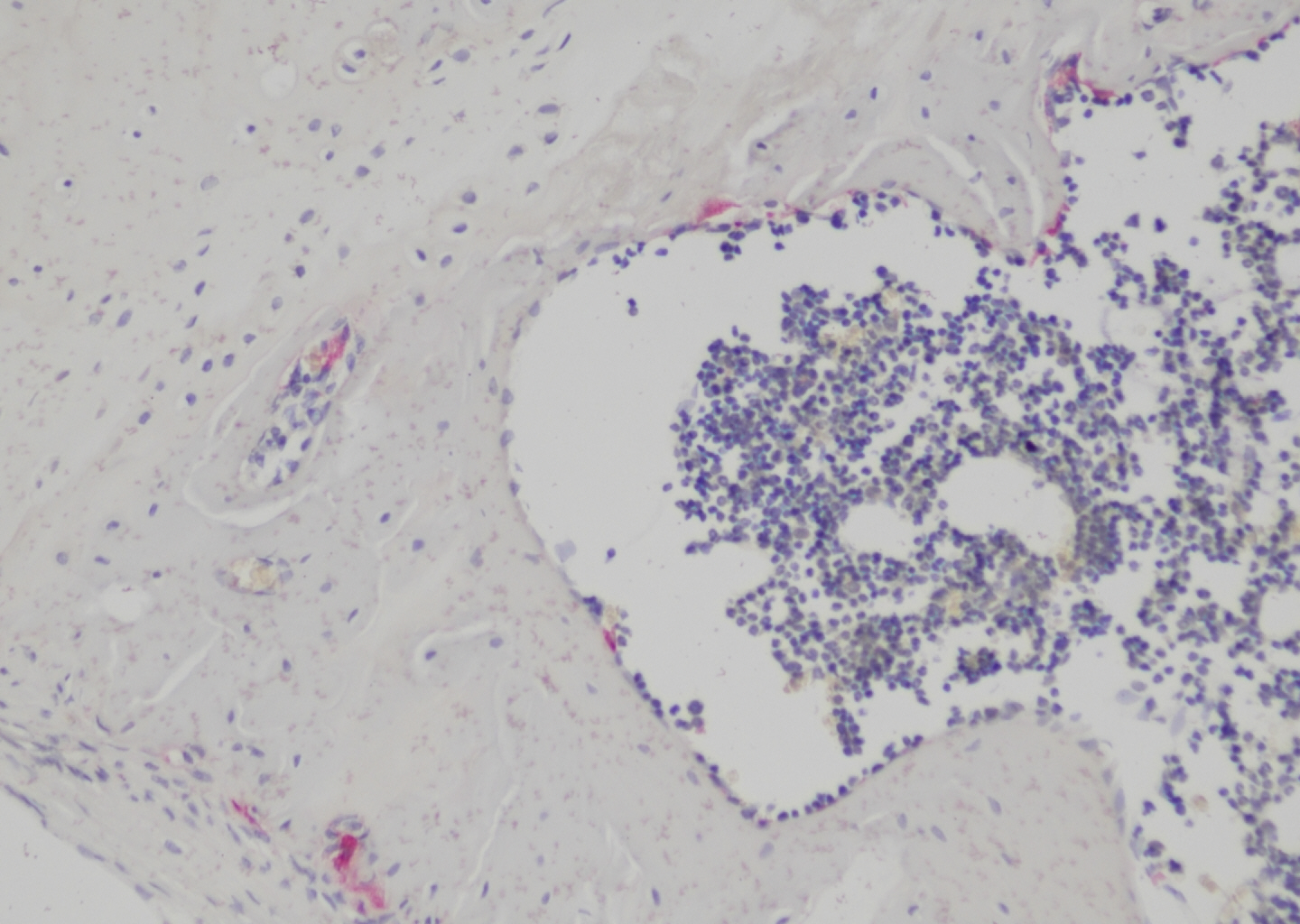

Supplement: Figure 6—source data 1. — The folders named ‘SHAM’, ‘OVX’, ‘OVX-L’, and ‘OVX-H’ contain micrographs of ‘SHAM’, ‘OVX’, ‘OVX-L’, and ‘OVX-H’ groups, respectively. [file elife-64872-fig6-data1.zip › Figure 6-source data 1/OVX-L/OVX-L-3.jpg]

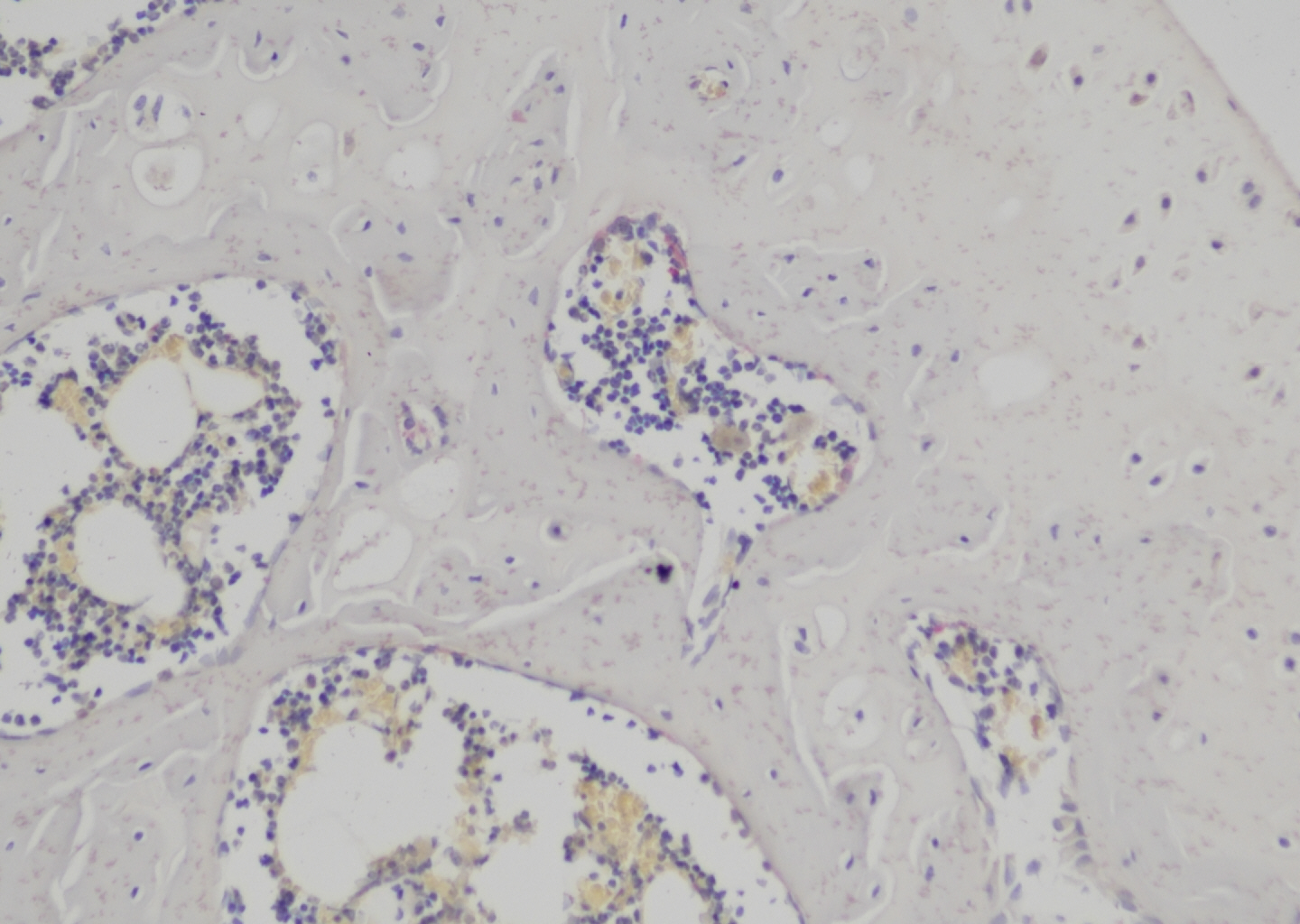

Supplement: Figure 6—source data 1. — The folders named ‘SHAM’, ‘OVX’, ‘OVX-L’, and ‘OVX-H’ contain micrographs of ‘SHAM’, ‘OVX’, ‘OVX-L’, and ‘OVX-H’ groups, respectively. [file elife-64872-fig6-data1.zip › Figure 6-source data 1/OVX-L/OVX-L-4.jpg]

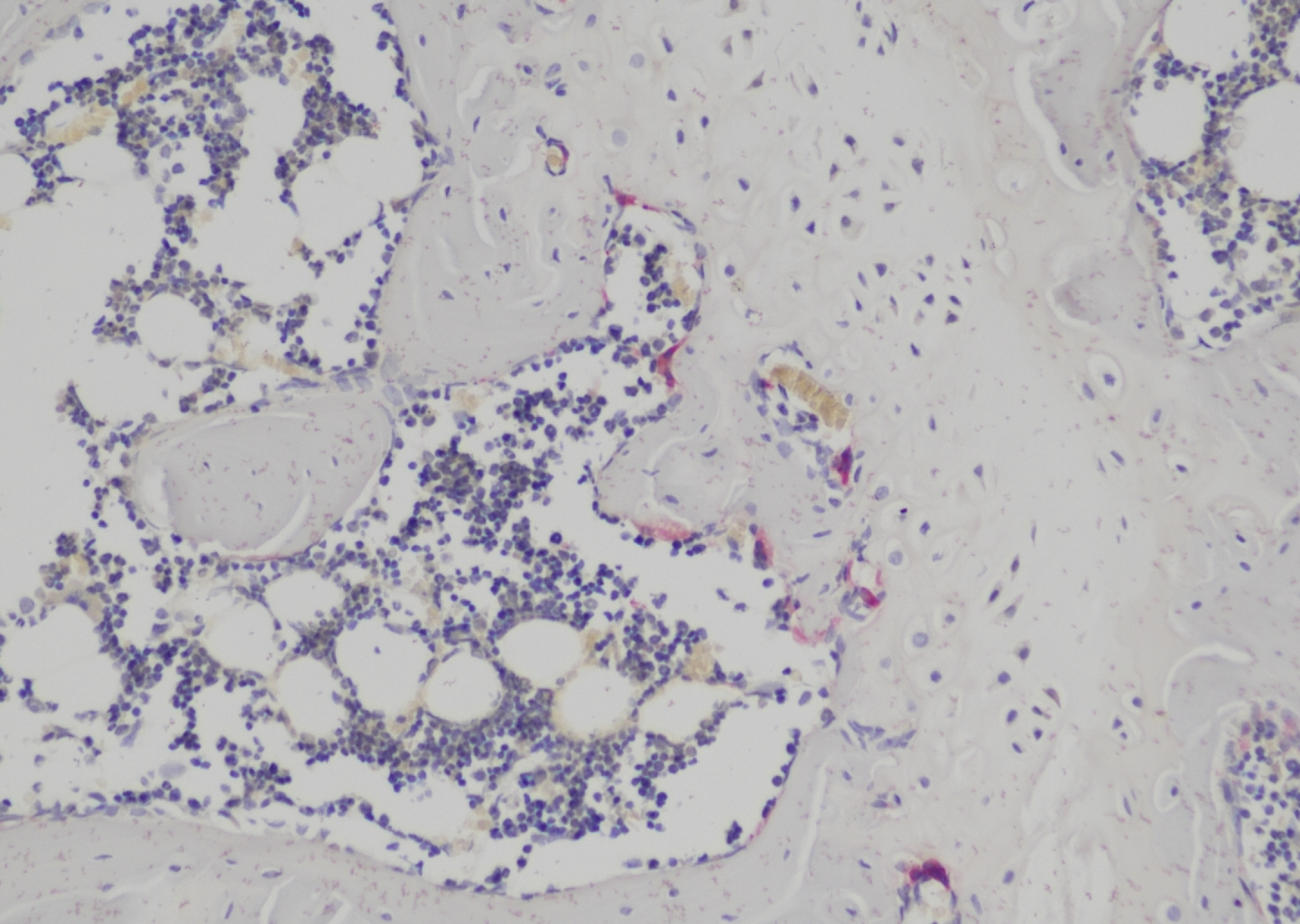

Supplement: Figure 6—source data 1. — The folders named ‘SHAM’, ‘OVX’, ‘OVX-L’, and ‘OVX-H’ contain micrographs of ‘SHAM’, ‘OVX’, ‘OVX-L’, and ‘OVX-H’ groups, respectively. [file elife-64872-fig6-data1.zip › Figure 6-source data 1/OVX-L/OVX-L-5.jpg]

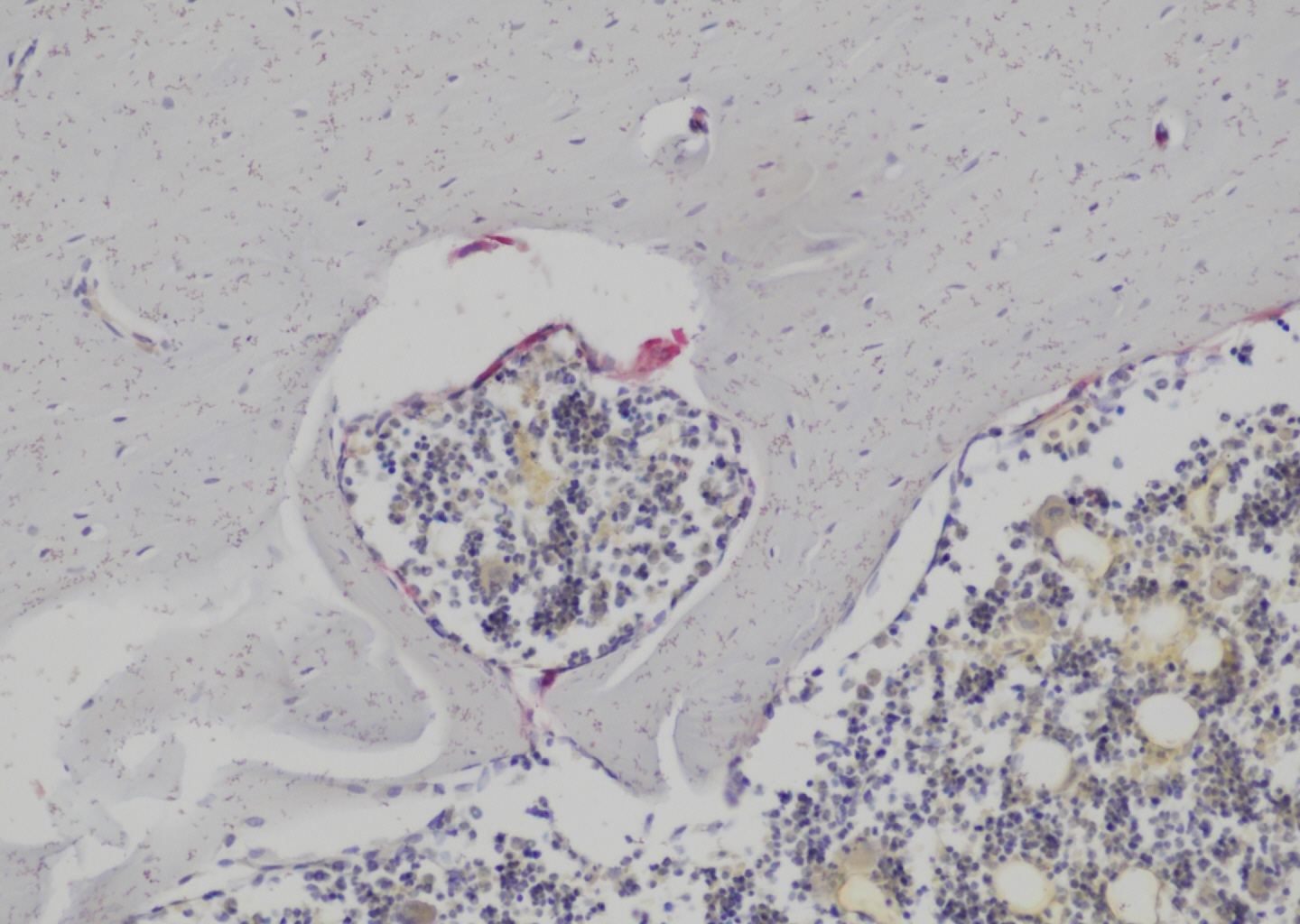

Supplement: Figure 6—source data 1. — The folders named ‘SHAM’, ‘OVX’, ‘OVX-L’, and ‘OVX-H’ contain micrographs of ‘SHAM’, ‘OVX’, ‘OVX-L’, and ‘OVX-H’ groups, respectively. [file elife-64872-fig6-data1.zip › Figure 6-source data 1/SHAM/SHAM-1.jpg]

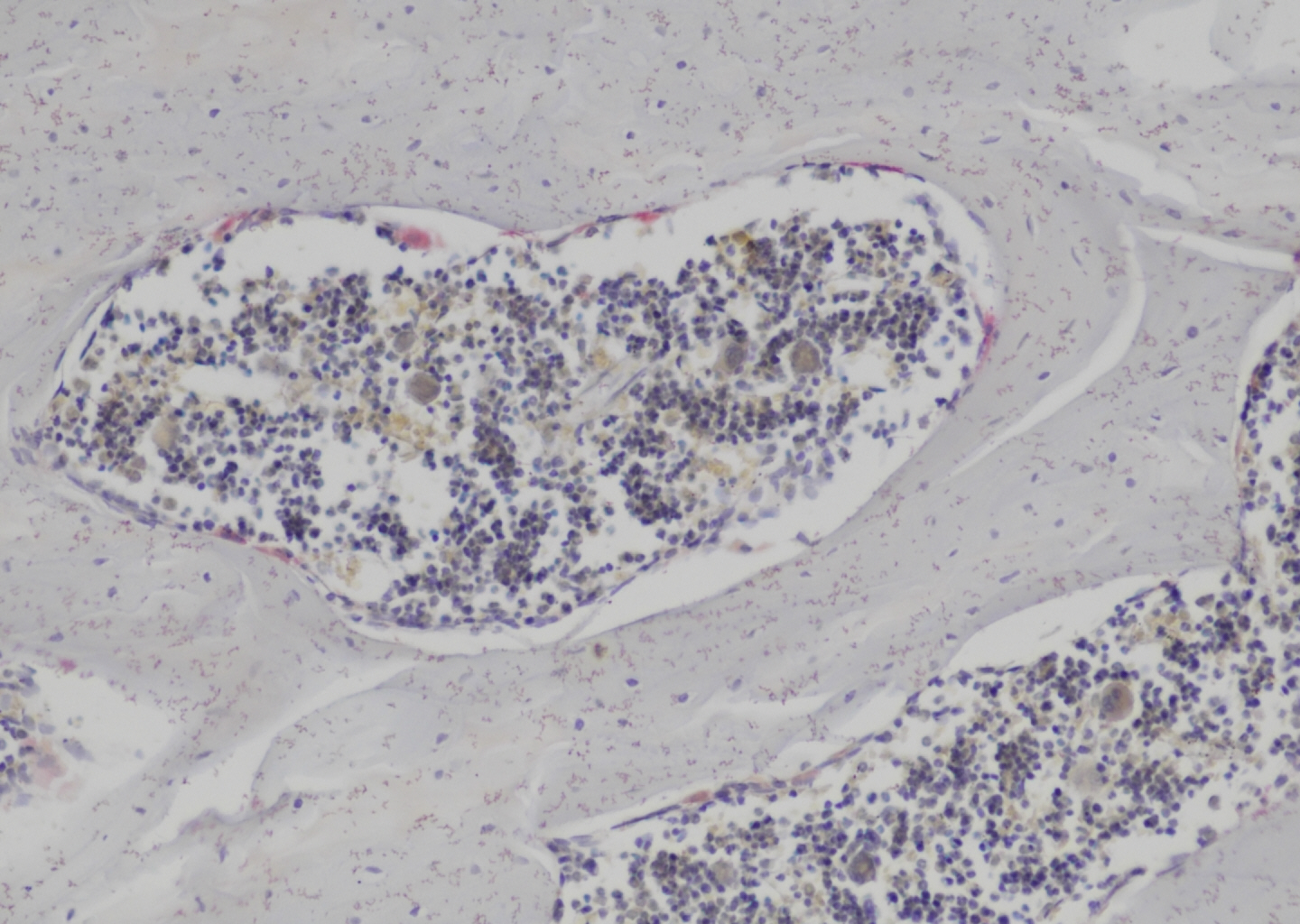

Supplement: Figure 6—source data 1. — The folders named ‘SHAM’, ‘OVX’, ‘OVX-L’, and ‘OVX-H’ contain micrographs of ‘SHAM’, ‘OVX’, ‘OVX-L’, and ‘OVX-H’ groups, respectively. [file elife-64872-fig6-data1.zip › Figure 6-source data 1/SHAM/SHAM-2.jpg]

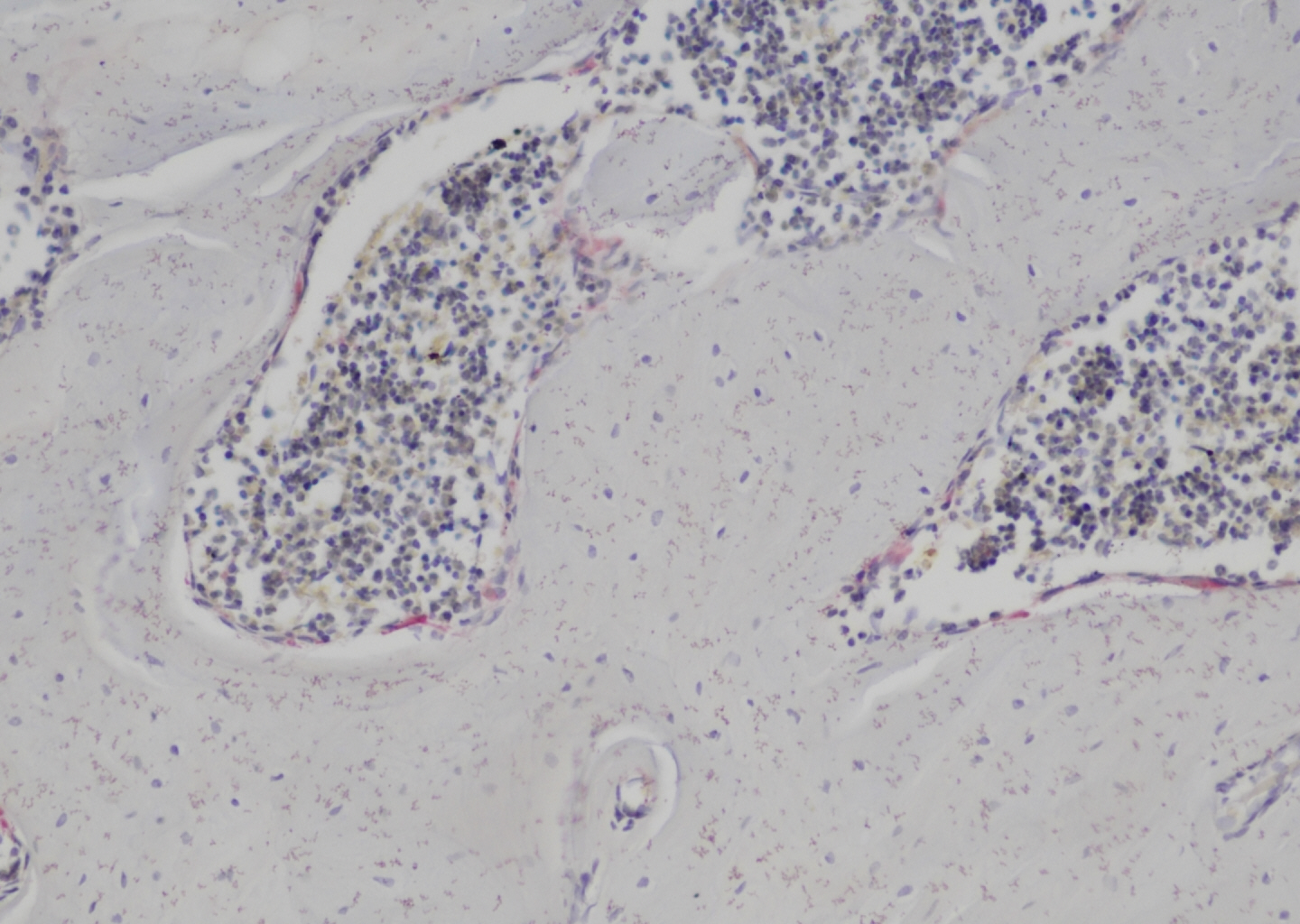

Supplement: Figure 6—source data 1. — The folders named ‘SHAM’, ‘OVX’, ‘OVX-L’, and ‘OVX-H’ contain micrographs of ‘SHAM’, ‘OVX’, ‘OVX-L’, and ‘OVX-H’ groups, respectively. [file elife-64872-fig6-data1.zip › Figure 6-source data 1/SHAM/SHAM-3.jpg]

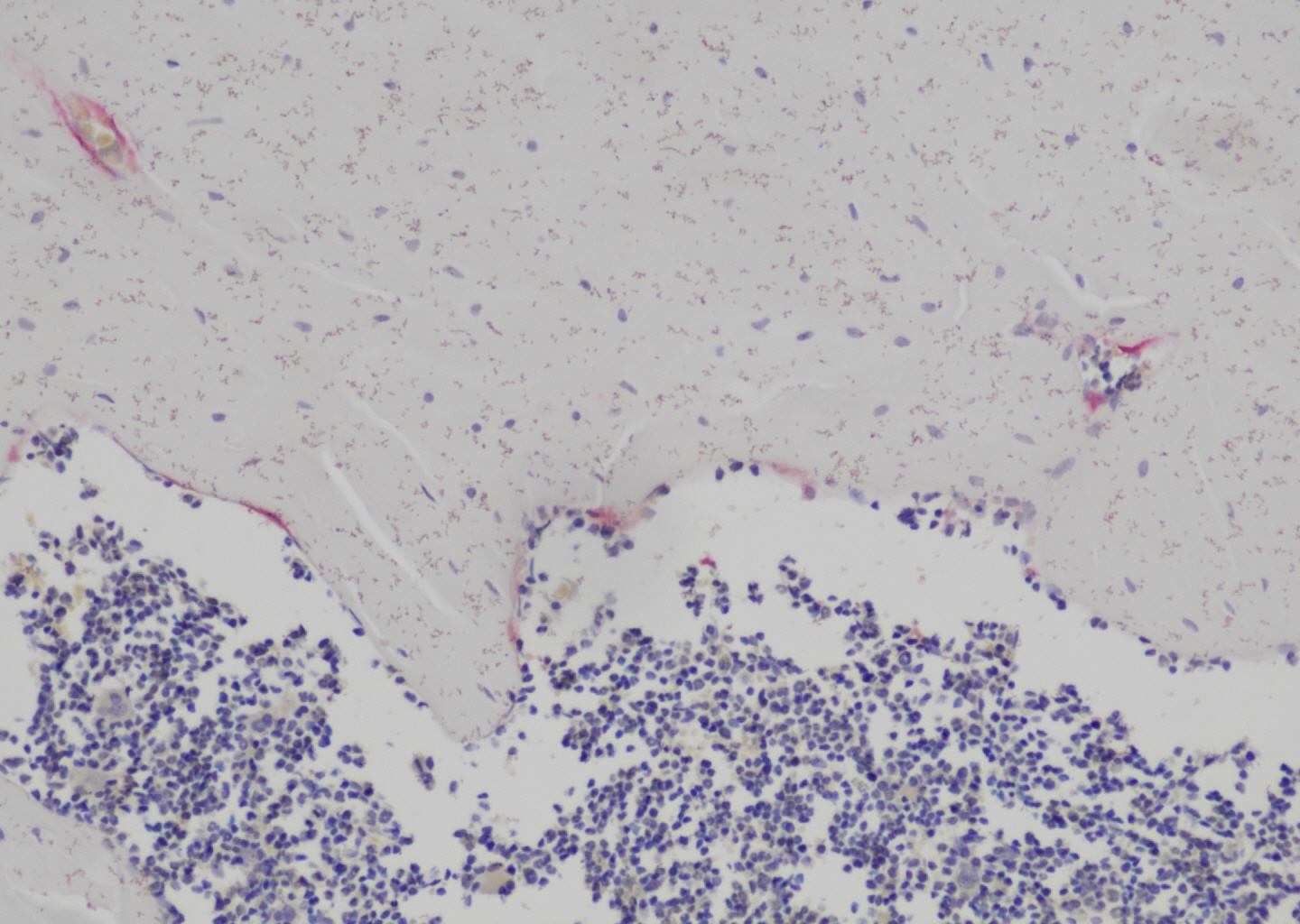

Supplement: Figure 6—source data 1. — The folders named ‘SHAM’, ‘OVX’, ‘OVX-L’, and ‘OVX-H’ contain micrographs of ‘SHAM’, ‘OVX’, ‘OVX-L’, and ‘OVX-H’ groups, respectively. [file elife-64872-fig6-data1.zip › Figure 6-source data 1/SHAM/SHAM-4.jpg]
