# Supplementary material for: 7,8-Dihydroxyflavone modulates bone formation and resorption and ameliorates ovariectomy-induced osteoporosis
Source: eLife. 2021 Jul 6;10:e64872. doi: 10.7554/eLife.64872 (PMC8285109; doi:10.7554/eLife.64872)
Supplement: Supplementary file 1. [file elife-64872-supp1.docx]

***Supplementary File* for**

7,8-dihydroxyflavone Modulates Bone Formation and Resorption and Ameliorates Ovariectomy-Induced Osteoporosis

Fan Xue^1^, Zhenlei Zhao^1^, Yanpei Gu^1^, Jianxin Han^1^, Keqiang Ye^2^*, Ying Zhang^1^*

^1^Department of Food Science and Nutrition, College of Biosystems Engineering and Food Science, Zhejiang Key Laboratory for Agro-Food Processing; Zhejiang Engineering Center for Food Technology and Equipment, Zhejiang University, Hangzhou 310058, China

^2^Department of Pathology and Laboratory Medicine, Emory University School of Medicine, Atlanta, GA 30322, USA

*To whom all correspondence is to be sent.

Ying Zhang, Ph.D., Professor

Postal address: Zhejiang University, 866 Yuhangtang Road, Hangzhou 310058, China

E-mail: yzhang@zju.edu.cn

Fax: +86 571 88982164

Keqiang Ye, Ph.D., Professor

Postal address: Emory University School of Medicine, 615 Michael Street, Atlanta, GA 30322, USA

E-mail: kye@emory.edu

Fax: +404-712-2979

**Supplementary file 1** Sequences of primers used for qRT-PCR

| **Gene** | **Primer Sequence (5’ to 3’)** | |
| --- | --- | --- |
|  | **Forward** | **Reverse** |
| **Cyclin D1** | CAGAGGCGGATGAGAACAAG | GAGGGTGGGTTGGAAATGAA |
| **β-catenin** | GACACCTCCCAAGTCCTTTATG | CTGAGCCCTAGTCATTGCATAC |
| **Runx2** | TCACTACCAGCCACCGAGAC | ACGCCATAGTCCCTCCTTTT |
| **Osterix** | ATGGCGTCCTCTGCTTGAG | AGGACTGCCTGCAGGAGAGA |
| **OPG** | CCAAAGTGAATGCCGAGAGT | ACGCTGCTTTCACAGAGGTC |
| **RANKL** | CCATCGGGTTCCCATAAAGTCA | CAGTTTTTCGTGCTCCCTCCTT |
| **c-fos** | GAATCCGAAGAACGGAATAAG | CAATCTCAGTCTGCAACGCA |
| **GAPDH** | AGTGTTTCCTCGTCCCGTAG | GAAGGGGTCGTTGATGGCAA |
